# Caucasian and Egyptian chitosan/propolis nanocomposites inhibit deformed wing virus in *Apis mellifera* L. cell lines

**DOI:** 10.1038/s41598-026-54534-9

**Published:** 2026-06-04

**Authors:** Heba Seyam, Sameh H. Ismail, Heba M. Hamama

**Affiliations:** 1 Honey Bee Research Department, Plant Protection Research Institute, Agricultural Research Center, Giza, Egypt; 2 Faculty of Postgraduate Studies for Nanotechnology, Cairo University, El-Sheikh Zayed Branch Campus, PO Box 12588, Giza, Egypt; 3 Entomology Department, Faculty of Science, Cairo University, Giza, 12613 Egypt

**Keywords:** Chitosan, Propolis, Nanoparticles, Cell lines, Deformed wing virus, Honey bee, Biochemistry, Biological techniques, Biotechnology, Microbiology

## Abstract

Honey bees face threats from a variety of pathogens and viruses, the most dangerous of which is the deformed wing virus [DWV]. Using natural materials as treatments or nutritional supplements for bees is the ideal solution, especially when transformed into nanoparticles. Propolis is a resinous product collected from honeybee colonies and is known for its wide-ranging therapeutic properties. The nanocomposite of the chitosan/ propolis alcoholic extracts were prepared and characterized for determining their chemical and physical properties. Cytotoxicity assay and antiviral activity of the prepared composites were also determined using cell culture from the head of adult honey bees in a dose–response assay. A quantitative real time-polymerase chain reaction [RT-qPCR] was done to measure their effects on the deformed wing virus load. The characterization results showed that the Caucasian propolis-chitosan nanocomposite have a higher phenols and flavonoid content and surface area compared to the Egyptian propolis. The Caucasian propolis showed higher toxicity to the cultured cells than the Egyptian propolis. The Chitosan-Caucasian propolis nanocomposites caused a more reduction in the deformed wing viral load of the honey bee cell line than the Egyptian one compared to the raw propolis. It is recommended to use the chitosan-Caucasian propolis nanocomposite as therapeutic and nutritional supplements for decreasing the deformed wing viral load in the honey bees a process that may help in increasing their performance and bee-product production.

**Supplementary Information:**

The online version contains supplementary material available at 10.1038/s41598-026-54534-9.

## Background

Honey bees are the main pollinators of diverse crops, and managed Honey bees become increasingly important in securing crop yields, particularly with decreasing diversity and abundance of many wild pollinator species ^1,2^ and the growing demand for food security through agricultural products^3,4^. Honey bee products like honey, the bee venom, the royal jelly, propolis, and bees wax, all have a therapeutic role in the treatment of inflammation, burns, cancer, in different forms in the pharmaceutical industry^5,6^. The population of the honey bee is facing a great challenge with respect to the population decline. Natural habitat loss, overuse of pesticides, climate change, infection with different microorganisms all are considered as factors of the population decline ^7,8^. At least 24 viruses were identified in the honey bees^6,9^. The most common virus infects honey bees is deformed wing virus [DWV]. About 32 countries lost almost half of the honey bee colonies and apiaries due to the DWV infection^10^. Viral infection in cultured cells of newly emerging honey bees was detected by RT-qPCR after virus infection through feeding^11^. Moreover, bee cell culture using L-15 medium breaks a deadlock that has hampered efforts for virus isolation and screening as a step in determination of the viral loads within the cultured cell sheets. Also, to measure the cell viability that may influence or cause colony collapse disorder in the colonies of honey bees^6,12^. So, honey bee-derived cell lines would offer a valuable tool for testing antiviral medicines for usage in the hive. There are no commercially accessible chemical therapies for viral infections in honey bee hives^6^.

Currently, there are natural methods to control the loss of honeybee colonies via application of organic acids such as lactic, formic, and oxalic acids, Also, essential oils as thymol, menthol and carvacrol and propolis proved an efficient role in bee protection^7,13–16^ Propolis is a natural component in the bee colonies having a strong acaricidal activity^17^. It is a resinous mixture having a fundamental role for the in sealing and sterilization of the bee hives for arresting the dissemination of the microbial diseases, such as bacteria, viruses, and fungi^18^. In addition, propolis has indirect and direct effects on bees’ immune response against pathogens and parasites hence improving the colony health and its resiliency^18^. Also, propolis is a tool of social immunity; a behavior defense in social insects that cooperatively results in hive protection against parasites after increasing the hovering rate over resinous plants for more propolis production^18^. The side effect of elevated bees’ immune response is the reduction in colony productivity, as it is considered a costly physiological effect. Nests with a propolis envelope are less vulnerable to attack by parasites and pathogens with less stress to face hence it does not suppress the insect immune system, but offers more protection. Moreover, it has been used in traditional medicine and is recognized to have benefits on human health^19^. Considering the chemical composition of propolis using the gas chromatography-mass spectrometry (GC–MS), red propolis contains an array of active ingredients, methyl eugenol (13.1%), (E)-β-farnesene (2.5%), δ-amorphene (2.3%), αcubebene (1.9%), and β-caryophyllene (1.5%) as the major components of the extract^20^ and these active components usually used in medical industry for used for Antimicrobial, anti-inflammatory, cytotoxic, and antiparasitic activities, as well as immunomodulatory and leishmanicidal effects^20^. Also, Egyptian propolis contains methyl gallate and phethalic acid that contribute to enhancing the cellular activities as well as providing an antiviral effects^6^. Generally speaking, the chemical analyses of propolis identified nearly 30 compounds, including flavones, flavonols, simple phenols, aglycones and conjugates aglycones. The most abundant compounds in propolis were kaempferol and its homologues, accounting for almost 2 g kg^−1^ of extract, whereas total phenols amounted to almost 120 g kg^−1^ and theses components used as pesticides^15^. Propolis application to brood cells affects the parasites reproduction showing a potential impact on *Varroa* population, so it can be regarded as a natural pesticide used by the honeybee to limit the dangerous parasite^15,18^.

Nanotechnology has provided a promising era in the agricultural, biomedical, and pharmaceutical applications regarding providing better bioavailability and penetrative capacity hence higher therapeutic efficacy^21^. The enhanced physicochemical properties such as particles size, charge, surface structure, and others properties all help in enhancing the encapsulation efficiency and the controlled drug release, all support the applications of chitosan-based nanocomposites ^21–23^.

Synthesis of nanopropolis has increased the antimicrobial efficacy than the native form^24,25^. In addition, the propolis extract was proved to increase the antimicrobial activity of chitosan^26^. Prior to application of natural materials and transforming them into nano-forms, study of the chemical and physical characterization of their changes is carried out by using FTIR, surface area properties, Zeta potential, GC–MS, HPLC, XRD , DLS, AFM, TEM, SEM, and BET instruments^27–29^.

This study aims at improving the honeybee viral infection therapy by the preparation and characterization of chitosan/propolis nano-particles using two different-origin propolis. The Egyptian and the Caucasian propolis were tested for their antiviral activity compared with chitosan separately. Results of the present study may lead to enhancing and raising the activity of honeybee hives by inhibiting the deformed wing virus in infected Honey bees.

## Materials and methods

### Preparation of chitosan/propolis nanoparticles

Raw Caucasian propolis samples were sourced from verified apiaries located in the Caucasus region [43° N, 42° E] and Egyptian propolis samples were obtained from Upper Egypt [26° N, 32° E] the Egyptian propolis was collected from the Beheira Governorate 30° 37′ N 30° 26′ E / 30.61° N 30.43° E / 30.61; 30.43; the propolis was collected from more than 100 hybrid Carniolian bee colonies. Analytical grade ethanol [99.9%, Sigma-Aldrich] was employed as the primary solvent. Ultrapure water [18.2 MΩ·cm] was obtained using a Milli-Q system [Millipore, USA]. All additional reagents used in the study were of analytical grade and utilized without further purification steps. The ultrasonic synthesis was performed using a Hielscher UP200St processor [Germany] equipped with a titanium sonotrode [diameter: 14 mm].

Raw propolis samples [100 g] were mechanically reduced to small pieces [approximately 2–3 mm in diameter]. The fragmented propolis was combined with 70% ethanol in a 1:10 weight-to-volume ratio in amber glass containers. The extraction proceeded under continuous stirring conditions at 150 rpm for 72 h at room temperature [25 ± 2 °C] in complete darkness. The resulting mixture underwent primary filtration through Whatman No. 1 filter paper to remove coarse particles. Secondary purification was achieved through centrifugation at 4000 rpm for 15 min. The collected purified supernatant was concentrated under reduced pressure at 40 °C until a solid content of 60% was achieved using a rotary evaporator^30,31^. The ethanolic propolis extract [EP] was then dried on a hot plate at 60 °C, and the dried solid extract was ready to use.

The concentrated propolis extract was dissolved in analytical grade ethanol to a final concentration of 10 mg/mL. The solution was maintained under constant stirring at 100 rpm for 30 min to ensure complete dissolution. The prepared solution was filtered through a 0.45 μm membrane filter to remove any undissolved particles.

#### Synthesis of Egyptian/Caucasian chitosan/propolis nano-capsules

**Fig. 1:**
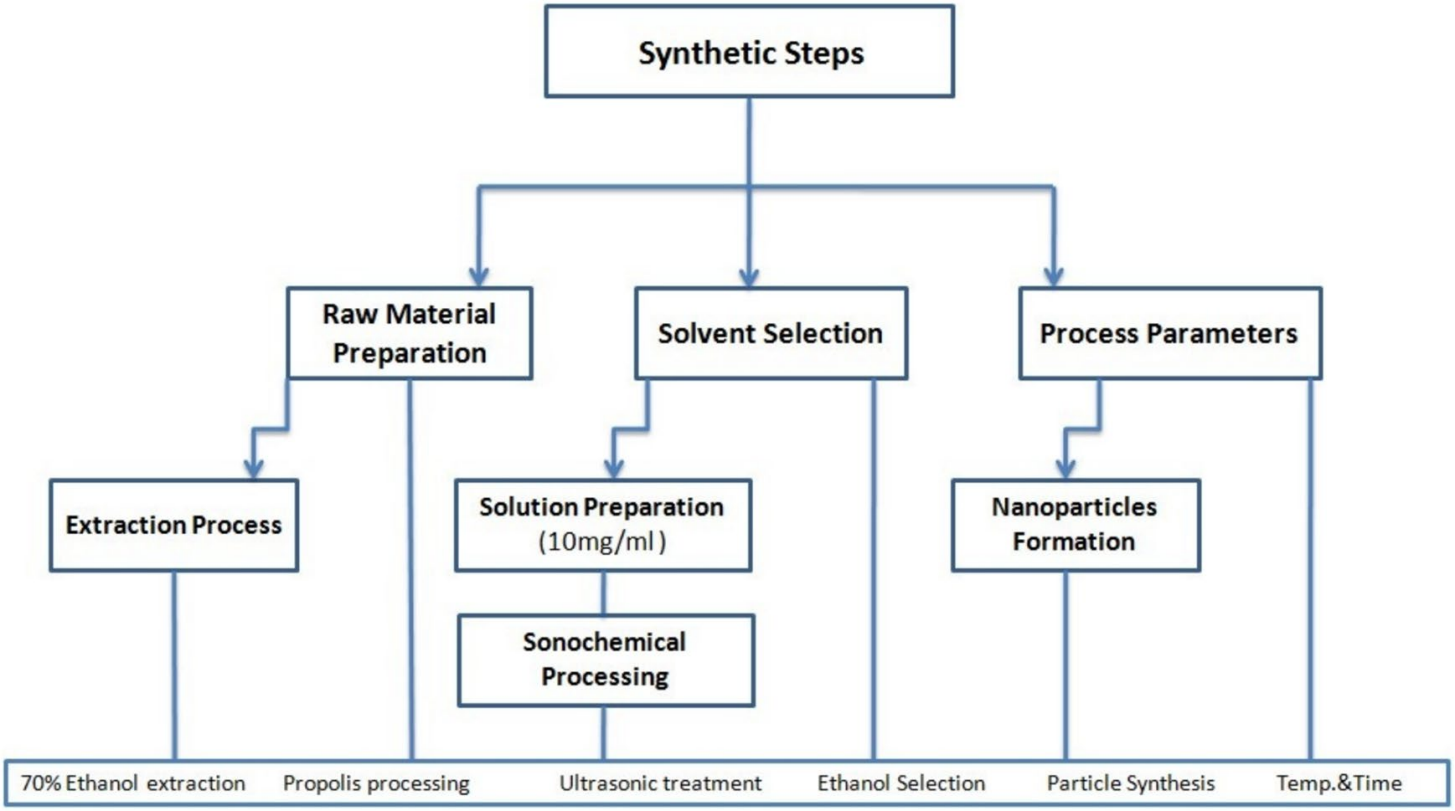
Preparation and synthesis Nanoparticles for both types of propolis.

Chitosan, a linear polycationic polysaccharide derived from the deacetylation of chitin, is dissolved in a 2% acetic acid solution to obtain a homogeneous chitosan solution. The dried propolis extract, ready for the ionic gelation method, is employed to encapsulate the Egyptian/Caucasian propolis extract [100 ml] within the chitosan matrix. In this process, the chitosan solution [5 g/100 ml doubled deionized water] is added dropwise to an aqueous solution of sodium tripolyphosphate [STPP] [5 g/100 ml 5 g/100 ml doubled deionized water] under continuous magnetic stirring. The resulting Egyptian/Caucasian propolis-chitosan nano-capsules consist of an Egyptian/Caucasian propolis extract core encapsulated within a cross-linked chitosan polymer shell. The nano-encapsulation process using STPP as a cross-linking agent offers several advantages, including increased stability, controlled release properties, and improved bioavailability of the encapsulated propolis components. The nanoparticle suspensions were subjected to centrifugation at 12,000 rpm for 15 min at 4 °C to separate any aggregates. The supernatant containing the stable nanoparticles was collected and filtered through a 0.22 μm sterile filter. The purified nanoparticle suspension was stored at 4 °C in amber glass vials for further characterization and analysis Fig. 1.

#### Process optimization

The synthesis parameters were systematically optimized to achieve optimal nanoparticle characteristics. Power density was evaluated across 50–150 W/cm^2^ at 25 W/cm^2^ intervals. Sonication duration was tested between 15 and 45 min at 5-min intervals. Extract concentration studies were conducted from 5–15 mg/mL at 2.5 mg/mL increments. Temperature effects were assessed between 20–30 °C at 2 °C intervals. Each parameter was optimized independently while maintaining all other variables constant. The optimal conditions were determined based on particle size distribution, stability, and morphological characteristics.

### Characterization methods

The characterization of Egyptian/Caucasian chitosan/propolis nano-capsules can be classified into three classes: identification, morphology, and index, as described by Ismail et al.^27,28^ and El-Hefnawy et al.^29^.

#### Identification analysis

##### X-ray diffraction [XRD] analysis

The crystalline structure and phase identification of both Caucasian and Egyptian propolis nanoparticles were analyzed using an X-ray diffractometer [Rigaku SmartLab, Japan] equipped with Cu Kα radiation [λ = 1.5406 Å]. The analysis was conducted at room temperature with a scanning rate of 2° min⁻^1^ over the 2θ range of 10–80°. The operating voltage and current were maintained at 40 kV and 40 mA, respectively. The crystallite size was calculated using the Debye–Scherrer equation according to Debye and Scherrer^32^.

##### UV–visible spectroscopy

UV–visible spectroscopic analysis was performed using a double-beam spectrophotometer [Shimadzu UV-2600, Japan] in the wavelength range of 200–800 nm. To maintain absorbance within the linear range, the nanoparticle suspensions were diluted appropriately with ultrapure water. Measurements were conducted using quartz cuvettes with a path length of 1 cm, with ultrapure water serving as the reference blank.

#### Morphological analysis

##### Transmission electron microscopy [TEM]

The internal structure and morphology of the nanoparticles were examined using a high-resolution transmission electron microscope [JEOL JEM-2100F, Japan] operating at an acceleration voltage of 200 kV. Sample preparation involved placing a drop of diluted nanoparticle suspension on a carbon-coated copper grid [300 mesh] and allowing it to air dry at room temperature. Images were captured at various magnifications to analyze particle size distribution and morphological features.

##### Scanning electron microscopy [SEM]

Surface morphology and external features were investigated using a field emission scanning electron microscope [FEI Quanta 450, USA] operating at 20 kV. For Sample preparation, the nanoparticle suspension was deposited on silicon wafers and allowing them to dry under ambient conditions. A thin layer of gold [approximately 5 nm] was sputter-coated on the samples to enhance conductivity and image quality.

##### Atomic force microscopy [AFM]

Three-dimensional surface topography was analyzed using an atomic force microscope [Bruker Dimension Icon, USA] operating in tapping mode. Silicon cantilevers with a resonance frequency of 300 kHz and spring constant of 40 N/m were employed. Samples were prepared by depositing diluted nanoparticle suspensions on freshly cleaved mica surfaces. Images were captured at different scan sizes [2 µm × 2 µm and 5 µm × 5 µm] with a scan rate of 0.5 Hz.

#### Physical properties analysis

##### Dynamic light scattering [DLS]

Polydispersity index and particle size distribution were measured using a dynamic light scattering analyzer [Malvern Zetasizer Nano ZS, UK] equipped with a 4 mW He–Ne laser [λ = 633 nm]. Measurements were performed at 25 °C with a scattering angle of 173°. Samples were appropriately diluted with ultrapure water and filtered through a 0.22 µm membrane before analysis. Each measurement was repeated three times with 12 runs per measurement.

##### Zeta potential analysis

Surface charge characteristics were evaluated using the same Malvern Zetasizer Nano ZS instrument. Measurements were conducted at 25 °C using folded capillary cells [DTS1070]. The zeta potential was calculated from electrophoretic mobility using the Smoluchowski equation by Smoluchowski^33^. pH-dependent measurements were performed over a range of 4–10 using 0.1 M HCl and 0.1 M NaOH for pH adjustment.

##### BET surface area analysis

Specific surface area and porosity characteristics were determined using a surface area analyzer [Micromeritics ASAP 2020 Plus, USA] based on nitrogen adsorption–desorption isotherms at -196 °C. Samples were degassed at 40 °C for 24 h under vacuum before analysis. The specific surface area was calculated using the Brunauer–Emmett–Teller [BET] method according to Brunauer et al.^34^, while the pore size distribution was determined using the Barrett-Joyner-Halenda [BJH] method by Barrett et al.^35^.

#### Density and aggregation [DA] analysis

Density measurements and aggregation behavior were studied using an analytical ultracentrifuge [Beckman Coulter ProteomeLab XL-I, USA] equipped with Rayleigh interference optics. Sedimentation velocity experiments were conducted at 40,000 rpm and 20 °C. The data were analyzed using SEDFIT software to determine the sedimentation coefficient distribution and assess the degree of aggregation [https://sedfitsedphat.github.io ]. All measurements were performed in triplicate, and the results are presented as mean values with standard deviations. Statistical analysis was conducted using one-way ANOVA with Tukey’s post-hoc test, with *p* < 0.05 considered statistically significant.

### Detection of the honey bee deformed wing virus [DWV] infection by RT-PCR

#### Honey bee samples collection and RNA extraction

Asymptomatic adult workers were randomly collected with tweezers in the summer season from different collapsing colonies. These colonies were characterized by having poor propolis production from widely separated apiaries from the Department of honey bee research, Plant protection research institute, Agricultural Research Center, Giza, Egypt. Collected adult worker samples were kept in cold conditions during sampling and then were stored immediately at − 70 °C.

Total RNA [TRNA] was extracted from 10 pooled heads of adult worker bees using BIOZOL-BIOFLUX commercial kit for extraction [bioWORLD, USA, catalog No.10760055–1] following the manufacturer’s instructions. The quality and quantity of RNA were analyzed and quantified using NanoDrop 2000 [Thermo Scientific, USA]. TRNAs were then conserved at − 20 °C.

#### Reverse transcription and conventional PCR [RT-PCR]

Complementary DNA [cDNA] synthesis was performed using Qiagen Kit [Qiagen, USA, cat. No.330401] used 0.5 µM of specific gene primers, and 0.61 µg of RNA that were added to each reaction then following the manufacturer’s protocol. RT-PCR was performed using specific primer pairs for deformed wing virus [DWV], which were synthesized by ThermoFisher Scientific [Invitrogen] Forward primer sequence [CTG TAT GTG GTT GCC TGG T] and Rev. [TTC AAA CAA TCC GTG AAT ATA GTG] according to Kukielka et al.^36^. RT-PCR reactions were carried out in 50ul mixture and were run in the thermal cycle TechneGene Amp. [PCR system FGENO2TD, England]. Gradient PCR was performed using the following parameters, an initial denaturation cycle at 94 °C for 3 min, followed by 35 cycles at 94 °C for 45 Sec, 55 °C for 1 min, followed by a final extension step at 72 °C for 7 min. Following Ethidium bromide staining [Sigma Aldrich, USA], PCR products were electrophoresed on a 1.5% agarose gel and observed with a UV trans-illuminator [CUV Pupland A, USA]. PCR amplicon generated from DWV was subsequently purified using a PCR Purification Kit ExoSAP-IT [Affymetrix, USA], following the manufacturer’s instructions. Big Dye Terminator v3.1 Cycle Sequencing [Applied Biosystems, CA, USA] was used to execute the sequencing procedures, and the 3130XL genetic analyzer was used to examine the results [Applied Biosystems, CA, USA].

To confirm the identification of the amplified virus, sequence data was gathered and analyzed using Bioinformatics tools used for sequence analyses and alignments included NCBI-BLAST tool [http://blast.ncbi.nlm.nih.gov/Blast.cgi] and BioEdit software version 7.0.0^37^.

### Primary culture and cytotoxicity of nanoprticles

#### Preparation of samples for primary cell culture

The collected honey bee samples from colonies with poor propolis and in the winter season were initially examined by RT-PCR for viral infection. The virus infection was detected in the heads of adult worker, indicating that the samples were naturally infected with the studied DWV and used as also positive control for infected samples. Since there were no virus-infected, commercial honey bee cell lines^6,38^, hence we employed two groups of virus infected cultured cells from colonies with poor propolis in the current study: one group served as a positive control [untreated cells], and the other group [treated cells] detect the antiviral activity of the prepared ethanolic propolis [EP] and the nanoparticles for both Egyptian and Caucasian propolis. 10 pooled heads of adult honey bee workers were taken from the same group of asymptomatic honey bee colonies, then the heads samples were crushed in 2 mL of the sterile filtered media and the media containing the cells were evenly dispersed to each positive control [untreated cells] and treated plate [with both Nanoparticles of EP; Egyptian and Caucasian types]. Untreated cells were used as a positive control cell for determining the first titer of the virus investigated, as a result, untreated [Positive control] cells were cultured without any therapies, while others were cultured by adding 5 mg/ml of Nano-EP to the cells, each treatment and concentration having its plate. The treated and untreated [positive control] cells were cultured in 96-well dishes [Nunc™, ThermoFisher, Scientific].

#### Cell cultures

Ten pooled adult worker heads were crushed in a sterile mortar with 2 mL of culture medium to release the cells in the culture media [Leibovitz’s L15 Sigma-Aldrich, Cat. No: L4386, with L-glutamine and Fetal bovine serum [10% FBS] according to Goblirsch et al.^12^. Samples were centrifuged at 2000 rpm for 10 min after removing the debris. The supernatants were removed and the cells were resuspended in 200 µL of culture medium in 96 wells of cell culture plate [Nunc™, ThermoFisher, Scientific] for heads of adult worker honey bees. All cell cultures were incubated at 34 °C. The subcultures was performed after the formation of confluent cell culture sheets which were examined under an inverted microscope [Helmut Hund GmbH, Wetzlar, Germany].

#### Cytotoxicity assay

The MTT assay was performed using different concentrations [from 1 to 12 mg/ml] of [chitosan/propolis nanocapsule] extracts from both the Egyptian and Caucasian propolis to study their effects on the viability and proliferation of the cells cultured from the heads of adult worker Honey bees^39,40^. The cells were sown in 100 µL of complete culture media on 96-well tissue culture plates [NuncTM, ThermoFisher Scientific] and incubated at 34ºC for 24 h. An inverted microscope was used to study and record morphological changes and cellular damage [Helmut HundGmbH, Wetzlar, Germany]. After the cells had been cultured for 24 h, 25 L of MTT solution [5 mg/mL, Cat.No.M6494] was added to each well and incubated for another 4 h. The supernatant was carefully removed and 150 L of dimethyl sulfoxide [DMSO] were added. Using a LERX800 Biotek-USA ELISA reader, the optical density [OD] was measured at 540 nm. The following formula was used to compute the cell viability percent [%]. [The viability % 1/4 OD of treated cells/OD of untreated cells [positive control] × 100] Using MasterPlex 2010 software [MiraiBio, Hitachi Solutions America, Ltd], the median inhibitory concentrations [IC_50_ values] were obtained by fitting the survival curve.

#### Antiviral assay

Cell culture media were filtered by MS® sterile syringe filter 0.22 µm [Lot. No. 280651471]. An antiviral assay was conducted to determine the antiviral activity level of three different concentrations that had no cytotoxic effects on the honeybee cells from nanoparticles of both EP types with concentration [5 mg/ml]. Both propolis extracts were added to the cell culture as a supplement and viral treatment in comparison to the untreated [positive control] cells and using 5mgp/ml of chitosan as a treat also and a reference of nanoparicles. The untreated cells are used as a positive control based on the results of PCR, which proved their natural multiple viral infections.

### Virus quantification using RT-qPCR

RNA extraction from untreated [positive control] and treated cell cultures were performed using BIOZOL-BIOFLUX [Catalog No.10760055–1] following the manufacturer’s instructions .Quality and quantity of RNA were measured using Gel Electrophoresis 1.5% and NanoDrop 2000 [Thermo Scientific, USA]The cDNA synthesis was performed using RT^2^ First Strand Kit, [Qiagen , Cat. No.330401] with 0.56 µg RNA for each reaction from untreated [positive control] and treated cell cultures following the manufacturer’s protocol but the RT primer mix in the kit was replaced by the specific gene primer [0.5 µM] for deformed wing virus [DWV], which were synthesized by ThermoFisher Scientific [Invitrogen] Fwd. [CTG TAT GTG GTT GCC TGG T] and Rev. [TTC AAA CAA TCC GTG AAT ATA GTG] in accession number DQ38550^36^ and B-actin using as a reference gene for normalization^41^ FWD. primer [TGCCAACACTGTCCTTTCTG] and REV. primer [AGAATTGACCCACCAATCCA].

#### Gene expression analysis using real-time PCR [RT-qPCR]

Following the manufacturer’s instructions, RT-qPCR reactions were done using the Quanti-Tect SYBR® Green PCR kit [Qiagen, Valencia, CA]. PCR reactions were incubated at 95 °C for 2 min in the CFX96TM Real-time PCR Detection System using SsoFastTM Eva Green® supermix [BioRad], followed by 40 cycles of 95 °C of denaturation for 15 s and annealing at 60 °C for 10 s and then 72 °C for 10 s. In a total of 25 μL for each reaction, 20 μM of each primer and 2 μL of cDNA as a template were used in triplicate to compare the level of viral protein from the cell culture before and after treatments.

### Statistical analysis

Results were expressed as the mean ± standard error [SE]. Statistical significance between different samples of untreated and treated honeybee colonies was analyzed using one-way ANOVA. Statistical significance was defined as *P* < 0.01 and *P* < 0.001.

## Results

### Identification analysis

#### X-ray diffraction [XRD] analysis

**Fig. 2:**
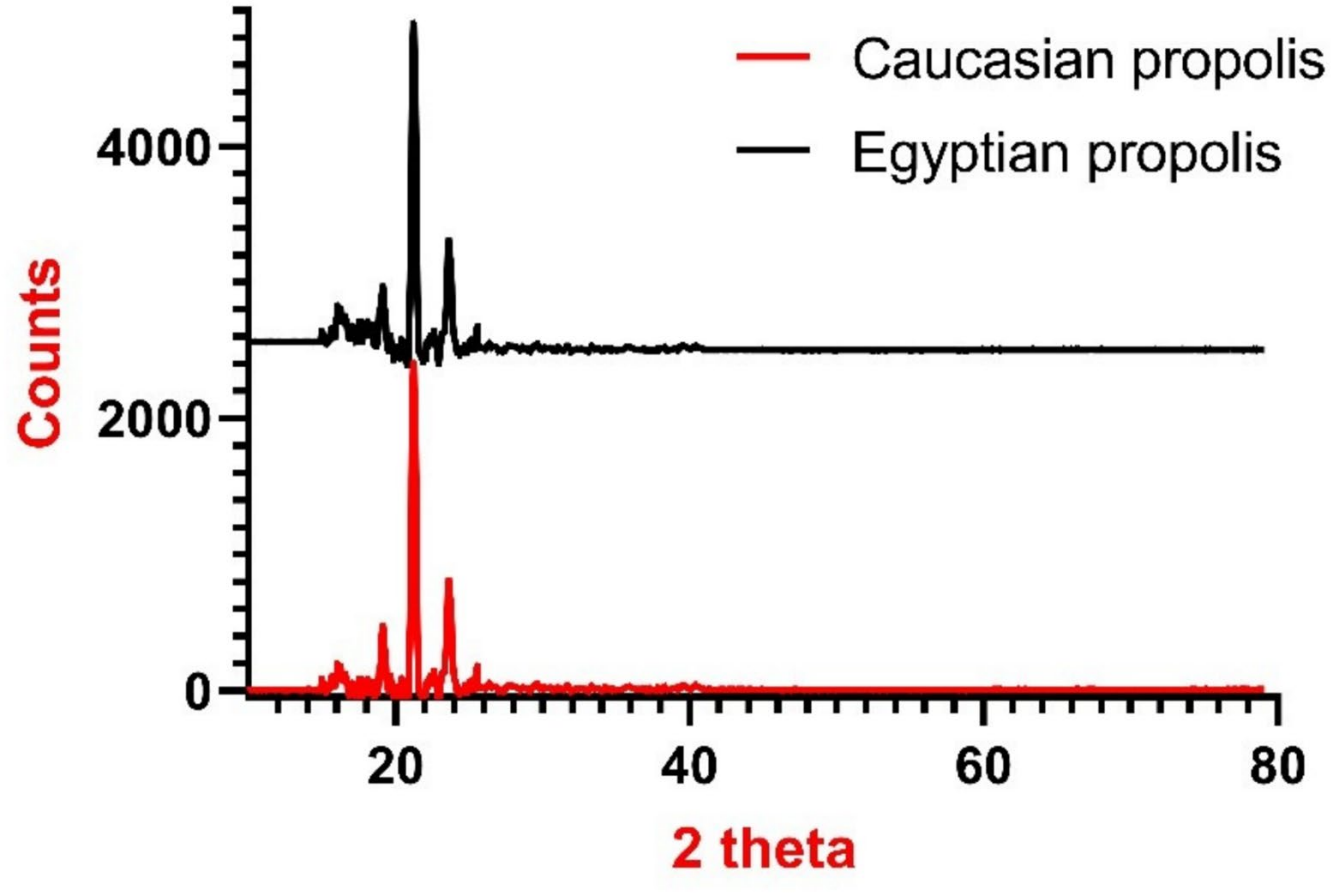
XRD Pattern of the Egyptian and the Caucasian Propolis Nanoparticles.

The X-ray diffraction patterns of sono-chemically synthesized Caucasian and Egyptian propolis nanoparticles are presented in Fig. 2. Both samples exhibit distinct diffraction profiles within the 2θ range of 10–80°. The XRD pattern of the Egyptian propolis nanoparticles shows several prominent peaks at 2θ values of 15.2°, 19.8°, 21.6°, and 24.3°. In contrast, the Caucasian propolis nanoparticles display a similar diffraction pattern but with reduced peak intensities. The primary peak occurs at 2θ = 19.7° with an intensity of approximately 2400 counts, while secondary peaks are observed at 21.5° and 24.1° with intensities of 1800 and 1500 counts, respectively. The overall lower peak intensities in the Caucasian sample suggest a lower degree of crystallinity compared to the Egyptian sample [In Supplementary Table 1].

#### UV–visible spectroscopy

**Fig. 3:**
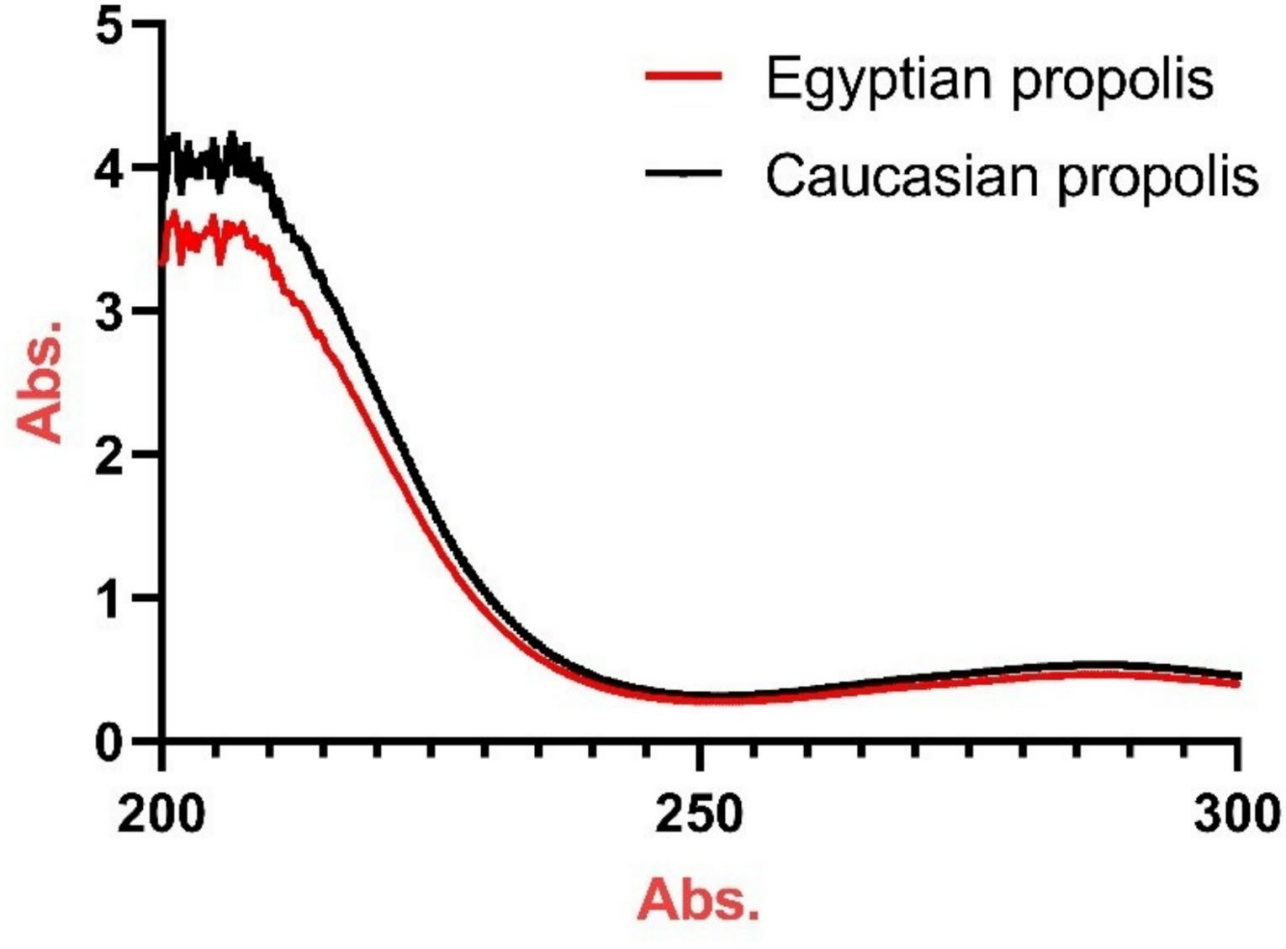
UV Absorbance spectra of of the Egyptian and the Caucasian propolis nanoparticles.

The UV–visible absorption spectra of sono-chemically synthesized Caucasian and Egyptian propolis nanoparticles in the wavelength range of 200–300 nm are presented in Fig. 3. Both samples exhibit strong absorption in the UV region, particularly in the 200–240 nm range, with a gradual decrease in absorption intensity at higher wavelengths.

The Caucasian propolis nanoparticles display a higher overall absorbance compared to the Egyptian counterpart, with maximum absorption values of approximately 4.1 and 3.6 absorbance units, respectively, at around 202–205 nm [In Supplementary Table 2].

The slightly higher absorbance values observed in the Caucasian propolis nanoparticles suggest a higher concentration of aromatic compounds, potentially stronger UV-protective and antioxidant capacity compared to the Egyptian sample.

#### Atomic force microscopy [AFM] analysis

**Fig. 4:**
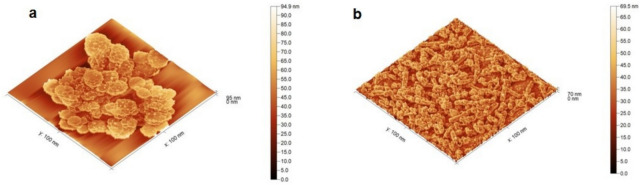
illustrated 3D AFM image of: (**a**) the Egyptian Propolis Nanoparticles; (**b**) the Caucasian Propolis Nanoparticles.

Representative AFM images of the samples are presented in Fig. 4a and b, providing valuable insights into their surface morphology at the nanoscale.

The Caucasian propolis nanoparticles (Fig. 4b) exhibit a densely packed, irregular surface topography characterized by numerous interconnected granular structures with varied heights. The surface demonstrates a relatively uniform distribution of small, closely arranged features with an intricate network of shallow depressions between them. The lateral dimensions of these features range from approximately 10–25 nm, while their heights vary between 20–40 nm.

In contrast, the Egyptian propolis nanoparticles (Fig. 4a) display a distinctly different topographical profile dominated by larger, well-defined spherical and flower-like aggregates. These structures appear more organized and discrete compared to the Caucasian sample, with clear boundaries between individual aggregates. The particle aggregates exhibit diameters ranging from 30–75 nm and heights reaching up to 90 nm. The background surface appears relatively smooth compared to the prominent aggregates, resulting in a higher peak-to-valley difference.

Interestingly, despite the higher roughness values, the Egyptian propolis nanoparticles exhibit a lower surface area [286,072 nm^2^ vs. 723,363 nm^2^] and surface slope [54.39 vs. 101.35] compared to the Caucasian sample. The Caucasian propolis nanoparticles feature a more intricate and densely packed surface structure than the Egyptian propolis.

#### Roughness analysis

Quantitative roughness analysis was performed on both samples to characterize their surface topography more precisely. The roughness parameters were calculated from multiple AFM images to ensure statistical significance. Table 1 summarizes the key roughness parameters for both propolis nanoparticle samples.

**Table 1:** Surface roughness parameters of the Caucasian and the Egyptian propolis nanoparticles derived from AFM analysis.

Parameter	Caucasian propolis	Egyptian propolis
Average height	29.69 ± 0.05 nm	47.38 ± 0.05 nm
RMS roughness (Sq)	8.82 ± 0.01 nm	10.55 ± 0.01 nm
Mean roughness (Sa)	7.14 ± 0.01 nm	8.33 ± 0.01 nm
Maximum peak height (Sp)	39.84 ± 0.05 nm	47.50 ± 0.05 nm
Maximum pit depth (Sv)	29.69 ± 0.05 nm	47.38 ± 0.05 nm
Maximum height (Sz)	69.52 ± 0.05 nm	94.87 ± 0.05 nm
Surface area	723,363 nm^2^	286,072 nm^2^
Surface slope (Sdq)	101.35	54.39
Skewness (Ssk)	0.64	0.23
Kurtosis (Sku)	−0.08	0.08

### Physical properties analysis

#### Dynamic light scattering [DLS] analysis

**Fig. 5:**
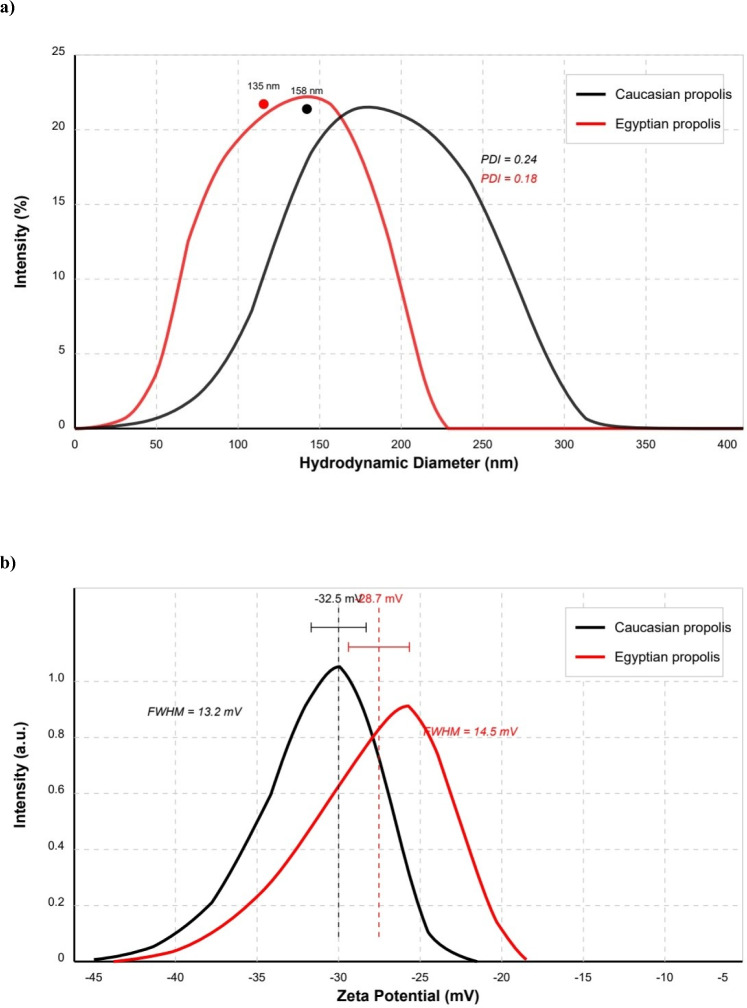

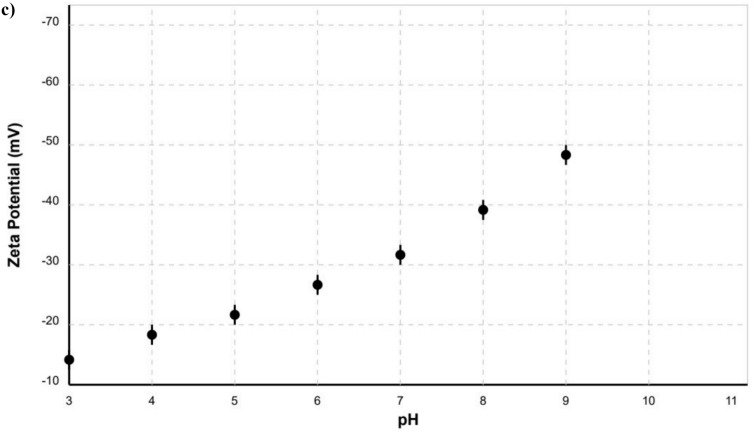
(**a**) illustrated DLS of the Caucasian and the Egyptian propolis Nanoparticles; (**b**) illustrated Zeta potential of the Caucasian and the Egyptian propolis nanoparticles; (**c**) illustrated PH-dependent Zeta potential of the Caucasian and the Egyptian propolis Nanoparticles.

The analysis was conducted at 25 °C with a scattering angle of 173° after appropriate dilution to ensure measurement reliability. The DLS data for both nanoparticle types are summarized in Supplementary Table3 & Fig. 5a, which presents the mean hydrodynamic diameter, polydispersity index [PDI], and zeta potential values in Supplementary Table 3 and 4. The particle size distributions are graphically represented in Fig. 5b, showing the intensity-weighted size distributions for both samples.

#### Zeta potential analysis

The negative values of Zeta potential measurements indicate good electrostatic stabilization in aqueous suspension for both samples in supplementary Table 4. The slightly more negative zeta potential for the Caucasian propolis nanoparticles may be attributed to a higher content of ionizable groups, such as phenolic and carboxylic acid moieties, on the particle surface. This enhanced surface charge may contribute to the better dispersibility of the Caucasian nanoparticles despite their larger size and higher polydispersity.

##### pH-dependent zeta potential behavior

The pH-dependent zeta potential profiles for both nanoparticle types showed that both samples exhibited increasingly negative zeta potentials with increasing pH, following a typical trend for particles with ionizable surface groups Fig. 5c.

The slope of the zeta potential versus pH curve was steeper for the Caucasian propolis nanoparticles compared to the Egyptian sample, suggesting a higher density of titratable groups on the Caucasian nanoparticle surface. At pH 7.4 [physiological pH], the zeta potentials were −38.2 ± 3.1 mV for the Caucasian and − 33.5 ± 2.9 mV for the Egyptian propolis nanoparticles, indicating enhanced electrostatic stabilization under physiological conditions for both samples.

#### BET surface area and pore size analysis

##### BET surface area analysis

The specific surface areas of the propolis nanoparticles were determined using the Brunauer–Emmett–Teller [BET] method in the relative pressure range of 0.05–0.30, where the BET plots showed high linearity [R^2^ > 0.999] for both samples. Table 2 summarizes the BET surface area analysis results.

**Table 2:** BET surface area analysis of the Caucasian and the Egyptian propolis nanoparticles.

Parameter	Caucasian Propolis	Egyptian Propolis
BET surface area (m^2^/g)	142.8 ± 3.2	98.6 ± 2.1
C constant	86.5	72.3
Linear range (P/P_0_)	0.05–0.30	0.05–0.30
Correlation coefficient (R^2^)	0.9998	0.9996
Total pore volume (cm^3^/g)	0.168	0.121
Micropore volume (cm^3^/g)	0.059	0.034
Micropore fraction (%)	35	28
Average pore width (nm)	4.7	4.9

C constant: surface binding energy; related to the energy of adsorption in the first adsorbed layer and consequently, its value is an indication of the magnitude of the adsorbent/adsorbate interactions.

These relatively high C values [typically > 20] indicate strong interactions between nitrogen molecules and the surface of the propolis nanoparticles, suggesting the presence of polar functional groups according to Brunauer et al.^34^; Barrett et al.^35^. The higher C value for the Caucasian sample suggests a more energetically heterogeneous surface, possibly due to a greater density of polar functional groups associated with its unique chemical composition.

##### Pore size distribution analysis

The pore size distributions of the propolis nanoparticles were analyzed using both the Barrett-Joyner-Halenda [BJH] method and the Dubinin-Astakhov [DA] approach [citation]. The BJH method was applied to the desorption branch of the isotherms for mesopore analysis, while the DA method provided information about the micropore characteristics Table 3.

**Table 3:** BJH and DA pore analysis of the Caucasian and the Egyptian propolis nanoparticles.

Parameter	Caucasian propolis	Egyptian propolis
*BJH analysis (desorption)*		
BJH surface area (m^2^/g)	138.5	95.3
BJH pore volume (cm^3^/g)	0.162	0.116
BJH average pore diameter (nm)	4.7	4.9
*Dubinin-Astakhov (DA) analysis*		
DA Limiting MICROPORE VOLUME (cm^3^/g)	0.059	0.034
DA characteristic energy (kJ/mol)	18.7	16.9
DA exponent (n)	2.4	2.2

BJH and DA analysis showed higher results for the Caucasian than the Egyptian nanoparticles indicating a more developed micropore structure in the Caucasian sample. This aligns with the higher initial uptake observed in the adsorption isotherm at low relative pressures.

### Deformed wing virus detection via conventional RT-PCR

**Fig. 6:**
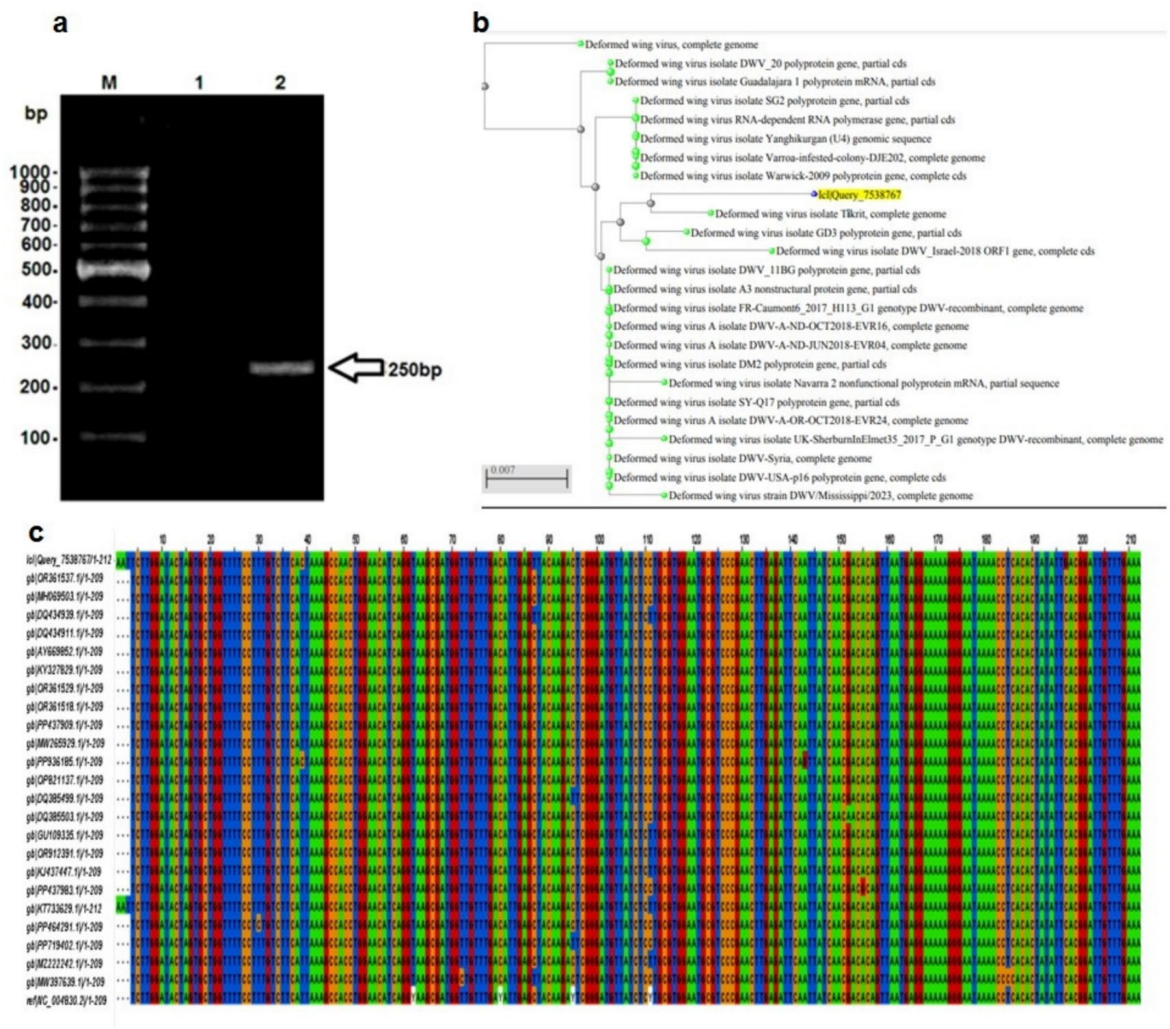
(**a**) Ethidium bromide-stained agarose gel (1.5%) electrophoresis of PCR amplified fragments using specific primers for viruses under investigation from asymptomatic honey bees: (1) DWV; M: Molecular marker (GeneDireX®, TransGen Biotech Co.), N: Negative control. (**b**) Neighbour joining phylogenetic tree of deformed wing virus (DWV) sequence to other sequences on NCBI. (**c**) Multiple Sequences alignment of the Deformed wing virus (which part of the virus) with other sequence.

The PCR product with the expected fragment of 250 bp for deformed wing virus [DWV] was detected from asymptomatic colonies of honey bees’ workers in summer with poor propolis before the cell culture (Fig. 6a). The obtained PCR fragments were recovered sequenced. The sequence of DWV was compared to other sequences on NCBI GenBank https://blast.ncbi.nlm.nih.gov/Blast.cgi Fig. 6b using Neighbour joining phylogenetic tree. Multiple alignment sequence was occurred between DWV and the other sequences using Jalview 2.11.4.1 version and our findings demonstrated that sequences derived from the GenBank database differed at various places in comparison to our query sequence due to substitution and deletion mutations at certain nucleotide sites (Fig. 6c).

### Cell culturing of the honey bee adult heads

**Fig. 7:**
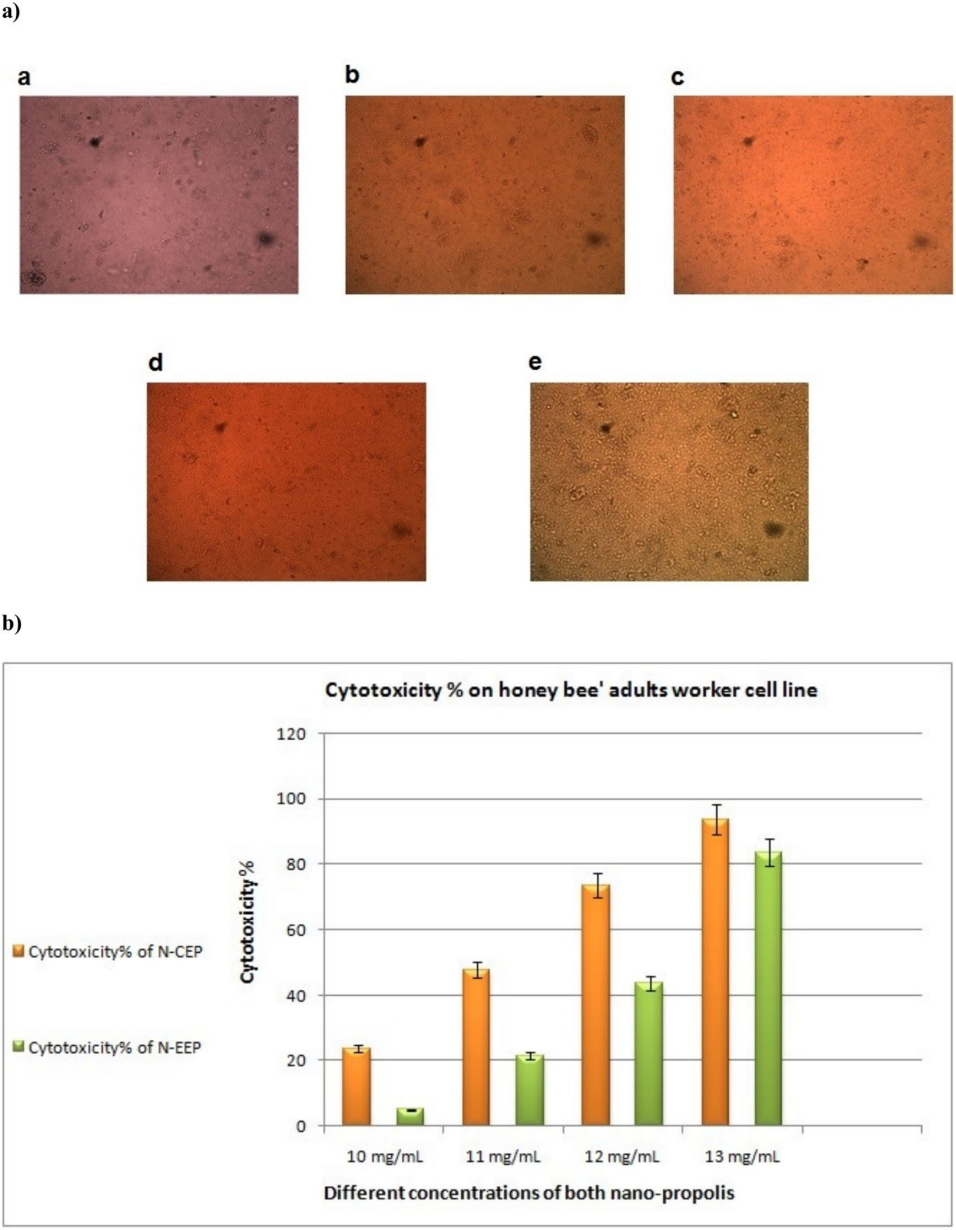
**(a)** The effect of treatments on Adults workers’ head honey bee cell cultures, the cells showed more condensed, clustered and multi layers than untreated cell cultures. (1) Untreated (positive control) suspended cells; (2–5) treated cells; (2) chitosan treatment (5 mg/ml); (3) chitosan nanoparticles treatment (5 mg/ml); (4) Egyptian propolis nanoparticle extract (5 mg/ml) and (5) Caucasian propolis nanoparticle extract(5 mg/ml). (**b**) The Cytotoxicity of the Caucasian and the Egyptian propolis nanoparticles treatments on the honey bee cell cultures; green columns: Nano-Egyptian extract propolis (N-EEP) and orange columns: Nano-Caucasian extract propolis N-CEP).

The infected honey bees’ cell cultures from adults were divided to groups of untreated [positive control], Cucasian or Egyptian propolis nanoparticles-treated, chitosan-treated [5 mg/ml] and chitosan nanoparticles treated [5 mg/ml]. Cell division was faster, appeared better and clear in the treated honey bee cell cultures than in the untreated cells [positive control] Fig. 7a.

#### Cytotoxicity assay

Cytotoxicity and cell viability were assessed using an MTT assay. No cell death was observed in treated cells for either type of cell until concentrations 11 mg/ml for Nano-propolis extract of the Egyptian type and 9 mg/ml for Nano-propolis extract of the Caucasian type. It was shown that the high doses of Nanoparticles from both types of propolis treatments significantly reduced the cell viability of the treated cell lines [*p* ≤ 0.01]. The median inhibitory concentration [IC_50_] values demonstrated that treatment of the cell culture of the honey bee adult workers’ head with Nano-propolis extract had dramatically decreased [*p* ≤ 0.01]. Whereas IC50 values were 12.55 g/L and 11.33 g/L for the Egyptian and the Caucasian types, respectively (Fig. 7b).

### Gene expression

**Fig. 8:**
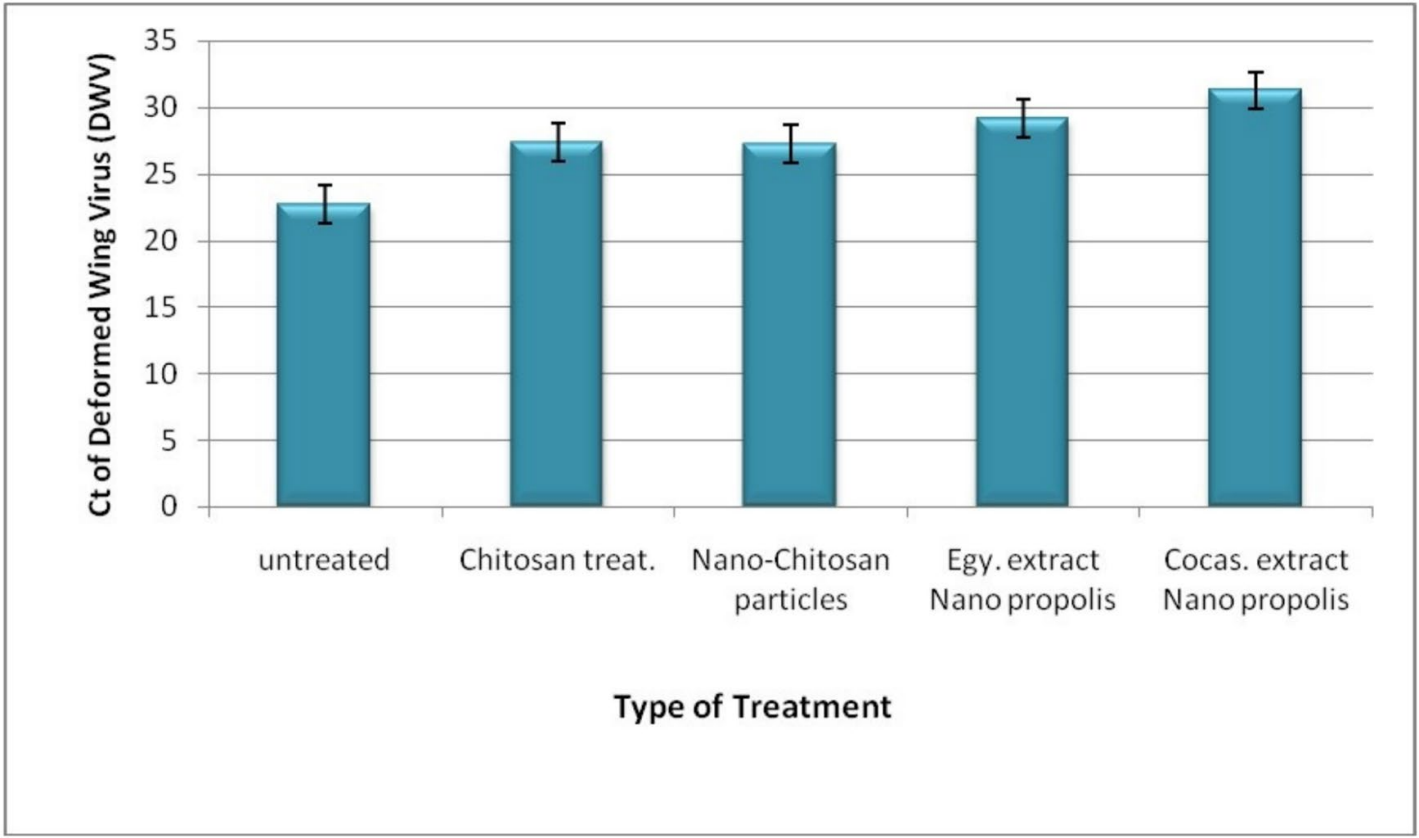
The effect of different treatments on the deformed wing virus (DWV) expression in honey bee.

The studied honey bee’s virus [DWV] was successfully amplified in all treatments. The obtained melting curve showed single peaks confirming the specificity of the amplified products. Expression titers of deformed wing virus [DWV] in untreated and treated honeybee colonies were compared. In general, the virus’ expressions decreased in the treated honeybee colonies than untreated colonies. The propolis nanoparticle extracts inhibited virus expression titer more than Chitosan and chitosan nanoparticles. The Caucasian type of the propolis nanoparticles proved to be the best treatment to decrease DWV titer (Fig. 8).

## Discussion

Deformed wing virus [DWV] is a single-stranded RNA virus infecting honey bees [*Apis mellifera* L.] and it is transmitted by the parasitic mite [*Varroa destructor*]. Although DWV represents a great risk to honey bee performance worldwide, the pathological basis of DWV infection still needs further studies^42^. Studies demonstrated the presence of a link between DWV infection and deformities during development of wings. DWV replication was detected in various body parts of honey bees, such as head, thorax, wings, legs, hemolymph, and gut^43^. In bees with crippled wings, viral replication was detected in all the honey bee body parts^44^.

### Role of natural compounds and propolis in insect protection

Propolis ethanolic extract has been shown to interfere with microorganism growth by inhibiting protein synthesis^45^. The extraction solvent may play a critical role in the isolation of bioactive compounds^46^. Also, propolis extract is characterized by having a variety of flavonoids, isoflavonoids and esters of phenolic acids, which have antibacterial properties against fungi and Gram-positive bacterial strains, and most of them showed antiviral activity according to Kujumgiev et al.^47^, methyl gallate and phthalic acid in the Egyptian propolis have antiviral activity and enhance the cells^6^; Also, propolis extracts defined as antimicrobial, antioxidant, anti-inflamatory and antitumor natural compound^31,46,48^.Chitosan-propolis nanoparticles [capsules] were prepared and characterized in the present study using Ethanolic extract of the Egyptian and the Caucasian propolis. According to the obtained characterization results, the XRD patterns of both propolis nanoparticles revealed a semi-crystalline nature with several distinct diffraction peaks. The observed peaks correspond to various bioactive compounds. The calculated crystallinity indices were 48.2% and 62.7% for the Caucasian and the Egyptian propolis nanoparticles, respectively, confirming the higher crystallinity of the Egyptian sample (Fig. 2; In Supplemental materials, ST1). The XRD patterns obtained for propolis nanoparticles align with previous studies on propolis-derived materials. Salama et al.^49^ reported similar diffraction peaks for propolis extracts, with prominent peaks at 2θ values 21.35 and 21.9. In UV characterization for both propolis nanoparticles the strong absorption in the 200–210 nm region is attributed to π → π* electronic transitions in the aromatic rings, which are abundant in propolis bioactive compounds. The shoulder observed at 225–230 nm is indicative of various phenolic compounds. The minor absorption band centered at approximately 285 nm corresponds to flavonoid compounds, particularly flavones and flavonols, which typically absorb in the 270–295 nm range due to the B-ring of their molecular structure (Fig. 3). The slightly higher absorbance values observed in the Caucasian propolis nanoparticles suggest a higher concentration of aromatic compounds compared to the Egyptian sample (In Supplemental materials, ST2). The spectra of aromatic and flavonoids structures are compatible with Salama et al.^49^ for propolis nanoparticles and Maldonado et al.^50^ for UV characterization of propolis. Atomic force microscopy [AFM] characterization for both types of propolis showed that the propolis is densely packed, irregular surface topography as mentioned by Mezdary and Khirouni^51^, but the Caucasian is more densely than Egyptian propolis. The Roughness analysis showed that the rough values of the Egyptian propolis are higher than the Caucasian, but the Egyptian propolis nanoparticles exhibit a lower surface area [286,072 nm^2^ vs. 723,363 nm^2^] and surface slope [54.39 vs. 101.35] compared to the Caucasian sample, respectively (Fig. 4). This apparent contradiction can be attributed to the different morphological characteristics of the samples. The Caucasian propolis nanoparticles feature a more intricate and densely packed surface structure with numerous small features, resulting in a higher effective surface area and steeper local slopes. In contrast, the Egyptian sample exhibits larger, more discrete aggregates with smoother transitions between features, leading to a lower overall surface area despite the greater height variations. Dynamic light scattering [DLS] measurements were performed for the sonochemically synthesized Caucasian and Egyptian propolis nanoparticles in aqueous suspension (Fig. 5a and Supplemental materials Table 3). The analysis was conducted at 25 °C with a scattering angle of 173° after appropriate dilution to ensure measurement reliability. The Caucasian results referred to were higher than the Egyptian propolis; the hydrodynamic diameters [nm] were 165 ± 12 and 142 ± 8, respectively and Polydispersity Index [PDI] were 0.24 ± 0.02 and 0.18 ± 0.01 respectively; and these results were close to the previous studies^52,53^. Both propolis nanoparticle samples exhibited strongly negative zeta potentials, indicating the predominance of negatively charged functional groups on their surfaces. The Caucasian propolis nanoparticles demonstrated a more negative mean zeta potential [− 32.5 ± 2.8 mV] compared to the Egyptian propolis nanoparticles [−28.7 ± 3.1 mV]. The distribution for the Caucasian sample was narrower, with a full width at half maximum [FWHM] of 13.2 mV, while the Egyptian sample showed a broader distribution with an FWHM of 14.5 mV (Fig. 5b and in supplementary Table 4). Salama et al.^49^ reported that the Zeta value of Propolis nanoparticles was − 27.0 mV for zinc oxide/propolis nano-composites [ZnO-P NCs] and − 34.0 mV for nano-propolis. Also, Abdel-Gawad et al.^54^ assigned the zeta value of propolis was − 28.0 mV, so the previous values referred to propolis as similar to the present results. These findings, in conjunction with the pH-dependent zeta potential profiles and correlations with other physicochemical properties, provide valuable insights for optimizing these nanoparticulate systems for specific biomedical applications. The strong negative surface charge ensures good colloidal stability according to Tao et al.,^55^. Brunauer–Emmett–Teller [BET] method showed that the Caucasian propolis nanoparticles exhibited a significantly higher BET surface area [142.8 ± 3.2 m^2^/g] compared to the Egyptian propolis nanoparticles [98.6 ± 2.1 m^2^/g]. This difference of approximately 45.0% in surface area can be attributed to the distinct morphological features observed in AFM analysis, where the Caucasian sample showed a more densely packed, fine-grained structure with higher surface area compared to the larger, discrete aggregates of the Egyptian sample (Table 2). As shown before, Brunauer–Emmett–Teller [BET] method indicated a high specific surface area, which is related to the large active surface area^56,57^. The Caucasian propolis nanoparticles exhibited a bimodal pore size distribution with maxima at approximately 2.0 nm and 4.7 nm, suggesting the presence of small and larger mesopores. In contrast, the Egyptian propolis nanoparticles showed a more unimodal distribution centered at 4.9 nm, with a smaller contribution from micropores. Dubinin-Astakhov [DA] analysis was applied in experimenta; isotherm in order to determine adsorption energy and heterogeneity parameter^58,59^. The Caucasian propolis nanoparticles were higher than the Egyptian propolis nanoparticles in Dubinin-Astakhov [DA] analysis characteristic energy [18.7 kJ/mol vs. 16.9 kJ/mol], respectively, suggesting stronger adsorption energetics in the micropores and in DA exponent [n] [2.4 vs. 2.2], which indicates a somewhat more heterogeneous micropore system (Table 3). The colloidal properties determined through these analyses provide valuable insights for optimizing these nanoparticles for specific biomedical or any biological applications, particularly those requiring consistent size distributions and stable colloidal behavior in physiological environments. The different values in these results between Caucasian and Egyptian propolis nanoparticles may be because of their different origin, sources, and geographical locations. However, the results of characterization showed that the Caucasian propolis nanoparticles have more functional groups and are more absorbent than the Egyptian type.

In general, propolis with/and chitosan proved to be effective in improving honey bee cells cultured from the heads of adult worker bees, as they divided cells faster and appeared purer than untreated colonies that were used as a positive control. As introduced before by Abd El-Samie et al.^6^ that methyl gallate in propolis, used as an antiviral compound, may have helped reduce the virus loads in the honey bee cell cultures used. Beside flavonoids, propolis contains esters of phenolic acids, which have antibacterial properties against fungi and Gram-positive bacterial strains, and most of them showed antiviral activity according to Kujumgiev et al.^47^ and Naree et al.^60^. Chitosan used as an enhancer to the honey bee health against the parasitic protozoan [*Nosema ceranae*] infection which reduced the spores of Nosema^60,61^. Additionally, chitosan nanoparticles are suitable as potential materials to boost colony strength during early spring^62^. Also, as reported by El Gamal et al.^63^, chitosan-based nanomaterials proved a superior effect against plant viruses. So, Chitosan feasibly can be involved in viral disease management strategies under field conditions without serious health concerns and environmental costs^63^.

The present study proved the antiviral role of the chitosan and its nanocomposites of propolis against deformed wing virus. The viral load decreased in vitro upon application of chitosan and chitosan nanoparticles (Fig. 7a), through induction of cell enhancement thus it can be recommended as a supplement or treat against the virus in the bee hives. The extract of the Caucasian propolis nanoparticles showed slightly higher effect than the Egyptian extract nanoparticles against deformed wing virus [DWV] titer by using qRT-PCR at Ct 31.24 and 29.16, respectively (Fig. 8). The cytotoxicity analysis of both treatments of nano-propolis types revealed a similar effect. The cell mortality started at a concenteration of 9 mg/ml for the Caucasian type and 10 mg/ml for the Egyptian type; The IC50 values were 12.55 g/L and 11.33 g/L for the Egyptian and the Caucasian types, respectively (Fig. 7b).

The previous studies showed the importance of using propolis as a treatment or supplement against viral diseases of honey bees for decreasing the titer of different types of viruses as deformed wing virus, black queen cell virus, varroa destructor virus-1 and kakugo virus^6^. Our findings are consistent with those of Drescher et al.^64^, who found that propolis treatment reduced deformed wing virus [DWV] titers in honey bee colonies. On the other hand, Borba et al.^65^, found no difference in DWV, black queen cell virus [BQCV], or Israeli acute bee paralysis virus [IAPV] or viral loads in fall and spring bees from propolis-rich or propolis-poor colonies. Additionally, propolis can be a detoxifying agent or a primer for detoxification pathways, as well as enhancing bee lifespan through antioxidant-related pathways according to Simone-Finstrom et al.^18^ and increasing honey bee immune status^66,67^. Interestingly, propolis effects extend as an antimicrobial, antioxidant, anti-inflammatory and antitumor compound^46^. Also, the mixture of chitosan and propolis nanoparticles investigated antiviral activities against other viral diseases related to human and animals^68,69^.

Although Nano-Caucasian propolis showed slightly better results than Nano-Egyptian propolis in vitro, in general we highlight the importance of propolis in its various types as a treatment or nutritional supplement and its use in various industries, as it can be used in smaller quantities and its transformation into nanoform gives better and cheaper results that benefit workers in the honey bee industry and other industries. We recommend that future studies shed light on the use of natural products in general and honeybee products in particular as antivirals, as bee viruses have spread rapidly and have different and rapid mutations, and they kill bees suddenly, along with other pathogens. This represents very large losses for those working in the beekeeping field. With this research, we are trying to produce a product that is easy to trade, cheap, and also effective for the most delicate diseases, such as viruses. Recommended applications of the propolis/chitoasn nanoparticles may enhance and raise the level of activity of honey bee hives by inhibiting the deformed wing virus in infected honey bees.

## Supplementary Information

Supplementary Information 1.

Supplementary Information 2.

Supplementary Information 3.

Supplementary Information 4.

Supplementary Information 5.

Supplementary Information 6.

## Data Availability

The datasets used and/or analysed during the current study available from the corresponding author on reasonable request.

## Abbreviations

DWV: Deformed wing virus
RT-qPCR: Quantitative real time-polymerase chain reaction
FTIR: Fourier transform infrared spectroscopy
GC–MS: Gas chromatography-mass spectrometry
HPLC: High-performance liquid chromatography
XRD: X-ray diffraction
DLS: Dynamic light scattering
AFM: Atomic force microscopy
TEM: Transmission electron microscope
SEM: Scanning electron microscope
BET: Brunauer–Emmett–Teller
mg/mL: Milligram/milliliter
g: Gram
STPP: Sodium tripolyphosphate
μm: Micrometer
rpm: Round per minute
W/cm^2^: Watt per square centimeter
UV: Visible spectroscopy
kHz: Kilohertz
BJH: Barrett-Joyner-Halenda
TRNA: Total RNA
cDNA: Complementary DNA
EP: Ethanolic propolis
MTT: 3-[4,5-dimethylthiazol-2-yl]-2,5-diphenyltetrazolium bromide
DMSO: Dimethyl sulfoxide
ELISA: Enzyme linked immunosorbent assay
OD: Optical density
nm: Nanometer
PDI: Polydispersity index
DA: Dubinin-Astakhov
ZnO-P NCs: Zinc oxide/propolis nano-composites
mV: Millivolt
DA: Dubinin-Astakhov
Ct: Thermal cycle
BQCV: Black queen cell virus
IAPV: Israeli acute bee paralysis virus
