# Supplementary material for: Caucasian and Egyptian chitosan/propolis nanocomposites inhibit deformed wing virus in Apis mellifera L. cell lines
Source: Sci Rep. 2026 Jun 4;16:17333. doi: 10.1038/s41598-026-54534-9 (PMC13237102; doi:10.1038/s41598-026-54534-9)
Supplement: Supplementary file 5 — Supplementary Information 5. [file 41598_2026_54534_MOESM5_ESM.doc]

Caucasian and Egyptian chitosan/propolis nanocomposites inhibit Deformed Wing Virus in *Apis mellifera* L. cell lines

**Heba Seyam^1,2*^, Sameh H. Ismail^2^, Heba M. Hamama^2,3^**

**1- *Honey Bee Research Department, Plant Protection Research Institute, Agricultural Research Center, Giza, Egypt, *, ORCID:0009-0006-4866-6380**

**2- Faculty of Postgraduate studies for Nanotechnology, Cairo University, El-Sheikh Zayed Branch Campus, PO Box 12588, Giza, Egypt. drsameheltayer@yahoo.com**

**3-Entomology Department, Faculty of Science, Cairo University, Giza, Egypt, 12613,**

***E.mail1:** [**heba.seyam@yahoo.com**](mailto:heba.seyam@yahoo.com) **(Correspond author)**

**E.mail2:** [**drsameheltayer@yahoo.com**](mailto:drsameheltayer@yahoo.com)

**E.mail3:** [**hebahmama@gmail.com**](mailto:hebahmama@gmail.com)**,** [**hahmed@sci.cu.edu.eg**](mailto:hahmed@sci.cu.edu.eg)

**Supplementary Materials**


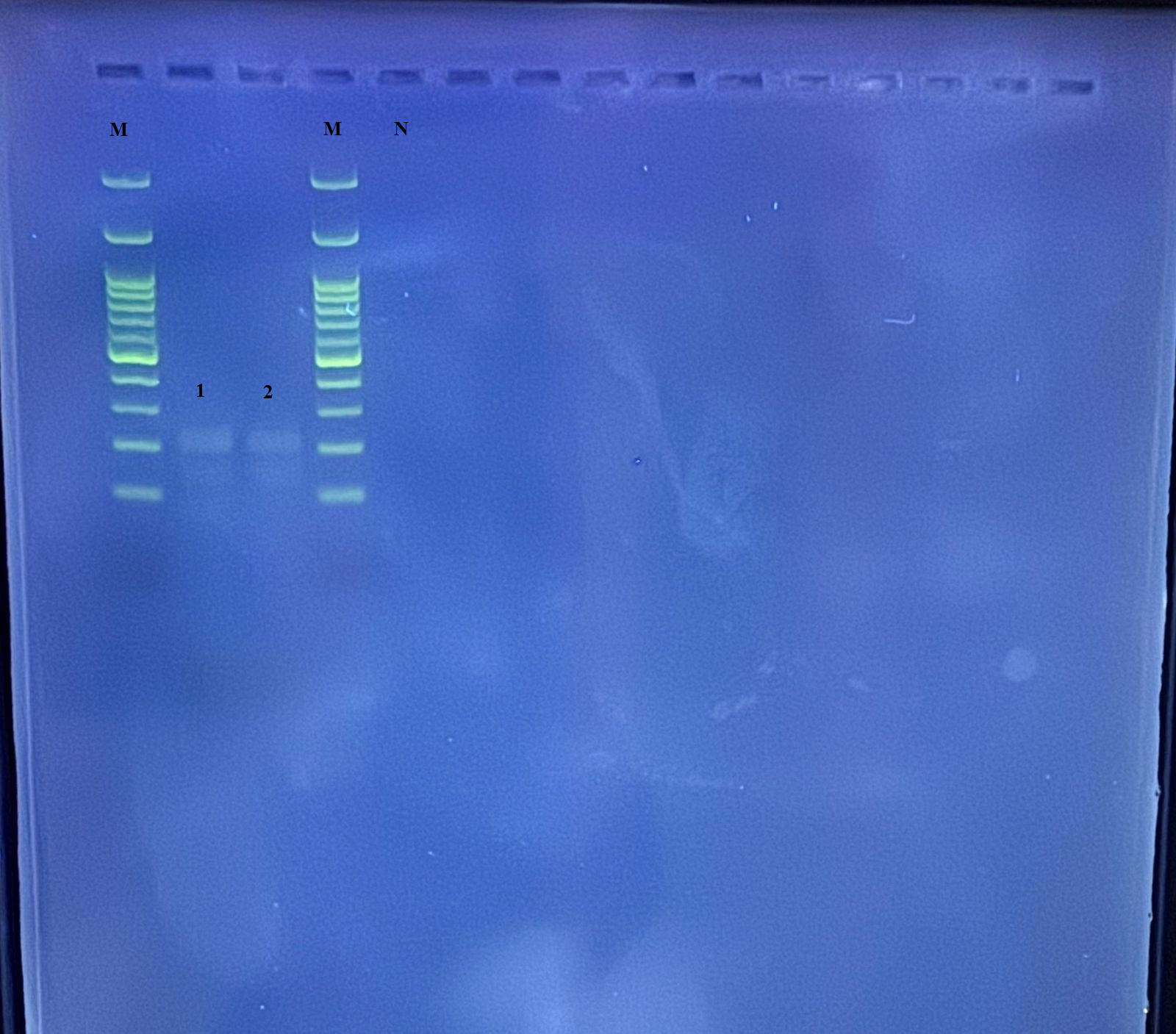


Fig. S1 Ethidium bromide-stained agarose gel (1.5%) electrophoresis of PCR amplified fragments using specific primers for viruses under investigation from asymptomatic honey bees: lanes (1&2) DWV; M lane: Molecular marker (GeneDireX®, TransGen Biotech Co.); N lane: Negative control.

**List of Tables**

**Table 1:** Major XRD Peak pPositions and Relative Intensities of the Caucasian and the Egyptian Propolis Nanoparticles

| 2θ (degrees) | Propolis Nanoparticles  Relative Intensity (%) -  - | | Possible Assignment |
| --- | --- | --- | --- |
|  | Caucasian | Egyptian |  |
| 13.7 ± 0.2 | 15 | 25 | Flavonoid compounds |
| 15.2 ± 0.2 | 25 | 35 | Wax esters |
| 17.5 ± 0.2 | 30 | 40 | Polyphenols |
| 19.8 ± 0.2 | 100 | 100 | Flavonoids/phenolic acids |
| 21.6 ± 0.2 | 75 | 70 | Terpenoids |
| 24.3 ± 0.2 | 60 | 60 | Aromatic compounds |
| 29.1 ± 0.2 | 10 | 15 | Wax components |

**Table 2:** UV-Visible spectral characteristics of the Caucasian and the Egyptian propolis nanoparticles.

| Spectral Feature | Wavelength (nm) | Probolis Nanoparticles Absorbance (a.u.) – | | Assignment |
| --- | --- | --- | --- | --- |
|  |  | Caucasian | Egyptian |  |
| Maximum absorption | 202-205 | 4.1 ± 0.1 | 3.6 ± 0.1 | π→π* transitions in aromatic rings |
| Shoulder | 225-230 | 3.2 ± 0.1 | 2.8 ± 0.1 | Phenolic compounds |
| Minor band | 285-290 | 0.5 ± 0.05 | 0.4 ± 0.05 | Flavonoids and phenolic acids |
| Minimum absorption | 250-260 | 0.3 ± 0.05 | 0.3 ± 0.05 | Background absorption |

**Table 3**: Dynamic light scattering parameters for the Caucasian and the Egyptian propolis nanoparticles.

| Parameter | Caucasian Propolis | Egyptian Propolis |
| --- | --- | --- |
| Mean Hydrodynamic Diameter (nm) | 165 ± 12 | 142 ± 8 |
| Z-Average (nm) | 172 ± 10 | 148 ± 7 |
| Polydispersity Index (PDI) | 0.24 ± 0.02 | 0.18 ± 0.01 |
| Size Distribution Range (nm) | 90-280 | 85-220 |
| Mode (nm) | 158 | 135 |
| Zeta Potential (mV) | -32.5 ± 2.8 | -28.7 ± 3.1 |
| Derived Count Rate (kcps) | 12,564 ± 843 | 14,218 ± 762 |
| pH of Suspension | 6.8 ± 0.1 | 6.7 ± 0.1 |

**Table 4: Zeta potential and electrokinetic parameters of the Caucasian and the Egyptian propolis nanoparticles.**

| Parameter | Caucasian Propolis | Egyptian Propolis |
| --- | --- | --- |
| Zeta Potential (mV) | -32.5 ± 2.8 | -28.7 ± 3.1 |
| Electrophoretic Mobility (μmcm/Vs) | -2.54 ± 0.15 | -2.23 ± 0.18 |
| Conductivity (mS/cm) | 0.42 ± 0.03 | 0.38 ± 0.02 |
| pH | 6.8 ± 0.1 | 6.7 ± 0.1 |
| FWHM* of Zeta Distribution (mV) | 13.2 | 14.5 |
| Distribution Skewness | -0.17 | -0.08 |

*FWHM: Full Width at Half Maximum.
