# Supplementary material for: Caucasian and Egyptian chitosan/propolis nanocomposites inhibit deformed wing virus in Apis mellifera L. cell lines
Source: Sci Rep. 2026 Jun 4;16:17333. doi: 10.1038/s41598-026-54534-9 (PMC13237102; doi:10.1038/s41598-026-54534-9)
Supplement: Supplementary file 6 — Supplementary Information 6. [file 41598_2026_54534_MOESM6_ESM.rtf]

<?xml version="1.0" encoding="UTF-8"?>
<GraphPadPrismFile xmlns="http://graphpad.com/prism/Prism.htm" PrismXMLVersion="5.00">
<Created>
<OriginalVersion CreatedByProgram="GraphPad Prism" CreatedByVersion="8.4.3.686" Login="Sameh" DateTime="2025-02-21T17:57:20+02:00"/>
</Created>
<InfoSequence>
<Ref ID="Info0" Selected="1"/>
</InfoSequence>
<Info ID="Info0">
<Title>Project info 1</Title>
<Notes/>
<Constant><Name>Experiment Date</Name><Value>2022-11-18</Value></Constant>
<Constant><Name>Experiment ID</Name><Value/></Constant>
<Constant><Name>Notebook ID</Name><Value/></Constant>
<Constant><Name>Project</Name><Value/></Constant>
<Constant><Name>Experimenter</Name><Value/></Constant>
<Constant><Name>Protocol</Name><Value/></Constant>
</Info>

<TableSequence>

<Ref ID="Table0" Selected="1"/>
</TableSequence>
<Table ID="Table0" XFormat="numbers" YFormat="replicates" Replicates="3" TableType="XY" EVFormat="AsteriskAfterNumber">
<Title>Data 1</Title>
<XColumn Width="114" Subcolumns="1" Decimals="6">
<Title>2 theta</Title>
<Subcolumn>
<d/>
<d>7.2</d>
<d>7.25021</d>
<d>7.30042</d>
<d>7.35063</d>
<d>7.400841</d>
<d>7.451051</d>
<d>7.501261</d>
<d>7.551471</d>
<d>7.601681</d>
<d>7.651892</d>
<d>7.702102</d>
<d>7.752312</d>
<d>7.802522</d>
<d>7.852732</d>
<d>7.902943</d>
<d>7.953153</d>
<d>8.003363</d>
<d>8.053573</d>
<d>8.103783</d>
<d>8.153993</d>
<d>8.204204</d>
<d>8.254414</d>
<d>8.304624</d>
<d>8.354834</d>
<d>8.405044</d>
<d>8.455255</d>
<d>8.505465</d>
<d>8.555675</d>
<d>8.605885</d>
<d>8.656095</d>
<d>8.706306</d>
<d>8.756516</d>
<d>8.806726</d>
<d>8.856936</d>
<d>8.907146</d>
<d>8.957357</d>
<d>9.007567</d>
<d>9.057777</d>
<d>9.107987</d>
<d>9.158197</d>
<d>9.208408</d>
<d>9.258618</d>
<d>9.308828</d>
<d>9.359038</d>
<d>9.409248</d>
<d>9.459458</d>
<d>9.509669</d>
<d>9.559879</d>
<d>9.610089</d>
<d>9.660299</d>
<d>9.710509</d>
<d>9.76072</d>
<d>9.81093</d>
<d>9.86114</d>
<d>9.91135</d>
<d>9.96156</d>
<d>10.01177</d>
<d>10.06198</d>
<d>10.11219</d>
<d>10.1624</d>
<d>10.21261</d>
<d>10.26282</d>
<d>10.31303</d>
<d>10.36324</d>
<d>10.41345</d>
<d>10.46366</d>
<d>10.51387</d>
<d>10.56408</d>
<d>10.61429</d>
<d>10.6645</d>
<d>10.71471</d>
<d>10.76492</d>
<d>10.81513</d>
<d>10.86534</d>
<d>10.91555</d>
<d>10.96576</d>
<d>11.01597</d>
<d>11.06618</d>
<d>11.11639</d>
<d>11.16661</d>
<d>11.21682</d>
<d>11.26703</d>
<d>11.31724</d>
<d>11.36745</d>
<d>11.41766</d>
<d>11.46787</d>
<d>11.51808</d>
<d>11.56829</d>
<d>11.6185</d>
<d>11.66871</d>
<d>11.71892</d>
<d>11.76913</d>
<d>11.81934</d>
<d>11.86955</d>
<d>11.91976</d>
<d>11.96997</d>
<d>12.02018</d>
<d>12.07039</d>
<d>12.1206</d>
<d>12.17081</d>
<d>12.22102</d>
<d>12.27123</d>
<d>12.32144</d>
<d>12.37165</d>
<d>12.42186</d>
<d>12.47207</d>
<d>12.52228</d>
<d>12.57249</d>
<d>12.6227</d>
<d>12.67291</d>
<d>12.72312</d>
<d>12.77333</d>
<d>12.82354</d>
<d>12.87375</d>
<d>12.92396</d>
<d>12.97417</d>
<d>13.02438</d>
<d>13.07459</d>
<d>13.1248</d>
<d>13.17501</d>
<d>13.22522</d>
<d>13.27543</d>
<d>13.32564</d>
<d>13.37585</d>
<d>13.42606</d>
<d>13.47627</d>
<d>13.52648</d>
<d>13.57669</d>
<d>13.6269</d>
<d>13.67711</d>
<d>13.72732</d>
<d>13.77754</d>
<d>13.82775</d>
<d>13.87796</d>
<d>13.92817</d>
<d>13.97838</d>
<d>14.02859</d>
<d>14.0788</d>
<d>14.12901</d>
<d>14.17922</d>
<d>14.22943</d>
<d>14.27964</d>
<d>14.32985</d>
<d>14.38006</d>
<d>14.43027</d>
<d>14.48048</d>
<d>14.53069</d>
<d>14.5809</d>
<d>14.63111</d>
<d>14.68132</d>
<d>14.73153</d>
<d>14.78174</d>
<d>14.83195</d>
<d>14.88216</d>
<d>14.93237</d>
<d>14.98258</d>
<d>15.03279</d>
<d>15.083</d>
<d>15.13321</d>
<d>15.18342</d>
<d>15.23363</d>
<d>15.28384</d>
<d>15.33405</d>
<d>15.38426</d>
<d>15.43447</d>
<d>15.48468</d>
<d>15.53489</d>
<d>15.5851</d>
<d>15.63531</d>
<d>15.68552</d>
<d>15.73573</d>
<d>15.78594</d>
<d>15.83615</d>
<d>15.88636</d>
<d>15.93657</d>
<d>15.98678</d>
<d>16.03699</d>
<d>16.0872</d>
<d>16.13741</d>
<d>16.18762</d>
<d>16.23783</d>
<d>16.28804</d>
<d>16.33825</d>
<d>16.38847</d>
<d>16.43868</d>
<d>16.48889</d>
<d>16.5391</d>
<d>16.58931</d>
<d>16.63952</d>
<d>16.68973</d>
<d>16.73994</d>
<d>16.79015</d>
<d>16.84036</d>
<d>16.89057</d>
<d>16.94078</d>
<d>16.99099</d>
<d>17.0412</d>
<d>17.09141</d>
<d>17.14162</d>
<d>17.19183</d>
<d>17.24204</d>
<d>17.29225</d>
<d>17.34246</d>
<d>17.39267</d>
<d>17.44288</d>
<d>17.49309</d>
<d>17.5433</d>
<d>17.59351</d>
<d>17.64372</d>
<d>17.69393</d>
<d>17.74414</d>
<d>17.79435</d>
<d>17.84456</d>
<d>17.89477</d>
<d>17.94498</d>
<d>17.99519</d>
<d>18.0454</d>
<d>18.09561</d>
<d>18.14582</d>
<d>18.19603</d>
<d>18.24624</d>
<d>18.29645</d>
<d>18.34666</d>
<d>18.39687</d>
<d>18.44708</d>
<d>18.49729</d>
<d>18.5475</d>
<d>18.59771</d>
<d>18.64792</d>
<d>18.69813</d>
<d>18.74834</d>
<d>18.79855</d>
<d>18.84876</d>
<d>18.89897</d>
<d>18.94918</d>
<d>18.9994</d>
<d>19.04961</d>
<d>19.09982</d>
<d>19.15003</d>
<d>19.20024</d>
<d>19.25045</d>
<d>19.30066</d>
<d>19.35087</d>
<d>19.40108</d>
<d>19.45129</d>
<d>19.5015</d>
<d>19.55171</d>
<d>19.60192</d>
<d>19.65213</d>
<d>19.70234</d>
<d>19.75255</d>
<d>19.80276</d>
<d>19.85297</d>
<d>19.90318</d>
<d>19.95339</d>
<d>20.0036</d>
<d>20.05381</d>
<d>20.10402</d>
<d>20.15423</d>
<d>20.20444</d>
<d>20.25465</d>
<d>20.30486</d>
<d>20.35507</d>
<d>20.40528</d>
<d>20.45549</d>
<d>20.5057</d>
<d>20.55591</d>
<d>20.60612</d>
<d>20.65633</d>
<d>20.70654</d>
<d>20.75675</d>
<d>20.80696</d>
<d>20.85717</d>
<d>20.90738</d>
<d>20.95759</d>
<d>21.0078</d>
<d>21.05801</d>
<d>21.10822</d>
<d>21.15843</d>
<d>21.20864</d>
<d>21.25885</d>
<d>21.30906</d>
<d>21.35927</d>
<d>21.40948</d>
<d>21.45969</d>
<d>21.5099</d>
<d>21.56011</d>
<d>21.61033</d>
<d>21.66054</d>
<d>21.71075</d>
<d>21.76096</d>
<d>21.81117</d>
<d>21.86138</d>
<d>21.91159</d>
<d>21.9618</d>
<d>22.01201</d>
<d>22.06222</d>
<d>22.11243</d>
<d>22.16264</d>
<d>22.21285</d>
<d>22.26306</d>
<d>22.31327</d>
<d>22.36348</d>
<d>22.41369</d>
<d>22.4639</d>
<d>22.51411</d>
<d>22.56432</d>
<d>22.61453</d>
<d>22.66474</d>
<d>22.71495</d>
<d>22.76516</d>
<d>22.81537</d>
<d>22.86558</d>
<d>22.91579</d>
<d>22.966</d>
<d>23.01621</d>
<d>23.06642</d>
<d>23.11663</d>
<d>23.16684</d>
<d>23.21705</d>
<d>23.26726</d>
<d>23.31747</d>
<d>23.36768</d>
<d>23.41789</d>
<d>23.4681</d>
<d>23.51831</d>
<d>23.56852</d>
<d>23.61873</d>
<d>23.66894</d>
<d>23.71915</d>
<d>23.76936</d>
<d>23.81957</d>
<d>23.86978</d>
<d>23.91999</d>
<d>23.9702</d>
<d>24.02041</d>
<d>24.07062</d>
<d>24.12083</d>
<d>24.17104</d>
<d>24.22126</d>
<d>24.27147</d>
<d>24.32168</d>
<d>24.37189</d>
<d>24.4221</d>
<d>24.47231</d>
<d>24.52252</d>
<d>24.57273</d>
<d>24.62294</d>
<d>24.67315</d>
<d>24.72336</d>
<d>24.77357</d>
<d>24.82378</d>
<d>24.87399</d>
<d>24.9242</d>
<d>24.97441</d>
<d>25.02462</d>
<d>25.07483</d>
<d>25.12504</d>
<d>25.17525</d>
<d>25.22546</d>
<d>25.27567</d>
<d>25.32588</d>
<d>25.37609</d>
<d>25.4263</d>
<d>25.47651</d>
<d>25.52672</d>
<d>25.57693</d>
<d>25.62714</d>
<d>25.67735</d>
<d>25.72756</d>
<d>25.77777</d>
<d>25.82798</d>
<d>25.87819</d>
<d>25.9284</d>
<d>25.97861</d>
<d>26.02882</d>
<d>26.07903</d>
<d>26.12924</d>
<d>26.17945</d>
<d>26.22966</d>
<d>26.27987</d>
<d>26.33008</d>
<d>26.38029</d>
<d>26.4305</d>
<d>26.48071</d>
<d>26.53092</d>
<d>26.58113</d>
<d>26.63134</d>
<d>26.68155</d>
<d>26.73176</d>
<d>26.78198</d>
<d>26.83219</d>
<d>26.8824</d>
<d>26.93261</d>
<d>26.98282</d>
<d>27.03303</d>
<d>27.08324</d>
<d>27.13345</d>
<d>27.18366</d>
<d>27.23387</d>
<d>27.28408</d>
<d>27.33429</d>
<d>27.3845</d>
<d>27.43471</d>
<d>27.48492</d>
<d>27.53513</d>
<d>27.58534</d>
<d>27.63555</d>
<d>27.68576</d>
<d>27.73597</d>
<d>27.78618</d>
<d>27.83639</d>
<d>27.8866</d>
<d>27.93681</d>
<d>27.98702</d>
<d>28.03723</d>
<d>28.08744</d>
<d>28.13765</d>
<d>28.18786</d>
<d>28.23807</d>
<d>28.28828</d>
<d>28.33849</d>
<d>28.3887</d>
<d>28.43891</d>
<d>28.48912</d>
<d>28.53933</d>
<d>28.58954</d>
<d>28.63975</d>
<d>28.68996</d>
<d>28.74017</d>
<d>28.79038</d>
<d>28.84059</d>
<d>28.8908</d>
<d>28.94101</d>
<d>28.99122</d>
<d>29.04143</d>
<d>29.09164</d>
<d>29.14185</d>
<d>29.19206</d>
<d>29.24227</d>
<d>29.29248</d>
<d>29.34269</d>
<d>29.39291</d>
<d>29.44312</d>
<d>29.49333</d>
<d>29.54354</d>
<d>29.59375</d>
<d>29.64396</d>
<d>29.69417</d>
<d>29.74438</d>
<d>29.79459</d>
<d>29.8448</d>
<d>29.89501</d>
<d>29.94522</d>
<d>29.99543</d>
<d>30.04564</d>
<d>30.09585</d>
<d>30.14606</d>
<d>30.19627</d>
<d>30.24648</d>
<d>30.29669</d>
<d>30.3469</d>
<d>30.39711</d>
<d>30.44732</d>
<d>30.49753</d>
<d>30.54774</d>
<d>30.59795</d>
<d>30.64816</d>
<d>30.69837</d>
<d>30.74858</d>
<d>30.79879</d>
<d>30.849</d>
<d>30.89921</d>
<d>30.94942</d>
<d>30.99963</d>
<d>31.04984</d>
<d>31.10005</d>
<d>31.15026</d>
<d>31.20047</d>
<d>31.25068</d>
<d>31.30089</d>
<d>31.3511</d>
<d>31.40131</d>
<d>31.45152</d>
<d>31.50173</d>
<d>31.55194</d>
<d>31.60215</d>
<d>31.65236</d>
<d>31.70257</d>
<d>31.75278</d>
<d>31.80299</d>
<d>31.8532</d>
<d>31.90341</d>
<d>31.95362</d>
<d>32.00384</d>
<d>32.05405</d>
<d>32.10426</d>
<d>32.15447</d>
<d>32.20468</d>
<d>32.25489</d>
<d>32.3051</d>
<d>32.35531</d>
<d>32.40552</d>
<d>32.45573</d>
<d>32.50594</d>
<d>32.55615</d>
<d>32.60636</d>
<d>32.65657</d>
<d>32.70678</d>
<d>32.75699</d>
<d>32.8072</d>
<d>32.85741</d>
<d>32.90762</d>
<d>32.95783</d>
<d>33.00804</d>
<d>33.05825</d>
<d>33.10846</d>
<d>33.15867</d>
<d>33.20888</d>
<d>33.25909</d>
<d>33.3093</d>
<d>33.35951</d>
<d>33.40972</d>
<d>33.45993</d>
<d>33.51014</d>
<d>33.56035</d>
<d>33.61056</d>
<d>33.66077</d>
<d>33.71098</d>
<d>33.76119</d>
<d>33.8114</d>
<d>33.86161</d>
<d>33.91182</d>
<d>33.96203</d>
<d>34.01224</d>
<d>34.06245</d>
<d>34.11266</d>
<d>34.16287</d>
<d>34.21308</d>
<d>34.26329</d>
<d>34.3135</d>
<d>34.36371</d>
<d>34.41392</d>
<d>34.46413</d>
<d>34.51434</d>
<d>34.56455</d>
<d>34.61477</d>
<d>34.66498</d>
<d>34.71519</d>
<d>34.7654</d>
<d>34.81561</d>
<d>34.86582</d>
<d>34.91603</d>
<d>34.96624</d>
<d>35.01645</d>
<d>35.06666</d>
<d>35.11687</d>
<d>35.16708</d>
<d>35.21729</d>
<d>35.2675</d>
<d>35.31771</d>
<d>35.36792</d>
<d>35.41813</d>
<d>35.46834</d>
<d>35.51855</d>
<d>35.56876</d>
<d>35.61897</d>
<d>35.66918</d>
<d>35.71939</d>
<d>35.7696</d>
<d>35.81981</d>
<d>35.87002</d>
<d>35.92023</d>
<d>35.97044</d>
<d>36.02065</d>
<d>36.07086</d>
<d>36.12107</d>
<d>36.17128</d>
<d>36.22149</d>
<d>36.2717</d>
<d>36.32191</d>
<d>36.37212</d>
<d>36.42233</d>
<d>36.47254</d>
<d>36.52275</d>
<d>36.57296</d>
<d>36.62317</d>
<d>36.67338</d>
<d>36.72359</d>
<d>36.7738</d>
<d>36.82401</d>
<d>36.87422</d>
<d>36.92443</d>
<d>36.97464</d>
<d>37.02485</d>
<d>37.07506</d>
<d>37.12527</d>
<d>37.17548</d>
<d>37.2257</d>
<d>37.27591</d>
<d>37.32612</d>
<d>37.37633</d>
<d>37.42654</d>
<d>37.47675</d>
<d>37.52696</d>
<d>37.57717</d>
<d>37.62738</d>
<d>37.67759</d>
<d>37.7278</d>
<d>37.77801</d>
<d>37.82822</d>
<d>37.87843</d>
<d>37.92864</d>
<d>37.97885</d>
<d>38.02906</d>
<d>38.07927</d>
<d>38.12948</d>
<d>38.17969</d>
<d>38.2299</d>
<d>38.28011</d>
<d>38.33032</d>
<d>38.38053</d>
<d>38.43074</d>
<d>38.48095</d>
<d>38.53116</d>
<d>38.58137</d>
<d>38.63158</d>
<d>38.68179</d>
<d>38.732</d>
<d>38.78221</d>
<d>38.83242</d>
<d>38.88263</d>
<d>38.93284</d>
<d>38.98305</d>
<d>39.03326</d>
<d>39.08347</d>
<d>39.13368</d>
<d>39.18389</d>
<d>39.2341</d>
<d>39.28431</d>
<d>39.33452</d>
<d>39.38473</d>
<d>39.43494</d>
<d>39.48515</d>
<d>39.53536</d>
<d>39.58557</d>
<d>39.63578</d>
<d>39.68599</d>
<d>39.7362</d>
<d>39.78641</d>
<d>39.83663</d>
<d>39.88684</d>
<d>39.93705</d>
<d>39.98726</d>
<d>40.03747</d>
<d>40.08768</d>
<d>40.13789</d>
<d>40.1881</d>
<d>40.23831</d>
<d>40.28852</d>
<d>40.33873</d>
<d>40.38894</d>
<d>40.43915</d>
<d>40.48936</d>
<d>40.53957</d>
<d>40.58978</d>
<d>40.63999</d>
<d>40.6902</d>
<d>40.74041</d>
<d>40.79062</d>
<d>40.84083</d>
<d>40.89104</d>
<d>40.94125</d>
<d>40.99146</d>
<d>41.04167</d>
<d>41.09188</d>
<d>41.14209</d>
<d>41.1923</d>
<d>41.24251</d>
<d>41.29272</d>
<d>41.34293</d>
<d>41.39314</d>
<d>41.44335</d>
<d>41.49356</d>
<d>41.54377</d>
<d>41.59398</d>
<d>41.64419</d>
<d>41.6944</d>
<d>41.74461</d>
<d>41.79482</d>
<d>41.84503</d>
<d>41.89524</d>
<d>41.94545</d>
<d>41.99566</d>
<d>42.04587</d>
<d>42.09608</d>
<d>42.14629</d>
<d>42.1965</d>
<d>42.24671</d>
<d>42.29692</d>
<d>42.34713</d>
<d>42.39734</d>
<d>42.44756</d>
<d>42.49777</d>
<d>42.54798</d>
<d>42.59819</d>
<d>42.6484</d>
<d>42.69861</d>
<d>42.74882</d>
<d>42.79903</d>
<d>42.84924</d>
<d>42.89945</d>
<d>42.94966</d>
<d>42.99987</d>
<d>43.05008</d>
<d>43.10029</d>
<d>43.1505</d>
<d>43.20071</d>
<d>43.25092</d>
<d>43.30113</d>
<d>43.35134</d>
<d>43.40155</d>
<d>43.45176</d>
<d>43.50197</d>
<d>43.55218</d>
<d>43.60239</d>
<d>43.6526</d>
<d>43.70281</d>
<d>43.75302</d>
<d>43.80323</d>
<d>43.85344</d>
<d>43.90365</d>
<d>43.95386</d>
<d>44.00407</d>
<d>44.05428</d>
<d>44.10449</d>
<d>44.1547</d>
<d>44.20491</d>
<d>44.25512</d>
<d>44.30533</d>
<d>44.35554</d>
<d>44.40575</d>
<d>44.45596</d>
<d>44.50617</d>
<d>44.55638</d>
<d>44.60659</d>
<d>44.6568</d>
<d>44.70701</d>
<d>44.75722</d>
<d>44.80743</d>
<d>44.85764</d>
<d>44.90785</d>
<d>44.95806</d>
<d>45.00827</d>
<d>45.05849</d>
<d>45.1087</d>
<d>45.15891</d>
<d>45.20912</d>
<d>45.25933</d>
<d>45.30954</d>
<d>45.35975</d>
<d>45.40996</d>
<d>45.46017</d>
<d>45.51038</d>
<d>45.56059</d>
<d>45.6108</d>
<d>45.66101</d>
<d>45.71122</d>
<d>45.76143</d>
<d>45.81164</d>
<d>45.86185</d>
<d>45.91206</d>
<d>45.96227</d>
<d>46.01248</d>
<d>46.06269</d>
<d>46.1129</d>
<d>46.16311</d>
<d>46.21332</d>
<d>46.26353</d>
<d>46.31374</d>
<d>46.36395</d>
<d>46.41416</d>
<d>46.46437</d>
<d>46.51458</d>
<d>46.56479</d>
<d>46.615</d>
<d>46.66521</d>
<d>46.71542</d>
<d>46.76563</d>
<d>46.81584</d>
<d>46.86605</d>
<d>46.91626</d>
<d>46.96647</d>
<d>47.01668</d>
<d>47.06689</d>
<d>47.1171</d>
<d>47.16731</d>
<d>47.21752</d>
<d>47.26773</d>
<d>47.31794</d>
<d>47.36815</d>
<d>47.41836</d>
<d>47.46857</d>
<d>47.51878</d>
<d>47.56899</d>
<d>47.61921</d>
<d>47.66942</d>
<d>47.71963</d>
<d>47.76984</d>
<d>47.82005</d>
<d>47.87026</d>
<d>47.92047</d>
<d>47.97068</d>
<d>48.02089</d>
<d>48.0711</d>
<d>48.12131</d>
<d>48.17152</d>
<d>48.22173</d>
<d>48.27194</d>
<d>48.32215</d>
<d>48.37236</d>
<d>48.42257</d>
<d>48.47278</d>
<d>48.52299</d>
<d>48.5732</d>
<d>48.62341</d>
<d>48.67362</d>
<d>48.72383</d>
<d>48.77404</d>
<d>48.82425</d>
<d>48.87446</d>
<d>48.92467</d>
<d>48.97488</d>
<d>49.02509</d>
<d>49.0753</d>
<d>49.12551</d>
<d>49.17572</d>
<d>49.22593</d>
<d>49.27614</d>
<d>49.32635</d>
<d>49.37656</d>
<d>49.42677</d>
<d>49.47698</d>
<d>49.52719</d>
<d>49.5774</d>
<d>49.62761</d>
<d>49.67782</d>
<d>49.72803</d>
<d>49.77824</d>
<d>49.82845</d>
<d>49.87866</d>
<d>49.92887</d>
<d>49.97908</d>
<d>50.02929</d>
<d>50.0795</d>
<d>50.12971</d>
<d>50.17992</d>
<d>50.23014</d>
<d>50.28035</d>
<d>50.33056</d>
<d>50.38077</d>
<d>50.43098</d>
<d>50.48119</d>
<d>50.5314</d>
<d>50.58161</d>
<d>50.63182</d>
<d>50.68203</d>
<d>50.73224</d>
<d>50.78245</d>
<d>50.83266</d>
<d>50.88287</d>
<d>50.93308</d>
<d>50.98329</d>
<d>51.0335</d>
<d>51.08371</d>
<d>51.13392</d>
<d>51.18413</d>
<d>51.23434</d>
<d>51.28455</d>
<d>51.33476</d>
<d>51.38497</d>
<d>51.43518</d>
<d>51.48539</d>
<d>51.5356</d>
<d>51.58581</d>
<d>51.63602</d>
<d>51.68623</d>
<d>51.73644</d>
<d>51.78665</d>
<d>51.83686</d>
<d>51.88707</d>
<d>51.93728</d>
<d>51.98749</d>
<d>52.0377</d>
<d>52.08791</d>
<d>52.13812</d>
<d>52.18833</d>
<d>52.23854</d>
<d>52.28875</d>
<d>52.33896</d>
<d>52.38917</d>
<d>52.43938</d>
<d>52.48959</d>
<d>52.5398</d>
<d>52.59001</d>
<d>52.64022</d>
<d>52.69043</d>
<d>52.74064</d>
<d>52.79085</d>
<d>52.84107</d>
<d>52.89128</d>
<d>52.94149</d>
<d>52.9917</d>
<d>53.04191</d>
<d>53.09212</d>
<d>53.14233</d>
<d>53.19254</d>
<d>53.24275</d>
<d>53.29296</d>
<d>53.34317</d>
<d>53.39338</d>
<d>53.44359</d>
<d>53.4938</d>
<d>53.54401</d>
<d>53.59422</d>
<d>53.64443</d>
<d>53.69464</d>
<d>53.74485</d>
<d>53.79506</d>
<d>53.84527</d>
<d>53.89548</d>
<d>53.94569</d>
<d>53.9959</d>
<d>54.04611</d>
<d>54.09632</d>
<d>54.14653</d>
<d>54.19674</d>
<d>54.24695</d>
<d>54.29716</d>
<d>54.34737</d>
<d>54.39758</d>
<d>54.44779</d>
<d>54.498</d>
<d>54.54821</d>
<d>54.59842</d>
<d>54.64863</d>
<d>54.69884</d>
<d>54.74905</d>
<d>54.79926</d>
<d>54.84947</d>
<d>54.89968</d>
<d>54.94989</d>
<d>55.0001</d>
<d>55.05031</d>
<d>55.10052</d>
<d>55.15073</d>
<d>55.20094</d>
<d>55.25115</d>
<d>55.30136</d>
<d>55.35157</d>
<d>55.40178</d>
<d>55.452</d>
<d>55.50221</d>
<d>55.55242</d>
<d>55.60263</d>
<d>55.65284</d>
<d>55.70305</d>
<d>55.75326</d>
<d>55.80347</d>
<d>55.85368</d>
<d>55.90389</d>
<d>55.9541</d>
<d>56.00431</d>
<d>56.05452</d>
<d>56.10473</d>
<d>56.15494</d>
<d>56.20515</d>
<d>56.25536</d>
<d>56.30557</d>
<d>56.35578</d>
<d>56.40599</d>
<d>56.4562</d>
<d>56.50641</d>
<d>56.55662</d>
<d>56.60683</d>
<d>56.65704</d>
<d>56.70725</d>
<d>56.75746</d>
<d>56.80767</d>
<d>56.85788</d>
<d>56.90809</d>
<d>56.9583</d>
<d>57.00851</d>
<d>57.05872</d>
<d>57.10893</d>
<d>57.15914</d>
<d>57.20935</d>
<d>57.25956</d>
<d>57.30977</d>
<d>57.35998</d>
<d>57.41019</d>
<d>57.4604</d>
<d>57.51061</d>
<d>57.56082</d>
<d>57.61103</d>
<d>57.66124</d>
<d>57.71145</d>
<d>57.76166</d>
<d>57.81187</d>
<d>57.86208</d>
<d>57.91229</d>
<d>57.9625</d>
<d>58.01271</d>
<d>58.06293</d>
<d>58.11314</d>
<d>58.16335</d>
<d>58.21356</d>
<d>58.26377</d>
<d>58.31398</d>
<d>58.36419</d>
<d>58.4144</d>
<d>58.46461</d>
<d>58.51482</d>
<d>58.56503</d>
<d>58.61524</d>
<d>58.66545</d>
<d>58.71566</d>
<d>58.76587</d>
<d>58.81608</d>
<d>58.86629</d>
<d>58.9165</d>
<d>58.96671</d>
<d>59.01692</d>
<d>59.06713</d>
<d>59.11734</d>
<d>59.16755</d>
<d>59.21776</d>
<d>59.26797</d>
<d>59.31818</d>
<d>59.36839</d>
<d>59.4186</d>
<d>59.46881</d>
<d>59.51902</d>
<d>59.56923</d>
<d>59.61944</d>
<d>59.66965</d>
<d>59.71986</d>
<d>59.77007</d>
<d>59.82028</d>
<d>59.87049</d>
<d>59.9207</d>
<d>59.97091</d>
<d>60.02112</d>
<d>60.07133</d>
<d>60.12154</d>
<d>60.17175</d>
<d>60.22196</d>
<d>60.27217</d>
<d>60.32238</d>
<d>60.37259</d>
<d>60.4228</d>
<d>60.47301</d>
<d>60.52322</d>
<d>60.57343</d>
<d>60.62364</d>
<d>60.67386</d>
<d>60.72407</d>
<d>60.77428</d>
<d>60.82449</d>
<d>60.8747</d>
<d>60.92491</d>
<d>60.97512</d>
<d>61.02533</d>
<d>61.07554</d>
<d>61.12575</d>
<d>61.17596</d>
<d>61.22617</d>
<d>61.27638</d>
<d>61.32659</d>
<d>61.3768</d>
<d>61.42701</d>
<d>61.47722</d>
<d>61.52743</d>
<d>61.57764</d>
<d>61.62785</d>
<d>61.67806</d>
<d>61.72827</d>
<d>61.77848</d>
<d>61.82869</d>
<d>61.8789</d>
<d>61.92911</d>
<d>61.97932</d>
<d>62.02953</d>
<d>62.07974</d>
<d>62.12995</d>
<d>62.18016</d>
<d>62.23037</d>
<d>62.28058</d>
<d>62.33079</d>
<d>62.381</d>
<d>62.43121</d>
<d>62.48142</d>
<d>62.53163</d>
<d>62.58184</d>
<d>62.63205</d>
<d>62.68226</d>
<d>62.73247</d>
<d>62.78268</d>
<d>62.83289</d>
<d>62.8831</d>
<d>62.93331</d>
<d>62.98352</d>
<d>63.03373</d>
<d>63.08394</d>
<d>63.13415</d>
<d>63.18436</d>
<d>63.23457</d>
<d>63.28479</d>
<d>63.335</d>
<d>63.38521</d>
<d>63.43542</d>
<d>63.48563</d>
<d>63.53584</d>
<d>63.58605</d>
<d>63.63626</d>
<d>63.68647</d>
<d>63.73668</d>
<d>63.78689</d>
<d>63.8371</d>
<d>63.88731</d>
<d>63.93752</d>
<d>63.98773</d>
<d>64.03794</d>
<d>64.08815</d>
<d>64.13836</d>
<d>64.18857</d>
<d>64.23878</d>
<d>64.28899</d>
<d>64.3392</d>
<d>64.38941</d>
<d>64.43962</d>
<d>64.48983</d>
<d>64.54004</d>
<d>64.59025</d>
<d>64.64046</d>
<d>64.69067</d>
<d>64.74088</d>
<d>64.79109</d>
<d>64.8413</d>
<d>64.89151</d>
<d>64.94172</d>
<d>64.99193</d>
<d>65.04214</d>
<d>65.09235</d>
<d>65.14256</d>
<d>65.19277</d>
<d>65.24298</d>
<d>65.29319</d>
<d>65.3434</d>
<d>65.39361</d>
<d>65.44382</d>
<d>65.49403</d>
<d>65.54424</d>
<d>65.59445</d>
<d>65.64466</d>
<d>65.69487</d>
<d>65.74508</d>
<d>65.79529</d>
<d>65.8455</d>
<d>65.89572</d>
<d>65.94593</d>
<d>65.99614</d>
<d>66.04635</d>
<d>66.09656</d>
<d>66.14677</d>
<d>66.19698</d>
<d>66.24719</d>
<d>66.2974</d>
<d>66.34761</d>
<d>66.39782</d>
<d>66.44803</d>
<d>66.49824</d>
<d>66.54845</d>
<d>66.59866</d>
<d>66.64887</d>
<d>66.69908</d>
<d>66.74929</d>
<d>66.7995</d>
<d>66.84971</d>
<d>66.89992</d>
<d>66.95013</d>
<d>67.00034</d>
<d>67.05055</d>
<d>67.10076</d>
<d>67.15097</d>
<d>67.20118</d>
<d>67.25139</d>
<d>67.3016</d>
<d>67.35181</d>
<d>67.40202</d>
<d>67.45223</d>
<d>67.50244</d>
<d>67.55265</d>
<d>67.60286</d>
<d>67.65307</d>
<d>67.70328</d>
<d>67.75349</d>
<d>67.8037</d>
<d>67.85391</d>
<d>67.90412</d>
<d>67.95433</d>
<d>68.00454</d>
<d>68.05475</d>
<d>68.10496</d>
<d>68.15517</d>
<d>68.20538</d>
<d>68.25559</d>
<d>68.3058</d>
<d>68.35601</d>
<d>68.40622</d>
<d>68.45644</d>
<d>68.50665</d>
<d>68.55686</d>
<d>68.60707</d>
<d>68.65728</d>
<d>68.70749</d>
<d>68.7577</d>
<d>68.80791</d>
<d>68.85812</d>
<d>68.90833</d>
<d>68.95854</d>
<d>69.00875</d>
<d>69.05896</d>
<d>69.10917</d>
<d>69.15938</d>
<d>69.20959</d>
<d>69.2598</d>
<d>69.31001</d>
<d>69.36022</d>
<d>69.41043</d>
<d>69.46064</d>
<d>69.51085</d>
<d>69.56106</d>
<d>69.61127</d>
<d>69.66148</d>
<d>69.71169</d>
<d>69.7619</d>
<d>69.81211</d>
<d>69.86232</d>
<d>69.91253</d>
<d>69.96274</d>
<d>70.01295</d>
<d>70.06316</d>
<d>70.11337</d>
<d>70.16358</d>
<d>70.21379</d>
<d>70.264</d>
<d>70.31421</d>
<d>70.36442</d>
<d>70.41463</d>
<d>70.46484</d>
<d>70.51505</d>
<d>70.56526</d>
<d>70.61547</d>
<d>70.66568</d>
<d>70.71589</d>
<d>70.7661</d>
<d>70.81631</d>
<d>70.86652</d>
<d>70.91673</d>
<d>70.96694</d>
<d>71.01715</d>
<d>71.06737</d>
<d>71.11758</d>
<d>71.16779</d>
<d>71.218</d>
<d>71.26821</d>
<d>71.31842</d>
<d>71.36863</d>
<d>71.41884</d>
<d>71.46905</d>
<d>71.51926</d>
<d>71.56947</d>
<d>71.61968</d>
<d>71.66989</d>
<d>71.7201</d>
<d>71.77031</d>
<d>71.82052</d>
<d>71.87073</d>
<d>71.92094</d>
<d>71.97115</d>
<d>72.02136</d>
<d>72.07157</d>
<d>72.12178</d>
<d>72.17199</d>
<d>72.2222</d>
<d>72.27241</d>
<d>72.32262</d>
<d>72.37283</d>
<d>72.42304</d>
<d>72.47325</d>
<d>72.52346</d>
<d>72.57367</d>
<d>72.62388</d>
<d>72.67409</d>
<d>72.7243</d>
<d>72.77451</d>
<d>72.82472</d>
<d>72.87493</d>
<d>72.92514</d>
<d>72.97535</d>
<d>73.02556</d>
<d>73.07577</d>
<d>73.12598</d>
<d>73.17619</d>
<d>73.2264</d>
<d>73.27661</d>
<d>73.32682</d>
<d>73.37703</d>
<d>73.42724</d>
<d>73.47745</d>
<d>73.52766</d>
<d>73.57787</d>
<d>73.62808</d>
<d>73.6783</d>
<d>73.72851</d>
<d>73.77872</d>
<d>73.82893</d>
<d>73.87914</d>
<d>73.92935</d>
<d>73.97956</d>
<d>74.02977</d>
<d>74.07998</d>
<d>74.13019</d>
<d>74.1804</d>
<d>74.23061</d>
<d>74.28082</d>
<d>74.33103</d>
<d>74.38124</d>
<d>74.43145</d>
<d>74.48166</d>
<d>74.53187</d>
<d>74.58208</d>
<d>74.63229</d>
<d>74.6825</d>
<d>74.73271</d>
<d>74.78292</d>
<d>74.83313</d>
<d>74.88334</d>
<d>74.93355</d>
<d>74.98376</d>
<d>75.03397</d>
<d>75.08418</d>
<d>75.13439</d>
<d>75.1846</d>
<d>75.23481</d>
<d>75.28502</d>
<d>75.33523</d>
<d>75.38544</d>
<d>75.43565</d>
<d>75.48586</d>
<d>75.53607</d>
<d>75.58628</d>
<d>75.63649</d>
<d>75.6867</d>
<d>75.73691</d>
<d>75.78712</d>
<d>75.83733</d>
<d>75.88754</d>
<d>75.93775</d>
<d>75.98796</d>
<d>76.03817</d>
<d>76.08838</d>
<d>76.13859</d>
<d>76.1888</d>
<d>76.23901</d>
<d>76.28923</d>
<d>76.33944</d>
<d>76.38965</d>
<d>76.43986</d>
<d>76.49007</d>
<d>76.54028</d>
<d>76.59049</d>
<d>76.6407</d>
<d>76.69091</d>
<d>76.74112</d>
<d>76.79133</d>
<d>76.84154</d>
<d>76.89175</d>
<d>76.94196</d>
<d>76.99217</d>
<d>77.04238</d>
<d>77.09259</d>
<d>77.1428</d>
<d>77.19301</d>
<d>77.24322</d>
<d>77.29343</d>
<d>77.34364</d>
<d>77.39385</d>
<d>77.44406</d>
<d>77.49427</d>
<d>77.54448</d>
<d>77.59469</d>
<d>77.6449</d>
<d>77.69511</d>
<d>77.74532</d>
<d>77.79553</d>
<d>77.84574</d>
<d>77.89595</d>
<d>77.94616</d>
<d>77.99637</d>
<d>78.04658</d>
<d>78.09679</d>
<d>78.147</d>
<d>78.19721</d>
<d>78.24742</d>
<d>78.29763</d>
<d>78.34784</d>
<d>78.39805</d>
<d>78.44826</d>
<d>78.49847</d>
<d>78.54868</d>
<d>78.59889</d>
<d>78.6491</d>
<d>78.69931</d>
<d>78.74952</d>
<d>78.79973</d>
<d>78.84994</d>
<d>78.90016</d>
<d>78.95037</d>
<d>79.00058</d>
<d>79.05079</d>
<d>79.101</d>
<d>79.15121</d>
</Subcolumn>
</XColumn>
<XAdvancedColumn Version="1" Width="114" Decimals="6" Subcolumns="1">
<Title>2 theta</Title>
<Subcolumn>
<d/>
<d>7.2</d>
<d>7.25021</d>
<d>7.30042</d>
<d>7.35063</d>
<d>7.400841</d>
<d>7.451051</d>
<d>7.501261</d>
<d>7.551471</d>
<d>7.601681</d>
<d>7.651892</d>
<d>7.702102</d>
<d>7.752312</d>
<d>7.802522</d>
<d>7.852732</d>
<d>7.902943</d>
<d>7.953153</d>
<d>8.003363</d>
<d>8.053573</d>
<d>8.103783</d>
<d>8.153993</d>
<d>8.204204</d>
<d>8.254414</d>
<d>8.304624</d>
<d>8.354834</d>
<d>8.405044</d>
<d>8.455255</d>
<d>8.505465</d>
<d>8.555675</d>
<d>8.605885</d>
<d>8.656095</d>
<d>8.706306</d>
<d>8.756516</d>
<d>8.806726</d>
<d>8.856936</d>
<d>8.907146</d>
<d>8.957357</d>
<d>9.007567</d>
<d>9.057777</d>
<d>9.107987</d>
<d>9.158197</d>
<d>9.208408</d>
<d>9.258618</d>
<d>9.308828</d>
<d>9.359038</d>
<d>9.409248</d>
<d>9.459458</d>
<d>9.509669</d>
<d>9.559879</d>
<d>9.610089</d>
<d>9.660299</d>
<d>9.710509</d>
<d>9.76072</d>
<d>9.81093</d>
<d>9.86114</d>
<d>9.91135</d>
<d>9.96156</d>
<d>10.01177</d>
<d>10.06198</d>
<d>10.11219</d>
<d>10.1624</d>
<d>10.21261</d>
<d>10.26282</d>
<d>10.31303</d>
<d>10.36324</d>
<d>10.41345</d>
<d>10.46366</d>
<d>10.51387</d>
<d>10.56408</d>
<d>10.61429</d>
<d>10.6645</d>
<d>10.71471</d>
<d>10.76492</d>
<d>10.81513</d>
<d>10.86534</d>
<d>10.91555</d>
<d>10.96576</d>
<d>11.01597</d>
<d>11.06618</d>
<d>11.11639</d>
<d>11.16661</d>
<d>11.21682</d>
<d>11.26703</d>
<d>11.31724</d>
<d>11.36745</d>
<d>11.41766</d>
<d>11.46787</d>
<d>11.51808</d>
<d>11.56829</d>
<d>11.6185</d>
<d>11.66871</d>
<d>11.71892</d>
<d>11.76913</d>
<d>11.81934</d>
<d>11.86955</d>
<d>11.91976</d>
<d>11.96997</d>
<d>12.02018</d>
<d>12.07039</d>
<d>12.1206</d>
<d>12.17081</d>
<d>12.22102</d>
<d>12.27123</d>
<d>12.32144</d>
<d>12.37165</d>
<d>12.42186</d>
<d>12.47207</d>
<d>12.52228</d>
<d>12.57249</d>
<d>12.6227</d>
<d>12.67291</d>
<d>12.72312</d>
<d>12.77333</d>
<d>12.82354</d>
<d>12.87375</d>
<d>12.92396</d>
<d>12.97417</d>
<d>13.02438</d>
<d>13.07459</d>
<d>13.1248</d>
<d>13.17501</d>
<d>13.22522</d>
<d>13.27543</d>
<d>13.32564</d>
<d>13.37585</d>
<d>13.42606</d>
<d>13.47627</d>
<d>13.52648</d>
<d>13.57669</d>
<d>13.6269</d>
<d>13.67711</d>
<d>13.72732</d>
<d>13.77754</d>
<d>13.82775</d>
<d>13.87796</d>
<d>13.92817</d>
<d>13.97838</d>
<d>14.02859</d>
<d>14.0788</d>
<d>14.12901</d>
<d>14.17922</d>
<d>14.22943</d>
<d>14.27964</d>
<d>14.32985</d>
<d>14.38006</d>
<d>14.43027</d>
<d>14.48048</d>
<d>14.53069</d>
<d>14.5809</d>
<d>14.63111</d>
<d>14.68132</d>
<d>14.73153</d>
<d>14.78174</d>
<d>14.83195</d>
<d>14.88216</d>
<d>14.93237</d>
<d>14.98258</d>
<d>15.03279</d>
<d>15.083</d>
<d>15.13321</d>
<d>15.18342</d>
<d>15.23363</d>
<d>15.28384</d>
<d>15.33405</d>
<d>15.38426</d>
<d>15.43447</d>
<d>15.48468</d>
<d>15.53489</d>
<d>15.5851</d>
<d>15.63531</d>
<d>15.68552</d>
<d>15.73573</d>
<d>15.78594</d>
<d>15.83615</d>
<d>15.88636</d>
<d>15.93657</d>
<d>15.98678</d>
<d>16.03699</d>
<d>16.0872</d>
<d>16.13741</d>
<d>16.18762</d>
<d>16.23783</d>
<d>16.28804</d>
<d>16.33825</d>
<d>16.38847</d>
<d>16.43868</d>
<d>16.48889</d>
<d>16.5391</d>
<d>16.58931</d>
<d>16.63952</d>
<d>16.68973</d>
<d>16.73994</d>
<d>16.79015</d>
<d>16.84036</d>
<d>16.89057</d>
<d>16.94078</d>
<d>16.99099</d>
<d>17.0412</d>
<d>17.09141</d>
<d>17.14162</d>
<d>17.19183</d>
<d>17.24204</d>
<d>17.29225</d>
<d>17.34246</d>
<d>17.39267</d>
<d>17.44288</d>
<d>17.49309</d>
<d>17.5433</d>
<d>17.59351</d>
<d>17.64372</d>
<d>17.69393</d>
<d>17.74414</d>
<d>17.79435</d>
<d>17.84456</d>
<d>17.89477</d>
<d>17.94498</d>
<d>17.99519</d>
<d>18.0454</d>
<d>18.09561</d>
<d>18.14582</d>
<d>18.19603</d>
<d>18.24624</d>
<d>18.29645</d>
<d>18.34666</d>
<d>18.39687</d>
<d>18.44708</d>
<d>18.49729</d>
<d>18.5475</d>
<d>18.59771</d>
<d>18.64792</d>
<d>18.69813</d>
<d>18.74834</d>
<d>18.79855</d>
<d>18.84876</d>
<d>18.89897</d>
<d>18.94918</d>
<d>18.9994</d>
<d>19.04961</d>
<d>19.09982</d>
<d>19.15003</d>
<d>19.20024</d>
<d>19.25045</d>
<d>19.30066</d>
<d>19.35087</d>
<d>19.40108</d>
<d>19.45129</d>
<d>19.5015</d>
<d>19.55171</d>
<d>19.60192</d>
<d>19.65213</d>
<d>19.70234</d>
<d>19.75255</d>
<d>19.80276</d>
<d>19.85297</d>
<d>19.90318</d>
<d>19.95339</d>
<d>20.0036</d>
<d>20.05381</d>
<d>20.10402</d>
<d>20.15423</d>
<d>20.20444</d>
<d>20.25465</d>
<d>20.30486</d>
<d>20.35507</d>
<d>20.40528</d>
<d>20.45549</d>
<d>20.5057</d>
<d>20.55591</d>
<d>20.60612</d>
<d>20.65633</d>
<d>20.70654</d>
<d>20.75675</d>
<d>20.80696</d>
<d>20.85717</d>
<d>20.90738</d>
<d>20.95759</d>
<d>21.0078</d>
<d>21.05801</d>
<d>21.10822</d>
<d>21.15843</d>
<d>21.20864</d>
<d>21.25885</d>
<d>21.30906</d>
<d>21.35927</d>
<d>21.40948</d>
<d>21.45969</d>
<d>21.5099</d>
<d>21.56011</d>
<d>21.61033</d>
<d>21.66054</d>
<d>21.71075</d>
<d>21.76096</d>
<d>21.81117</d>
<d>21.86138</d>
<d>21.91159</d>
<d>21.9618</d>
<d>22.01201</d>
<d>22.06222</d>
<d>22.11243</d>
<d>22.16264</d>
<d>22.21285</d>
<d>22.26306</d>
<d>22.31327</d>
<d>22.36348</d>
<d>22.41369</d>
<d>22.4639</d>
<d>22.51411</d>
<d>22.56432</d>
<d>22.61453</d>
<d>22.66474</d>
<d>22.71495</d>
<d>22.76516</d>
<d>22.81537</d>
<d>22.86558</d>
<d>22.91579</d>
<d>22.966</d>
<d>23.01621</d>
<d>23.06642</d>
<d>23.11663</d>
<d>23.16684</d>
<d>23.21705</d>
<d>23.26726</d>
<d>23.31747</d>
<d>23.36768</d>
<d>23.41789</d>
<d>23.4681</d>
<d>23.51831</d>
<d>23.56852</d>
<d>23.61873</d>
<d>23.66894</d>
<d>23.71915</d>
<d>23.76936</d>
<d>23.81957</d>
<d>23.86978</d>
<d>23.91999</d>
<d>23.9702</d>
<d>24.02041</d>
<d>24.07062</d>
<d>24.12083</d>
<d>24.17104</d>
<d>24.22126</d>
<d>24.27147</d>
<d>24.32168</d>
<d>24.37189</d>
<d>24.4221</d>
<d>24.47231</d>
<d>24.52252</d>
<d>24.57273</d>
<d>24.62294</d>
<d>24.67315</d>
<d>24.72336</d>
<d>24.77357</d>
<d>24.82378</d>
<d>24.87399</d>
<d>24.9242</d>
<d>24.97441</d>
<d>25.02462</d>
<d>25.07483</d>
<d>25.12504</d>
<d>25.17525</d>
<d>25.22546</d>
<d>25.27567</d>
<d>25.32588</d>
<d>25.37609</d>
<d>25.4263</d>
<d>25.47651</d>
<d>25.52672</d>
<d>25.57693</d>
<d>25.62714</d>
<d>25.67735</d>
<d>25.72756</d>
<d>25.77777</d>
<d>25.82798</d>
<d>25.87819</d>
<d>25.9284</d>
<d>25.97861</d>
<d>26.02882</d>
<d>26.07903</d>
<d>26.12924</d>
<d>26.17945</d>
<d>26.22966</d>
<d>26.27987</d>
<d>26.33008</d>
<d>26.38029</d>
<d>26.4305</d>
<d>26.48071</d>
<d>26.53092</d>
<d>26.58113</d>
<d>26.63134</d>
<d>26.68155</d>
<d>26.73176</d>
<d>26.78198</d>
<d>26.83219</d>
<d>26.8824</d>
<d>26.93261</d>
<d>26.98282</d>
<d>27.03303</d>
<d>27.08324</d>
<d>27.13345</d>
<d>27.18366</d>
<d>27.23387</d>
<d>27.28408</d>
<d>27.33429</d>
<d>27.3845</d>
<d>27.43471</d>
<d>27.48492</d>
<d>27.53513</d>
<d>27.58534</d>
<d>27.63555</d>
<d>27.68576</d>
<d>27.73597</d>
<d>27.78618</d>
<d>27.83639</d>
<d>27.8866</d>
<d>27.93681</d>
<d>27.98702</d>
<d>28.03723</d>
<d>28.08744</d>
<d>28.13765</d>
<d>28.18786</d>
<d>28.23807</d>
<d>28.28828</d>
<d>28.33849</d>
<d>28.3887</d>
<d>28.43891</d>
<d>28.48912</d>
<d>28.53933</d>
<d>28.58954</d>
<d>28.63975</d>
<d>28.68996</d>
<d>28.74017</d>
<d>28.79038</d>
<d>28.84059</d>
<d>28.8908</d>
<d>28.94101</d>
<d>28.99122</d>
<d>29.04143</d>
<d>29.09164</d>
<d>29.14185</d>
<d>29.19206</d>
<d>29.24227</d>
<d>29.29248</d>
<d>29.34269</d>
<d>29.39291</d>
<d>29.44312</d>
<d>29.49333</d>
<d>29.54354</d>
<d>29.59375</d>
<d>29.64396</d>
<d>29.69417</d>
<d>29.74438</d>
<d>29.79459</d>
<d>29.8448</d>
<d>29.89501</d>
<d>29.94522</d>
<d>29.99543</d>
<d>30.04564</d>
<d>30.09585</d>
<d>30.14606</d>
<d>30.19627</d>
<d>30.24648</d>
<d>30.29669</d>
<d>30.3469</d>
<d>30.39711</d>
<d>30.44732</d>
<d>30.49753</d>
<d>30.54774</d>
<d>30.59795</d>
<d>30.64816</d>
<d>30.69837</d>
<d>30.74858</d>
<d>30.79879</d>
<d>30.849</d>
<d>30.89921</d>
<d>30.94942</d>
<d>30.99963</d>
<d>31.04984</d>
<d>31.10005</d>
<d>31.15026</d>
<d>31.20047</d>
<d>31.25068</d>
<d>31.30089</d>
<d>31.3511</d>
<d>31.40131</d>
<d>31.45152</d>
<d>31.50173</d>
<d>31.55194</d>
<d>31.60215</d>
<d>31.65236</d>
<d>31.70257</d>
<d>31.75278</d>
<d>31.80299</d>
<d>31.8532</d>
<d>31.90341</d>
<d>31.95362</d>
<d>32.00384</d>
<d>32.05405</d>
<d>32.10426</d>
<d>32.15447</d>
<d>32.20468</d>
<d>32.25489</d>
<d>32.3051</d>
<d>32.35531</d>
<d>32.40552</d>
<d>32.45573</d>
<d>32.50594</d>
<d>32.55615</d>
<d>32.60636</d>
<d>32.65657</d>
<d>32.70678</d>
<d>32.75699</d>
<d>32.8072</d>
<d>32.85741</d>
<d>32.90762</d>
<d>32.95783</d>
<d>33.00804</d>
<d>33.05825</d>
<d>33.10846</d>
<d>33.15867</d>
<d>33.20888</d>
<d>33.25909</d>
<d>33.3093</d>
<d>33.35951</d>
<d>33.40972</d>
<d>33.45993</d>
<d>33.51014</d>
<d>33.56035</d>
<d>33.61056</d>
<d>33.66077</d>
<d>33.71098</d>
<d>33.76119</d>
<d>33.8114</d>
<d>33.86161</d>
<d>33.91182</d>
<d>33.96203</d>
<d>34.01224</d>
<d>34.06245</d>
<d>34.11266</d>
<d>34.16287</d>
<d>34.21308</d>
<d>34.26329</d>
<d>34.3135</d>
<d>34.36371</d>
<d>34.41392</d>
<d>34.46413</d>
<d>34.51434</d>
<d>34.56455</d>
<d>34.61477</d>
<d>34.66498</d>
<d>34.71519</d>
<d>34.7654</d>
<d>34.81561</d>
<d>34.86582</d>
<d>34.91603</d>
<d>34.96624</d>
<d>35.01645</d>
<d>35.06666</d>
<d>35.11687</d>
<d>35.16708</d>
<d>35.21729</d>
<d>35.2675</d>
<d>35.31771</d>
<d>35.36792</d>
<d>35.41813</d>
<d>35.46834</d>
<d>35.51855</d>
<d>35.56876</d>
<d>35.61897</d>
<d>35.66918</d>
<d>35.71939</d>
<d>35.7696</d>
<d>35.81981</d>
<d>35.87002</d>
<d>35.92023</d>
<d>35.97044</d>
<d>36.02065</d>
<d>36.07086</d>
<d>36.12107</d>
<d>36.17128</d>
<d>36.22149</d>
<d>36.2717</d>
<d>36.32191</d>
<d>36.37212</d>
<d>36.42233</d>
<d>36.47254</d>
<d>36.52275</d>
<d>36.57296</d>
<d>36.62317</d>
<d>36.67338</d>
<d>36.72359</d>
<d>36.7738</d>
<d>36.82401</d>
<d>36.87422</d>
<d>36.92443</d>
<d>36.97464</d>
<d>37.02485</d>
<d>37.07506</d>
<d>37.12527</d>
<d>37.17548</d>
<d>37.2257</d>
<d>37.27591</d>
<d>37.32612</d>
<d>37.37633</d>
<d>37.42654</d>
<d>37.47675</d>
<d>37.52696</d>
<d>37.57717</d>
<d>37.62738</d>
<d>37.67759</d>
<d>37.7278</d>
<d>37.77801</d>
<d>37.82822</d>
<d>37.87843</d>
<d>37.92864</d>
<d>37.97885</d>
<d>38.02906</d>
<d>38.07927</d>
<d>38.12948</d>
<d>38.17969</d>
<d>38.2299</d>
<d>38.28011</d>
<d>38.33032</d>
<d>38.38053</d>
<d>38.43074</d>
<d>38.48095</d>
<d>38.53116</d>
<d>38.58137</d>
<d>38.63158</d>
<d>38.68179</d>
<d>38.732</d>
<d>38.78221</d>
<d>38.83242</d>
<d>38.88263</d>
<d>38.93284</d>
<d>38.98305</d>
<d>39.03326</d>
<d>39.08347</d>
<d>39.13368</d>
<d>39.18389</d>
<d>39.2341</d>
<d>39.28431</d>
<d>39.33452</d>
<d>39.38473</d>
<d>39.43494</d>
<d>39.48515</d>
<d>39.53536</d>
<d>39.58557</d>
<d>39.63578</d>
<d>39.68599</d>
<d>39.7362</d>
<d>39.78641</d>
<d>39.83663</d>
<d>39.88684</d>
<d>39.93705</d>
<d>39.98726</d>
<d>40.03747</d>
<d>40.08768</d>
<d>40.13789</d>
<d>40.1881</d>
<d>40.23831</d>
<d>40.28852</d>
<d>40.33873</d>
<d>40.38894</d>
<d>40.43915</d>
<d>40.48936</d>
<d>40.53957</d>
<d>40.58978</d>
<d>40.63999</d>
<d>40.6902</d>
<d>40.74041</d>
<d>40.79062</d>
<d>40.84083</d>
<d>40.89104</d>
<d>40.94125</d>
<d>40.99146</d>
<d>41.04167</d>
<d>41.09188</d>
<d>41.14209</d>
<d>41.1923</d>
<d>41.24251</d>
<d>41.29272</d>
<d>41.34293</d>
<d>41.39314</d>
<d>41.44335</d>
<d>41.49356</d>
<d>41.54377</d>
<d>41.59398</d>
<d>41.64419</d>
<d>41.6944</d>
<d>41.74461</d>
<d>41.79482</d>
<d>41.84503</d>
<d>41.89524</d>
<d>41.94545</d>
<d>41.99566</d>
<d>42.04587</d>
<d>42.09608</d>
<d>42.14629</d>
<d>42.1965</d>
<d>42.24671</d>
<d>42.29692</d>
<d>42.34713</d>
<d>42.39734</d>
<d>42.44756</d>
<d>42.49777</d>
<d>42.54798</d>
<d>42.59819</d>
<d>42.6484</d>
<d>42.69861</d>
<d>42.74882</d>
<d>42.79903</d>
<d>42.84924</d>
<d>42.89945</d>
<d>42.94966</d>
<d>42.99987</d>
<d>43.05008</d>
<d>43.10029</d>
<d>43.1505</d>
<d>43.20071</d>
<d>43.25092</d>
<d>43.30113</d>
<d>43.35134</d>
<d>43.40155</d>
<d>43.45176</d>
<d>43.50197</d>
<d>43.55218</d>
<d>43.60239</d>
<d>43.6526</d>
<d>43.70281</d>
<d>43.75302</d>
<d>43.80323</d>
<d>43.85344</d>
<d>43.90365</d>
<d>43.95386</d>
<d>44.00407</d>
<d>44.05428</d>
<d>44.10449</d>
<d>44.1547</d>
<d>44.20491</d>
<d>44.25512</d>
<d>44.30533</d>
<d>44.35554</d>
<d>44.40575</d>
<d>44.45596</d>
<d>44.50617</d>
<d>44.55638</d>
<d>44.60659</d>
<d>44.6568</d>
<d>44.70701</d>
<d>44.75722</d>
<d>44.80743</d>
<d>44.85764</d>
<d>44.90785</d>
<d>44.95806</d>
<d>45.00827</d>
<d>45.05849</d>
<d>45.1087</d>
<d>45.15891</d>
<d>45.20912</d>
<d>45.25933</d>
<d>45.30954</d>
<d>45.35975</d>
<d>45.40996</d>
<d>45.46017</d>
<d>45.51038</d>
<d>45.56059</d>
<d>45.6108</d>
<d>45.66101</d>
<d>45.71122</d>
<d>45.76143</d>
<d>45.81164</d>
<d>45.86185</d>
<d>45.91206</d>
<d>45.96227</d>
<d>46.01248</d>
<d>46.06269</d>
<d>46.1129</d>
<d>46.16311</d>
<d>46.21332</d>
<d>46.26353</d>
<d>46.31374</d>
<d>46.36395</d>
<d>46.41416</d>
<d>46.46437</d>
<d>46.51458</d>
<d>46.56479</d>
<d>46.615</d>
<d>46.66521</d>
<d>46.71542</d>
<d>46.76563</d>
<d>46.81584</d>
<d>46.86605</d>
<d>46.91626</d>
<d>46.96647</d>
<d>47.01668</d>
<d>47.06689</d>
<d>47.1171</d>
<d>47.16731</d>
<d>47.21752</d>
<d>47.26773</d>
<d>47.31794</d>
<d>47.36815</d>
<d>47.41836</d>
<d>47.46857</d>
<d>47.51878</d>
<d>47.56899</d>
<d>47.61921</d>
<d>47.66942</d>
<d>47.71963</d>
<d>47.76984</d>
<d>47.82005</d>
<d>47.87026</d>
<d>47.92047</d>
<d>47.97068</d>
<d>48.02089</d>
<d>48.0711</d>
<d>48.12131</d>
<d>48.17152</d>
<d>48.22173</d>
<d>48.27194</d>
<d>48.32215</d>
<d>48.37236</d>
<d>48.42257</d>
<d>48.47278</d>
<d>48.52299</d>
<d>48.5732</d>
<d>48.62341</d>
<d>48.67362</d>
<d>48.72383</d>
<d>48.77404</d>
<d>48.82425</d>
<d>48.87446</d>
<d>48.92467</d>
<d>48.97488</d>
<d>49.02509</d>
<d>49.0753</d>
<d>49.12551</d>
<d>49.17572</d>
<d>49.22593</d>
<d>49.27614</d>
<d>49.32635</d>
<d>49.37656</d>
<d>49.42677</d>
<d>49.47698</d>
<d>49.52719</d>
<d>49.5774</d>
<d>49.62761</d>
<d>49.67782</d>
<d>49.72803</d>
<d>49.77824</d>
<d>49.82845</d>
<d>49.87866</d>
<d>49.92887</d>
<d>49.97908</d>
<d>50.02929</d>
<d>50.0795</d>
<d>50.12971</d>
<d>50.17992</d>
<d>50.23014</d>
<d>50.28035</d>
<d>50.33056</d>
<d>50.38077</d>
<d>50.43098</d>
<d>50.48119</d>
<d>50.5314</d>
<d>50.58161</d>
<d>50.63182</d>
<d>50.68203</d>
<d>50.73224</d>
<d>50.78245</d>
<d>50.83266</d>
<d>50.88287</d>
<d>50.93308</d>
<d>50.98329</d>
<d>51.0335</d>
<d>51.08371</d>
<d>51.13392</d>
<d>51.18413</d>
<d>51.23434</d>
<d>51.28455</d>
<d>51.33476</d>
<d>51.38497</d>
<d>51.43518</d>
<d>51.48539</d>
<d>51.5356</d>
<d>51.58581</d>
<d>51.63602</d>
<d>51.68623</d>
<d>51.73644</d>
<d>51.78665</d>
<d>51.83686</d>
<d>51.88707</d>
<d>51.93728</d>
<d>51.98749</d>
<d>52.0377</d>
<d>52.08791</d>
<d>52.13812</d>
<d>52.18833</d>
<d>52.23854</d>
<d>52.28875</d>
<d>52.33896</d>
<d>52.38917</d>
<d>52.43938</d>
<d>52.48959</d>
<d>52.5398</d>
<d>52.59001</d>
<d>52.64022</d>
<d>52.69043</d>
<d>52.74064</d>
<d>52.79085</d>
<d>52.84107</d>
<d>52.89128</d>
<d>52.94149</d>
<d>52.9917</d>
<d>53.04191</d>
<d>53.09212</d>
<d>53.14233</d>
<d>53.19254</d>
<d>53.24275</d>
<d>53.29296</d>
<d>53.34317</d>
<d>53.39338</d>
<d>53.44359</d>
<d>53.4938</d>
<d>53.54401</d>
<d>53.59422</d>
<d>53.64443</d>
<d>53.69464</d>
<d>53.74485</d>
<d>53.79506</d>
<d>53.84527</d>
<d>53.89548</d>
<d>53.94569</d>
<d>53.9959</d>
<d>54.04611</d>
<d>54.09632</d>
<d>54.14653</d>
<d>54.19674</d>
<d>54.24695</d>
<d>54.29716</d>
<d>54.34737</d>
<d>54.39758</d>
<d>54.44779</d>
<d>54.498</d>
<d>54.54821</d>
<d>54.59842</d>
<d>54.64863</d>
<d>54.69884</d>
<d>54.74905</d>
<d>54.79926</d>
<d>54.84947</d>
<d>54.89968</d>
<d>54.94989</d>
<d>55.0001</d>
<d>55.05031</d>
<d>55.10052</d>
<d>55.15073</d>
<d>55.20094</d>
<d>55.25115</d>
<d>55.30136</d>
<d>55.35157</d>
<d>55.40178</d>
<d>55.452</d>
<d>55.50221</d>
<d>55.55242</d>
<d>55.60263</d>
<d>55.65284</d>
<d>55.70305</d>
<d>55.75326</d>
<d>55.80347</d>
<d>55.85368</d>
<d>55.90389</d>
<d>55.9541</d>
<d>56.00431</d>
<d>56.05452</d>
<d>56.10473</d>
<d>56.15494</d>
<d>56.20515</d>
<d>56.25536</d>
<d>56.30557</d>
<d>56.35578</d>
<d>56.40599</d>
<d>56.4562</d>
<d>56.50641</d>
<d>56.55662</d>
<d>56.60683</d>
<d>56.65704</d>
<d>56.70725</d>
<d>56.75746</d>
<d>56.80767</d>
<d>56.85788</d>
<d>56.90809</d>
<d>56.9583</d>
<d>57.00851</d>
<d>57.05872</d>
<d>57.10893</d>
<d>57.15914</d>
<d>57.20935</d>
<d>57.25956</d>
<d>57.30977</d>
<d>57.35998</d>
<d>57.41019</d>
<d>57.4604</d>
<d>57.51061</d>
<d>57.56082</d>
<d>57.61103</d>
<d>57.66124</d>
<d>57.71145</d>
<d>57.76166</d>
<d>57.81187</d>
<d>57.86208</d>
<d>57.91229</d>
<d>57.9625</d>
<d>58.01271</d>
<d>58.06293</d>
<d>58.11314</d>
<d>58.16335</d>
<d>58.21356</d>
<d>58.26377</d>
<d>58.31398</d>
<d>58.36419</d>
<d>58.4144</d>
<d>58.46461</d>
<d>58.51482</d>
<d>58.56503</d>
<d>58.61524</d>
<d>58.66545</d>
<d>58.71566</d>
<d>58.76587</d>
<d>58.81608</d>
<d>58.86629</d>
<d>58.9165</d>
<d>58.96671</d>
<d>59.01692</d>
<d>59.06713</d>
<d>59.11734</d>
<d>59.16755</d>
<d>59.21776</d>
<d>59.26797</d>
<d>59.31818</d>
<d>59.36839</d>
<d>59.4186</d>
<d>59.46881</d>
<d>59.51902</d>
<d>59.56923</d>
<d>59.61944</d>
<d>59.66965</d>
<d>59.71986</d>
<d>59.77007</d>
<d>59.82028</d>
<d>59.87049</d>
<d>59.9207</d>
<d>59.97091</d>
<d>60.02112</d>
<d>60.07133</d>
<d>60.12154</d>
<d>60.17175</d>
<d>60.22196</d>
<d>60.27217</d>
<d>60.32238</d>
<d>60.37259</d>
<d>60.4228</d>
<d>60.47301</d>
<d>60.52322</d>
<d>60.57343</d>
<d>60.62364</d>
<d>60.67386</d>
<d>60.72407</d>
<d>60.77428</d>
<d>60.82449</d>
<d>60.8747</d>
<d>60.92491</d>
<d>60.97512</d>
<d>61.02533</d>
<d>61.07554</d>
<d>61.12575</d>
<d>61.17596</d>
<d>61.22617</d>
<d>61.27638</d>
<d>61.32659</d>
<d>61.3768</d>
<d>61.42701</d>
<d>61.47722</d>
<d>61.52743</d>
<d>61.57764</d>
<d>61.62785</d>
<d>61.67806</d>
<d>61.72827</d>
<d>61.77848</d>
<d>61.82869</d>
<d>61.8789</d>
<d>61.92911</d>
<d>61.97932</d>
<d>62.02953</d>
<d>62.07974</d>
<d>62.12995</d>
<d>62.18016</d>
<d>62.23037</d>
<d>62.28058</d>
<d>62.33079</d>
<d>62.381</d>
<d>62.43121</d>
<d>62.48142</d>
<d>62.53163</d>
<d>62.58184</d>
<d>62.63205</d>
<d>62.68226</d>
<d>62.73247</d>
<d>62.78268</d>
<d>62.83289</d>
<d>62.8831</d>
<d>62.93331</d>
<d>62.98352</d>
<d>63.03373</d>
<d>63.08394</d>
<d>63.13415</d>
<d>63.18436</d>
<d>63.23457</d>
<d>63.28479</d>
<d>63.335</d>
<d>63.38521</d>
<d>63.43542</d>
<d>63.48563</d>
<d>63.53584</d>
<d>63.58605</d>
<d>63.63626</d>
<d>63.68647</d>
<d>63.73668</d>
<d>63.78689</d>
<d>63.8371</d>
<d>63.88731</d>
<d>63.93752</d>
<d>63.98773</d>
<d>64.03794</d>
<d>64.08815</d>
<d>64.13836</d>
<d>64.18857</d>
<d>64.23878</d>
<d>64.28899</d>
<d>64.3392</d>
<d>64.38941</d>
<d>64.43962</d>
<d>64.48983</d>
<d>64.54004</d>
<d>64.59025</d>
<d>64.64046</d>
<d>64.69067</d>
<d>64.74088</d>
<d>64.79109</d>
<d>64.8413</d>
<d>64.89151</d>
<d>64.94172</d>
<d>64.99193</d>
<d>65.04214</d>
<d>65.09235</d>
<d>65.14256</d>
<d>65.19277</d>
<d>65.24298</d>
<d>65.29319</d>
<d>65.3434</d>
<d>65.39361</d>
<d>65.44382</d>
<d>65.49403</d>
<d>65.54424</d>
<d>65.59445</d>
<d>65.64466</d>
<d>65.69487</d>
<d>65.74508</d>
<d>65.79529</d>
<d>65.8455</d>
<d>65.89572</d>
<d>65.94593</d>
<d>65.99614</d>
<d>66.04635</d>
<d>66.09656</d>
<d>66.14677</d>
<d>66.19698</d>
<d>66.24719</d>
<d>66.2974</d>
<d>66.34761</d>
<d>66.39782</d>
<d>66.44803</d>
<d>66.49824</d>
<d>66.54845</d>
<d>66.59866</d>
<d>66.64887</d>
<d>66.69908</d>
<d>66.74929</d>
<d>66.7995</d>
<d>66.84971</d>
<d>66.89992</d>
<d>66.95013</d>
<d>67.00034</d>
<d>67.05055</d>
<d>67.10076</d>
<d>67.15097</d>
<d>67.20118</d>
<d>67.25139</d>
<d>67.3016</d>
<d>67.35181</d>
<d>67.40202</d>
<d>67.45223</d>
<d>67.50244</d>
<d>67.55265</d>
<d>67.60286</d>
<d>67.65307</d>
<d>67.70328</d>
<d>67.75349</d>
<d>67.8037</d>
<d>67.85391</d>
<d>67.90412</d>
<d>67.95433</d>
<d>68.00454</d>
<d>68.05475</d>
<d>68.10496</d>
<d>68.15517</d>
<d>68.20538</d>
<d>68.25559</d>
<d>68.3058</d>
<d>68.35601</d>
<d>68.40622</d>
<d>68.45644</d>
<d>68.50665</d>
<d>68.55686</d>
<d>68.60707</d>
<d>68.65728</d>
<d>68.70749</d>
<d>68.7577</d>
<d>68.80791</d>
<d>68.85812</d>
<d>68.90833</d>
<d>68.95854</d>
<d>69.00875</d>
<d>69.05896</d>
<d>69.10917</d>
<d>69.15938</d>
<d>69.20959</d>
<d>69.2598</d>
<d>69.31001</d>
<d>69.36022</d>
<d>69.41043</d>
<d>69.46064</d>
<d>69.51085</d>
<d>69.56106</d>
<d>69.61127</d>
<d>69.66148</d>
<d>69.71169</d>
<d>69.7619</d>
<d>69.81211</d>
<d>69.86232</d>
<d>69.91253</d>
<d>69.96274</d>
<d>70.01295</d>
<d>70.06316</d>
<d>70.11337</d>
<d>70.16358</d>
<d>70.21379</d>
<d>70.264</d>
<d>70.31421</d>
<d>70.36442</d>
<d>70.41463</d>
<d>70.46484</d>
<d>70.51505</d>
<d>70.56526</d>
<d>70.61547</d>
<d>70.66568</d>
<d>70.71589</d>
<d>70.7661</d>
<d>70.81631</d>
<d>70.86652</d>
<d>70.91673</d>
<d>70.96694</d>
<d>71.01715</d>
<d>71.06737</d>
<d>71.11758</d>
<d>71.16779</d>
<d>71.218</d>
<d>71.26821</d>
<d>71.31842</d>
<d>71.36863</d>
<d>71.41884</d>
<d>71.46905</d>
<d>71.51926</d>
<d>71.56947</d>
<d>71.61968</d>
<d>71.66989</d>
<d>71.7201</d>
<d>71.77031</d>
<d>71.82052</d>
<d>71.87073</d>
<d>71.92094</d>
<d>71.97115</d>
<d>72.02136</d>
<d>72.07157</d>
<d>72.12178</d>
<d>72.17199</d>
<d>72.2222</d>
<d>72.27241</d>
<d>72.32262</d>
<d>72.37283</d>
<d>72.42304</d>
<d>72.47325</d>
<d>72.52346</d>
<d>72.57367</d>
<d>72.62388</d>
<d>72.67409</d>
<d>72.7243</d>
<d>72.77451</d>
<d>72.82472</d>
<d>72.87493</d>
<d>72.92514</d>
<d>72.97535</d>
<d>73.02556</d>
<d>73.07577</d>
<d>73.12598</d>
<d>73.17619</d>
<d>73.2264</d>
<d>73.27661</d>
<d>73.32682</d>
<d>73.37703</d>
<d>73.42724</d>
<d>73.47745</d>
<d>73.52766</d>
<d>73.57787</d>
<d>73.62808</d>
<d>73.6783</d>
<d>73.72851</d>
<d>73.77872</d>
<d>73.82893</d>
<d>73.87914</d>
<d>73.92935</d>
<d>73.97956</d>
<d>74.02977</d>
<d>74.07998</d>
<d>74.13019</d>
<d>74.1804</d>
<d>74.23061</d>
<d>74.28082</d>
<d>74.33103</d>
<d>74.38124</d>
<d>74.43145</d>
<d>74.48166</d>
<d>74.53187</d>
<d>74.58208</d>
<d>74.63229</d>
<d>74.6825</d>
<d>74.73271</d>
<d>74.78292</d>
<d>74.83313</d>
<d>74.88334</d>
<d>74.93355</d>
<d>74.98376</d>
<d>75.03397</d>
<d>75.08418</d>
<d>75.13439</d>
<d>75.1846</d>
<d>75.23481</d>
<d>75.28502</d>
<d>75.33523</d>
<d>75.38544</d>
<d>75.43565</d>
<d>75.48586</d>
<d>75.53607</d>
<d>75.58628</d>
<d>75.63649</d>
<d>75.6867</d>
<d>75.73691</d>
<d>75.78712</d>
<d>75.83733</d>
<d>75.88754</d>
<d>75.93775</d>
<d>75.98796</d>
<d>76.03817</d>
<d>76.08838</d>
<d>76.13859</d>
<d>76.1888</d>
<d>76.23901</d>
<d>76.28923</d>
<d>76.33944</d>
<d>76.38965</d>
<d>76.43986</d>
<d>76.49007</d>
<d>76.54028</d>
<d>76.59049</d>
<d>76.6407</d>
<d>76.69091</d>
<d>76.74112</d>
<d>76.79133</d>
<d>76.84154</d>
<d>76.89175</d>
<d>76.94196</d>
<d>76.99217</d>
<d>77.04238</d>
<d>77.09259</d>
<d>77.1428</d>
<d>77.19301</d>
<d>77.24322</d>
<d>77.29343</d>
<d>77.34364</d>
<d>77.39385</d>
<d>77.44406</d>
<d>77.49427</d>
<d>77.54448</d>
<d>77.59469</d>
<d>77.6449</d>
<d>77.69511</d>
<d>77.74532</d>
<d>77.79553</d>
<d>77.84574</d>
<d>77.89595</d>
<d>77.94616</d>
<d>77.99637</d>
<d>78.04658</d>
<d>78.09679</d>
<d>78.147</d>
<d>78.19721</d>
<d>78.24742</d>
<d>78.29763</d>
<d>78.34784</d>
<d>78.39805</d>
<d>78.44826</d>
<d>78.49847</d>
<d>78.54868</d>
<d>78.59889</d>
<d>78.6491</d>
<d>78.69931</d>
<d>78.74952</d>
<d>78.79973</d>
<d>78.84994</d>
<d>78.90016</d>
<d>78.95037</d>
<d>79.00058</d>
<d>79.05079</d>
<d>79.101</d>
<d>79.15121</d>
</Subcolumn>
</XAdvancedColumn>
<YColumn Width="372" Decimals="9" Subcolumns="3">
<Title>Caucasian propolis</Title>
<Subcolumn>
<d>0</d>
<d>5.779227073</d>
<d>7.533234657</d>
<d>5.440355046</d>
<d>5.278377822</d>
<d>5.502110404</d>
<d>3.704965604</d>
<d>2.997083023</d>
<d>0.725243628</d>
<d>-0.682789469</d>
<d>0.983672262</d>
<d>2.095671116</d>
<d>1.856052891</d>
<d>1.910602292</d>
<d>4.401291845</d>
<d>4.944866091</d>
<d>6.177300061</d>
<d>5.139313569</d>
<d>4.255634569</d>
<d>2.968036588</d>
<d>4.025888203</d>
<d>3.637911398</d>
<d>2.735293005</d>
<d>4.179581084</d>
<d>2.788787692</d>
<d>0.154671698</d>
<d>1.85852659</d>
<d>2.195224387</d>
<d>0.467771139</d>
<d>-1.6218373</d>
<d>-0.331211744</d>
<d>-1.735200494</d>
<d>-0.894421248</d>
<d>1.571989246</d>
<d>0.759699405</d>
<d>1.981451829</d>
<d>3.753417907</d>
<d>3.86053848</d>
<d>2.159918254</d>
<d>2.106782423</d>
<d>1.776840076</d>
<d>-0.792078483</d>
<d>0.203736693</d>
<d>0.774750993</d>
<d>-0.859945576</d>
<d>-0.866289058</d>
<d>-1.381748505</d>
<d>0.151038859</d>
<d>1.373268737</d>
<d>1.943425021</d>
<d>0.677310009</d>
<d>0.504823548</d>
<d>1.800868498</d>
<d>-0.050546607</d>
<d>-1.205089167</d>
<d>0.402363112</d>
<d>1.673519756</d>
<d>2.509555983</d>
<d>2.699831745</d>
<d>2.482060709</d>
<d>0.289736527</d>
<d>-0.476184254</d>
<d>-0.763035512</d>
<d>-0.868141439</d>
<d>0.512159528</d>
<d>2.440003209</d>
<d>2.118672543</d>
<d>0.39182037</d>
<d>-0.279646257</d>
<d>1.062411705</d>
<d>0.028789886</d>
<d>-1.473946134</d>
<d>-1.606910387</d>
<d>-0.280715801</d>
<d>1.044319354</d>
<d>0.769915268</d>
<d>0.099726427</d>
<d>-1.150906253</d>
<d>-0.211853705</d>
<d>1.555918701</d>
<d>1.393087066</d>
<d>0.005508005</d>
<d>0.186499199</d>
<d>0.19387929</d>
<d>1.869989889</d>
<d>2.595731451</d>
<d>3.051343847</d>
<d>1.620544119</d>
<d>3.371076821</d>
<d>3.147663409</d>
<d>3.236992797</d>
<d>1.453750387</d>
<d>-0.599014156</d>
<d>-1.617882082</d>
<d>-1.212410516</d>
<d>1.285499779</d>
<d>1.714766994</d>
<d>1.470957312</d>
<d>3.188710295</d>
<d>4.08976265</d>
<d>2.89327721</d>
<d>2.34668005</d>
<d>0.460485041</d>
<d>-0.920659483</d>
<d>-1.462278982</d>
<d>-0.419003723</d>
<d>-0.31520735</d>
<d>-0.208286142</d>
<d>0.170099701</d>
<d>2.231542004</d>
<d>0.295801094</d>
<d>1.022501803</d>
<d>-0.198155609</d>
<d>-0.982599945</d>
<d>0.255950006</d>
<d>1.443266469</d>
<d>2.016995156</d>
<d>1.011591233</d>
<d>1.535907529</d>
<d>3.457246885</d>
<d>2.788017191</d>
<d>2.602470461</d>
<d>2.899514039</d>
<d>2.161568341</d>
<d>3.033592471</d>
<d>3.101906453</d>
<d>-0.414423943</d>
<d>-2.437456031</d>
<d>-1.330874801</d>
<d>-1.032530796</d>
<d>-0.094056713</d>
<d>0</d>
<d>1.008417615</d>
<d>0.90952206</d>
<d>2.703386223</d>
<d>3.622066518</d>
<d>3.277426956</d>
<d>3.931268312</d>
<d>5.524587259</d>
<d>4.72708592</d>
<d>4.573195189</d>
<d>2.64522885</d>
<d>0.88386784</d>
<d>-0.096450051</d>
<d>-0.280063234</d>
<d>0.000739235</d>
<d>1.519680299</d>
<d>2.008403576</d>
<d>1.565855425</d>
<d>0.660664591</d>
<d>0.480913949</d>
<d>0.891721197</d>
<d>-1.210424442</d>
<d>-0.623988425</d>
<d>1.14e-013</d>
<d>1.277816277</d>
<d>72.91203815</d>
<d>63.2597255</d>
<d>22.92183473</d>
<d>28.27288277</d>
<d>19.9189382</d>
<d>32.84912722</d>
<d>-10.75402393</d>
<d>15.953619</d>
<d>-4.513714549</d>
<d>-1.78e-012</d>
<d>9.027416391</d>
<d>13.56276631</d>
<d>46.65752045</d>
<d>83.7395991</d>
<d>48.25579619</d>
<d>62.0286458</d>
<d>98.18262084</d>
<d>51.66301143</d>
<d>37.62573424</d>
<d>74.58590772</d>
<d>83.87340762</d>
<d>135.3828961</d>
<d>186.8496672</d>
<d>182.9423396</d>
<d>170.1215926</d>
<d>155.0008559</d>
<d>171.9678301</d>
<d>84.25286998</d>
<d>103.5574027</d>
<d>110.5263709</d>
<d>84.3872214</d>
<d>90.28300549</d>
<d>115.74488</d>
<d>70.03725082</d>
<d>75.66778481</d>
<d>36.21275703</d>
<d>-18.86307165</d>
<d>-31.31934892</d>
<d>-0.535465629</d>
<d>40.54779607</d>
<d>34.06268095</d>
<d>43.27279468</d>
<d>17.06891791</d>
<d>15.69404139</d>
<d>10.44112062</d>
<d>-45.24933566</d>
<d>-56.2480131</d>
<d>-11.57842998</d>
<d>8.710879675</d>
<d>6.752031325</d>
<d>38.63805522</d>
<d>77.23813107</d>
<d>44.46289005</d>
<d>78.22667725</d>
<d>47.58100806</d>
<d>-54.12559347</d>
<d>-17.01566361</d>
<d>-19.33193548</d>
<d>-4.765097569</d>
<d>-10.55663058</d>
<d>28.59971966</d>
<d>44.83847241</d>
<d>79.04211467</d>
<d>61.84626172</d>
<d>39.32105082</d>
<d>-12.48143825</d>
<d>42.57657115</d>
<d>39.45229274</d>
<d>55.04947299</d>
<d>13.22967339</d>
<d>-52.90258072</d>
<d>-73.94012358</d>
<d>-39.5422983</d>
<d>-53.31723904</d>
<d>-58.72646622</d>
<d>-85.0176662</d>
<d>-50.74878607</d>
<d>43.1131395</d>
<d>135.4266416</d>
<d>168.267423</d>
<d>210.6390758</d>
<d>301.0334607</d>
<d>413.5533189</d>
<d>466.1132044</d>
<d>437.4150815</d>
<d>326.6251716</d>
<d>267.1619112</d>
<d>201.3749555</d>
<d>80.78148034</d>
<d>15.19105125</d>
<d>2.657382102</d>
<d>12.11586108</d>
<d>46.28782165</d>
<d>102.4577003</d>
<d>62.78081086</d>
<d>19.29382116</d>
<d>6.074748052</d>
<d>-36.70179637</d>
<d>-64.64013713</d>
<d>-22.52430971</d>
<d>23.94335087</d>
<d>-7.049319091</d>
<d>-24.5569585</d>
<d>11.27060898</d>
<d>-47.50320378</d>
<d>-50.36045031</d>
<d>-8.837256945</d>
<d>24.42195261</d>
<d>44.57102113</d>
<d>77.26016606</d>
<d>68.35906083</d>
<d>64.41199442</d>
<d>19.92168988</d>
<d>-5.215349891</d>
<d>-23.159651</d>
<d>-83.54702943</d>
<d>-104.4062357</d>
<d>-64.02421518</d>
<d>14.2259967</d>
<d>238.0720587</d>
<d>621.8218198</d>
<d>1110.547985</d>
<d>1581.30651</d>
<d>2069.366395</d>
<d>2401.527655</d>
<d>2390.693566</d>
<d>2195.470502</d>
<d>1795.433921</d>
<d>1286.118252</d>
<d>823.4908033</d>
<d>450.4934841</d>
<d>132.000103</d>
<d>-11.12649352</d>
<d>-16.38063967</d>
<d>-39.02037923</d>
<d>-35.85186019</d>
<d>-28.82419615</d>
<d>-81.10817737</d>
<d>-57.32370851</d>
<d>-49.71415062</d>
<d>-68.32698614</d>
<d>-55.14272277</d>
<d>18.29441887</d>
<d>58.80448094</d>
<d>35.49880068</d>
<d>70.80883355</d>
<d>87.4463071</d>
<d>94.9370868</d>
<d>79.40354255</d>
<d>53.98847826</d>
<d>71.85906411</d>
<d>112.4230446</d>
<d>137.1193254</d>
<d>109.315386</d>
<d>46.80655194</d>
<d>50.52823897</d>
<d>8.579973659</d>
<d>-41.38021519</d>
<d>-96.11613118</d>
<d>-75.95234243</d>
<d>-27.6197058</d>
<d>19.31424671</d>
<d>99.64064406</d>
<d>124.0782036</d>
<d>118.8021261</d>
<d>129.6827941</d>
<d>131.9498778</d>
<d>172.4954331</d>
<d>292.524403</d>
<d>383.0621082</d>
<d>512.8435277</d>
<d>701.0934166</d>
<d>801.9289525</d>
<d>762.0228179</d>
<d>690.8631816</d>
<d>593.3705705</d>
<d>464.75684</d>
<d>296.5902085</d>
<d>208.1053537</d>
<d>93.79938581</d>
<d>79.93517069</d>
<d>69.22207813</d>
<d>19.54106805</d>
<d>-6.956321648</d>
<d>8.074563869</d>
<d>11.15735602</d>
<d>-18.45677882</d>
<d>-28.07939767</d>
<d>-9.092875202</d>
<d>-2.609659005</d>
<d>4.632720035</d>
<d>-6.672595114</d>
<d>3.923169328</d>
<d>14.83638861</d>
<d>26.86276879</d>
<d>21.84525828</d>
<d>-12.98205261</d>
<d>8.772342764</d>
<d>23.40081167</d>
<d>33.60271166</d>
<d>60.6989224</d>
<d>74.63076063</d>
<d>67.75694699</d>
<d>30.7571811</d>
<d>8.313957569</d>
<d>18.0893369</d>
<d>6.240409559</d>
<d>30.62674789</d>
<d>82.3620838</d>
<d>107.5920856</d>
<d>162.6381748</d>
<d>170.531879</d>
<d>22.94668919</d>
<d>17.06838304</d>
<d>17.21395475</d>
<d>25.01918121</d>
<d>16.57691106</d>
<d>17.50553483</d>
<d>13.43111751</d>
<d>15.53353037</d>
<d>11.371045</d>
<d>12.00999339</d>
<d>6.825954712</d>
<d>11.68926231</d>
<d>19.32932192</d>
<d>34.7581728</d>
<d>37.00844576</d>
<d>48.57654219</d>
<d>38.61378012</d>
<d>39.61277266</d>
<d>24.70482988</d>
<d>26.89927034</d>
<d>17.33646172</d>
<d>18.90349655</d>
<d>15.07526977</d>
<d>26.14115338</d>
<d>28.60234587</d>
<d>28.3543742</d>
<d>12.6096933</d>
<d>1.460821175</d>
<d>-6.226879733</d>
<d>-1.679612175</d>
<d>-6.833630525</d>
<d>-2.787162467</d>
<d>-9.751742214</d>
<d>1.121546427</d>
<d>-0.159631681</d>
<d>-1.021326983</d>
<d>-0.76570497</d>
<d>6.870486546</d>
<d>19.42425386</d>
<d>27.25715609</d>
<d>29.14825551</d>
<d>34.09511018</d>
<d>31.44683845</d>
<d>37.04388193</d>
<d>36.93110191</d>
<d>33.08757705</d>
<d>31.52402184</d>
<d>26.22004578</d>
<d>24.46515992</d>
<d>20.53628421</d>
<d>18.62187951</d>
<d>21.68212189</d>
<d>21.52178772</d>
<d>26.05007894</d>
<d>26.06142756</d>
<d>15.78117434</d>
<d>11.58362497</d>
<d>3.463204242</d>
<d>8.472000809</d>
<d>11.53047092</d>
<d>12.48638949</d>
<d>21.33396243</d>
<d>22.91536026</d>
<d>28.95363898</d>
<d>21.31235502</d>
<d>12.32364842</d>
<d>10.67955803</d>
<d>2.092275197</d>
<d>-5.477812139</d>
<d>-5.205766794</d>
<d>-10.09392822</d>
<d>0.926584633</d>
<d>3.582992243</d>
<d>18.09689776</d>
<d>21.46060479</d>
<d>25.59629509</d>
<d>32.76841545</d>
<d>35.09687786</d>
<d>36.03432161</d>
<d>43.23624642</d>
<d>33.65274465</d>
<d>34.74255307</d>
<d>27.61056553</d>
<d>21.38071126</d>
<d>12.59853993</d>
<d>5.701597748</d>
<d>8.927255841</d>
<d>18.52123849</d>
<d>17.68653537</d>
<d>8.549115086</d>
<d>9.115481637</d>
<d>9.201094963</d>
<d>9.802649856</d>
<d>13.93335216</d>
<d>16.65390204</d>
<d>14.71041125</d>
<d>17.18669484</d>
<d>19.88179738</d>
<d>15.81543157</d>
<d>7.452822295</d>
<d>6.403521713</d>
<d>-0.361611979</d>
<d>-0.324033504</d>
<d>5.20273189</d>
<d>2.203041725</d>
<d>4.540234088</d>
<d>4.127441944</d>
<d>11.77858402</d>
<d>21.38711131</d>
<d>20.24887297</d>
<d>15.72141236</d>
<d>11.18493364</d>
<d>2.803382511</d>
<d>-4.177567549</d>
<d>-8.928760539</d>
<d>-3.078540148</d>
<d>-2.779080661</d>
<d>2.686776989</d>
<d>13.87950017</d>
<d>22.41780483</d>
<d>31.63259997</d>
<d>31.435459</d>
<d>27.1900225</d>
<d>16.78183299</d>
<d>10.74347253</d>
<d>1.379368038</d>
<d>-10.12348926</d>
<d>-7.570546807</d>
<d>-0.412858896</d>
<d>-0.908352643</d>
<d>3.446706255</d>
<d>5.711957517</d>
<d>12.22496329</d>
<d>11.91636331</d>
<d>9.303535962</d>
<d>8.319719442</d>
<d>6.629516046</d>
<d>18.09563189</d>
<d>22.75347294</d>
<d>12.35614753</d>
<d>9.228732475</d>
<d>10.24176015</d>
<d>14.35298117</d>
<d>6.424284255</d>
<d>8.344825831</d>
<d>5.34859902</d>
<d>-1.198777152</d>
<d>-2.977906321</d>
<d>-4.589956901</d>
<d>-8.688799554</d>
<d>-2.146641319</d>
<d>4.727541816</d>
<d>14.11414364</d>
<d>16.52706194</d>
<d>28.35720316</d>
<d>24.14538181</d>
<d>22.26506716</d>
<d>14.61716846</d>
<d>8.453828821</d>
<d>10.08730318</d>
<d>10.05481311</d>
<d>12.65551425</d>
<d>16.60996849</d>
<d>13.65437418</d>
<d>10.27415117</d>
<d>5.840499554</d>
<d>1.239381455</d>
<d>-3.941804384</d>
<d>-7.648391364</d>
<d>-7.27841386</d>
<d>-3.57489532</d>
<d>11.87502537</d>
<d>21.16962136</d>
<d>17.60419025</d>
<d>14.45688268</d>
<d>9.606486604</d>
<d>7.147893073</d>
<d>0.643359532</d>
<d>-2.951306215</d>
<d>-2.190008654</d>
<d>0.126844401</d>
<d>8.887558893</d>
<d>11.37846012</d>
<d>4.937834885</d>
<d>2.352104607</d>
<d>-1.199049098</d>
<d>0.634412368</d>
<d>-2.041061496</d>
<d>-1.699927089</d>
<d>-3.220095055</d>
<d>0.85807701</d>
<d>4.185666355</d>
<d>0.75053258</d>
<d>0.051898308</d>
<d>1.086267075</d>
<d>1.004959896</d>
<d>7.994359507</d>
<d>10.75153318</d>
<d>13.87651958</d>
<d>15.98645163</d>
<d>24.99848131</d>
<d>23.08015011</d>
<d>22.70989578</d>
<d>29.14315711</d>
<d>26.09759208</d>
<d>24.88021269</d>
<d>27.75024572</d>
<d>26.46283913</d>
<d>21.01119417</d>
<d>17.13890035</d>
<d>15.28447747</d>
<d>6.423256106</d>
<d>6.951797388</d>
<d>7.496655685</d>
<d>4.813464382</d>
<d>9.11327591</d>
<d>10.77924916</d>
<d>13.6098494</d>
<d>8.073899859</d>
<d>2.253082418</d>
<d>2.697998995</d>
<d>3.662046805</d>
<d>4.733044086</d>
<d>2.781234001</d>
<d>3.908691392</d>
<d>5.549502295</d>
<d>2.056909038</d>
<d>6.455832016</d>
<d>-2.788133135</d>
<d>-5.541933559</d>
<d>-2.451242261</d>
<d>1.329224344</d>
<d>-0.761984867</d>
<d>5.411406049</d>
<d>4.48275634</d>
<d>2.811002543</d>
<d>-4.424173721</d>
<d>2.320253188</d>
<d>-0.797596122</d>
<d>1.098441099</d>
<d>-5.040137475</d>
<d>-2.364128208</d>
<d>-0.860224568</d>
<d>10.29791334</d>
<d>3.308810329</d>
<d>3.741909816</d>
<d>6.85779863</d>
<d>14.59888151</d>
<d>13.85103699</d>
<d>4.623783324</d>
<d>8.399151521</d>
<d>18.64146121</d>
<d>22.48667788</d>
<d>16.27392159</d>
<d>7.566040573</d>
<d>3.109778032</d>
<d>-1.79470303</d>
<d>-1.32767332</d>
<d>-3.343859693</d>
<d>-6.676131833</d>
<d>2.310126109</d>
<d>7.545022908</d>
<d>15.32788724</d>
<d>11.85136513</d>
<d>14.05068665</d>
<d>12.81705761</d>
<d>14.33138416</d>
<d>23.6738342</d>
<d>21.10093918</d>
<d>18.56909858</d>
<d>11.8815341</d>
<d>4.401674242</d>
<d>11.50702112</d>
<d>13.5247596</d>
<d>17.25438599</d>
<d>25.33523148</d>
<d>35.48568205</d>
<d>38.23783337</d>
<d>34.85629408</d>
<d>33.69337287</d>
<d>26.35807751</d>
<d>20.40861582</d>
<d>22.68320475</d>
<d>18.85620972</d>
<d>13.06265773</d>
<d>8.160087534</d>
<d>7.059066517</d>
<d>4.696578893</d>
<d>7.341774886</d>
<d>8.964173033</d>
<d>13.44723751</d>
<d>20.30759015</d>
<d>27.43762889</d>
<d>27.64971995</d>
<d>29.98214109</d>
<d>24.31087406</d>
<d>25.03578437</d>
<d>24.75719823</d>
<d>24.68937353</d>
<d>21.71032129</d>
<d>21.10675459</d>
<d>16.30155634</d>
<d>16.11543328</d>
<d>14.71437244</d>
<d>17.34429433</d>
<d>9.292689847</d>
<d>1.530990049</d>
<d>1.100390751</d>
<d>2.657456487</d>
<d>3.737030983</d>
<d>3.886632711</d>
<d>4.491386907</d>
<d>5.045029022</d>
<d>4.674525876</d>
<d>5.446942264</d>
<d>4.304416032</d>
<d>2.803255394</d>
<d>2.589730679</d>
<d>3.48270359</d>
<d>2.833572832</d>
<d>2.09265886</d>
<d>1.792935626</d>
<d>0.908883548</d>
<d>1.23343185</d>
<d>3.652847008</d>
<d>3.888040224</d>
<d>4.542813843</d>
<d>5.378118729</d>
<d>5.583931605</d>
<d>6.643959676</d>
<d>6.921766527</d>
<d>6.036621792</d>
<d>4.858766308</d>
<d>4.10704717</d>
<d>4.068833248</d>
<d>3.115458136</d>
<d>3.666307905</d>
<d>4.037476252</d>
<d>4.285659949</d>
<d>6.340127569</d>
<d>6.763986279</d>
<d>7.431871786</d>
<d>6.786261812</d>
<d>7.109231832</d>
<d>6.80538078</d>
<d>5.541083963</d>
<d>4.859294892</d>
<d>3.726845389</d>
<d>2.538184738</d>
<d>3.035858519</d>
<d>2.935484538</d>
<d>5.075907865</d>
<d>6.318531111</d>
<d>7.334245858</d>
<d>7.794647884</d>
<d>7.706293133</d>
<d>7.213533775</d>
<d>6.858085018</d>
<d>5.899610838</d>
<d>4.978529819</d>
<d>3.507842179</d>
<d>3.366190033</d>
<d>2.202539093</d>
<d>1.558993028</d>
<d>2.377179617</d>
<d>3.305603186</d>
<d>4.465548697</d>
<d>6.086231368</d>
<d>6.025678676</d>
<d>5.874314021</d>
<d>4.517626958</d>
<d>4.116043782</d>
<d>3.359711202</d>
<d>2.801271</d>
<d>1.577402503</d>
<d>1.288848653</d>
<d>1.311944947</d>
<d>1.907957885</d>
<d>1.945258724</d>
<d>1.820967689</d>
<d>1.605500472</d>
<d>3.313474321</d>
<d>3.727499115</d>
<d>3.801828896</d>
<d>4.046248996</d>
<d>4.973094283</d>
<d>3.704912462</d>
<d>3.125833177</d>
<d>2.414056734</d>
<d>1.414182983</d>
<d>0.836560575</d>
<d>0.920360052</d>
<d>0.019553896</d>
<d>0.199264403</d>
<d>0.685890204</d>
<d>0.727076047</d>
<d>0.974288493</d>
<d>1.472205087</d>
<d>1.478668708</d>
<d>2.271117147</d>
<d>1.89126571</d>
<d>2.766634698</d>
<d>2.6754876</d>
<d>3.544543457</d>
<d>4.161559695</d>
<d>3.988985596</d>
<d>3.667545998</d>
<d>3.04147634</d>
<d>2.528292919</d>
<d>1.986314309</d>
<d>0.465656896</d>
<d>0.197890902</d>
<d>-0.159374074</d>
<d>-0.210960518</d>
<d>0.65162007</d>
<d>-0.487813449</d>
<d>-0.867873483</d>
<d>0.575566552</d>
<d>1.544890948</d>
<d>1.131494232</d>
<d>1.119928361</d>
<d>0.511625738</d>
<d>-0.049838718</d>
<d>-0.57563463</d>
<d>0.221630874</d>
<d>-0.045377239</d>
<d>1.322811346</d>
<d>2.89882173</d>
<d>4.381080246</d>
<d>5.050860951</d>
<d>5.193104327</d>
<d>4.378760016</d>
<d>2.844084541</d>
<d>2.864232944</d>
<d>2.217347039</d>
<d>1.23389468</d>
<d>0.699738999</d>
<d>0.687602127</d>
<d>-0.016579888</d>
<d>-0.096398103</d>
<d>-0.384336269</d>
<d>-0.509862452</d>
<d>-0.12065568</d>
<d>0.776242174</d>
<d>0.615538513</d>
<d>0.197543791</d>
<d>0.845708492</d>
<d>1.383847413</d>
<d>0.81108614</d>
<d>0.84156083</d>
<d>1.328976474</d>
<d>0.248881568</d>
<d>1.185070301</d>
<d>1.442563794</d>
<d>1.585415673</d>
<d>0.993360447</d>
<d>1.400930509</d>
<d>1.723845229</d>
<d>0.754839319</d>
<d>0.885132193</d>
<d>0.88028172</d>
<d>-0.155673024</d>
<d>0.819104474</d>
<d>1.984247155</d>
<d>1.289201488</d>
<d>1.346256829</d>
<d>1.278316443</d>
<d>1.794352747</d>
<d>2.111979525</d>
<d>1.821039404</d>
<d>1.169029943</d>
<d>-0.016499581</d>
<d>0.378199397</d>
<d>1.140458023</d>
<d>0.425925309</d>
<d>1.661223802</d>
<d>2.1987669</d>
<d>3.579724056</d>
<d>3.444956706</d>
<d>1.055365001</d>
<d>0.221058358</d>
<d>-1.16848776</d>
<d>-0.557312159</d>
<d>-0.550693144</d>
<d>-0.117194798</d>
<d>1.405042663</d>
<d>2.472272508</d>
<d>3.085988971</d>
<d>2.36564721</d>
<d>1.055823209</d>
<d>0.928162534</d>
<d>1.459455254</d>
<d>1.469160206</d>
<d>0.874571149</d>
<d>1.506501824</d>
<d>1.530429415</d>
<d>1.464880515</d>
<d>1.518674072</d>
<d>1.537016971</d>
<d>1.714159397</d>
<d>2.159488486</d>
<d>2.751476467</d>
<d>2.566827921</d>
<d>2.825919644</d>
<d>2.518043636</d>
<d>1.95679254</d>
<d>1.895735675</d>
<d>1.740346063</d>
<d>0.641628982</d>
<d>1.479343449</d>
<d>0.756743292</d>
<d>0.856549863</d>
<d>1.414301248</d>
<d>2.118882246</d>
<d>1.899216644</d>
<d>2.466059098</d>
<d>2.376147838</d>
<d>1.902663889</d>
<d>1.152703963</d>
<d>1.239944565</d>
<d>0.662361656</d>
<d>0.864584748</d>
<d>1.782890299</d>
<d>2.823251412</d>
<d>2.583924295</d>
<d>2.390558854</d>
<d>2.356197887</d>
<d>1.477916565</d>
<d>1.087676998</d>
<d>0.553814434</d>
<d>0.787010317</d>
<d>0.465646955</d>
<d>0.809870079</d>
<d>0.564227939</d>
<d>-0.634516082</d>
<d>-1.127575809</d>
<d>-0.630606782</d>
<d>0.36094213</d>
<d>1.257896121</d>
<d>1.110017626</d>
<d>1.927684524</d>
<d>1.55169423</d>
<d>1.133716978</d>
<d>0.099024273</d>
<d>0.384056711</d>
<d>0.042921912</d>
<d>0.148375209</d>
<d>1.449623793</d>
<d>1.34870115</d>
<d>1.398160056</d>
<d>2.437412121</d>
<d>1.041348762</d>
<d>0.341290663</d>
<d>-0.400662553</d>
<d>0.51822078</d>
<d>0.142916925</d>
<d>0.319052552</d>
<d>1.086820116</d>
<d>1.025650532</d>
<d>2.316858669</d>
<d>3.311145587</d>
<d>2.279933864</d>
<d>1.956788772</d>
<d>0.989704312</d>
<d>1.321182348</d>
<d>1.114830181</d>
<d>1.377349401</d>
<d>0.911187609</d>
<d>0.721103895</d>
<d>1.67345409</d>
<d>1.936701749</d>
<d>1.565843955</d>
<d>1.721039947</d>
<d>1.398259074</d>
<d>2.198972718</d>
<d>2.042518567</d>
<d>2.271408224</d>
<d>2.005809749</d>
<d>2.287511176</d>
<d>1.981970007</d>
<d>1.343775393</d>
<d>1.1750592</d>
<d>1.505201766</d>
<d>1.436888638</d>
<d>0.530628767</d>
<d>-0.056089547</d>
<d>-0.133386747</d>
<d>0.978734227</d>
<d>1.761358322</d>
<d>2.244594316</d>
<d>2.760731437</d>
<d>2.57174038</d>
<d>3.40596218</d>
<d>3.197060479</d>
<d>2.102742146</d>
<d>2.179509626</d>
<d>1.421365522</d>
<d>2.258501076</d>
<d>1.328202744</d>
<d>0.350070305</d>
<d>0.08714277</d>
<d>-0.183311959</d>
<d>0.212511452</d>
<d>0.794450823</d>
<d>2.716352262</d>
<d>3.57819262</d>
<d>3.532966962</d>
<d>3.774952327</d>
<d>3.806385165</d>
<d>3.139335789</d>
<d>3.151404685</d>
<d>2.753579139</d>
<d>1.678646082</d>
<d>1.930298481</d>
<d>2.0939116</d>
<d>2.226912849</d>
<d>1.937404631</d>
<d>1.409441108</d>
<d>2.539270385</d>
<d>2.6269383</d>
<d>2.032659527</d>
<d>1.411301949</d>
<d>0.980670494</d>
<d>0.989528672</d>
<d>-0.592278948</d>
<d>-0.104227216</d>
<d>0.319604515</d>
<d>0.795161049</d>
<d>2.123847502</d>
<d>2.443413273</d>
<d>1.574785597</d>
<d>2.079033372</d>
<d>1.827661741</d>
<d>1.782089256</d>
<d>0.419153147</d>
<d>0.64253096</d>
<d>1.099150187</d>
<d>1.826526818</d>
<d>2.145029409</d>
<d>2.08331123</d>
<d>1.379500095</d>
<d>1.461700413</d>
<d>2.151081902</d>
<d>2.548995909</d>
<d>2.01830307</d>
<d>1.930046711</d>
<d>1.853963873</d>
<d>2.214006772</d>
<d>0.394597809</d>
<d>-0.084569902</d>
<d>-1.153856467</d>
<d>-1.276377589</d>
<d>1.71120786</d>
<d>1.732269485</d>
<d>1.91605743</d>
<d>2.20781845</d>
<d>2.582179626</d>
<d>1.93118745</d>
<d>0.337884851</d>
<d>0.048533383</d>
<d>-1.019470821</d>
<d>-0.686639743</d>
<d>0.207381099</d>
<d>0.793080204</d>
<d>0.843549955</d>
<d>1.333198717</d>
<d>2.728106385</d>
<d>2.378337034</d>
<d>3.520706631</d>
<d>4.008994237</d>
<d>4.060760861</d>
<d>4.271384111</d>
<d>3.453510496</d>
<d>4.077638929</d>
<d>2.725088284</d>
<d>2.365094805</d>
<d>2.174645458</d>
<d>1.464759828</d>
<d>2.538517391</d>
<d>2.460070082</d>
<d>2.873382193</d>
<d>3.458548618</d>
<d>4.380197051</d>
<d>4.503785647</d>
<d>4.109885158</d>
<d>3.05098748</d>
<d>2.731157863</d>
<d>2.32701894</d>
<d>1.986201457</d>
<d>1.628942022</d>
<d>1.789307827</d>
<d>3.078216284</d>
<d>4.006497469</d>
<d>3.998928574</d>
<d>4.093413178</d>
<d>3.733060821</d>
<d>4.556979391</d>
<d>4.39915843</d>
<d>5.656038726</d>
<d>6.573663013</d>
<d>6.152371772</d>
<d>6.066723515</d>
<d>5.040859226</d>
<d>5.042897995</d>
<d>4.700584137</d>
<d>3.953700563</d>
<d>4.512377817</d>
<d>3.679452513</d>
<d>4.807561915</d>
<d>4.035862602</d>
<d>2.744866806</d>
<d>2.050056655</d>
<d>1.899834868</d>
<d>2.491244396</d>
<d>2.858862228</d>
<d>2.647696554</d>
<d>3.566663793</d>
<d>3.169426056</d>
<d>3.687432752</d>
<d>2.779605299</d>
<d>3.049017986</d>
<d>3.448055651</d>
<d>4.035115127</d>
<d>4.084186708</d>
<d>3.823757537</d>
<d>3.433509393</d>
<d>2.290263747</d>
<d>1.600314607</d>
<d>1.307086946</d>
<d>1.927529412</d>
<d>3.285603107</d>
<d>4.094279468</d>
<d>5.430797511</d>
<d>4.522357551</d>
<d>3.909630937</d>
<d>2.670415222</d>
<d>0.999446526</d>
<d>0.029810774</d>
<d>1.096978299</d>
<d>3.005218093</d>
<d>4.183532066</d>
<d>5.439394252</d>
<d>5.271615215</d>
<d>4.389223314</d>
<d>2.773536958</d>
<d>1.45432495</d>
<d>0.990663607</d>
<d>0.522267415</d>
<d>1.940647567</d>
<d>3.321998777</d>
<d>4.144273669</d>
<d>4.511396092</d>
<d>3.550939897</d>
<d>4.065571056</d>
<d>2.383596824</d>
<d>2.174403226</d>
<d>2.255370843</d>
<d>2.850737236</d>
<d>3.380665597</d>
<d>4.015003433</d>
<d>4.398879935</d>
<d>4.121698771</d>
<d>3.625136303</d>
<d>2.82432948</d>
<d>2.099765855</d>
<d>1.487503119</d>
<d>2.210953229</d>
<d>2.044737484</d>
<d>1.741696764</d>
<d>1.991176418</d>
<d>2.360119937</d>
<d>2.262498789</d>
<d>1.231626901</d>
<d>1.32157859</d>
<d>1.162040195</d>
<d>0.778507054</d>
<d>1.650558556</d>
<d>0.591641798</d>
<d>0.767913413</d>
<d>0.744957181</d>
<d>1.012040758</d>
<d>1.368965047</d>
<d>1.523504922</d>
<d>2.935693349</d>
<d>2.264669932</d>
<d>2.142039073</d>
<d>3.088512678</d>
<d>2.809267605</d>
<d>3.311272502</d>
<d>3.74941765</d>
<d>3.771867399</d>
<d>3.931749155</d>
<d>4.280782029</d>
<d>4.595092683</d>
<d>4.112613354</d>
<d>3.673840316</d>
<d>3.903894951</d>
<d>3.702107141</d>
<d>3.517073409</d>
<d>3.834419222</d>
<d>3.933112169</d>
<d>4.367129501</d>
<d>3.976797603</d>
<d>4.703889904</d>
<d>4.281385319</d>
<d>4.044875288</d>
<d>3.214202498</d>
<d>3.615197298</d>
<d>3.428828096</d>
<d>4.04252685</d>
<d>4.766462585</d>
<d>4.391913515</d>
<d>4.110483129</d>
<d>4.856879041</d>
<d>3.522855456</d>
<d>2.256090998</d>
<d>1.986679842</d>
<d>3.47936069</d>
<d>3.30428228</d>
<d>2.691533919</d>
<d>3.032068348</d>
<d>2.976575209</d>
<d>3.843891863</d>
<d>4.040923967</d>
<d>3.692113047</d>
<d>3.13970365</d>
<d>4.779407608</d>
<d>5.406218249</d>
<d>5.682771524</d>
<d>5.386893779</d>
<d>6.298766569</d>
<d>5.86205744</d>
<d>5.973740868</d>
<d>5.480577778</d>
<d>5.170009863</d>
<d>4.093974807</d>
<d>3.558805114</d>
<d>2.966938648</d>
<d>2.327480886</d>
<d>2.202543306</d>
<d>2.375658355</d>
<d>3.013031351</d>
<d>3.91992648</d>
<d>4.119750931</d>
<d>4.509090719</d>
<d>3.84280215</d>
<d>3.995763743</d>
<d>3.692237398</d>
<d>1.723379922</d>
<d>2.402638114</d>
<d>2.998004506</d>
<d>3.480220165</d>
<d>3.095108741</d>
<d>2.269049394</d>
<d>2.420068485</d>
<d>1.946937445</d>
<d>3.32112568</d>
<d>2.505628686</d>
<d>1.955283197</d>
<d>2.46044466</d>
<d>2.267276701</d>
<d>1.211395102</d>
<d>1.029639342</d>
<d>0.940395234</d>
<d>0.859649571</d>
<d>0.554666349</d>
<d>1.970375561</d>
<d>2.432680576</d>
<d>3.94320816</d>
<d>3.907382852</d>
<d>4.418129008</d>
<d>4.205414624</d>
<d>4.268899725</d>
<d>3.247254721</d>
<d>1.966649937</d>
<d>0.663220757</d>
<d>0.616104657</d>
<d>0.496144738</d>
<d>0.980424305</d>
<d>1.067705054</d>
<d>1.589013563</d>
<d>2.048283419</d>
<d>2.798200116</d>
<d>2.827205808</d>
<d>3.005784145</d>
<d>2.429457693</d>
<d>2.497677642</d>
<d>2.516728013</d>
<d>2.625619095</d>
<d>2.719577193</d>
<d>2.727336772</d>
<d>3.530429457</d>
<d>3.9112708</d>
<d>2.809497595</d>
<d>1.647506831</d>
<d>1.043742541</d>
<d>0.914312925</d>
<d>1.566618904</d>
<d>1.713631584</d>
<d>1.736688365</d>
<d>1.57447903</d>
<d>2.426377075</d>
<d>1.485710118</d>
<d>1.062234282</d>
<d>0.531435219</d>
<d>0.752384377</d>
<d>0.828374333</d>
<d>1.233011324</d>
<d>1.018597252</d>
<d>1.615420521</d>
<d>2.048345195</d>
<d>2.221338838</d>
<d>2.62561161</d>
<d>3.630845896</d>
<d>3.897565254</d>
<d>4.363391054</d>
<d>4.308019322</d>
<d>4.540866304</d>
<d>3.492026274</d>
<d>2.941559378</d>
<d>1.891019659</d>
<d>0.625954891</d>
<d>0.866086255</d>
<d>0.571665387</d>
<d>0.650204877</d>
<d>1.459489287</d>
<d>2.322799531</d>
<d>3.315407555</d>
<d>3.738134094</d>
<d>3.560141932</d>
<d>3.683601778</d>
<d>3.45935379</d>
<d>3.496372302</d>
<d>3.436022909</d>
<d>2.40685071</d>
<d>2.765353282</d>
<d>1.799312394</d>
<d>1.425370356</d>
<d>1.480478417</d>
<d>1.43530482</d>
<d>0.900742159</d>
<d>1.247468312</d>
<d>1.691076747</d>
<d>2.261312022</d>
<d>1.841478377</d>
<d>2.929876024</d>
<d>2.230200705</d>
<d>2.771662671</d>
<d>3.032797336</d>
<d>3.745320872</d>
<d>3.92584171</d>
<d>4.084023777</d>
<d>4.225330359</d>
<d>4.559066031</d>
<d>3.690635862</d>
<d>4.009681364</d>
<d>2.744252376</d>
<d>2.137088708</d>
<d>1.725510696</d>
<d>2.258231404</d>
<d>3.205190634</d>
<d>2.310051064</d>
<d>2.662847536</d>
<d>2.942314563</d>
<d>3.106688355</d>
<d>4.202856009</d>
<d>4.395153265</d>
<d>4.798090567</d>
<d>5.686774741</d>
<d>6.482218826</d>
<d>6.662132632</d>
<d>5.418071164</d>
<d>5.026039578</d>
<d>4.36472867</d>
<d>3.540490418</d>
<d>3.1745612</d>
<d>2.736638007</d>
<d>2.935734019</d>
<d>2.845882879</d>
<d>2.251831098</d>
<d>2.548295016</d>
<d>3.027718329</d>
<d>3.47241942</d>
<d>3.579853626</d>
<d>4.389380849</d>
<d>4.837481319</d>
<d>3.937728307</d>
<d>4.605574301</d>
<d>4.458419355</d>
<d>3.966227516</d>
<d>4.388711243</d>
<d>4.57360271</d>
<d>4.13021623</d>
<d>3.318361428</d>
<d>3.019084305</d>
<d>2.926440818</d>
<d>2.050604861</d>
<d>2.227847727</d>
<d>1.89263432</d>
<d>2.478409609</d>
<d>3.189354863</d>
<d>3.523576161</d>
<d>3.530000269</d>
<d>3.165285115</d>
<d>3.71645959</d>
<d>4.426433593</d>
<d>4.423266598</d>
<d>3.730390035</d>
<d>2.717000664</d>
<d>2.363819115</d>
<d>1.660744417</d>
<d>1.150584436</d>
<d>1.523291552</d>
<d>2.784571865</d>
<d>4.455841479</d>
<d>5.033968167</d>
<d>5.647788328</d>
<d>5.615483805</d>
<d>4.330508392</d>
<d>3.593439911</d>
<d>3.329856261</d>
<d>3.574115441</d>
<d>4.190242322</d>
<d>5.537114124</d>
<d>5.929974697</d>
<d>4.965390686</d>
<d>4.408853472</d>
<d>3.155079496</d>
<d>2.45928918</d>
<d>2.19934772</d>
<d>2.442028617</d>
<d>3.530062046</d>
<d>4.388758847</d>
<d>5.394357353</d>
<d>4.970517298</d>
<d>4.675489398</d>
<d>4.765441087</d>
<d>4.429499241</d>
<d>5.301793556</d>
<d>5.11991639</d>
<d>5.906133591</d>
<d>5.741253316</d>
<d>5.41502398</d>
<d>4.928295427</d>
<d>4.54196994</d>
<d>3.847150875</d>
<d>3.786437262</d>
<d>3.745512888</d>
<d>4.0876256</d>
<d>3.804252711</d>
<d>4.479868712</d>
<d>3.727503029</d>
<d>3.858004444</d>
<d>3.989962736</d>
<d>4.595891482</d>
<d>4.305598431</d>
<d>5.619690513</d>
<d>5.320049172</d>
<d>5.90351774</d>
<d>6.127866277</d>
<d>6.38038109</d>
<d>5.552764948</d>
<d>4.793831309</d>
<d>3.795287081</d>
<d>3.007071962</d>
<d>1.39897863</d>
</Subcolumn>
<Subcolumn>
<d/>
<d/>
<d/>
<d/>
<d/>
<d/>
<d/>
<d/>
<d/>
<d/>
<d/>
<d/>
<d/>
<d/>
<d/>
<d/>
<d/>
<d/>
<d/>
<d/>
<d/>
<d/>
<d/>
<d/>
<d/>
<d/>
<d/>
<d/>
<d/>
<d/>
<d/>
<d/>
<d/>
<d/>
<d/>
<d/>
<d/>
<d/>
<d/>
<d/>
<d/>
<d/>
<d/>
<d/>
<d/>
<d/>
<d/>
<d/>
<d/>
<d/>
<d/>
<d/>
<d/>
<d/>
<d/>
<d/>
<d/>
<d/>
<d/>
<d/>
<d/>
<d/>
<d/>
<d/>
<d/>
<d/>
<d/>
<d/>
<d/>
<d/>
<d/>
<d/>
<d/>
<d/>
<d/>
<d/>
<d/>
<d/>
<d/>
<d/>
<d/>
<d/>
<d/>
<d/>
<d/>
<d/>
<d/>
<d/>
<d/>
<d/>
<d/>
<d/>
<d/>
<d/>
<d/>
<d/>
<d/>
<d/>
<d/>
<d/>
<d/>
<d/>
<d/>
<d/>
<d/>
<d/>
<d/>
<d/>
<d/>
<d/>
<d/>
<d/>
<d/>
<d/>
<d/>
<d/>
<d/>
<d/>
<d/>
<d/>
<d/>
<d/>
<d/>
<d/>
<d/>
<d/>
<d/>
<d/>
<d/>
<d/>
<d/>
<d/>
<d/>
<d/>
<d/>
<d/>
<d/>
<d/>
<d/>
<d/>
<d/>
<d/>
<d/>
<d/>
<d/>
<d/>
<d/>
<d/>
<d/>
<d/>
<d/>
<d/>
<d/>
<d/>
<d/>
<d/>
<d/>
<d/>
<d/>
<d/>
<d/>
<d/>
<d/>
<d/>
<d/>
<d/>
<d/>
<d/>
<d/>
<d/>
<d/>
<d/>
<d/>
<d/>
<d/>
<d/>
<d/>
<d/>
<d/>
<d/>
<d/>
<d/>
<d/>
<d/>
<d/>
<d/>
<d/>
<d/>
<d/>
<d/>
<d/>
<d/>
<d/>
<d/>
<d/>
<d/>
<d/>
<d/>
<d/>
<d/>
<d/>
<d/>
<d/>
<d/>
<d/>
<d/>
<d/>
<d/>
<d/>
<d/>
<d/>
<d/>
<d/>
<d/>
<d/>
<d/>
<d/>
<d/>
<d/>
<d/>
<d/>
<d/>
<d/>
<d/>
<d/>
<d/>
<d/>
<d/>
<d/>
<d/>
<d/>
<d/>
<d/>
<d/>
<d/>
<d/>
<d/>
<d/>
<d/>
<d/>
<d/>
<d/>
<d/>
<d/>
<d/>
<d/>
<d/>
<d/>
<d/>
<d/>
<d/>
<d/>
<d/>
<d/>
<d/>
<d/>
<d/>
<d/>
<d/>
<d/>
<d/>
<d/>
<d/>
<d/>
<d/>
<d/>
<d/>
<d/>
<d/>
<d/>
<d/>
<d/>
<d/>
<d/>
<d/>
<d/>
<d/>
<d/>
<d/>
<d/>
<d/>
<d/>
<d/>
<d/>
<d/>
<d/>
<d/>
<d/>
<d/>
<d/>
<d/>
<d/>
<d/>
<d/>
<d/>
<d/>
<d/>
<d/>
<d/>
<d/>
<d/>
<d/>
<d/>
<d/>
<d/>
<d/>
<d/>
<d/>
<d/>
<d/>
<d/>
<d/>
<d/>
<d/>
<d/>
<d/>
<d/>
<d/>
<d/>
<d/>
<d/>
<d/>
<d/>
<d/>
<d/>
<d/>
<d/>
<d/>
<d/>
<d/>
<d/>
<d/>
<d/>
<d/>
<d/>
<d/>
<d/>
<d/>
<d/>
<d/>
<d/>
<d/>
<d/>
<d/>
<d/>
<d/>
<d/>
<d/>
<d/>
<d/>
<d/>
<d/>
<d/>
<d/>
<d/>
<d/>
<d/>
<d/>
<d/>
<d/>
<d/>
<d/>
<d/>
<d/>
<d/>
<d/>
<d/>
<d/>
<d/>
<d/>
<d/>
<d/>
<d/>
<d/>
<d/>
<d/>
<d/>
<d/>
<d/>
<d/>
<d/>
<d/>
<d/>
<d/>
<d/>
<d/>
<d/>
<d/>
<d/>
<d/>
<d/>
<d/>
<d/>
<d/>
<d/>
<d/>
<d/>
<d/>
<d/>
<d/>
<d/>
<d/>
<d/>
<d/>
<d/>
<d/>
<d/>
<d/>
<d/>
<d/>
<d/>
<d/>
<d/>
<d/>
<d/>
<d/>
<d/>
<d/>
<d/>
<d/>
<d/>
<d/>
<d/>
<d/>
<d/>
<d/>
<d/>
<d/>
<d/>
<d/>
<d/>
<d/>
<d/>
<d/>
<d/>
<d/>
<d/>
<d/>
<d/>
<d/>
<d/>
<d/>
<d/>
<d/>
<d/>
<d/>
<d/>
<d/>
<d/>
<d/>
<d/>
<d/>
<d/>
<d/>
<d/>
<d/>
<d/>
<d/>
<d/>
<d/>
<d/>
<d/>
<d/>
<d/>
<d/>
<d/>
<d/>
<d/>
<d/>
<d/>
<d/>
<d/>
<d/>
<d/>
<d/>
<d/>
<d/>
<d/>
<d/>
<d/>
<d/>
<d/>
<d/>
<d/>
<d/>
<d/>
<d/>
<d/>
<d/>
<d/>
<d/>
<d/>
<d/>
<d/>
<d/>
<d/>
<d/>
<d/>
<d/>
<d/>
<d/>
<d/>
<d/>
<d/>
<d/>
<d/>
<d/>
<d/>
<d/>
<d/>
<d/>
<d/>
<d/>
<d/>
<d/>
<d/>
<d/>
<d/>
<d/>
<d/>
<d/>
<d/>
<d/>
<d/>
<d/>
<d/>
<d/>
<d/>
<d/>
<d/>
<d/>
<d/>
<d/>
<d/>
<d/>
<d/>
<d/>
<d/>
<d/>
<d/>
<d/>
<d/>
<d/>
<d/>
<d/>
<d/>
<d/>
<d/>
<d/>
<d/>
<d/>
<d/>
<d/>
<d/>
<d/>
<d/>
<d/>
<d/>
<d/>
<d/>
<d/>
<d/>
<d/>
<d/>
<d/>
<d/>
<d/>
<d/>
<d/>
<d/>
<d/>
<d/>
<d/>
<d/>
<d/>
<d/>
<d/>
<d/>
<d/>
<d/>
<d/>
<d/>
<d/>
<d/>
<d/>
<d/>
<d/>
<d/>
<d/>
<d/>
<d/>
<d/>
<d/>
<d/>
<d/>
<d/>
<d/>
<d/>
<d/>
<d/>
<d/>
<d/>
<d/>
<d/>
<d/>
<d/>
<d/>
<d/>
<d/>
<d/>
<d/>
<d/>
<d/>
<d/>
<d/>
<d/>
<d/>
<d/>
<d/>
<d/>
<d/>
<d/>
<d/>
<d/>
<d/>
<d/>
<d/>
<d/>
<d/>
<d/>
<d/>
<d/>
<d/>
<d/>
<d/>
<d/>
<d/>
<d/>
<d/>
<d/>
<d/>
<d/>
<d/>
<d/>
<d/>
<d/>
<d/>
<d/>
<d/>
<d/>
<d/>
<d/>
<d/>
<d/>
<d/>
<d/>
<d/>
<d/>
<d/>
<d/>
<d/>
<d/>
<d/>
<d/>
<d/>
<d/>
<d/>
<d/>
<d/>
<d/>
<d/>
<d/>
<d/>
<d/>
<d/>
<d/>
<d/>
<d/>
<d/>
<d/>
<d/>
<d/>
<d/>
<d/>
<d/>
<d/>
<d/>
<d/>
<d/>
<d/>
<d/>
<d/>
<d/>
<d/>
<d/>
<d/>
<d/>
<d/>
<d/>
<d/>
<d/>
<d/>
<d/>
<d/>
<d/>
<d/>
<d/>
<d/>
<d/>
<d/>
<d/>
<d/>
<d/>
<d/>
<d/>
<d/>
<d/>
<d/>
<d/>
<d/>
<d/>
<d/>
<d/>
<d/>
<d/>
<d/>
<d/>
<d/>
<d/>
<d/>
<d/>
<d/>
<d/>
<d/>
<d/>
<d/>
<d/>
<d/>
<d/>
<d/>
<d/>
<d/>
<d/>
<d/>
<d/>
<d/>
<d/>
<d/>
<d/>
<d/>
<d/>
<d/>
<d/>
<d/>
<d/>
<d/>
<d/>
<d/>
<d/>
<d/>
<d/>
<d/>
<d/>
<d/>
<d/>
<d/>
<d/>
<d/>
<d/>
<d/>
<d/>
<d/>
<d/>
<d/>
<d/>
<d/>
<d/>
<d/>
<d/>
<d/>
<d/>
<d/>
<d/>
<d/>
<d/>
<d/>
<d/>
<d/>
<d/>
<d/>
<d/>
<d/>
<d/>
<d/>
<d/>
<d/>
<d/>
<d/>
<d/>
<d/>
<d/>
<d/>
<d/>
<d/>
<d/>
<d/>
<d/>
<d/>
<d/>
<d/>
<d/>
<d/>
<d/>
<d/>
<d/>
<d/>
<d/>
<d/>
<d/>
<d/>
<d/>
<d/>
<d/>
<d/>
<d/>
<d/>
<d/>
<d/>
<d/>
<d/>
<d/>
<d/>
<d/>
<d/>
<d/>
<d/>
<d/>
<d/>
<d/>
<d/>
<d/>
<d/>
<d/>
<d/>
<d/>
<d/>
<d/>
<d/>
<d/>
<d/>
<d/>
<d/>
<d/>
<d/>
<d/>
<d/>
<d/>
<d/>
<d/>
<d/>
<d/>
<d/>
<d/>
<d/>
<d/>
<d/>
<d/>
<d/>
<d/>
<d/>
<d/>
<d/>
<d/>
<d/>
<d/>
<d/>
<d/>
<d/>
<d/>
<d/>
<d/>
<d/>
<d/>
<d/>
<d/>
<d/>
<d/>
<d/>
<d/>
<d/>
<d/>
<d/>
<d/>
<d/>
<d/>
<d/>
<d/>
<d/>
<d/>
<d/>
<d/>
<d/>
<d/>
<d/>
<d/>
<d/>
<d/>
<d/>
<d/>
<d/>
<d/>
<d/>
<d/>
<d/>
<d/>
<d/>
<d/>
<d/>
<d/>
<d/>
<d/>
<d/>
<d/>
<d/>
<d/>
<d/>
<d/>
<d/>
<d/>
<d/>
<d/>
<d/>
<d/>
<d/>
<d/>
<d/>
<d/>
<d/>
<d/>
<d/>
<d/>
<d/>
<d/>
<d/>
<d/>
<d/>
<d/>
<d/>
<d/>
<d/>
<d/>
<d/>
<d/>
<d/>
<d/>
<d/>
<d/>
<d/>
<d/>
<d/>
<d/>
<d/>
<d/>
<d/>
<d/>
<d/>
<d/>
<d/>
<d/>
<d/>
<d/>
<d/>
<d/>
<d/>
<d/>
<d/>
<d/>
<d/>
<d/>
<d/>
<d/>
<d/>
<d/>
<d/>
<d/>
<d/>
<d/>
<d/>
<d/>
<d/>
<d/>
<d/>
<d/>
<d/>
<d/>
<d/>
<d/>
<d/>
<d/>
<d/>
<d/>
<d/>
<d/>
<d/>
<d/>
<d/>
<d/>
<d/>
<d/>
<d/>
<d/>
<d/>
<d/>
<d/>
<d/>
<d/>
<d/>
<d/>
<d/>
<d/>
<d/>
<d/>
<d/>
<d/>
<d/>
<d/>
<d/>
<d/>
<d/>
<d/>
<d/>
<d/>
<d/>
<d/>
<d/>
<d/>
<d/>
<d/>
<d/>
<d/>
<d/>
<d/>
<d/>
<d/>
<d/>
<d/>
<d/>
<d/>
<d/>
<d/>
<d/>
<d/>
<d/>
<d/>
<d/>
<d/>
<d/>
<d/>
<d/>
<d/>
<d/>
<d/>
<d/>
<d/>
<d/>
<d/>
<d/>
<d/>
<d/>
<d/>
<d/>
<d/>
<d/>
<d/>
<d/>
<d/>
<d/>
<d/>
<d/>
<d/>
<d/>
<d/>
<d/>
<d/>
<d/>
<d/>
<d/>
<d/>
<d/>
<d/>
<d/>
<d/>
<d/>
<d/>
<d/>
<d/>
<d/>
<d/>
<d/>
<d/>
<d/>
<d/>
<d/>
<d/>
<d/>
<d/>
<d/>
<d/>
<d/>
<d/>
<d/>
<d/>
<d/>
<d/>
<d/>
<d/>
<d/>
<d/>
<d/>
<d/>
<d/>
<d/>
<d/>
<d/>
<d/>
<d/>
<d/>
<d/>
<d/>
<d/>
<d/>
<d/>
<d/>
<d/>
<d/>
<d/>
<d/>
<d/>
<d/>
<d/>
<d/>
<d/>
<d/>
<d/>
<d/>
<d/>
<d/>
<d/>
<d/>
<d/>
<d/>
<d/>
<d/>
<d/>
<d/>
<d/>
<d/>
<d/>
<d/>
<d/>
<d/>
<d/>
<d/>
<d/>
<d/>
<d/>
<d/>
<d/>
<d/>
<d/>
<d/>
<d/>
<d/>
<d/>
<d/>
<d/>
<d/>
<d/>
<d/>
<d/>
<d/>
<d/>
<d/>
<d/>
<d/>
<d/>
<d/>
<d/>
<d/>
<d/>
<d/>
<d/>
<d/>
<d/>
<d/>
<d/>
<d/>
<d/>
<d/>
<d/>
<d/>
<d/>
<d/>
<d/>
<d/>
<d/>
<d/>
<d/>
<d/>
<d/>
<d/>
<d/>
<d/>
<d/>
<d/>
<d/>
<d/>
<d/>
<d/>
<d/>
<d/>
<d/>
<d/>
<d/>
<d/>
<d/>
<d/>
<d/>
<d/>
<d/>
<d/>
<d/>
<d/>
<d/>
<d/>
<d/>
<d/>
<d/>
<d/>
<d/>
<d/>
<d/>
<d/>
<d/>
<d/>
<d/>
<d/>
<d/>
<d/>
<d/>
<d/>
<d/>
<d/>
<d/>
<d/>
<d/>
<d/>
<d/>
<d/>
<d/>
<d/>
<d/>
<d/>
<d/>
<d/>
<d/>
<d/>
<d/>
<d/>
<d/>
<d/>
<d/>
<d/>
<d/>
<d/>
<d/>
<d/>
<d/>
<d/>
<d/>
<d/>
<d/>
<d/>
<d/>
<d/>
<d/>
<d/>
<d/>
<d/>
<d/>
<d/>
<d/>
<d/>
<d/>
<d/>
<d/>
<d/>
<d/>
<d/>
<d/>
<d/>
<d/>
<d/>
<d/>
<d/>
<d/>
<d/>
<d/>
<d/>
<d/>
<d/>
<d/>
<d/>
<d/>
<d/>
<d/>
<d/>
<d/>
<d/>
<d/>
<d/>
<d/>
<d/>
<d/>
<d/>
<d/>
<d/>
<d/>
<d/>
<d/>
<d/>
<d/>
<d/>
<d/>
<d/>
<d/>
<d/>
<d/>
<d/>
<d/>
<d/>
<d/>
<d/>
<d/>
<d/>
<d/>
<d/>
<d/>
<d/>
<d/>
<d/>
<d/>
<d/>
<d/>
<d/>
<d/>
<d/>
<d/>
<d/>
<d/>
<d/>
<d/>
<d/>
<d/>
<d/>
<d/>
<d/>
<d/>
<d/>
<d/>
<d/>
<d/>
<d/>
<d/>
<d/>
<d/>
<d/>
<d/>
<d/>
<d/>
<d/>
<d/>
<d/>
<d/>
<d/>
<d/>
<d/>
<d/>
<d/>
<d/>
<d/>
<d/>
<d/>
<d/>
<d/>
<d/>
<d/>
<d/>
<d/>
<d/>
<d/>
<d/>
<d/>
<d/>
<d/>
<d/>
<d/>
<d/>
<d/>
<d/>
<d/>
<d/>
<d/>
<d/>
<d/>
<d/>
<d/>
<d/>
<d/>
<d/>
<d/>
<d/>
<d/>
<d/>
<d/>
<d/>
<d/>
<d/>
<d/>
<d/>
<d/>
<d/>
<d/>
<d/>
</Subcolumn>
<Subcolumn>
<d/>
<d/>
<d/>
<d/>
<d/>
<d/>
<d/>
<d/>
<d/>
<d/>
<d/>
<d/>
<d/>
<d/>
<d/>
<d/>
<d/>
<d/>
<d/>
<d/>
<d/>
<d/>
<d/>
<d/>
<d/>
<d/>
<d/>
<d/>
<d/>
<d/>
<d/>
<d/>
<d/>
<d/>
<d/>
<d/>
<d/>
<d/>
<d/>
<d/>
<d/>
<d/>
<d/>
<d/>
<d/>
<d/>
<d/>
<d/>
<d/>
<d/>
<d/>
<d/>
<d/>
<d/>
<d/>
<d/>
<d/>
<d/>
<d/>
<d/>
<d/>
<d/>
<d/>
<d/>
<d/>
<d/>
<d/>
<d/>
<d/>
<d/>
<d/>
<d/>
<d/>
<d/>
<d/>
<d/>
<d/>
<d/>
<d/>
<d/>
<d/>
<d/>
<d/>
<d/>
<d/>
<d/>
<d/>
<d/>
<d/>
<d/>
<d/>
<d/>
<d/>
<d/>
<d/>
<d/>
<d/>
<d/>
<d/>
<d/>
<d/>
<d/>
<d/>
<d/>
<d/>
<d/>
<d/>
<d/>
<d/>
<d/>
<d/>
<d/>
<d/>
<d/>
<d/>
<d/>
<d/>
<d/>
<d/>
<d/>
<d/>
<d/>
<d/>
<d/>
<d/>
<d/>
<d/>
<d/>
<d/>
<d/>
<d/>
<d/>
<d/>
<d/>
<d/>
<d/>
<d/>
<d/>
<d/>
<d/>
<d/>
<d/>
<d/>
<d/>
<d/>
<d/>
<d/>
<d/>
<d/>
<d/>
<d/>
<d/>
<d/>
<d/>
<d/>
<d/>
<d/>
<d/>
<d/>
<d/>
<d/>
<d/>
<d/>
<d/>
<d/>
<d/>
<d/>
<d/>
<d/>
<d/>
<d/>
<d/>
<d/>
<d/>
<d/>
<d/>
<d/>
<d/>
<d/>
<d/>
<d/>
<d/>
<d/>
<d/>
<d/>
<d/>
<d/>
<d/>
<d/>
<d/>
<d/>
<d/>
<d/>
<d/>
<d/>
<d/>
<d/>
<d/>
<d/>
<d/>
<d/>
<d/>
<d/>
<d/>
<d/>
<d/>
<d/>
<d/>
<d/>
<d/>
<d/>
<d/>
<d/>
<d/>
<d/>
<d/>
<d/>
<d/>
<d/>
<d/>
<d/>
<d/>
<d/>
<d/>
<d/>
<d/>
<d/>
<d/>
<d/>
<d/>
<d/>
<d/>
<d/>
<d/>
<d/>
<d/>
<d/>
<d/>
<d/>
<d/>
<d/>
<d/>
<d/>
<d/>
<d/>
<d/>
<d/>
<d/>
<d/>
<d/>
<d/>
<d/>
<d/>
<d/>
<d/>
<d/>
<d/>
<d/>
<d/>
<d/>
<d/>
<d/>
<d/>
<d/>
<d/>
<d/>
<d/>
<d/>
<d/>
<d/>
<d/>
<d/>
<d/>
<d/>
<d/>
<d/>
<d/>
<d/>
<d/>
<d/>
<d/>
<d/>
<d/>
<d/>
<d/>
<d/>
<d/>
<d/>
<d/>
<d/>
<d/>
<d/>
<d/>
<d/>
<d/>
<d/>
<d/>
<d/>
<d/>
<d/>
<d/>
<d/>
<d/>
<d/>
<d/>
<d/>
<d/>
<d/>
<d/>
<d/>
<d/>
<d/>
<d/>
<d/>
<d/>
<d/>
<d/>
<d/>
<d/>
<d/>
<d/>
<d/>
<d/>
<d/>
<d/>
<d/>
<d/>
<d/>
<d/>
<d/>
<d/>
<d/>
<d/>
<d/>
<d/>
<d/>
<d/>
<d/>
<d/>
<d/>
<d/>
<d/>
<d/>
<d/>
<d/>
<d/>
<d/>
<d/>
<d/>
<d/>
<d/>
<d/>
<d/>
<d/>
<d/>
<d/>
<d/>
<d/>
<d/>
<d/>
<d/>
<d/>
<d/>
<d/>
<d/>
<d/>
<d/>
<d/>
<d/>
<d/>
<d/>
<d/>
<d/>
<d/>
<d/>
<d/>
<d/>
<d/>
<d/>
<d/>
<d/>
<d/>
<d/>
<d/>
<d/>
<d/>
<d/>
<d/>
<d/>
<d/>
<d/>
<d/>
<d/>
<d/>
<d/>
<d/>
<d/>
<d/>
<d/>
<d/>
<d/>
<d/>
<d/>
<d/>
<d/>
<d/>
<d/>
<d/>
<d/>
<d/>
<d/>
<d/>
<d/>
<d/>
<d/>
<d/>
<d/>
<d/>
<d/>
<d/>
<d/>
<d/>
<d/>
<d/>
<d/>
<d/>
<d/>
<d/>
<d/>
<d/>
<d/>
<d/>
<d/>
<d/>
<d/>
<d/>
<d/>
<d/>
<d/>
<d/>
<d/>
<d/>
<d/>
<d/>
<d/>
<d/>
<d/>
<d/>
<d/>
<d/>
<d/>
<d/>
<d/>
<d/>
<d/>
<d/>
<d/>
<d/>
<d/>
<d/>
<d/>
<d/>
<d/>
<d/>
<d/>
<d/>
<d/>
<d/>
<d/>
<d/>
<d/>
<d/>
<d/>
<d/>
<d/>
<d/>
<d/>
<d/>
<d/>
<d/>
<d/>
<d/>
<d/>
<d/>
<d/>
<d/>
<d/>
<d/>
<d/>
<d/>
<d/>
<d/>
<d/>
<d/>
<d/>
<d/>
<d/>
<d/>
<d/>
<d/>
<d/>
<d/>
<d/>
<d/>
<d/>
<d/>
<d/>
<d/>
<d/>
<d/>
<d/>
<d/>
<d/>
<d/>
<d/>
<d/>
<d/>
<d/>
<d/>
<d/>
<d/>
<d/>
<d/>
<d/>
<d/>
<d/>
<d/>
<d/>
<d/>
<d/>
<d/>
<d/>
<d/>
<d/>
<d/>
<d/>
<d/>
<d/>
<d/>
<d/>
<d/>
<d/>
<d/>
<d/>
<d/>
<d/>
<d/>
<d/>
<d/>
<d/>
<d/>
<d/>
<d/>
<d/>
<d/>
<d/>
<d/>
<d/>
<d/>
<d/>
<d/>
<d/>
<d/>
<d/>
<d/>
<d/>
<d/>
<d/>
<d/>
<d/>
<d/>
<d/>
<d/>
<d/>
<d/>
<d/>
<d/>
<d/>
<d/>
<d/>
<d/>
<d/>
<d/>
<d/>
<d/>
<d/>
<d/>
<d/>
<d/>
<d/>
<d/>
<d/>
<d/>
<d/>
<d/>
<d/>
<d/>
<d/>
<d/>
<d/>
<d/>
<d/>
<d/>
<d/>
<d/>
<d/>
<d/>
<d/>
<d/>
<d/>
<d/>
<d/>
<d/>
<d/>
<d/>
<d/>
<d/>
<d/>
<d/>
<d/>
<d/>
<d/>
<d/>
<d/>
<d/>
<d/>
<d/>
<d/>
<d/>
<d/>
<d/>
<d/>
<d/>
<d/>
<d/>
<d/>
<d/>
<d/>
<d/>
<d/>
<d/>
<d/>
<d/>
<d/>
<d/>
<d/>
<d/>
<d/>
<d/>
<d/>
<d/>
<d/>
<d/>
<d/>
<d/>
<d/>
<d/>
<d/>
<d/>
<d/>
<d/>
<d/>
<d/>
<d/>
<d/>
<d/>
<d/>
<d/>
<d/>
<d/>
<d/>
<d/>
<d/>
<d/>
<d/>
<d/>
<d/>
<d/>
<d/>
<d/>
<d/>
<d/>
<d/>
<d/>
<d/>
<d/>
<d/>
<d/>
<d/>
<d/>
<d/>
<d/>
<d/>
<d/>
<d/>
<d/>
<d/>
<d/>
<d/>
<d/>
<d/>
<d/>
<d/>
<d/>
<d/>
<d/>
<d/>
<d/>
<d/>
<d/>
<d/>
<d/>
<d/>
<d/>
<d/>
<d/>
<d/>
<d/>
<d/>
<d/>
<d/>
<d/>
<d/>
<d/>
<d/>
<d/>
<d/>
<d/>
<d/>
<d/>
<d/>
<d/>
<d/>
<d/>
<d/>
<d/>
<d/>
<d/>
<d/>
<d/>
<d/>
<d/>
<d/>
<d/>
<d/>
<d/>
<d/>
<d/>
<d/>
<d/>
<d/>
<d/>
<d/>
<d/>
<d/>
<d/>
<d/>
<d/>
<d/>
<d/>
<d/>
<d/>
<d/>
<d/>
<d/>
<d/>
<d/>
<d/>
<d/>
<d/>
<d/>
<d/>
<d/>
<d/>
<d/>
<d/>
<d/>
<d/>
<d/>
<d/>
<d/>
<d/>
<d/>
<d/>
<d/>
<d/>
<d/>
<d/>
<d/>
<d/>
<d/>
<d/>
<d/>
<d/>
<d/>
<d/>
<d/>
<d/>
<d/>
<d/>
<d/>
<d/>
<d/>
<d/>
<d/>
<d/>
<d/>
<d/>
<d/>
<d/>
<d/>
<d/>
<d/>
<d/>
<d/>
<d/>
<d/>
<d/>
<d/>
<d/>
<d/>
<d/>
<d/>
<d/>
<d/>
<d/>
<d/>
<d/>
<d/>
<d/>
<d/>
<d/>
<d/>
<d/>
<d/>
<d/>
<d/>
<d/>
<d/>
<d/>
<d/>
<d/>
<d/>
<d/>
<d/>
<d/>
<d/>
<d/>
<d/>
<d/>
<d/>
<d/>
<d/>
<d/>
<d/>
<d/>
<d/>
<d/>
<d/>
<d/>
<d/>
<d/>
<d/>
<d/>
<d/>
<d/>
<d/>
<d/>
<d/>
<d/>
<d/>
<d/>
<d/>
<d/>
<d/>
<d/>
<d/>
<d/>
<d/>
<d/>
<d/>
<d/>
<d/>
<d/>
<d/>
<d/>
<d/>
<d/>
<d/>
<d/>
<d/>
<d/>
<d/>
<d/>
<d/>
<d/>
<d/>
<d/>
<d/>
<d/>
<d/>
<d/>
<d/>
<d/>
<d/>
<d/>
<d/>
<d/>
<d/>
<d/>
<d/>
<d/>
<d/>
<d/>
<d/>
<d/>
<d/>
<d/>
<d/>
<d/>
<d/>
<d/>
<d/>
<d/>
<d/>
<d/>
<d/>
<d/>
<d/>
<d/>
<d/>
<d/>
<d/>
<d/>
<d/>
<d/>
<d/>
<d/>
<d/>
<d/>
<d/>
<d/>
<d/>
<d/>
<d/>
<d/>
<d/>
<d/>
<d/>
<d/>
<d/>
<d/>
<d/>
<d/>
<d/>
<d/>
<d/>
<d/>
<d/>
<d/>
<d/>
<d/>
<d/>
<d/>
<d/>
<d/>
<d/>
<d/>
<d/>
<d/>
<d/>
<d/>
<d/>
<d/>
<d/>
<d/>
<d/>
<d/>
<d/>
<d/>
<d/>
<d/>
<d/>
<d/>
<d/>
<d/>
<d/>
<d/>
<d/>
<d/>
<d/>
<d/>
<d/>
<d/>
<d/>
<d/>
<d/>
<d/>
<d/>
<d/>
<d/>
<d/>
<d/>
<d/>
<d/>
<d/>
<d/>
<d/>
<d/>
<d/>
<d/>
<d/>
<d/>
<d/>
<d/>
<d/>
<d/>
<d/>
<d/>
<d/>
<d/>
<d/>
<d/>
<d/>
<d/>
<d/>
<d/>
<d/>
<d/>
<d/>
<d/>
<d/>
<d/>
<d/>
<d/>
<d/>
<d/>
<d/>
<d/>
<d/>
<d/>
<d/>
<d/>
<d/>
<d/>
<d/>
<d/>
<d/>
<d/>
<d/>
<d/>
<d/>
<d/>
<d/>
<d/>
<d/>
<d/>
<d/>
<d/>
<d/>
<d/>
<d/>
<d/>
<d/>
<d/>
<d/>
<d/>
<d/>
<d/>
<d/>
<d/>
<d/>
<d/>
<d/>
<d/>
<d/>
<d/>
<d/>
<d/>
<d/>
<d/>
<d/>
<d/>
<d/>
<d/>
<d/>
<d/>
<d/>
<d/>
<d/>
<d/>
<d/>
<d/>
<d/>
<d/>
<d/>
<d/>
<d/>
<d/>
<d/>
<d/>
<d/>
<d/>
<d/>
<d/>
<d/>
<d/>
<d/>
<d/>
<d/>
<d/>
<d/>
<d/>
<d/>
<d/>
<d/>
<d/>
<d/>
<d/>
<d/>
<d/>
<d/>
<d/>
<d/>
<d/>
<d/>
<d/>
<d/>
<d/>
<d/>
<d/>
<d/>
<d/>
<d/>
<d/>
<d/>
<d/>
<d/>
<d/>
<d/>
<d/>
<d/>
<d/>
<d/>
<d/>
<d/>
<d/>
<d/>
<d/>
<d/>
<d/>
<d/>
<d/>
<d/>
<d/>
<d/>
<d/>
<d/>
<d/>
<d/>
<d/>
<d/>
<d/>
<d/>
<d/>
<d/>
<d/>
<d/>
<d/>
<d/>
<d/>
<d/>
<d/>
<d/>
<d/>
<d/>
<d/>
<d/>
<d/>
<d/>
<d/>
<d/>
<d/>
<d/>
<d/>
<d/>
<d/>
<d/>
<d/>
<d/>
<d/>
<d/>
<d/>
<d/>
<d/>
<d/>
<d/>
<d/>
<d/>
<d/>
<d/>
<d/>
<d/>
<d/>
<d/>
<d/>
<d/>
<d/>
<d/>
<d/>
<d/>
<d/>
<d/>
<d/>
<d/>
<d/>
<d/>
<d/>
<d/>
<d/>
<d/>
<d/>
<d/>
<d/>
<d/>
<d/>
<d/>
<d/>
<d/>
<d/>
<d/>
<d/>
<d/>
<d/>
<d/>
<d/>
<d/>
<d/>
<d/>
<d/>
<d/>
<d/>
<d/>
<d/>
<d/>
<d/>
<d/>
<d/>
<d/>
<d/>
<d/>
<d/>
<d/>
<d/>
<d/>
<d/>
<d/>
<d/>
<d/>
<d/>
<d/>
<d/>
<d/>
<d/>
<d/>
<d/>
<d/>
<d/>
<d/>
<d/>
<d/>
<d/>
<d/>
<d/>
<d/>
<d/>
<d/>
<d/>
<d/>
<d/>
<d/>
<d/>
<d/>
<d/>
<d/>
<d/>
<d/>
<d/>
<d/>
<d/>
<d/>
<d/>
<d/>
<d/>
<d/>
<d/>
<d/>
<d/>
<d/>
<d/>
<d/>
<d/>
<d/>
<d/>
<d/>
<d/>
<d/>
<d/>
<d/>
<d/>
<d/>
<d/>
<d/>
<d/>
<d/>
<d/>
<d/>
<d/>
<d/>
<d/>
<d/>
<d/>
<d/>
<d/>
<d/>
<d/>
<d/>
<d/>
<d/>
<d/>
<d/>
<d/>
<d/>
<d/>
<d/>
<d/>
<d/>
<d/>
<d/>
<d/>
<d/>
<d/>
<d/>
<d/>
<d/>
<d/>
<d/>
<d/>
<d/>
<d/>
<d/>
<d/>
<d/>
<d/>
<d/>
<d/>
<d/>
<d/>
<d/>
<d/>
<d/>
<d/>
<d/>
<d/>
<d/>
<d/>
<d/>
<d/>
<d/>
<d/>
<d/>
<d/>
<d/>
<d/>
<d/>
<d/>
<d/>
<d/>
<d/>
<d/>
<d/>
<d/>
<d/>
<d/>
<d/>
<d/>
<d/>
<d/>
<d/>
<d/>
<d/>
<d/>
<d/>
<d/>
<d/>
<d/>
<d/>
<d/>
<d/>
<d/>
<d/>
<d/>
<d/>
<d/>
<d/>
<d/>
<d/>
<d/>
<d/>
<d/>
<d/>
<d/>
<d/>
<d/>
<d/>
<d/>
<d/>
<d/>
<d/>
<d/>
<d/>
<d/>
<d/>
<d/>
<d/>
<d/>
<d/>
<d/>
<d/>
<d/>
<d/>
<d/>
<d/>
<d/>
</Subcolumn>
</YColumn>
<YColumn Width="342" Decimals="6" Subcolumns="3">
<Title>Egyptian propolis</Title>
<Subcolumn>
<d>2562.5</d>
<d>2568.423708</d>
<d>2570.221566</d>
<d>2568.076364</d>
<d>2567.910337</d>
<d>2568.139663</d>
<d>2566.29759</d>
<d>2565.57201</d>
<d>2563.243375</d>
<d>2561.800141</d>
<d>2563.508264</d>
<d>2564.648063</d>
<d>2564.402454</d>
<d>2564.458367</d>
<d>2567.011324</d>
<d>2567.568488</d>
<d>2568.831733</d>
<d>2567.767796</d>
<d>2566.862025</d>
<d>2565.542238</d>
<d>2566.626535</d>
<d>2566.228859</d>
<d>2565.303675</d>
<d>2566.784071</d>
<d>2565.358507</d>
<d>2562.658538</d>
<d>2564.40499</d>
<d>2564.750105</d>
<d>2562.979465</d>
<d>2560.837617</d>
<d>2562.160508</d>
<d>2560.721419</d>
<d>2561.583218</d>
<d>2564.111289</d>
<d>2563.278692</d>
<d>2564.530988</d>
<d>2566.347253</d>
<d>2566.457052</d>
<d>2564.713916</d>
<d>2564.659452</d>
<d>2564.321261</d>
<d>2561.68812</d>
<d>2562.70883</d>
<d>2563.29412</d>
<d>2561.618556</d>
<d>2561.612054</d>
<d>2561.083708</d>
<d>2562.654815</d>
<d>2563.9076</d>
<d>2564.492011</d>
<d>2563.194243</d>
<d>2563.017444</d>
<d>2564.34589</d>
<d>2562.44819</d>
<d>2561.264784</d>
<d>2562.912422</d>
<d>2564.215358</d>
<d>2565.072295</d>
<d>2565.267328</d>
<d>2565.044112</d>
<d>2562.79698</d>
<d>2562.011911</d>
<d>2561.717889</d>
<d>2561.610155</d>
<d>2563.024964</d>
<d>2565.001003</d>
<d>2564.671639</d>
<d>2562.901616</d>
<d>2562.213363</d>
<d>2563.588972</d>
<d>2562.52951</d>
<d>2560.989205</d>
<d>2560.852917</d>
<d>2562.212266</d>
<d>2563.570427</d>
<d>2563.289163</d>
<d>2562.60222</d>
<d>2561.320321</d>
<d>2562.28285</d>
<d>2564.094817</d>
<d>2563.927914</d>
<d>2562.505646</d>
<d>2562.691162</d>
<d>2562.698726</d>
<d>2564.41674</d>
<d>2565.160625</d>
<d>2565.627627</d>
<d>2564.161058</d>
<d>2565.955354</d>
<d>2565.726355</d>
<d>2565.817918</d>
<d>2563.990094</d>
<d>2561.88601</d>
<d>2560.841671</d>
<d>2561.257279</d>
<d>2563.817637</d>
<d>2564.257636</d>
<d>2564.007731</d>
<d>2565.768428</d>
<d>2566.692007</d>
<d>2565.465609</d>
<d>2564.905347</d>
<d>2562.971997</d>
<d>2561.556324</d>
<d>2561.001164</d>
<d>2562.070521</d>
<d>2562.176912</d>
<d>2562.286507</d>
<d>2562.674352</d>
<d>2564.787331</d>
<d>2562.803196</d>
<d>2563.548064</d>
<d>2562.296891</d>
<d>2561.492835</d>
<d>2562.762349</d>
<d>2563.979348</d>
<d>2564.56742</d>
<d>2563.536881</d>
<d>2564.074305</d>
<d>2566.043678</d>
<d>2565.357718</d>
<d>2565.167532</d>
<d>2565.472002</d>
<d>2564.715608</d>
<d>2565.609432</d>
<d>2565.679454</d>
<d>2562.075215</d>
<d>2560.001608</d>
<d>2561.135853</d>
<d>2561.441656</d>
<d>2562.403592</d>
<d>2562.5</d>
<d>2563.533628</d>
<d>2563.43226</d>
<d>2565.270971</d>
<d>2566.212618</d>
<d>2565.859363</d>
<d>2566.52955</d>
<d>2568.162702</d>
<d>2567.345263</d>
<d>2567.187525</d>
<d>2565.21136</d>
<d>2563.405965</d>
<d>2562.401139</d>
<d>2562.212935</d>
<d>2562.500758</d>
<d>2564.057672</d>
<d>2564.558614</d>
<d>2564.105002</d>
<d>2563.177181</d>
<d>2562.992937</d>
<d>2563.414014</d>
<d>2561.259315</d>
<d>2561.860412</d>
<d>2560</d>
<d>2563.809762</d>
<d>2637.234839</d>
<d>2627.341219</d>
<d>2585.994881</d>
<d>2591.479705</d>
<d>2582.916912</d>
<d>2596.170355</d>
<d>2551.477125</d>
<d>2578.852459</d>
<d>2557.873443</d>
<d>2560</d>
<d>2571.753102</d>
<d>2576.401835</d>
<d>2610.323958</d>
<d>2648.333089</d>
<d>2611.962191</d>
<d>2626.079362</d>
<d>2663.137186</d>
<d>2615.454587</d>
<d>2601.066378</d>
<d>2638.950555</d>
<d>2714.681999</d>
<d>2768.799155</d>
<d>2822.871432</d>
<d>2818.766296</d>
<d>2805.296498</d>
<d>2789.410274</d>
<d>2807.236201</d>
<d>2715.080672</d>
<d>2735.362496</d>
<d>2742.684268</d>
<d>2715.221824</d>
<d>2721.416083</d>
<d>2748.166965</d>
<d>2700.145387</d>
<d>2706.060966</d>
<d>2664.608528</d>
<d>2606.744485</d>
<d>2593.657609</d>
<d>2625.999926</d>
<d>2669.163028</d>
<d>2662.349604</d>
<d>2672.02598</d>
<d>2644.495532</d>
<d>2643.051052</d>
<d>2637.532202</d>
<d>2579.022417</d>
<d>2567.466931</d>
<d>2614.397912</d>
<d>2635.714368</d>
<d>2633.656353</d>
<d>2667.156607</d>
<d>2707.710811</d>
<d>2673.276324</d>
<d>2708.749403</d>
<d>2676.552297</d>
<d>2569.696798</d>
<d>2608.685418</d>
<d>2606.251885</d>
<d>2621.556169</d>
<d>2615.47144</d>
<d>2656.61008</d>
<d>2673.67092</d>
<d>2709.606122</d>
<d>2691.539729</d>
<d>2667.874179</d>
<d>2613.449189</d>
<d>2671.29451</d>
<d>2668.012065</d>
<d>2684.398853</d>
<d>2640.461926</d>
<d>2570.981726</d>
<d>2548.879158</d>
<d>2585.018373</d>
<d>2570.546076</d>
<d>2564.863006</d>
<d>2537.240814</d>
<d>2573.244557</d>
<d>2671.858242</d>
<d>2768.845115</d>
<d>2803.348461</d>
<d>2710.639076</d>
<d>2801.033461</d>
<d>2913.553319</d>
<d>2966.113204</d>
<d>2937.415082</d>
<d>2826.625172</d>
<d>2767.161911</d>
<d>2701.374955</d>
<d>2580.78148</d>
<d>2515.191051</d>
<d>2502.657382</d>
<d>2512.115861</d>
<d>2546.287822</d>
<d>2602.4577</d>
<d>2562.780811</d>
<d>2519.293821</d>
<d>2506.074748</d>
<d>2463.298204</d>
<d>2435.359863</d>
<d>2477.47569</d>
<d>2523.943351</d>
<d>2492.950681</d>
<d>2475.443041</d>
<d>2511.270609</d>
<d>2452.496796</d>
<d>2449.63955</d>
<d>2491.162743</d>
<d>2524.421953</d>
<d>2544.571021</d>
<d>2577.260166</d>
<d>2568.359061</d>
<d>2564.411994</d>
<d>2519.92169</d>
<d>2494.78465</d>
<d>2476.840349</d>
<d>2416.452971</d>
<d>2395.593764</d>
<d>2435.975785</d>
<d>2514.225997</d>
<d>2738.072059</d>
<d>3121.82182</d>
<d>3610.547985</d>
<d>4081.30651</d>
<d>4569.366395</d>
<d>4901.527655</d>
<d>4890.693566</d>
<d>4695.470502</d>
<d>4295.433921</d>
<d>3786.118252</d>
<d>3323.490803</d>
<d>2950.493484</d>
<d>2632.000103</d>
<d>2488.873506</d>
<d>2483.61936</d>
<d>2460.979621</d>
<d>2464.14814</d>
<d>2471.175804</d>
<d>2418.891823</d>
<d>2442.676291</d>
<d>2450.285849</d>
<d>2431.673014</d>
<d>2444.857277</d>
<d>2518.294419</d>
<d>2558.804481</d>
<d>2535.498801</d>
<d>2570.808834</d>
<d>2587.446307</d>
<d>2594.937087</d>
<d>2579.403543</d>
<d>2553.988478</d>
<d>2571.859064</d>
<d>2612.423045</d>
<d>2637.119325</d>
<d>2609.315386</d>
<d>2546.806552</d>
<d>2550.528239</d>
<d>2508.579974</d>
<d>2458.619785</d>
<d>2403.883869</d>
<d>2424.047658</d>
<d>2472.380294</d>
<d>2519.314247</d>
<d>2599.640644</d>
<d>2624.078204</d>
<d>2618.802126</d>
<d>2629.682794</d>
<d>2631.949878</d>
<d>2672.495433</d>
<d>2792.524403</d>
<d>2883.062108</d>
<d>3012.843528</d>
<d>3201.093417</d>
<d>3301.928952</d>
<d>3262.022818</d>
<d>3190.863182</d>
<d>3093.370571</d>
<d>2964.75684</d>
<d>2796.590208</d>
<d>2708.105354</d>
<d>2593.799386</d>
<d>2579.935171</d>
<d>2569.222078</d>
<d>2519.541068</d>
<d>2493.043678</d>
<d>2508.074564</d>
<d>2511.157356</d>
<d>2481.543221</d>
<d>2471.920602</d>
<d>2490.907125</d>
<d>2497.390341</d>
<d>2504.63272</d>
<d>2493.327405</d>
<d>2503.923169</d>
<d>2514.836389</d>
<d>2526.862769</d>
<d>2521.845258</d>
<d>2487.017947</d>
<d>2508.772343</d>
<d>2523.400812</d>
<d>2533.602712</d>
<d>2560.698922</d>
<d>2574.630761</d>
<d>2567.756947</d>
<d>2530.757181</d>
<d>2508.313958</d>
<d>2518.089337</d>
<d>2506.24041</d>
<d>2530.626748</d>
<d>2582.362084</d>
<d>2607.592086</d>
<d>2662.638175</d>
<d>2670.531879</d>
<d>2522.946689</d>
<d>2517.068383</d>
<d>2517.213955</d>
<d>2525.019181</d>
<d>2516.576911</d>
<d>2517.505535</d>
<d>2513.431118</d>
<d>2515.53353</d>
<d>2511.371045</d>
<d>2512.009993</d>
<d>2506.825955</d>
<d>2511.689262</d>
<d>2519.329322</d>
<d>2534.758173</d>
<d>2537.008446</d>
<d>2548.576542</d>
<d>2538.61378</d>
<d>2539.612773</d>
<d>2524.70483</d>
<d>2526.89927</d>
<d>2517.336462</d>
<d>2518.903497</d>
<d>2515.07527</d>
<d>2526.141153</d>
<d>2528.602346</d>
<d>2528.354374</d>
<d>2512.609693</d>
<d>2501.460821</d>
<d>2493.77312</d>
<d>2498.320388</d>
<d>2493.166369</d>
<d>2497.212838</d>
<d>2490.248258</d>
<d>2501.121546</d>
<d>2499.840368</d>
<d>2498.978673</d>
<d>2499.234295</d>
<d>2506.870487</d>
<d>2519.424254</d>
<d>2527.257156</d>
<d>2529.148256</d>
<d>2534.09511</d>
<d>2531.446838</d>
<d>2537.043882</d>
<d>2536.931102</d>
<d>2533.087577</d>
<d>2531.524022</d>
<d>2526.220046</d>
<d>2524.46516</d>
<d>2520.536284</d>
<d>2518.62188</d>
<d>2521.682122</d>
<d>2521.521788</d>
<d>2526.050079</d>
<d>2526.061428</d>
<d>2515.781174</d>
<d>2511.583625</d>
<d>2503.463204</d>
<d>2508.472001</d>
<d>2511.530471</d>
<d>2512.486389</d>
<d>2521.333962</d>
<d>2522.91536</d>
<d>2528.953639</d>
<d>2521.312355</d>
<d>2512.323648</d>
<d>2510.679558</d>
<d>2502.092275</d>
<d>2494.522188</d>
<d>2494.794233</d>
<d>2489.906072</d>
<d>2500.926585</d>
<d>2503.582992</d>
<d>2518.096898</d>
<d>2521.460605</d>
<d>2525.596295</d>
<d>2532.768415</d>
<d>2535.096878</d>
<d>2536.034322</d>
<d>2543.236246</d>
<d>2533.652745</d>
<d>2534.742553</d>
<d>2527.610566</d>
<d>2521.380711</d>
<d>2512.59854</d>
<d>2505.701598</d>
<d>2508.927256</d>
<d>2518.521238</d>
<d>2517.686535</d>
<d>2508.549115</d>
<d>2509.115482</d>
<d>2509.201095</d>
<d>2509.80265</d>
<d>2513.933352</d>
<d>2516.653902</d>
<d>2514.710411</d>
<d>2517.186695</d>
<d>2519.881797</d>
<d>2515.815432</d>
<d>2507.452822</d>
<d>2506.403522</d>
<d>2499.638388</d>
<d>2499.675966</d>
<d>2505.202732</d>
<d>2502.203042</d>
<d>2504.540234</d>
<d>2504.127442</d>
<d>2511.778584</d>
<d>2521.387111</d>
<d>2520.248873</d>
<d>2515.721412</d>
<d>2511.184934</d>
<d>2502.803383</d>
<d>2495.822432</d>
<d>2491.071239</d>
<d>2496.92146</d>
<d>2497.220919</d>
<d>2502.686777</d>
<d>2513.8795</d>
<d>2522.417805</d>
<d>2531.6326</d>
<d>2531.435459</d>
<d>2527.190022</d>
<d>2516.781833</d>
<d>2510.743473</d>
<d>2501.379368</d>
<d>2489.876511</d>
<d>2492.429453</d>
<d>2499.587141</d>
<d>2499.091647</d>
<d>2503.446706</d>
<d>2505.711958</d>
<d>2512.224963</d>
<d>2511.916363</d>
<d>2509.303536</d>
<d>2508.319719</d>
<d>2506.629516</d>
<d>2518.095632</d>
<d>2522.753473</d>
<d>2512.356148</d>
<d>2509.228732</d>
<d>2510.24176</d>
<d>2514.352981</d>
<d>2506.424284</d>
<d>2508.344826</d>
<d>2505.348599</d>
<d>2498.801223</d>
<d>2497.022094</d>
<d>2495.410043</d>
<d>2491.3112</d>
<d>2497.853359</d>
<d>2504.727542</d>
<d>2514.114144</d>
<d>2516.527062</d>
<d>2528.357203</d>
<d>2524.145382</d>
<d>2522.265067</d>
<d>2514.617168</d>
<d>2508.453829</d>
<d>2510.087303</d>
<d>2510.054813</d>
<d>2512.655514</d>
<d>2516.609968</d>
<d>2513.654374</d>
<d>2510.274151</d>
<d>2505.8405</d>
<d>2501.239381</d>
<d>2496.058196</d>
<d>2492.351609</d>
<d>2492.721586</d>
<d>2496.425105</d>
<d>2511.875025</d>
<d>2521.169621</d>
<d>2517.60419</d>
<d>2514.456883</d>
<d>2509.606487</d>
<d>2507.147893</d>
<d>2500.64336</d>
<d>2497.048694</d>
<d>2497.809991</d>
<d>2500.126844</d>
<d>2508.887559</d>
<d>2511.37846</d>
<d>2504.937835</d>
<d>2502.352105</d>
<d>2498.800951</d>
<d>2500.634412</d>
<d>2497.958939</d>
<d>2498.300073</d>
<d>2496.779905</d>
<d>2500.858077</d>
<d>2504.185666</d>
<d>2500.750533</d>
<d>2500.051898</d>
<d>2501.086267</d>
<d>2501.00496</d>
<d>2507.99436</d>
<d>2510.751533</d>
<d>2513.87652</d>
<d>2515.986452</d>
<d>2524.998481</d>
<d>2523.08015</d>
<d>2522.709896</d>
<d>2529.143157</d>
<d>2526.097592</d>
<d>2524.880213</d>
<d>2527.750246</d>
<d>2526.462839</d>
<d>2521.011194</d>
<d>2517.1389</d>
<d>2515.284477</d>
<d>2506.423256</d>
<d>2506.951797</d>
<d>2507.496656</d>
<d>2504.813464</d>
<d>2509.113276</d>
<d>2510.779249</d>
<d>2513.609849</d>
<d>2508.0739</d>
<d>2502.253082</d>
<d>2502.697999</d>
<d>2503.662047</d>
<d>2504.733044</d>
<d>2502.781234</d>
<d>2503.908691</d>
<d>2505.549502</d>
<d>2502.056909</d>
<d>2506.455832</d>
<d>2497.211867</d>
<d>2494.458066</d>
<d>2497.548758</d>
<d>2501.329224</d>
<d>2499.238015</d>
<d>2505.411406</d>
<d>2504.482756</d>
<d>2502.811003</d>
<d>2495.575826</d>
<d>2502.320253</d>
<d>2499.202404</d>
<d>2501.098441</d>
<d>2494.959863</d>
<d>2497.635872</d>
<d>2499.139775</d>
<d>2510.297913</d>
<d>2503.30881</d>
<d>2503.74191</d>
<d>2506.857799</d>
<d>2514.598882</d>
<d>2513.851037</d>
<d>2504.623783</d>
<d>2508.399152</d>
<d>2518.641461</d>
<d>2522.486678</d>
<d>2516.273922</d>
<d>2507.566041</d>
<d>2503.109778</d>
<d>2498.205297</d>
<d>2498.672327</d>
<d>2496.65614</d>
<d>2493.323868</d>
<d>2502.310126</d>
<d>2507.545023</d>
<d>2515.327887</d>
<d>2511.851365</d>
<d>2514.050687</d>
<d>2512.817058</d>
<d>2514.331384</d>
<d>2523.673834</d>
<d>2521.100939</d>
<d>2518.569099</d>
<d>2511.881534</d>
<d>2504.401674</d>
<d>2511.507021</d>
<d>2513.52476</d>
<d>2517.254386</d>
<d>2525.335231</d>
<d>2535.485682</d>
<d>2538.237833</d>
<d>2534.856294</d>
<d>2533.693373</d>
<d>2526.358078</d>
<d>2520.408616</d>
<d>2522.683205</d>
<d>2518.85621</d>
<d>2513.062658</d>
<d>2508.160088</d>
<d>2507.059067</d>
<d>2504.696579</d>
<d>2507.341775</d>
<d>2508.964173</d>
<d>2513.447238</d>
<d>2520.30759</d>
<d>2527.437629</d>
<d>2527.64972</d>
<d>2529.982141</d>
<d>2524.310874</d>
<d>2525.035784</d>
<d>2524.757198</d>
<d>2524.689374</d>
<d>2521.710321</d>
<d>2521.106755</d>
<d>2516.301556</d>
<d>2516.115433</d>
<d>2514.714372</d>
<d>2517.344294</d>
<d>2509.29269</d>
<d>2501.53099</d>
<d>2501.100391</d>
<d>2502.657456</d>
<d>2503.737031</d>
<d>2503.886633</d>
<d>2504.491387</d>
<d>2505.045029</d>
<d>2504.674526</d>
<d>2505.446942</d>
<d>2504.304416</d>
<d>2502.803255</d>
<d>2502.589731</d>
<d>2503.482704</d>
<d>2502.833573</d>
<d>2502.092659</d>
<d>2501.792936</d>
<d>2500.908884</d>
<d>2501.233432</d>
<d>2503.652847</d>
<d>2503.88804</d>
<d>2504.542814</d>
<d>2505.378119</d>
<d>2505.583932</d>
<d>2506.64396</d>
<d>2506.921767</d>
<d>2506.036622</d>
<d>2504.858766</d>
<d>2504.107047</d>
<d>2504.068833</d>
<d>2503.115458</d>
<d>2503.666308</d>
<d>2504.037476</d>
<d>2504.28566</d>
<d>2506.340128</d>
<d>2506.763986</d>
<d>2507.431872</d>
<d>2506.786262</d>
<d>2507.109232</d>
<d>2506.805381</d>
<d>2505.541084</d>
<d>2504.859295</d>
<d>2503.726845</d>
<d>2502.538185</d>
<d>2503.035859</d>
<d>2502.935485</d>
<d>2505.075908</d>
<d>2506.318531</d>
<d>2507.334246</d>
<d>2507.794648</d>
<d>2507.706293</d>
<d>2507.213534</d>
<d>2506.858085</d>
<d>2505.899611</d>
<d>2504.97853</d>
<d>2503.507842</d>
<d>2503.36619</d>
<d>2502.202539</d>
<d>2501.558993</d>
<d>2502.37718</d>
<d>2503.305603</d>
<d>2504.465549</d>
<d>2506.086231</d>
<d>2506.025679</d>
<d>2505.874314</d>
<d>2504.517627</d>
<d>2504.116044</d>
<d>2503.359711</d>
<d>2502.801271</d>
<d>2501.577403</d>
<d>2501.288849</d>
<d>2501.311945</d>
<d>2501.907958</d>
<d>2501.945259</d>
<d>2501.820968</d>
<d>2501.6055</d>
<d>2503.313474</d>
<d>2503.727499</d>
<d>2503.801829</d>
<d>2504.046249</d>
<d>2504.973094</d>
<d>2503.704912</d>
<d>2503.125833</d>
<d>2502.414057</d>
<d>2501.414183</d>
<d>2500.836561</d>
<d>2500.92036</d>
<d>2500.019554</d>
<d>2500.199264</d>
<d>2500.68589</d>
<d>2500.727076</d>
<d>2500.974288</d>
<d>2501.472205</d>
<d>2501.478669</d>
<d>2502.271117</d>
<d>2501.891266</d>
<d>2502.766635</d>
<d>2502.675488</d>
<d>2503.544543</d>
<d>2504.16156</d>
<d>2503.988986</d>
<d>2503.667546</d>
<d>2503.041476</d>
<d>2502.528293</d>
<d>2501.986314</d>
<d>2500.465657</d>
<d>2500.197891</d>
<d>2499.840626</d>
<d>2499.789039</d>
<d>2500.65162</d>
<d>2499.512187</d>
<d>2499.132127</d>
<d>2500.575567</d>
<d>2501.544891</d>
<d>2501.131494</d>
<d>2501.119928</d>
<d>2500.511626</d>
<d>2499.950161</d>
<d>2499.424365</d>
<d>2500.221631</d>
<d>2499.954623</d>
<d>2501.322811</d>
<d>2502.898822</d>
<d>2504.38108</d>
<d>2505.050861</d>
<d>2505.193104</d>
<d>2504.37876</d>
<d>2502.844085</d>
<d>2502.864233</d>
<d>2502.217347</d>
<d>2501.233895</d>
<d>2500.699739</d>
<d>2500.687602</d>
<d>2499.98342</d>
<d>2499.903602</d>
<d>2499.615664</d>
<d>2499.490138</d>
<d>2499.879344</d>
<d>2500.776242</d>
<d>2500.615539</d>
<d>2500.197544</d>
<d>2500.845708</d>
<d>2501.383847</d>
<d>2500.811086</d>
<d>2500.841561</d>
<d>2501.328976</d>
<d>2500.248882</d>
<d>2501.18507</d>
<d>2501.442564</d>
<d>2501.585416</d>
<d>2500.99336</d>
<d>2501.400931</d>
<d>2501.723845</d>
<d>2500.754839</d>
<d>2500.885132</d>
<d>2500.880282</d>
<d>2499.844327</d>
<d>2500.819104</d>
<d>2501.984247</d>
<d>2501.289201</d>
<d>2501.346257</d>
<d>2501.278316</d>
<d>2501.794353</d>
<d>2502.11198</d>
<d>2501.821039</d>
<d>2501.16903</d>
<d>2499.9835</d>
<d>2500.378199</d>
<d>2501.140458</d>
<d>2500.425925</d>
<d>2501.661224</d>
<d>2502.198767</d>
<d>2503.579724</d>
<d>2503.444957</d>
<d>2501.055365</d>
<d>2500.221058</d>
<d>2498.831512</d>
<d>2499.442688</d>
<d>2499.449307</d>
<d>2499.882805</d>
<d>2501.405043</d>
<d>2502.472273</d>
<d>2503.085989</d>
<d>2502.365647</d>
<d>2501.055823</d>
<d>2500.928163</d>
<d>2501.459455</d>
<d>2501.46916</d>
<d>2500.874571</d>
<d>2501.506502</d>
<d>2501.530429</d>
<d>2501.464881</d>
<d>2501.518674</d>
<d>2501.537017</d>
<d>2501.714159</d>
<d>2502.159488</d>
<d>2502.751476</d>
<d>2502.566828</d>
<d>2502.82592</d>
<d>2502.518044</d>
<d>2501.956793</d>
<d>2501.895736</d>
<d>2501.740346</d>
<d>2500.641629</d>
<d>2501.479343</d>
<d>2500.756743</d>
<d>2500.85655</d>
<d>2501.414301</d>
<d>2502.118882</d>
<d>2501.899217</d>
<d>2502.466059</d>
<d>2502.376148</d>
<d>2501.902664</d>
<d>2501.152704</d>
<d>2501.239945</d>
<d>2500.662362</d>
<d>2500.864585</d>
<d>2501.78289</d>
<d>2502.823251</d>
<d>2502.583924</d>
<d>2502.390559</d>
<d>2502.356198</d>
<d>2501.477917</d>
<d>2501.087677</d>
<d>2500.553814</d>
<d>2500.78701</d>
<d>2500.465647</d>
<d>2500.80987</d>
<d>2500.564228</d>
<d>2499.365484</d>
<d>2498.872424</d>
<d>2499.369393</d>
<d>2500.360942</d>
<d>2501.257896</d>
<d>2501.110018</d>
<d>2501.927685</d>
<d>2501.551694</d>
<d>2501.133717</d>
<d>2500.099024</d>
<d>2500.384057</d>
<d>2500.042922</d>
<d>2500.148375</d>
<d>2501.449624</d>
<d>2501.348701</d>
<d>2501.39816</d>
<d>2502.437412</d>
<d>2501.041349</d>
<d>2500.341291</d>
<d>2499.599337</d>
<d>2500.518221</d>
<d>2500.142917</d>
<d>2500.319053</d>
<d>2501.08682</d>
<d>2501.025651</d>
<d>2502.316859</d>
<d>2503.311146</d>
<d>2502.279934</d>
<d>2501.956789</d>
<d>2500.989704</d>
<d>2501.321182</d>
<d>2501.11483</d>
<d>2501.377349</d>
<d>2500.911188</d>
<d>2500.721104</d>
<d>2501.673454</d>
<d>2501.936702</d>
<d>2501.565844</d>
<d>2501.72104</d>
<d>2501.398259</d>
<d>2502.198973</d>
<d>2502.042519</d>
<d>2502.271408</d>
<d>2502.00581</d>
<d>2502.287511</d>
<d>2501.98197</d>
<d>2501.343775</d>
<d>2501.175059</d>
<d>2501.505202</d>
<d>2501.436889</d>
<d>2500.530629</d>
<d>2499.94391</d>
<d>2499.866613</d>
<d>2500.978734</d>
<d>2501.761358</d>
<d>2502.244594</d>
<d>2502.760731</d>
<d>2502.57174</d>
<d>2503.405962</d>
<d>2503.19706</d>
<d>2502.102742</d>
<d>2502.17951</d>
<d>2501.421366</d>
<d>2502.258501</d>
<d>2501.328203</d>
<d>2500.35007</d>
<d>2500.087143</d>
<d>2499.816688</d>
<d>2500.212511</d>
<d>2500.794451</d>
<d>2502.716352</d>
<d>2503.578193</d>
<d>2503.532967</d>
<d>2503.774952</d>
<d>2503.806385</d>
<d>2503.139336</d>
<d>2503.151405</d>
<d>2502.753579</d>
<d>2501.678646</d>
<d>2501.930298</d>
<d>2502.093912</d>
<d>2502.226913</d>
<d>2501.937405</d>
<d>2501.409441</d>
<d>2502.53927</d>
<d>2502.626938</d>
<d>2502.03266</d>
<d>2501.411302</d>
<d>2500.98067</d>
<d>2500.989529</d>
<d>2499.407721</d>
<d>2499.895773</d>
<d>2500.319605</d>
<d>2500.795161</d>
<d>2502.123848</d>
<d>2502.443413</d>
<d>2501.574786</d>
<d>2502.079033</d>
<d>2501.827662</d>
<d>2501.782089</d>
<d>2500.419153</d>
<d>2500.642531</d>
<d>2501.09915</d>
<d>2501.826527</d>
<d>2502.145029</d>
<d>2502.083311</d>
<d>2501.3795</d>
<d>2501.4617</d>
<d>2502.151082</d>
<d>2502.548996</d>
<d>2502.018303</d>
<d>2501.930047</d>
<d>2501.853964</d>
<d>2502.214007</d>
<d>2500.394598</d>
<d>2499.91543</d>
<d>2498.846144</d>
<d>2498.723622</d>
<d>2501.711208</d>
<d>2501.732269</d>
<d>2501.916057</d>
<d>2502.207818</d>
<d>2502.58218</d>
<d>2501.931187</d>
<d>2500.337885</d>
<d>2500.048533</d>
<d>2498.980529</d>
<d>2499.31336</d>
<d>2500.207381</d>
<d>2500.79308</d>
<d>2500.84355</d>
<d>2501.333199</d>
<d>2502.728106</d>
<d>2502.378337</d>
<d>2503.520707</d>
<d>2504.008994</d>
<d>2504.060761</d>
<d>2504.271384</d>
<d>2503.45351</d>
<d>2504.077639</d>
<d>2502.725088</d>
<d>2502.365095</d>
<d>2502.174645</d>
<d>2501.46476</d>
<d>2502.538517</d>
<d>2502.46007</d>
<d>2502.873382</d>
<d>2503.458549</d>
<d>2504.380197</d>
<d>2504.503786</d>
<d>2504.109885</d>
<d>2503.050987</d>
<d>2502.731158</d>
<d>2502.327019</d>
<d>2501.986201</d>
<d>2501.628942</d>
<d>2501.789308</d>
<d>2503.078216</d>
<d>2504.006497</d>
<d>2503.998929</d>
<d>2504.093413</d>
<d>2503.733061</d>
<d>2504.556979</d>
<d>2504.399158</d>
<d>2505.656039</d>
<d>2506.573663</d>
<d>2506.152372</d>
<d>2506.066724</d>
<d>2505.040859</d>
<d>2505.042898</d>
<d>2504.700584</d>
<d>2503.953701</d>
<d>2504.512378</d>
<d>2503.679453</d>
<d>2504.807562</d>
<d>2504.035863</d>
<d>2502.744867</d>
<d>2502.050057</d>
<d>2501.899835</d>
<d>2502.491244</d>
<d>2502.858862</d>
<d>2502.647697</d>
<d>2503.566664</d>
<d>2503.169426</d>
<d>2503.687433</d>
<d>2502.779605</d>
<d>2503.049018</d>
<d>2503.448056</d>
<d>2504.035115</d>
<d>2504.084187</d>
<d>2503.823758</d>
<d>2503.433509</d>
<d>2502.290264</d>
<d>2501.600315</d>
<d>2501.307087</d>
<d>2501.927529</d>
<d>2503.285603</d>
<d>2504.094279</d>
<d>2505.430798</d>
<d>2504.522358</d>
<d>2503.909631</d>
<d>2502.670415</d>
<d>2500.999447</d>
<d>2500.029811</d>
<d>2501.096978</d>
<d>2503.005218</d>
<d>2504.183532</d>
<d>2505.439394</d>
<d>2505.271615</d>
<d>2504.389223</d>
<d>2502.773537</d>
<d>2501.454325</d>
<d>2500.990664</d>
<d>2500.522267</d>
<d>2501.940648</d>
<d>2503.321999</d>
<d>2504.144274</d>
<d>2504.511396</d>
<d>2503.55094</d>
<d>2504.065571</d>
<d>2502.383597</d>
<d>2502.174403</d>
<d>2502.255371</d>
<d>2502.850737</d>
<d>2503.380666</d>
<d>2504.015003</d>
<d>2504.39888</d>
<d>2504.121699</d>
<d>2503.625136</d>
<d>2502.824329</d>
<d>2502.099766</d>
<d>2501.487503</d>
<d>2502.210953</d>
<d>2502.044737</d>
<d>2501.741697</d>
<d>2501.991176</d>
<d>2502.36012</d>
<d>2502.262499</d>
<d>2501.231627</d>
<d>2501.321579</d>
<d>2501.16204</d>
<d>2500.778507</d>
<d>2501.650559</d>
<d>2500.591642</d>
<d>2500.767913</d>
<d>2500.744957</d>
<d>2501.012041</d>
<d>2501.368965</d>
<d>2501.523505</d>
<d>2502.935693</d>
<d>2502.26467</d>
<d>2502.142039</d>
<d>2503.088513</d>
<d>2502.809268</d>
<d>2503.311273</d>
<d>2503.749418</d>
<d>2503.771867</d>
<d>2503.931749</d>
<d>2504.280782</d>
<d>2504.595093</d>
<d>2504.112613</d>
<d>2503.67384</d>
<d>2503.903895</d>
<d>2503.702107</d>
<d>2503.517073</d>
<d>2503.834419</d>
<d>2503.933112</d>
<d>2504.36713</d>
<d>2503.976798</d>
<d>2504.70389</d>
<d>2504.281385</d>
<d>2504.044875</d>
<d>2503.214202</d>
<d>2503.615197</d>
<d>2503.428828</d>
<d>2504.042527</d>
<d>2504.766463</d>
<d>2504.391914</d>
<d>2504.110483</d>
<d>2504.856879</d>
<d>2503.522855</d>
<d>2502.256091</d>
<d>2501.98668</d>
<d>2503.479361</d>
<d>2503.304282</d>
<d>2502.691534</d>
<d>2503.032068</d>
<d>2502.976575</d>
<d>2503.843892</d>
<d>2504.040924</d>
<d>2503.692113</d>
<d>2503.139704</d>
<d>2504.779408</d>
<d>2505.406218</d>
<d>2505.682772</d>
<d>2505.386894</d>
<d>2506.298767</d>
<d>2505.862057</d>
<d>2505.973741</d>
<d>2505.480578</d>
<d>2505.17001</d>
<d>2504.093975</d>
<d>2503.558805</d>
<d>2502.966939</d>
<d>2502.327481</d>
<d>2502.202543</d>
<d>2502.375658</d>
<d>2503.013031</d>
<d>2503.919926</d>
<d>2504.119751</d>
<d>2504.509091</d>
<d>2503.842802</d>
<d>2503.995764</d>
<d>2503.692237</d>
<d>2501.72338</d>
<d>2502.402638</d>
<d>2502.998005</d>
<d>2503.48022</d>
<d>2503.095109</d>
<d>2502.269049</d>
<d>2502.420068</d>
<d>2501.946937</d>
<d>2503.321126</d>
<d>2502.505629</d>
<d>2501.955283</d>
<d>2502.460445</d>
<d>2502.267277</d>
<d>2501.211395</d>
<d>2501.029639</d>
<d>2500.940395</d>
<d>2500.85965</d>
<d>2500.554666</d>
<d>2501.970376</d>
<d>2502.432681</d>
<d>2503.943208</d>
<d>2503.907383</d>
<d>2504.418129</d>
<d>2504.205415</d>
<d>2504.2689</d>
<d>2503.247255</d>
<d>2501.96665</d>
<d>2500.663221</d>
<d>2500.616105</d>
<d>2500.496145</d>
<d>2500.980424</d>
<d>2501.067705</d>
<d>2501.589014</d>
<d>2502.048283</d>
<d>2502.7982</d>
<d>2502.827206</d>
<d>2503.005784</d>
<d>2502.429458</d>
<d>2502.497678</d>
<d>2502.516728</d>
<d>2502.625619</d>
<d>2502.719577</d>
<d>2502.727337</d>
<d>2503.530429</d>
<d>2503.911271</d>
<d>2502.809498</d>
<d>2501.647507</d>
<d>2501.043743</d>
<d>2500.914313</d>
<d>2501.566619</d>
<d>2501.713632</d>
<d>2501.736688</d>
<d>2501.574479</d>
<d>2502.426377</d>
<d>2501.48571</d>
<d>2501.062234</d>
<d>2500.531435</d>
<d>2500.752384</d>
<d>2500.828374</d>
<d>2501.233011</d>
<d>2501.018597</d>
<d>2501.615421</d>
<d>2502.048345</d>
<d>2502.221339</d>
<d>2502.625612</d>
<d>2503.630846</d>
<d>2503.897565</d>
<d>2504.363391</d>
<d>2504.308019</d>
<d>2504.540866</d>
<d>2503.492026</d>
<d>2502.941559</d>
<d>2501.89102</d>
<d>2500.625955</d>
<d>2500.866086</d>
<d>2500.571665</d>
<d>2500.650205</d>
<d>2501.459489</d>
<d>2502.3228</d>
<d>2503.315408</d>
<d>2503.738134</d>
<d>2503.560142</d>
<d>2503.683602</d>
<d>2503.459354</d>
<d>2503.496372</d>
<d>2503.436023</d>
<d>2502.406851</d>
<d>2502.765353</d>
<d>2501.799312</d>
<d>2501.42537</d>
<d>2501.480478</d>
<d>2501.435305</d>
<d>2500.900742</d>
<d>2501.247468</d>
<d>2501.691077</d>
<d>2502.261312</d>
<d>2501.841478</d>
<d>2502.929876</d>
<d>2502.230201</d>
<d>2502.771663</d>
<d>2503.032797</d>
<d>2503.745321</d>
<d>2503.925842</d>
<d>2504.084024</d>
<d>2504.22533</d>
<d>2504.559066</d>
<d>2503.690636</d>
<d>2504.009681</d>
<d>2502.744252</d>
<d>2502.137089</d>
<d>2501.725511</d>
<d>2502.258231</d>
<d>2503.205191</d>
<d>2502.310051</d>
<d>2502.662848</d>
<d>2502.942315</d>
<d>2503.106688</d>
<d>2504.202856</d>
<d>2504.395153</d>
<d>2504.798091</d>
<d>2505.686775</d>
<d>2506.482219</d>
<d>2506.662133</d>
<d>2505.418071</d>
<d>2505.02604</d>
<d>2504.364729</d>
<d>2503.54049</d>
<d>2503.174561</d>
<d>2502.736638</d>
<d>2502.935734</d>
<d>2502.845883</d>
<d>2502.251831</d>
<d>2502.548295</d>
<d>2503.027718</d>
<d>2503.472419</d>
<d>2503.579854</d>
<d>2504.389381</d>
<d>2504.837481</d>
<d>2503.937728</d>
<d>2504.605574</d>
<d>2504.458419</d>
<d>2503.966228</d>
<d>2504.388711</d>
<d>2504.573603</d>
<d>2504.130216</d>
<d>2503.318361</d>
<d>2503.019084</d>
<d>2502.926441</d>
<d>2502.050605</d>
<d>2502.227848</d>
<d>2501.892634</d>
<d>2502.47841</d>
<d>2503.189355</d>
<d>2503.523576</d>
<d>2503.53</d>
<d>2503.165285</d>
<d>2503.71646</d>
<d>2504.426434</d>
<d>2504.423267</d>
<d>2503.73039</d>
<d>2502.717001</d>
<d>2502.363819</d>
<d>2501.660744</d>
<d>2501.150584</d>
<d>2501.523292</d>
<d>2502.784572</d>
<d>2504.455841</d>
<d>2505.033968</d>
<d>2505.647788</d>
<d>2505.615484</d>
<d>2504.330508</d>
<d>2503.59344</d>
<d>2503.329856</d>
<d>2503.574115</d>
<d>2504.190242</d>
<d>2505.537114</d>
<d>2505.929975</d>
<d>2504.965391</d>
<d>2504.408853</d>
<d>2503.155079</d>
<d>2502.459289</d>
<d>2502.199348</d>
<d>2502.442029</d>
<d>2503.530062</d>
<d>2504.388759</d>
<d>2505.394357</d>
<d>2504.970517</d>
<d>2504.675489</d>
<d>2504.765441</d>
<d>2504.429499</d>
<d>2505.301794</d>
<d>2505.119916</d>
<d>2505.906134</d>
<d>2505.741253</d>
<d>2505.415024</d>
<d>2504.928295</d>
<d>2504.54197</d>
<d>2503.847151</d>
<d>2503.786437</d>
<d>2503.745513</d>
<d>2504.087626</d>
<d>2503.804253</d>
<d>2504.479869</d>
<d>2503.727503</d>
<d>2503.858004</d>
<d>2503.989963</d>
<d>2504.595891</d>
<d>2504.305598</d>
<d>2505.619691</d>
<d>2505.320049</d>
<d>2505.903518</d>
<d>2506.127866</d>
<d>2506.380381</d>
<d>2505.552765</d>
<d>2504.793831</d>
<d>2503.795287</d>
<d>2503.007072</d>
<d>2501.398979</d>
</Subcolumn>
<Subcolumn>
<d/>
<d/>
<d/>
<d/>
<d/>
<d/>
<d/>
<d/>
<d/>
<d/>
<d/>
<d/>
<d/>
<d/>
<d/>
<d/>
<d/>
<d/>
<d/>
<d/>
<d/>
<d/>
<d/>
<d/>
<d/>
<d/>
<d/>
<d/>
<d/>
<d/>
<d/>
<d/>
<d/>
<d/>
<d/>
<d/>
<d/>
<d/>
<d/>
<d/>
<d/>
<d/>
<d/>
<d/>
<d/>
<d/>
<d/>
<d/>
<d/>
<d/>
<d/>
<d/>
<d/>
<d/>
<d/>
<d/>
<d/>
<d/>
<d/>
<d/>
<d/>
<d/>
<d/>
<d/>
<d/>
<d/>
<d/>
<d/>
<d/>
<d/>
<d/>
<d/>
<d/>
<d/>
<d/>
<d/>
<d/>
<d/>
<d/>
<d/>
<d/>
<d/>
<d/>
<d/>
<d/>
<d/>
<d/>
<d/>
<d/>
<d/>
<d/>
<d/>
<d/>
<d/>
<d/>
<d/>
<d/>
<d/>
<d/>
<d/>
<d/>
<d/>
<d/>
<d/>
<d/>
<d/>
<d/>
<d/>
<d/>
<d/>
<d/>
<d/>
<d/>
<d/>
<d/>
<d/>
<d/>
<d/>
<d/>
<d/>
<d/>
<d/>
<d/>
<d/>
<d/>
<d/>
<d/>
<d/>
<d/>
<d/>
<d/>
<d/>
<d/>
<d/>
<d/>
<d/>
<d/>
<d/>
<d/>
<d/>
<d/>
<d/>
<d/>
<d/>
<d/>
<d/>
<d/>
<d/>
<d/>
<d/>
<d/>
<d/>
<d/>
<d/>
<d/>
<d/>
<d/>
<d/>
<d/>
<d/>
<d/>
<d/>
<d/>
<d/>
<d/>
<d/>
<d/>
<d/>
<d/>
<d/>
<d/>
<d/>
<d/>
<d/>
<d/>
<d/>
<d/>
<d/>
<d/>
<d/>
<d/>
<d/>
<d/>
<d/>
<d/>
<d/>
<d/>
<d/>
<d/>
<d/>
<d/>
<d/>
<d/>
<d/>
<d/>
<d/>
<d/>
<d/>
<d/>
<d/>
<d/>
<d/>
<d/>
<d/>
<d/>
<d/>
<d/>
<d/>
<d/>
<d/>
<d/>
<d/>
<d/>
<d/>
<d/>
<d/>
<d/>
<d/>
<d/>
<d/>
<d/>
<d/>
<d/>
<d/>
<d/>
<d/>
<d/>
<d/>
<d/>
<d/>
<d/>
<d/>
<d/>
<d/>
<d/>
<d/>
<d/>
<d/>
<d/>
<d/>
<d/>
<d/>
<d/>
<d/>
<d/>
<d/>
<d/>
<d/>
<d/>
<d/>
<d/>
<d/>
<d/>
<d/>
<d/>
<d/>
<d/>
<d/>
<d/>
<d/>
<d/>
<d/>
<d/>
<d/>
<d/>
<d/>
<d/>
<d/>
<d/>
<d/>
<d/>
<d/>
<d/>
<d/>
<d/>
<d/>
<d/>
<d/>
<d/>
<d/>
<d/>
<d/>
<d/>
<d/>
<d/>
<d/>
<d/>
<d/>
<d/>
<d/>
<d/>
<d/>
<d/>
<d/>
<d/>
<d/>
<d/>
<d/>
<d/>
<d/>
<d/>
<d/>
<d/>
<d/>
<d/>
<d/>
<d/>
<d/>
<d/>
<d/>
<d/>
<d/>
<d/>
<d/>
<d/>
<d/>
<d/>
<d/>
<d/>
<d/>
<d/>
<d/>
<d/>
<d/>
<d/>
<d/>
<d/>
<d/>
<d/>
<d/>
<d/>
<d/>
<d/>
<d/>
<d/>
<d/>
<d/>
<d/>
<d/>
<d/>
<d/>
<d/>
<d/>
<d/>
<d/>
<d/>
<d/>
<d/>
<d/>
<d/>
<d/>
<d/>
<d/>
<d/>
<d/>
<d/>
<d/>
<d/>
<d/>
<d/>
<d/>
<d/>
<d/>
<d/>
<d/>
<d/>
<d/>
<d/>
<d/>
<d/>
<d/>
<d/>
<d/>
<d/>
<d/>
<d/>
<d/>
<d/>
<d/>
<d/>
<d/>
<d/>
<d/>
<d/>
<d/>
<d/>
<d/>
<d/>
<d/>
<d/>
<d/>
<d/>
<d/>
<d/>
<d/>
<d/>
<d/>
<d/>
<d/>
<d/>
<d/>
<d/>
<d/>
<d/>
<d/>
<d/>
<d/>
<d/>
<d/>
<d/>
<d/>
<d/>
<d/>
<d/>
<d/>
<d/>
<d/>
<d/>
<d/>
<d/>
<d/>
<d/>
<d/>
<d/>
<d/>
<d/>
<d/>
<d/>
<d/>
<d/>
<d/>
<d/>
<d/>
<d/>
<d/>
<d/>
<d/>
<d/>
<d/>
<d/>
<d/>
<d/>
<d/>
<d/>
<d/>
<d/>
<d/>
<d/>
<d/>
<d/>
<d/>
<d/>
<d/>
<d/>
<d/>
<d/>
<d/>
<d/>
<d/>
<d/>
<d/>
<d/>
<d/>
<d/>
<d/>
<d/>
<d/>
<d/>
<d/>
<d/>
<d/>
<d/>
<d/>
<d/>
<d/>
<d/>
<d/>
<d/>
<d/>
<d/>
<d/>
<d/>
<d/>
<d/>
<d/>
<d/>
<d/>
<d/>
<d/>
<d/>
<d/>
<d/>
<d/>
<d/>
<d/>
<d/>
<d/>
<d/>
<d/>
<d/>
<d/>
<d/>
<d/>
<d/>
<d/>
<d/>
<d/>
<d/>
<d/>
<d/>
<d/>
<d/>
<d/>
<d/>
<d/>
<d/>
<d/>
<d/>
<d/>
<d/>
<d/>
<d/>
<d/>
<d/>
<d/>
<d/>
<d/>
<d/>
<d/>
<d/>
<d/>
<d/>
<d/>
<d/>
<d/>
<d/>
<d/>
<d/>
<d/>
<d/>
<d/>
<d/>
<d/>
<d/>
<d/>
<d/>
<d/>
<d/>
<d/>
<d/>
<d/>
<d/>
<d/>
<d/>
<d/>
<d/>
<d/>
<d/>
<d/>
<d/>
<d/>
<d/>
<d/>
<d/>
<d/>
<d/>
<d/>
<d/>
<d/>
<d/>
<d/>
<d/>
<d/>
<d/>
<d/>
<d/>
<d/>
<d/>
<d/>
<d/>
<d/>
<d/>
<d/>
<d/>
<d/>
<d/>
<d/>
<d/>
<d/>
<d/>
<d/>
<d/>
<d/>
<d/>
<d/>
<d/>
<d/>
<d/>
<d/>
<d/>
<d/>
<d/>
<d/>
<d/>
<d/>
<d/>
<d/>
<d/>
<d/>
<d/>
<d/>
<d/>
<d/>
<d/>
<d/>
<d/>
<d/>
<d/>
<d/>
<d/>
<d/>
<d/>
<d/>
<d/>
<d/>
<d/>
<d/>
<d/>
<d/>
<d/>
<d/>
<d/>
<d/>
<d/>
<d/>
<d/>
<d/>
<d/>
<d/>
<d/>
<d/>
<d/>
<d/>
<d/>
<d/>
<d/>
<d/>
<d/>
<d/>
<d/>
<d/>
<d/>
<d/>
<d/>
<d/>
<d/>
<d/>
<d/>
<d/>
<d/>
<d/>
<d/>
<d/>
<d/>
<d/>
<d/>
<d/>
<d/>
<d/>
<d/>
<d/>
<d/>
<d/>
<d/>
<d/>
<d/>
<d/>
<d/>
<d/>
<d/>
<d/>
<d/>
<d/>
<d/>
<d/>
<d/>
<d/>
<d/>
<d/>
<d/>
<d/>
<d/>
<d/>
<d/>
<d/>
<d/>
<d/>
<d/>
<d/>
<d/>
<d/>
<d/>
<d/>
<d/>
<d/>
<d/>
<d/>
<d/>
<d/>
<d/>
<d/>
<d/>
<d/>
<d/>
<d/>
<d/>
<d/>
<d/>
<d/>
<d/>
<d/>
<d/>
<d/>
<d/>
<d/>
<d/>
<d/>
<d/>
<d/>
<d/>
<d/>
<d/>
<d/>
<d/>
<d/>
<d/>
<d/>
<d/>
<d/>
<d/>
<d/>
<d/>
<d/>
<d/>
<d/>
<d/>
<d/>
<d/>
<d/>
<d/>
<d/>
<d/>
<d/>
<d/>
<d/>
<d/>
<d/>
<d/>
<d/>
<d/>
<d/>
<d/>
<d/>
<d/>
<d/>
<d/>
<d/>
<d/>
<d/>
<d/>
<d/>
<d/>
<d/>
<d/>
<d/>
<d/>
<d/>
<d/>
<d/>
<d/>
<d/>
<d/>
<d/>
<d/>
<d/>
<d/>
<d/>
<d/>
<d/>
<d/>
<d/>
<d/>
<d/>
<d/>
<d/>
<d/>
<d/>
<d/>
<d/>
<d/>
<d/>
<d/>
<d/>
<d/>
<d/>
<d/>
<d/>
<d/>
<d/>
<d/>
<d/>
<d/>
<d/>
<d/>
<d/>
<d/>
<d/>
<d/>
<d/>
<d/>
<d/>
<d/>
<d/>
<d/>
<d/>
<d/>
<d/>
<d/>
<d/>
<d/>
<d/>
<d/>
<d/>
<d/>
<d/>
<d/>
<d/>
<d/>
<d/>
<d/>
<d/>
<d/>
<d/>
<d/>
<d/>
<d/>
<d/>
<d/>
<d/>
<d/>
<d/>
<d/>
<d/>
<d/>
<d/>
<d/>
<d/>
<d/>
<d/>
<d/>
<d/>
<d/>
<d/>
<d/>
<d/>
<d/>
<d/>
<d/>
<d/>
<d/>
<d/>
<d/>
<d/>
<d/>
<d/>
<d/>
<d/>
<d/>
<d/>
<d/>
<d/>
<d/>
<d/>
<d/>
<d/>
<d/>
<d/>
<d/>
<d/>
<d/>
<d/>
<d/>
<d/>
<d/>
<d/>
<d/>
<d/>
<d/>
<d/>
<d/>
<d/>
<d/>
<d/>
<d/>
<d/>
<d/>
<d/>
<d/>
<d/>
<d/>
<d/>
<d/>
<d/>
<d/>
<d/>
<d/>
<d/>
<d/>
<d/>
<d/>
<d/>
<d/>
<d/>
<d/>
<d/>
<d/>
<d/>
<d/>
<d/>
<d/>
<d/>
<d/>
<d/>
<d/>
<d/>
<d/>
<d/>
<d/>
<d/>
<d/>
<d/>
<d/>
<d/>
<d/>
<d/>
<d/>
<d/>
<d/>
<d/>
<d/>
<d/>
<d/>
<d/>
<d/>
<d/>
<d/>
<d/>
<d/>
<d/>
<d/>
<d/>
<d/>
<d/>
<d/>
<d/>
<d/>
<d/>
<d/>
<d/>
<d/>
<d/>
<d/>
<d/>
<d/>
<d/>
<d/>
<d/>
<d/>
<d/>
<d/>
<d/>
<d/>
<d/>
<d/>
<d/>
<d/>
<d/>
<d/>
<d/>
<d/>
<d/>
<d/>
<d/>
<d/>
<d/>
<d/>
<d/>
<d/>
<d/>
<d/>
<d/>
<d/>
<d/>
<d/>
<d/>
<d/>
<d/>
<d/>
<d/>
<d/>
<d/>
<d/>
<d/>
<d/>
<d/>
<d/>
<d/>
<d/>
<d/>
<d/>
<d/>
<d/>
<d/>
<d/>
<d/>
<d/>
<d/>
<d/>
<d/>
<d/>
<d/>
<d/>
<d/>
<d/>
<d/>
<d/>
<d/>
<d/>
<d/>
<d/>
<d/>
<d/>
<d/>
<d/>
<d/>
<d/>
<d/>
<d/>
<d/>
<d/>
<d/>
<d/>
<d/>
<d/>
<d/>
<d/>
<d/>
<d/>
<d/>
<d/>
<d/>
<d/>
<d/>
<d/>
<d/>
<d/>
<d/>
<d/>
<d/>
<d/>
<d/>
<d/>
<d/>
<d/>
<d/>
<d/>
<d/>
<d/>
<d/>
<d/>
<d/>
<d/>
<d/>
<d/>
<d/>
<d/>
<d/>
<d/>
<d/>
<d/>
<d/>
<d/>
<d/>
<d/>
<d/>
<d/>
<d/>
<d/>
<d/>
<d/>
<d/>
<d/>
<d/>
<d/>
<d/>
<d/>
<d/>
<d/>
<d/>
<d/>
<d/>
<d/>
<d/>
<d/>
<d/>
<d/>
<d/>
<d/>
<d/>
<d/>
<d/>
<d/>
<d/>
<d/>
<d/>
<d/>
<d/>
<d/>
<d/>
<d/>
<d/>
<d/>
<d/>
<d/>
<d/>
<d/>
<d/>
<d/>
<d/>
<d/>
<d/>
<d/>
<d/>
<d/>
<d/>
<d/>
<d/>
<d/>
<d/>
<d/>
<d/>
<d/>
<d/>
<d/>
<d/>
<d/>
<d/>
<d/>
<d/>
<d/>
<d/>
<d/>
<d/>
<d/>
<d/>
<d/>
<d/>
<d/>
<d/>
<d/>
<d/>
<d/>
<d/>
<d/>
<d/>
<d/>
<d/>
<d/>
<d/>
<d/>
<d/>
<d/>
<d/>
<d/>
<d/>
<d/>
<d/>
<d/>
<d/>
<d/>
<d/>
<d/>
<d/>
<d/>
<d/>
<d/>
<d/>
<d/>
<d/>
<d/>
<d/>
<d/>
<d/>
<d/>
<d/>
<d/>
<d/>
<d/>
<d/>
<d/>
<d/>
<d/>
<d/>
<d/>
<d/>
<d/>
<d/>
<d/>
<d/>
<d/>
<d/>
<d/>
<d/>
<d/>
<d/>
<d/>
<d/>
<d/>
<d/>
<d/>
<d/>
<d/>
<d/>
<d/>
<d/>
<d/>
<d/>
<d/>
<d/>
<d/>
<d/>
<d/>
<d/>
<d/>
<d/>
<d/>
<d/>
<d/>
<d/>
<d/>
<d/>
<d/>
<d/>
<d/>
<d/>
<d/>
<d/>
<d/>
<d/>
<d/>
<d/>
<d/>
<d/>
<d/>
<d/>
<d/>
<d/>
<d/>
<d/>
<d/>
<d/>
<d/>
<d/>
<d/>
<d/>
<d/>
<d/>
<d/>
<d/>
<d/>
<d/>
<d/>
<d/>
<d/>
<d/>
<d/>
<d/>
<d/>
<d/>
<d/>
<d/>
<d/>
<d/>
<d/>
<d/>
<d/>
<d/>
<d/>
<d/>
<d/>
<d/>
<d/>
<d/>
<d/>
<d/>
<d/>
<d/>
<d/>
<d/>
<d/>
<d/>
<d/>
<d/>
<d/>
<d/>
<d/>
<d/>
<d/>
<d/>
<d/>
<d/>
<d/>
<d/>
<d/>
<d/>
<d/>
<d/>
<d/>
<d/>
<d/>
<d/>
<d/>
<d/>
<d/>
<d/>
<d/>
<d/>
<d/>
<d/>
<d/>
<d/>
<d/>
<d/>
<d/>
<d/>
<d/>
<d/>
<d/>
<d/>
<d/>
<d/>
<d/>
<d/>
<d/>
<d/>
<d/>
<d/>
<d/>
<d/>
<d/>
<d/>
<d/>
<d/>
<d/>
<d/>
<d/>
<d/>
<d/>
<d/>
<d/>
<d/>
<d/>
<d/>
<d/>
<d/>
<d/>
<d/>
<d/>
<d/>
<d/>
<d/>
<d/>
<d/>
<d/>
<d/>
<d/>
<d/>
<d/>
<d/>
<d/>
<d/>
<d/>
<d/>
<d/>
<d/>
<d/>
<d/>
<d/>
<d/>
<d/>
<d/>
<d/>
<d/>
<d/>
<d/>
<d/>
<d/>
<d/>
<d/>
<d/>
<d/>
<d/>
<d/>
<d/>
<d/>
<d/>
<d/>
<d/>
<d/>
<d/>
<d/>
<d/>
<d/>
<d/>
<d/>
<d/>
<d/>
<d/>
<d/>
<d/>
<d/>
<d/>
<d/>
<d/>
</Subcolumn>
<Subcolumn>
<d/>
<d/>
<d/>
<d/>
<d/>
<d/>
<d/>
<d/>
<d/>
<d/>
<d/>
<d/>
<d/>
<d/>
<d/>
<d/>
<d/>
<d/>
<d/>
<d/>
<d/>
<d/>
<d/>
<d/>
<d/>
<d/>
<d/>
<d/>
<d/>
<d/>
<d/>
<d/>
<d/>
<d/>
<d/>
<d/>
<d/>
<d/>
<d/>
<d/>
<d/>
<d/>
<d/>
<d/>
<d/>
<d/>
<d/>
<d/>
<d/>
<d/>
<d/>
<d/>
<d/>
<d/>
<d/>
<d/>
<d/>
<d/>
<d/>
<d/>
<d/>
<d/>
<d/>
<d/>
<d/>
<d/>
<d/>
<d/>
<d/>
<d/>
<d/>
<d/>
<d/>
<d/>
<d/>
<d/>
<d/>
<d/>
<d/>
<d/>
<d/>
<d/>
<d/>
<d/>
<d/>
<d/>
<d/>
<d/>
<d/>
<d/>
<d/>
<d/>
<d/>
<d/>
<d/>
<d/>
<d/>
<d/>
<d/>
<d/>
<d/>
<d/>
<d/>
<d/>
<d/>
<d/>
<d/>
<d/>
<d/>
<d/>
<d/>
<d/>
<d/>
<d/>
<d/>
<d/>
<d/>
<d/>
<d/>
<d/>
<d/>
<d/>
<d/>
<d/>
<d/>
<d/>
<d/>
<d/>
<d/>
<d/>
<d/>
<d/>
<d/>
<d/>
<d/>
<d/>
<d/>
<d/>
<d/>
<d/>
<d/>
<d/>
<d/>
<d/>
<d/>
<d/>
<d/>
<d/>
<d/>
<d/>
<d/>
<d/>
<d/>
<d/>
<d/>
<d/>
<d/>
<d/>
<d/>
<d/>
<d/>
<d/>
<d/>
<d/>
<d/>
<d/>
<d/>
<d/>
<d/>
<d/>
<d/>
<d/>
<d/>
<d/>
<d/>
<d/>
<d/>
<d/>
<d/>
<d/>
<d/>
<d/>
<d/>
<d/>
<d/>
<d/>
<d/>
<d/>
<d/>
<d/>
<d/>
<d/>
<d/>
<d/>
<d/>
<d/>
<d/>
<d/>
<d/>
<d/>
<d/>
<d/>
<d/>
<d/>
<d/>
<d/>
<d/>
<d/>
<d/>
<d/>
<d/>
<d/>
<d/>
<d/>
<d/>
<d/>
<d/>
<d/>
<d/>
<d/>
<d/>
<d/>
<d/>
<d/>
<d/>
<d/>
<d/>
<d/>
<d/>
<d/>
<d/>
<d/>
<d/>
<d/>
<d/>
<d/>
<d/>
<d/>
<d/>
<d/>
<d/>
<d/>
<d/>
<d/>
<d/>
<d/>
<d/>
<d/>
<d/>
<d/>
<d/>
<d/>
<d/>
<d/>
<d/>
<d/>
<d/>
<d/>
<d/>
<d/>
<d/>
<d/>
<d/>
<d/>
<d/>
<d/>
<d/>
<d/>
<d/>
<d/>
<d/>
<d/>
<d/>
<d/>
<d/>
<d/>
<d/>
<d/>
<d/>
<d/>
<d/>
<d/>
<d/>
<d/>
<d/>
<d/>
<d/>
<d/>
<d/>
<d/>
<d/>
<d/>
<d/>
<d/>
<d/>
<d/>
<d/>
<d/>
<d/>
<d/>
<d/>
<d/>
<d/>
<d/>
<d/>
<d/>
<d/>
<d/>
<d/>
<d/>
<d/>
<d/>
<d/>
<d/>
<d/>
<d/>
<d/>
<d/>
<d/>
<d/>
<d/>
<d/>
<d/>
<d/>
<d/>
<d/>
<d/>
<d/>
<d/>
<d/>
<d/>
<d/>
<d/>
<d/>
<d/>
<d/>
<d/>
<d/>
<d/>
<d/>
<d/>
<d/>
<d/>
<d/>
<d/>
<d/>
<d/>
<d/>
<d/>
<d/>
<d/>
<d/>
<d/>
<d/>
<d/>
<d/>
<d/>
<d/>
<d/>
<d/>
<d/>
<d/>
<d/>
<d/>
<d/>
<d/>
<d/>
<d/>
<d/>
<d/>
<d/>
<d/>
<d/>
<d/>
<d/>
<d/>
<d/>
<d/>
<d/>
<d/>
<d/>
<d/>
<d/>
<d/>
<d/>
<d/>
<d/>
<d/>
<d/>
<d/>
<d/>
<d/>
<d/>
<d/>
<d/>
<d/>
<d/>
<d/>
<d/>
<d/>
<d/>
<d/>
<d/>
<d/>
<d/>
<d/>
<d/>
<d/>
<d/>
<d/>
<d/>
<d/>
<d/>
<d/>
<d/>
<d/>
<d/>
<d/>
<d/>
<d/>
<d/>
<d/>
<d/>
<d/>
<d/>
<d/>
<d/>
<d/>
<d/>
<d/>
<d/>
<d/>
<d/>
<d/>
<d/>
<d/>
<d/>
<d/>
<d/>
<d/>
<d/>
<d/>
<d/>
<d/>
<d/>
<d/>
<d/>
<d/>
<d/>
<d/>
<d/>
<d/>
<d/>
<d/>
<d/>
<d/>
<d/>
<d/>
<d/>
<d/>
<d/>
<d/>
<d/>
<d/>
<d/>
<d/>
<d/>
<d/>
<d/>
<d/>
<d/>
<d/>
<d/>
<d/>
<d/>
<d/>
<d/>
<d/>
<d/>
<d/>
<d/>
<d/>
<d/>
<d/>
<d/>
<d/>
<d/>
<d/>
<d/>
<d/>
<d/>
<d/>
<d/>
<d/>
<d/>
<d/>
<d/>
<d/>
<d/>
<d/>
<d/>
<d/>
<d/>
<d/>
<d/>
<d/>
<d/>
<d/>
<d/>
<d/>
<d/>
<d/>
<d/>
<d/>
<d/>
<d/>
<d/>
<d/>
<d/>
<d/>
<d/>
<d/>
<d/>
<d/>
<d/>
<d/>
<d/>
<d/>
<d/>
<d/>
<d/>
<d/>
<d/>
<d/>
<d/>
<d/>
<d/>
<d/>
<d/>
<d/>
<d/>
<d/>
<d/>
<d/>
<d/>
<d/>
<d/>
<d/>
<d/>
<d/>
<d/>
<d/>
<d/>
<d/>
<d/>
<d/>
<d/>
<d/>
<d/>
<d/>
<d/>
<d/>
<d/>
<d/>
<d/>
<d/>
<d/>
<d/>
<d/>
<d/>
<d/>
<d/>
<d/>
<d/>
<d/>
<d/>
<d/>
<d/>
<d/>
<d/>
<d/>
<d/>
<d/>
<d/>
<d/>
<d/>
<d/>
<d/>
<d/>
<d/>
<d/>
<d/>
<d/>
<d/>
<d/>
<d/>
<d/>
<d/>
<d/>
<d/>
<d/>
<d/>
<d/>
<d/>
<d/>
<d/>
<d/>
<d/>
<d/>
<d/>
<d/>
<d/>
<d/>
<d/>
<d/>
<d/>
<d/>
<d/>
<d/>
<d/>
<d/>
<d/>
<d/>
<d/>
<d/>
<d/>
<d/>
<d/>
<d/>
<d/>
<d/>
<d/>
<d/>
<d/>
<d/>
<d/>
<d/>
<d/>
<d/>
<d/>
<d/>
<d/>
<d/>
<d/>
<d/>
<d/>
<d/>
<d/>
<d/>
<d/>
<d/>
<d/>
<d/>
<d/>
<d/>
<d/>
<d/>
<d/>
<d/>
<d/>
<d/>
<d/>
<d/>
<d/>
<d/>
<d/>
<d/>
<d/>
<d/>
<d/>
<d/>
<d/>
<d/>
<d/>
<d/>
<d/>
<d/>
<d/>
<d/>
<d/>
<d/>
<d/>
<d/>
<d/>
<d/>
<d/>
<d/>
<d/>
<d/>
<d/>
<d/>
<d/>
<d/>
<d/>
<d/>
<d/>
<d/>
<d/>
<d/>
<d/>
<d/>
<d/>
<d/>
<d/>
<d/>
<d/>
<d/>
<d/>
<d/>
<d/>
<d/>
<d/>
<d/>
<d/>
<d/>
<d/>
<d/>
<d/>
<d/>
<d/>
<d/>
<d/>
<d/>
<d/>
<d/>
<d/>
<d/>
<d/>
<d/>
<d/>
<d/>
<d/>
<d/>
<d/>
<d/>
<d/>
<d/>
<d/>
<d/>
<d/>
<d/>
<d/>
<d/>
<d/>
<d/>
<d/>
<d/>
<d/>
<d/>
<d/>
<d/>
<d/>
<d/>
<d/>
<d/>
<d/>
<d/>
<d/>
<d/>
<d/>
<d/>
<d/>
<d/>
<d/>
<d/>
<d/>
<d/>
<d/>
<d/>
<d/>
<d/>
<d/>
<d/>
<d/>
<d/>
<d/>
<d/>
<d/>
<d/>
<d/>
<d/>
<d/>
<d/>
<d/>
<d/>
<d/>
<d/>
<d/>
<d/>
<d/>
<d/>
<d/>
<d/>
<d/>
<d/>
<d/>
<d/>
<d/>
<d/>
<d/>
<d/>
<d/>
<d/>
<d/>
<d/>
<d/>
<d/>
<d/>
<d/>
<d/>
<d/>
<d/>
<d/>
<d/>
<d/>
<d/>
<d/>
<d/>
<d/>
<d/>
<d/>
<d/>
<d/>
<d/>
<d/>
<d/>
<d/>
<d/>
<d/>
<d/>
<d/>
<d/>
<d/>
<d/>
<d/>
<d/>
<d/>
<d/>
<d/>
<d/>
<d/>
<d/>
<d/>
<d/>
<d/>
<d/>
<d/>
<d/>
<d/>
<d/>
<d/>
<d/>
<d/>
<d/>
<d/>
<d/>
<d/>
<d/>
<d/>
<d/>
<d/>
<d/>
<d/>
<d/>
<d/>
<d/>
<d/>
<d/>
<d/>
<d/>
<d/>
<d/>
<d/>
<d/>
<d/>
<d/>
<d/>
<d/>
<d/>
<d/>
<d/>
<d/>
<d/>
<d/>
<d/>
<d/>
<d/>
<d/>
<d/>
<d/>
<d/>
<d/>
<d/>
<d/>
<d/>
<d/>
<d/>
<d/>
<d/>
<d/>
<d/>
<d/>
<d/>
<d/>
<d/>
<d/>
<d/>
<d/>
<d/>
<d/>
<d/>
<d/>
<d/>
<d/>
<d/>
<d/>
<d/>
<d/>
<d/>
<d/>
<d/>
<d/>
<d/>
<d/>
<d/>
<d/>
<d/>
<d/>
<d/>
<d/>
<d/>
<d/>
<d/>
<d/>
<d/>
<d/>
<d/>
<d/>
<d/>
<d/>
<d/>
<d/>
<d/>
<d/>
<d/>
<d/>
<d/>
<d/>
<d/>
<d/>
<d/>
<d/>
<d/>
<d/>
<d/>
<d/>
<d/>
<d/>
<d/>
<d/>
<d/>
<d/>
<d/>
<d/>
<d/>
<d/>
<d/>
<d/>
<d/>
<d/>
<d/>
<d/>
<d/>
<d/>
<d/>
<d/>
<d/>
<d/>
<d/>
<d/>
<d/>
<d/>
<d/>
<d/>
<d/>
<d/>
<d/>
<d/>
<d/>
<d/>
<d/>
<d/>
<d/>
<d/>
<d/>
<d/>
<d/>
<d/>
<d/>
<d/>
<d/>
<d/>
<d/>
<d/>
<d/>
<d/>
<d/>
<d/>
<d/>
<d/>
<d/>
<d/>
<d/>
<d/>
<d/>
<d/>
<d/>
<d/>
<d/>
<d/>
<d/>
<d/>
<d/>
<d/>
<d/>
<d/>
<d/>
<d/>
<d/>
<d/>
<d/>
<d/>
<d/>
<d/>
<d/>
<d/>
<d/>
<d/>
<d/>
<d/>
<d/>
<d/>
<d/>
<d/>
<d/>
<d/>
<d/>
<d/>
<d/>
<d/>
<d/>
<d/>
<d/>
<d/>
<d/>
<d/>
<d/>
<d/>
<d/>
<d/>
<d/>
<d/>
<d/>
<d/>
<d/>
<d/>
<d/>
<d/>
<d/>
<d/>
<d/>
<d/>
<d/>
<d/>
<d/>
<d/>
<d/>
<d/>
<d/>
<d/>
<d/>
<d/>
<d/>
<d/>
<d/>
<d/>
<d/>
<d/>
<d/>
<d/>
<d/>
<d/>
<d/>
<d/>
<d/>
<d/>
<d/>
<d/>
<d/>
<d/>
<d/>
<d/>
<d/>
<d/>
<d/>
<d/>
<d/>
<d/>
<d/>
<d/>
<d/>
<d/>
<d/>
<d/>
<d/>
<d/>
<d/>
<d/>
<d/>
<d/>
<d/>
<d/>
<d/>
<d/>
<d/>
<d/>
<d/>
<d/>
<d/>
<d/>
<d/>
<d/>
<d/>
<d/>
<d/>
<d/>
<d/>
<d/>
<d/>
<d/>
<d/>
<d/>
<d/>
<d/>
<d/>
<d/>
<d/>
<d/>
<d/>
<d/>
<d/>
<d/>
<d/>
<d/>
<d/>
<d/>
<d/>
<d/>
<d/>
<d/>
<d/>
<d/>
<d/>
<d/>
<d/>
<d/>
<d/>
<d/>
<d/>
<d/>
<d/>
<d/>
<d/>
<d/>
<d/>
<d/>
<d/>
<d/>
<d/>
<d/>
<d/>
<d/>
<d/>
<d/>
<d/>
<d/>
<d/>
<d/>
<d/>
<d/>
<d/>
<d/>
<d/>
<d/>
<d/>
<d/>
<d/>
<d/>
<d/>
<d/>
<d/>
<d/>
<d/>
<d/>
<d/>
<d/>
<d/>
<d/>
<d/>
<d/>
<d/>
<d/>
<d/>
<d/>
<d/>
<d/>
<d/>
<d/>
<d/>
<d/>
<d/>
<d/>
<d/>
<d/>
<d/>
<d/>
<d/>
<d/>
<d/>
<d/>
<d/>
<d/>
<d/>
<d/>
<d/>
<d/>
<d/>
<d/>
<d/>
<d/>
<d/>
<d/>
<d/>
<d/>
<d/>
<d/>
<d/>
<d/>
<d/>
<d/>
<d/>
<d/>
<d/>
<d/>
<d/>
<d/>
<d/>
<d/>
<d/>
<d/>
<d/>
<d/>
<d/>
<d/>
<d/>
<d/>
<d/>
<d/>
<d/>
<d/>
<d/>
<d/>
<d/>
<d/>
<d/>
<d/>
<d/>
<d/>
<d/>
<d/>
<d/>
<d/>
<d/>
<d/>
<d/>
<d/>
<d/>
<d/>
<d/>
<d/>
<d/>
<d/>
<d/>
<d/>
<d/>
<d/>
<d/>
<d/>
<d/>
<d/>
<d/>
<d/>
<d/>
<d/>
<d/>
<d/>
<d/>
<d/>
<d/>
<d/>
<d/>
<d/>
<d/>
<d/>
<d/>
<d/>
<d/>
<d/>
<d/>
<d/>
<d/>
<d/>
<d/>
<d/>
<d/>
<d/>
<d/>
<d/>
<d/>
<d/>
<d/>
<d/>
<d/>
<d/>
<d/>
<d/>
<d/>
<d/>
<d/>
<d/>
<d/>
<d/>
<d/>
<d/>
<d/>
<d/>
<d/>
<d/>
<d/>
<d/>
<d/>
<d/>
<d/>
<d/>
<d/>
<d/>
<d/>
<d/>
<d/>
<d/>
<d/>
<d/>
<d/>
<d/>
<d/>
<d/>
<d/>
<d/>
<d/>
<d/>
<d/>
<d/>
<d/>
<d/>
<d/>
<d/>
<d/>
<d/>
<d/>
<d/>
<d/>
<d/>
<d/>
<d/>
<d/>
<d/>
<d/>
<d/>
<d/>
<d/>
<d/>
<d/>
<d/>
<d/>
<d/>
<d/>
<d/>
<d/>
<d/>
<d/>
<d/>
<d/>
<d/>
<d/>
<d/>
<d/>
<d/>
<d/>
<d/>
<d/>
<d/>
<d/>
<d/>
<d/>
<d/>
<d/>
<d/>
<d/>
<d/>
<d/>
<d/>
<d/>
<d/>
<d/>
<d/>
<d/>
<d/>
<d/>
<d/>
<d/>
<d/>
<d/>
<d/>
<d/>
</Subcolumn>
</YColumn>
</Table>

<!--Analyses, graphs and layouts as compressed binary. Don't edit this part of the file.-->

<Template xmlns:dt="urn:schemas-microsoft-com:datatypes" dt:dt="bin.base64">eNrsnQVAFM3fx/eOjoPjqKNPLCxUQsEgBFFMFFSwEAUUpSQMVELBwMJ8BAsLW7EwMLCwfezE
DuzHTpR3927vfrvjDXI+PM+rz//meVb2s/vd2d/MTu3M7Jyfl49Pm66eTpxUXYIgsjhZHC75
bygRyF1CDCKPBHIJlhOpEIQq+Zf8Q7yoRhBc8nwYEU1U1nUj/OUe56b61SYIb3KPQ24DyU2N
3N5yJGfpP0Q56aTMZRznKHg3pVM6pVM6pVM6pft1HUcZBUqndEqndEqndEqndEqndEqndEqn
dEqndEqndEqndEqndEqndEqndEqndEqndIQK0Yr895khQRw1I4iuKgTRQVWR66npdi8JDqEm
GYlXJRZU8sIF/08BVk/liu3UEAdc4jzjIkIiK7imBrnpMThLMutAhcuxpf2RTDf0HxnVP6YC
j2qTm6FcfwS0P1zx1iMiemAoucVXYI+lXH+4tD8q4gMBEVFh8aJOYcNFXWOiQr6fglmd3HgY
e9Q9xoincVJTOJNle7MJTXjw5RLisIjLIhUWqdI0mZCmsTnEBQ3w7+etZTvlpE6lUzqlUzql
U7p/31EV83xZHV9MDONKWyRKp3T/puN6uKZKvk/SErcf/SOiYiPDRJER0WEhcaK4sIFxYfHx
ETHRophwkXdIQoioMaFN2HHYb0Lib4q4qbJvi5SOEMcR5ZoRfHGGVxP/+4WOrR1Efbq5L69o
kDdnu7nsVYJyLcTXaxPwHVf5D5ymaDSpslbgiqp2LcWviBziK7lfRm7fyC2XsCPgOzTmmW9y
fHhCSMNAMMKhQsckFcPq5Ea9L2mKE7QkhnTITZd+NaIs0Cc36pkYkJuAfuE0IjdjcjMhN1Ny
E5Ib+bpPmJObBf0yaUVu7oSIvqPs2zlVI1keENGWeYj9DwjpnxgpzkTxiZEJ8YxrObTFHD9C
JH051JRdS4WjA5n/GBfInCY8Oq7sArEZ8sUqiohVFRGrKSJWV0SsoYhYUxGxlnyxtkieWBsR
64gqEOsoItZVRMxTRKyniFhfETFfEbGBImKBImJDRcRGioiNFRGbKCI2VUQsVERspojYXBGx
hSJiS0XEVvLFPoS3muTAFWNTZetB6ZTuP+KozK5Shc3NUIZPqmQFro7xWb41NTwkfwPov0YI
o+6lu/SvxNe/Uqrqr7QpHyqJG/q4+K94U6UbiupwvlLbBbGv2bKXizxxPSi9G5/eqLMriTFI
AS1V+UfGxIZVZSLYael+UHTeoIgKIXWTMfRxMAFdhyKoQUR0QljcgLDYhKoxwa3epmWP1qZ6
SEzgyDGBi5gQWNUmDG3euRnnRR/aBK4cE1QYJuj8Ew/iWNlM3+lXt7nTD0LnexNUEROq/EE0
Wahx5nFbvvRByDFBDTGhyh8EMy+SD0KOCerI63iVP4gjb+fnjkoWSHME53sTNBATqvxBnHsx
J4MXKcsRckzQREyo8gcRP7P+mM+OshwhxwQt5G2/yh/EWu+7Dw7XlhVN3O9N0EZMqPIHsWbg
yf0Hn8oehBwTdBATqvxBqNdKr/51W1/pg5Bjgi7DBOo905vqGwyLpzoGfcid0JioqrCj3Xra
BELvexN4iAldRfFDE0PiwkKrKi3cm+NWPOPVNbp05MgxQQ8xwT8xiooBf7Ed8VVgwkzXz5Of
PhrvST8IOSbooyaMtB9RpS02tzneLnU79qYfhIrMhNWyTktm+wo1gdl5WbkruApfoaLwFUqr
lFYprVJapbRKaZXSKqVVSquUVv2vWOVKz/HgEP2I5eT7omUC24YaJBxFekf+jZ5hygbmZPDq
5IEjDLa0I4hoC4IQte7oQ6kpuzeqfD9toruaJMB3yev1kbkT4T01iG62qkQ1ujfDgH6TlU4Q
4DPCr4OwCa2lxuQE9D7lvTG9L+0b4DPuZ03r0bg1I2TjvkRNeJtO7UD3AOPiQHp9NeIl42iq
u/x9+Y55P2k8MMMjPSa18eXLl9wA8m+H73rMJJ23vWrwWnWryWvFvEcHAmYmUPyU3OrTx1G/
npBbPfreOL8EGL9q0/ErfcxFh49xmWHkMMJBnfvZcDymWV44HisYDnl+MZ+JHcN2W0gn5fKe
lQmjJ6YrucVyJMffIXl2N11kUJ9gdCT8CRG5hRDRRLx4L4yIIyKIcEZa1TTGLBSZKvlze1IN
cZGi+mjSt28awOkWmWLOOmEg7p3ZayBh20r0wFJ/PenrpexvXW0ntS/92/Ne5jdmqXk5WnL/
2JAjWky91N9vwlHDCUYX8tzswcPpdCLm2LVzWXY8Wi1hNVn+FYn/+q174MGh9aHDJB2hzDQm
jXuunLinv7tQxr2CcW9JT+3iVxD3XEZ51Z/xPAwY++0ZZbIao7xWRfZ9CMkcEcqvgE5+furk
xV0oe+m6QVr2WzCYQMbR/MitEf2opEOI44kXsvN2DC3nB4uxUlFpU16H3urWgX3Zsbo2ZGy7
k1tKOaWX/KUeEjUxri5pBWX/S45i9udS1zAaJsXERIz9mt/ZbI3YX7rNg3hW0JYo3e5JPNre
ivhwJpZ4uM2deLKrLfFwqzvx8Ww8UX5jIvHl4kji1ZF+5N8korTAgyi7PIb4dj2duLvJk/AI
eE+8KPQmz/cn7m9rS3z98JRYvPoz8XBXN+LTsz+Je9vaE1vXHSDv943gcDiGTEcxhyOU/i+X
heCqRG9qbmkFrC8wMbOwlOk19Qx0dMzMpeeNNIx1dHRMTGV6Vb6OjoFAZr+Wto4uT0+fI3Nq
6lwNTQ7DcTkqHFUZoWlYg8OuQ5hlllCWBopk7ZRqdNp4TNctJgw9s96xQNo5zHqIqheprxVD
aL9W0fmMWS9Kz/GQ+phZJ1M/luBLlpMJ4jIyloghW4chJEWQe9F0ntWQ81eDMWCmytjX+IHd
7ek824guJ0SMkS5+Be1jKTPLJCHDX2bcmtBxW/1vxO06Om5N6Li1lRO3JrQfuLiNFPsVQpaZ
kWTsisgYDSf/jSP344lE8lgC+VeDHvdUp8OkzhgLVWFs6gRMtuUyhmz/ybhmpmk7Rrzb/UPt
WIJuq1F2tpLTjqXO2VB1aCXaf/L8+rfasdJ7cyoIB6FgOJh+VYWNNhXEtY2CcS3Pr6qMR8rf
BhWkh/oKpocGVWyjZwVp1lPBNIv6pcg7C5q+H02ciE3f1LnfOcxxVBtKVVKOOTDuJ2W+rIwr
5/zoHV7anKbKaye6bUaoEpj2mEjSzyN3QYJy6f//KTdgwIDksLCw8sGDB5cPGTJE7kadr8iP
8PDw5KFDh5YPHz68fMSIEd9tMTExP4w28v7JiYmJ5aNGjSofPXo0a4uPj69UtEdHRycnJSWV
p6amlqelpck2yobKxgdpQzJ1/fjx48snTJgg3pKTkxV67GQYkqnrJ0+eXD5lypTycePGKZxs
SLuTqWtnzJhRnpmZ+VPJLiMjIzkrK6t86tSpP51syXsn/53rKUden/xfyjPMfqI3FfQTeYp7
JkIkfbL8VjpMXWX7KvhrZrMOu3LEr9MExzTuK9V3kO84VNwnYHBjl7h8b9ZLohu0VnKdH339
SyJiOJNT62ggN5Scj6Wvk17v3Udi9pj12eLzR78ztE1YgufwkLiw6LD4eJ+4mCjv2AiaCcIr
JjohbAQ1parFuVixvWWcb6wAR6+6OZzlnUgST1c2zpbbTyRtA6vLaZ++P3r0b9V7rei6KlBO
vdeKrod6VLLeQ/2S1t9cTP3NrcL6W7o+DC4chILhYPpVFTbaVBDXNgrGtTy/qioee1SQHnoo
mB5Qv+S1g7iYtp/0XXh7VJTYhgCaW9P+ByA28OlzHPpeqnLSJNVP1Zh+p2fes6L89Xff/wLp
+0fLic9AOj6jKhmfqF9o/kLf/7hV+E4QSNuICwehYDiYflXV+x8urm0UjGt5flVVPEZVkB6i
FEwPqF8/k7/GZUxl5a9etP+D5eSvXnLyFzOe/Oh3lX8zf0XT90+XE5/RdHyOq2R8on79m/kr
mrYRFw5CwXAw/aqq/IWLaxsF41qeX1UVj+MqSA/jFEwPqF9Vkb+G0v6nyslfQ+XkL2Y8UfnL
8V/OX+n0/XPkxGc6HZ/ZlYxP1K9/M3+l0zbiwkEoGA6mX1WVv3BxbaNgXMvzq6riMbuC9JCt
YHpA/aqK/DWB9n+unPw1QU7+YsaTH91f9m/mrxz6/vly4jOHjs+NlYxP1K9/M3/l0DbiwkEo
GA6mX1WVv3BxbaNgXMvzq6ricWMF6WGjgukB9asq8tdC2v91cvLXQjn5ixlPVP5y/pfzVz59
/2I58ZlPx+fhSsYn6te/mb/yaRtx4SAUDAfTr6rKX7i4tlEwruX5VVXxeLiC9HBYwfSA+lUV
+WsL7f8BOflri5z8xYwnKn81+ZfzVzF9/xI58VlMx+eRSsYn6te/mb+KaRtx4SAUDAfTr6rK
X7i4tlEwruX5VVXxeL2C9FBZvwQYv/5u/qrIBip/HZOTv5jxROWvpnLyl/Se0jk7TzFzfH7U
l/938qL0nsEcyT2YcS89R80LN65E+pDn17+VF5/S875w4TBWMByoX4qkoX8rzH05+GenaL6R
59ffzTfU6loqdLwLkHivyYGlV6T5Bk0/fvRcBi3knsz5z28qmP+sHFOs2jFFZK5hOVoOUY/i
7+RfE/rZ15eTf03odFSvkvkX9UuaJ1UweVKlCvMkLhw/kydRv+TlSZUK2m3MeZDM5/h39n92
LmkslU84kudAPcvayLOUnlNnlAnPaX/60HFAjRt4ETFEoniubry4bNBgbNJ5uRXZ8qNwqbPT
OaserspxdOk3OdTzbSUnzden48mzkmke9QtN8+g4ukoVjf8y792ggnDUVzAcDapwjPopXZfg
4tpTwbhG/apMvkTbB//0c+nLwaevnymL6v9EmOW1W9Ex+h/ZQ7UVGjLsUf1B3lHELyOMX/K+
Y+DKyf9/t85rRd83UE6abEWnyR6VTJOoX2g6kzZJmensV0y31DcL3SuIl+4KxIs8v37FMPfh
wPML+pt5VZ5fioS5qtJ2IG1DtJxnGEg/w6hKpm3Ur386bX++eQH7nKlzfydeoiqIlygF4wX1
6++E+XnGBGyYqXN/px7CpYWfqYcC/0aYqyptR9M2pMt5htH0MxxXyWeI+vU7p+1xFcTLOAXj
BfXrV03buLTwM2k7+ifCzHy3Yby3cP/Ou9/PvuNF0u9s1LueLnlBCvKspefUkHe8aLreGkeX
9c6EI/mfvWzcT5H3O1x8VHUZkE4/qxw5aT2dTuvZlUzrqF+/cxmQXUG8ZCsYL6hfv2oZgEsL
P1MGpP9EmH+lMiCWztPjKR2H+h3R78uA8XL6edLpMiCbLgMaEE5kCeBElgFN5H53/auUAzn0
88qXk95z6PS+sZLpHfXrdy4HNlYQLxsVjBfUr1+1HMClhZ8pB3L+RpirKm3n0zYUy3mG+fQz
PFzJZ4j69Tun7cMVxMthBeMF9etXTdu4tPAzaTv/J8L8K9VxuXSdtZl+1vvl1HHUOT2kjsun
6zjqmQ8St3MdyI1q54qIBCKG/NeZrPEai4/Ia/eqEgS2LvyV2sbF9PMtkZM/iglYa64y+QP1
63cuN65j4kXRPCTPr1+13CiuojDL8+t3KzfW/SDcz+kxfj5SbhTT5cZ1+h1b0jZuQpYeTrKS
Q3LMgXAhXCssIypTlvxMGVLZMZO/M19JRI/VSMc7mOlJeu4JUblxPHl+/VvzBJj3blBBOOor
GI6qXNNFRMcjLq49FYxr1K9fdewJl74eK5i+5PlVmTD/U+ONItoO6XgK+jxb0WHrUcnnifr1
u9bLIjrMuHjpoWC8oH79qulc+vyC5KRzaVoIrGQ6R/1StF6WN9cVPV6Z/Z+tl0Np27zJrSW5
+SPhlp5TZdTLtnS4H9PPXBJ/EUQ0ESZ+lqr0M9VUoD6t6vweyBhjQtN1IP2MoyqZrlG/fuf8
HlVBvEQpGC+oX79imB9XkBYeK5gW5Pn1u+V36VqWPcltJDX7Vk5+p87pI/k9kA5/FN0ObyVe
xTKBbHeFk34kkOlhmHjGcaL4uGTusibd162K2aQ/Gs8sL9T/H8uMaMaYFJo3oul0Mq6SeQP1
63cuM8ZVEC/jFIwX1K9ftczApYXHCqYFeX79bJnB/QXe46XlRyy9pcgpP2LllB/RdFyMo8sP
key/IMb6wwPIf2OJBEx5oUWw1xzGvef/f/cDiuhnLR3XQvNMOp1+siuZZ1C/fueyJLuCeMlW
MF5Qv37VsgSXFh4rmBbk+fW79QNOpG0bT89PnSOn/KDOaSPlRzodfiqe+rHKD3+yPIkhS42w
H5YZv8rYuYh+ftKxQTQf5NBpYmMl8wHq1+9cPmysIF42KhgvqF+/avmASwuPFUwL8vz63d5P
imjbFtBhWSunfKDOGSLlQw4dfiqeqPaDK+FM2iMivMjWhYiwI99N4sn3nShx6yJG/PsGA4g6
ZOilZYI2o7xglh/SdxINAn7DSpXxV/Un2h1VXZbkM8YL0TyTT6efw5XMM6hfv3NZcriCeDms
YLygfv2qZQkuLTxWMC3I8+u/8N6ymX4/2S+nXNks570ln46Lw5V6bykv/93fW4rpZ14iJ88U
0+nnSCXzDOrX71yWXMfEi6Jj+fL8+lXLElxaeKxgWpDn1+/43lJRHNrS8xfQ95ZiOvzX/5/e
W5jxydyn5mcZ0Xo+va8lvtI3OiEsLjYmMiQhIiZaFBMu8g5JCBE1JkNmRwa4K2lUB1WIB4Kb
KnnWEdFhHcJC4hNE8UMTQ+LC4kXhEQnE/56zo1eZaEY/KTXxL9CVU1s5QeyQ/Y4s6izpDXXN
xbleunRFC/FvkWjRca9CwG/SynMbxXfTRtRqFVyhKaJ+085agSuq2rUUzxzkEF/J/a/0sVxx
qLl0PDDPyHNu4va65Jf/nhDS8Kgzxhs4dBxKKyBmOKnY0qHzCY+ex6hP5xEDuswwpPOLMZ3v
TOk8R5W75nQ+pZ6lFbm5i0t85i8DS3/Qj7JVulKOh+QJg1gaWELVSPyXEotkYsqQgJD+kWFU
9iTzWmJkQjzjYg4dHg6fI5L+Pqym7GIqJrwS44aFIVeIbyfk2KlU6gpmaFR/FBqmWE0Rsboi
Yg1FxJqKiLXki7VF8sTaiFhHVIFYRxGxriJiniJiPUXE+oqI+YqIDRQRCxQRGyoiNlJEbKyI
2EQRsakiYqEiYjNFxOaKiC0UEVsqIraSL/YhvMkSpT+niLhibEoondIp3X/TPWKUCBwvVXGr
I1jOOaaT/nIql/X2xqV/fZsrbpNDy5GqYemWtsd0D5ZHRf09crIpt9pd/p2MaH2Ah8Svv1Iq
+5f9Vvn9cYl76f6j+JEWjuQfyo8UaetW+mvh8trKBOMN+CChp021BKg4sNQYS1zhOsl+JZzD
slByhElcFqmwSJVFaixSZ5EGizRZpMUibRbpYCz73molKUlJSlKSkhSlVBalVaD8detGXRbx
WKTHIn0W8VlkwCIBiwxZZMQiYxaZsMiURUIWmbHInEUWLLJkkRWLrFlkwyIRi+xYZMui6iyq
waKaLKrFotoV3KEOi+qyqB6L6rOoAYvsWdSQRY1Y1JhFDixyZFGb1PqM3mfpX0eCo81h9Bpy
GK3LtqlxjPa2uIVZ5PjprzaVaMeKdaaVa+8y/RTvjxT3NHGpOy86lpJSLvn3KL0eOReE5bAX
T4gYbWfqF8GZbWUpnxT3PhOMljRlHVsJx3zF/SVBLUVB4nGUAWGxCaJ6osC6/pExsWE8bcKX
b033EuM1fpJVoiKiwwg/vhbdMS3GzZJnsJmvJn0cWyQzYciTIXGiuLCBcWHx8REx0fbEFr6p
dJrM9ye3it+eqCg6IesJpZ75V2nntNh1IbaqEvQblNIpndIp3e/hukoKR3/xsvlQyhLiElYy
6sI4SpVvkjMBrPF1qdvEKhUPwW9cpLqLy1Cqp57e9T0mG6tkVGPi6uaY6XeHYqYQz7S+6+TB
qinTlU7plE7p/r/dr9Z7zfxtnHcV/DZOR7JSoOZehRDR4u9U/cXzNyOIcKLyv41ze1IN8XiD
6qNJ36jflpFyukWmmLNOGFCj78ReAwnbViLs1F9P+nop+1tX20ntS//2vJf5jfkmdjlacv/Y
kCNaTL3U32/CUcMJ+vWBcnOzB4u56PAxMceuncseYVnNZkvxzBny7X/dAw/qjrHEXLm/jSOd
m9if8TwMGPvtCZi3pkZIhsx1Ccm8Eea+DyEZe6f8Cujk56fOod5BJN8EaNE6ISEZZJay1PHp
zU/8tit5VNIhnPHEC9l5O4aWQ3B+9FwIm/I69Fa3jg0Zs8DiY3WpY+7kllIuedul/lIPabT4
TT5VbP9LjmL251LXMF64i4mJGPs1v7PZGrG/dJsH8aygLVG63ZN4tL0V8eFMLPFwmzvxZFdb
4uFWd+Lj2Xii/MZE4svFkcSrI/3Iv0lEaYEHUXZ5DPHtejpxd5Mn4RHwnnhR6E2e70/c39aW
+PrhKbF49Wfi4a5uxKdnf0putu4Aeb9vBIfDMZQ5SVo15HCE0v8NOeKNYLFQ4iRqzvd68Ynv
9eQVQsPv9WK1FbC+wMTMwlLmv6aegY6Ombn0vJGGsQ6hY2Iq06vydXQMBLTthoZa2jq6PD19
jsypSc2UOi5HhaNK7YgPommYS+cR6TaFP0X6YH+Rf0aL02EW0Y+YQaa+toQLsVo2S7EcKUmZ
+YX5O16KX9HWo3uqNiEp5SWaHnLb3oFyjwaJ45gjq104RNN5/dov3VTNY5W4T4dbQa7mVGgv
0zFz1z/lb3ePNh4XJKVzqsT3P8TfOFIunPZ1PenhYXp94IHIHXsS/9LcW6lR81IlE6+owtOU
MUunvni+EmUK7ztbeA3oWZ2SJ6ZN3Fel+iv59Bj9EEKyrgT1jfgscQwq4uZ57BTXy7sIEW1N
a/LO4snLg8IiY5VNtf9Z50E9/2ZdwwYGS7pAg4dHJAwKjo6JjhTj/0QciJCZMTs9HtM95WTZ
oybOfuLvEuZ6ZMvmpeexSld9AlZxWUmMIWBqHoehYnSnVI3Tr7dp2aO1qR7UxwbUTcbQx8EE
DmIC3blTda6fpftB0XmDIokJHDkmcBEzqjwW8l7MyeBFymKB870JaExUeSxEv52fOypZII0F
OSaoMm7P/SdiYcTAk/sPPpXFAvd7E9QQE6o8Frp6331wuLYsLcgxQfpFELVRtZ03Nd5C1n8x
4SIfcic0Jqoq7Gi3XhoLej82oStdCYdWVSzcn+NWPOPVNXc6Fiphgmfo4MT4hLBQUVXZEn1v
yTetv6QmcCthgn9iFPUQ/CXtkSqIhZmunyc/fTTeU2KCSmVMGGk/okqLdbc53i51O/am04Jq
ZdJCR//WVWpCzf4XHjp0kJqgVpm04Os1oEpN6L+zNNLP/w5tgvoPTBAPz8aLfCJDBg4MC3Wv
UksIOlOW/9iETolR/cPiqBQZQZaS4oZqVSRKPh0LHJkJ8l9GpCbIe0Gr3BVcha9QUfgKpVVK
q5RWKa1SWqW0SmmV0iqlVUqr/lescvXQpIcCtMRDJtj1Nyinyq14rOF/yVHRIF1/Q4doRD8F
bXprSUfzInpLIJXdye0Nodx+xU26dskbzAhaDt3nJ1qiIcui38gkMJ/DIV5xuXKPMbMbh5EV
PYZJMu008Vo/1JHajA7Flkh/N+WjFm0dB58Wy9UZ1+jKZpdQjpoVECe9d4r0LKfCs1zMWZ54
voPUPipkHiMJYiFtJxUyWSgTYF9ylcQy+qphHmT46asYX7Uw9/VTWzBCwZyfE8bo6VCtIG60
xM/OQZQwKCwhhOAz5pa/kY1LSOc3UG66eBaHOuNOUl81kO5/ptNGOIuOPX0PSQg4jHQgLwSa
jPICrQK0xJPqvUISB4TER4REi2LjYmJjIiPi6cBwfiowAsbjQ11LuYHhygKjyzBS8cBQd249
cGRsQpWFxUDhsHDIsEyiHxEMQ2nS9SCHtNKR6kSNiIqNDBNFop8zwBpVzQJC+idGik9K1sSR
1JnU1+GsL8S5yrryx3WmLqNscOFIti6EcvuVt770s+uCqTPtCKgzme4audXjcCo8xpGTrTmy
OlOFvkLqdBn1IOWkdSbxgzpTn3HN93VmF1a9Jz0rHX/8vqZCiyBKFc4Yu6UO6CLhEtGblsRO
PjGeIz3MIVKJsWLRPF/qoBe5NeL4iffFo3ucebL9efQ+ZXI/1Vhfthmp4h9U8VPrB/6ogz+p
KvO+058m/wpVb8v0L7VA308N7vtR7ftrKZuz1CUaaskMO42J4n0qivrpe3ynNyBPaqry21Ea
qiHUT8AX6ymbR2ilysL10jBWvE/9uGOs9u3v/KFcromH3OP9TAlf2QQunTOy/X7qwnay47yX
suOxGkS7N0SWiuRBUbWQJeNhSX0VD1IyXo9ESHL7p89V5P4JO//t+ynD/u/Z+buEz0BclpaR
p1uFxSc0CI9IEA0LiUwMiyf0ORwR6SSTMbQkENQgQjY7hD4f+P2hxg3jxRfpcDj+CaH2otZx
cTFxhPaP/XN1rinyiokOjwgNix4QJhLPRCGtiafmD/+ELW1iYkKjyWameBZHhEwFkxl4tLfU
AL8mh+MbLxIbLoqPGBgdER4xICQ6IXKkKDomukFSWFyMOyGS6H0INcmOt090ffKfUEJXwn6S
uKMCLj4dNixCMpcxPC4mSiTxgwyKdIojdU+q7UuoSvQwuBwofQgmkjMdQ0ZERCVGiaJliiCy
mRwbSZqYQKpsJaqAmISQSIaG9sMc9T0qgmx6Rw+UnrfkcC4qU4EyFXDgrZA59aGMA+9d8hp/
upjX9Znin30RifcNyf945L4h+S5oSPiIpyUSRDdCjxhAHo8lJBP2x5GtgsUkryN5H33sHPn3
AVlKvafvw+XUIGpxtIiGHD7hztEnOnEkC+8O5kjOD+NoEskcc7IFV4vYy7EmSjgiooCgvoAx
Z9hoSIeF+ssjYKFwQzrsjvS+9MMW6TTKbvRkGKoBPoC+lmqax9J6bULyI1mWhOQ71cW0Rpdu
Z0nvt4/e1xeHULJPtYoe0G/g1Paevs5QHGpquQrJxwm16A9SqI9vGnIk11FLU7pzJP5RM7k7
cSQ2mIljRuKPhTh2JPZS55LpOLESt3epJS8kH6Ds5Uj+2pBbCUcSL1TqlvZYqCIvAtJmufQj
CU2iomY5FXNeMYnRCfGsxjnazreXXOYrbcTmEnzZfiOOZN+Z3Io4jXxx1axUE8ttJLtWpMKv
UE91sl3h5PpKAzWCmyXb11TJqvBayuZGGmDzGY2K70WFu58mIQvLbc1Gsv0srUayhrqHNvoC
InHz1Ioq9J9y+bp8WSM81Ty3rXQ/iwcNeL4e4Su/cf7jRsyv0RD7J+z8t++nDPs/0Tj/vcNn
QDwnJL9A0MDJ3sm5CaHO4Tg7Oto7U02Pxo1d7ZtSOw0a2Ts4ODkRGhxOI/tGLs6OztSuk32j
po3Fp53smzg4iRJiROSeg4srwSc9cXC2d6YOOTs1Jn3To3xrYu9CHWns4GDvQh1pZO/k0LgR
dcvGjR3tHSivHJo6NKGaKY3rixybOrlSt2lB3rJRo8bUrj+0l6i2T5CopUhidt1AUT2RxG4j
yjcHZ0dxAKhv7hybOjemvGzq3MiB+nuR+EAPG/3PBJmuDtVxDbDHdMOiogYYM9XIa4BJ+oW1
xeMfWmT5riM7WpvcdyBgIYvmZCOjMyEgQsSDCxJH9cWPYWjGk5X8NPK6bHJbTjfmtjD6+06Q
2guEGnGV1N0htwLiI9144TDGeLj0X+mvZRgS8Ate0gaUBl2X6tANCwdaoyO2VOInFf7O9DAM
1bgKoff5Yssl96AaVGPoa40Iya8UatKNqGm0fyaE5EM5HboRtZxuoJmJQyf5+tdSHDqJP1TD
ifr6T41uOF2l/aQaTnfoffKhVjg8wiV9sqvc8Ih4DSnlmMjPj4no0H+Z8wiU4w6/9lb/B2Mi
oZgxEcmqEpwKjzE/OaLK2qKhXMaYiLT0RUel0TERHUyprMrwQ9ExEekHUR7x6JgIF5lyxKXf
TdQI+PUhHUa4mOFER++t5bzWizyle46Oju5vxPMMmIPH3B+MAlHuorjU1GHcS4NZtUkXC5F6
ocMYBEKdDqZqk76LqiHmSKND6p8LYzID97vJDKx30R/NZbgofvHXhkvUKwqU9g/mN8gPFL6e
UCHNrUNgf9+p2Xc/KKOsKKpm8LwOR7IpC+P/5uA51ZizRAbP0WPSjKwhd/Bcg77i/2PwXBM7
eI7aqkKXjDpyBs+lWhN6kw2e63Gkh6nhqloSla90UNqDgIHrK/R+O6ofi9tPrKHaz7c5O2BQ
nQuafur9ZP7kqkiOr6AKbNUrsv42vlqW7HiumuT4JKpPTn2H+Fot8sA8FV3xgDPlT1vNfJnm
o6bkvllUH5hWkWwAPFZbMgD+mvJHTdgOHdyeTNWBupL+P8rPIt18GCTn3fZ9Q4zgsvvJTJCh
cXl9DRWfIxQ+J/oH/CT+MT//C7b8XD9SVYfBgDhNnjrOoRcUVuVwMMN1jMWJZYckw2ba9Pia
r8guJH5kVGxCTELEgDqEYSWuwwykieR8K29Kn4JBNj36iJxPyqVi5FNv0qSussEtEbkliMJj
4kSRIQOGUDJqlRwb6ZXezHEwARxliqVB9I6IH0CqQ6IHjBTZ+dSh+mdEzGE7ehRP1HoYPQBJ
fewbHRIaRo3XhYmiYkLDIt2pwTYYSouNiRA3Iukrq7MG7wSyo0H0IVFIdEjkyCQy6EKqw0v5
TP9rzxTeCzQYb4HHf3ogtbesntEk/zMkLMjNnnwfaUGYEv7kfji5T0YGMYE8s5QQEFvJv3+S
5x4S1cla0oSMU1OiCUdIdOLwib4cST2ezjEi5pHnNnAsiQIy2Un6wgikYaEpHYCkBxClA6T2
9ECoKj0RzZR+GfOnNep0HS4dLE2gj1P+TSBgFb2ldF8d1XTbSh+nmih/0n5ScfKQoFbul/Tt
UXW+Ce2vDUeioRpOTTiS/jk+Pfgp/cXPvvTApqE4xJK+PyN6wNOE7v/bQA+WUslK+mKpKWfe
ZDgBv0fFHOT8vvnEerGERlRFg3W1JUNxvtKBxCxC5Ctv8uM8QtIooiLUjpPrK29QMUuFkPnD
VxXJ9vPp/Rbi2Yse310bLB7knOhbVW9YskFXDUm4KNvyNYgKGlF/505VX3n/On7+27b8Lu7n
GlET6FkasnElxlDTd6NE8saSxCNBqszxImqvkQu15+TSyMnZycGZmtbTKYZZh/EYA0HSMaCL
xAzSkKm/hjHyOraYFdi4nx6IklZg2ozeNy2aDMkKoCb9c+auZMHqSR5vT27d6AoxhHxigfTr
m3STujjSl1HiAaYpzHdbxqCS9FMvNboSENDhqknAUp+u9F9K60nAR4ftGQMH3Qj4WekQunIx
IiQfc0ln44yi/aGisaLOPFXyCusKOvMkP9es7MH7e0M91shQj/KzzN/7k9FSlZ8f6pF+Mirt
qi9Krmiox/onh3r0Kt2D9/0no9+f5WJ7/xohZRxzmAi3L2nWqiPxoUq3KjXkDB+pfzdewh4+
qiXWfV8XSH9MlSDGOjYusnbZ8s8NI6lX0TCSFtLal0aL9C1oEKM4kd6TKpR36Hw/pbGQOk2d
kE7leziw2o6ub1I8pK3h4lHn5oaUWcplSn+njXnEx+eg11p9Z6DbK0u5TOlLahuaL3oE+vTl
NVdZPrSUy5T+kobuCa97oF8T0Sao7JqlXKb0Z0tVRz++AfoGHV+PvvGnpVym9CeOfK0//Qro
9xMXjIoOWcplSl+88v0Np/OgPz53ge3ynZZyWTzlc8Jf066dAr3d+G8rJ22wlMuUvnDgI/fU
o6B/tX5DXuwyS7lM6Qva3HlZ8yDo2waFioLmWcplSp9f+9qSE3tAb+t11cRrqqVcpvRrNc53
HboD9HVyjk22H2splyn9itITKqZbQD9H9W2qxXBLuUzpc48c2rp7Peh5y80+akVaymVKn7Ny
T1j/VaCfb/bt9ad+lnKZ0s+ZUGCiuQz0mZMGxj/tZimXKf30gRuPrF8I+qGLrUfdbGcplyl9
ZptVI/zngb7azBc6590t5TKlT6+9pM6nmaBfWXtF7WNOlnKZ0qdo5FxbNBX0vXcKzhTVsZTL
lD6pdOZk74mgf9NUW2OXtaVcpvQJRya3eDIW9BMahpzeamgplyl9zMr059PHgL7wxF+1N2la
ymVKHzFhzCLnEaC/eT3LeONXC7lMudCBwzpfj0uRsfblatkbXltguW+bGCItCvQht8du3Vhq
geWg2oM21RoEevsTcyM2l1hg2V8jpP/J/qB3svHaVXDWAsudSoMM4/qA/pZl7KrdxRZY9j3S
9ZBpIOhn79dscqjQAsteK9sn7ukKeu7Jz6Gn8i2w7DbBu9aAjqCPKWrS9MoKCyy7DGx5WbMt
6E2fb9xwP8cCy45tmkzc0Ar0M6J7n3o93QLLDWo3dA1oyYh/J+c53AwLLNtp2D351BT0w1Sb
aBolWWDZtlQ0f7EDw560Pna1YyywbHXErEPrBqA/2mD9h+ahFlgWrhR8fVIb9FqxdUZ0DbLA
smCCzoYsW9BHzbu6dnAnCyzzBqoGN7EC/dn7R2ane1lgWaPNV/0SU9D3zuA457pYYJlb+31R
mgD0jZbMnbangQWWy9T/Glqbx0hvvXKWldhaYPnDw1LbUxqM+LxoO+yrqQWWXxXfPh/HBX2k
q6+miGeB5Wd5V9OFX5NlnL69Tm9vrgWWH44/57z3A+gFWffiB38wx/Kd8BMPB7wGfQeT7C4z
n5ljucTn0B9az0E/YFvMh8I75li+VGtP242loO/zbvrA0kvmWD6rXvAp4C7oX6fUXG500hzL
Jx5uWPO5BPSDagZv9NpvjuXi4pW9ci+DfvSd6MkJBeZYLsrL1fU5B/rA25OarVxrjuXC8dl7
np4E/bhxj/ZezzXHckH4zOgZR0B/N7zYXDDXHMv5PpNtmh4A/e6w/n7tJptjeW2t9DMlu0F/
ZPG3kPRUcyyvUB+TNnY76Jsm3+5SlGiO5dyHiQ52m0F/fYef7bfB5ljOKY6+d2od6Jv3mn+x
ZT9zLM/JGzg7fiXoDx/Tj07pZo7l6eP7tTZbCvo3xcSLA+3MsZwZHvR+7wLQTxpxqIemhzmW
0326rgz9A/QnI7eu7+xsjuWUWu0DtWeC/vjiBq/n1TXHcpK6t1b+FNAvTc2qfd/GHMsJD1vs
6jYB9Jffe/g1NjbHckyx85AvaaBfOTAvOlXbHMsRefaWS0aDfmhhvYyz5WZYDh1f+5TPcNAb
D3CfW+OdGZb7houSnw1l5N8+/stHPjHDcpCPWcOZkaDPbXFj05lbZlgOqCW43XQg6O/V676/
zkUzLHdS15lxIwT0m982u5h+3AzLvg9VWo3rDfoJqrov7+wzw7JXcdlrux6gD47raOS5zQzL
bnnvlp3uAvouqhO8l64xw7LL+BcBCR1A39jZe5xarhmWHcNL1czbgN65mdH5wXPMsNzA53bB
Pk/Qr9DMbHIu0wzLdrWuDgxrAfrJvVrkN081w7Kt+jmhTlPQD995uNXKRDMsWz08fiy/Meib
JB16aTTEDMvC4oNJ3euDfs3Ja7vT+5lhWZC3u15ZLYb9uzesft/NDMu88dtKllQDvZ/Lk52D
25thWSN8w9Q2lqAvN2n+8raHGZa5PivdnpuA3tLav3NQEzMsl9XM/WumAeizNB5dPV/PDMsf
1LJzXXRBv3jClpl+1cyw/OrBjC431Rn5q2v86D9NzLD87HAmN50D+k41Hmf76Zph+eGKcVvq
lI2Rcf2D156f55hh+U7G6NA/34O+mZZgVNAHIZZLwhKNE1+BvuBRb+87z4RYvtQ6utj8GejH
xE3tMOSuEMtnaw4cXvQQ9I1KRs79cFmI5RNq/ezC74C+5/iyGuNPCbFc/CDwqk4J6Fdw3342
OSjEctHhLpmbLoFe19zLYs0OIZYLV7Rr3uMs6C3Pn5novkGI5YIMr2dlJ0DvOmKiz+VlQizn
h7VYuLQY9PcXBvUYmi3E8trWzp3a7ge9QR2XvbzpQiyvqGlf/rwQ9KO7WSavSxdiOVetdv6s
AtCr7ebmdEwSYjnngU2I6yZG/AQ9M3oVI8TynMNCwa21oK826+aruWFCLE9fYXAwPQ/0Nzzu
Obr3EmI5M0M7oe4S0L/eqHnzsZ8Qy+lhKjXPzAd98NqQN3N9hFhOaV12MXEu6L+lqQ33aSHE
clLNd+MtZoBeh8eP/NhYiOUEtRdN908G/Z6CJRfX1RZiOebBw0fh40F/MvVWwQArIZYjDt/K
1k0DvbnOG4GVoRDLoSuutNs8CvTtcrSfX9EUYrlvxtkvPYaBfqB1UNs/vpliOSjs+LqvsaCf
5WpiF/jWFMsBrQ/2WTYE9Ft9kqdbPDHFcqeau/V8w0HfsN+5tDu3TLHsq7Zt34t+oM8JD+Su
uWiKZa8H62Nn9wL9ZI1eRoknTLHsdjivWrPuoO92qE5hq/2mWHZZsfjcLT/Qn/0o/CzYbopl
x4x54zLag77J3bQLD9aZYtk+bIZTPZ8xjPrmQMfCpaZYtmud+eCMB+g7BbpFzJpnimXbmuPm
DmsO+oMqfepGTzPFspXa6DaWTUAfsnHy7PbpplgWPkj4uL8R6N+FtVhbL8kUy4LDUasH1gP9
8xYPh+rFmmKZtyK8J68W6Fv2Unv2JswUyxoZwTpbRKAfQFhYlfQyxTI3LHB3oAXoO05er3mk
iymWy7y7RH0zBv2gtLabt7YxxfKHGu2sl/NBP3XOaKsVLU2x/FrV609fHdALNt7qOM/RFMvP
7jdP/UsN9Cde6raZVscUy6WHnBrPIUBvXLRcd4KNKZbvLG9wt9mX0ZAeT3RYOtbYFMsl6bVm
3X4H+ouG+TppOqZYvhRq4z3+Jeib/5XcYSzHFMtnvYXv6j0FfQujmEHjP5hg+WQNg7yzDxj2
L2o+YOpzEywXq2r3GH4b9B2EKzz+uGeC5aL7XE2r66BfNmao6vKrJlguPPRlx4GLoO/TJnLz
lj9NsFyw/G3EoDOg948N71R82ATL+enPzfVOgP7u6CYl1wtNsLw29OGJLYdBrz92R/DbTSZY
zvO+NTqoCPT59U+X6K8ywXJujSsNyneBftbRvj3sF5lgOUf17M3l20C/ok2P851nm2B5zv1j
09vlg97ad01AQqYJlrMOHfB4uQb0eSK/W/NTTbCcubzw1ZwVoE+54TO8eJgJltPTty5tngv6
bV1mit5GmmA5JXS9/50c0Lcya3GrxgATLCd556lOmAN6tyX+2wODTLCcUGPxtvpZoFdr+GLd
9M4mWI5RnRd+LhP0QaW1Dh1rbYLliPtZpiMyQP/HRWuORgsTLIcemnTUKpVRPrwuG9jGwQTL
fZePHXkwiWF/gxdlmXYmWA5KH1U3IhH01aNrHjprbYLlgNCE63qxoG+77Pp+C2MTLHfyjpqy
dTDoLx/s/HmQjgmWfWuEt+wZBvppV4qH7uSYYNlLNfhFeTAjfXKn1Od9NMay2/0ei1f0BP2T
sS/tQ18YY9nlkJ9f+26gP17Ufsze+8ZYdlzuy3nVGfQDunyxsLxujGX79Fab57YD/YJv/fXH
nDXGsl1o8wEtWoNeePpZrztHjLFs6+1kdNcd9B01tdR99xpj2apGg8MTmoE+0d1esHWrMZaF
qrWGNXAG/aTuV9Nt1xpjWXDfuvb5hqB3nTi1/+wlxljmHTK9MqIu6K9EXdioNc8Yy5rL+ZOs
azLKB3W3oWOnGWOZm67V7JAN6Hfu7rf2W7oxlssGcJ9GmIP+SMzV/smjjLH8wevLfH1j0GuP
aL+AE2eM5dfV33bYpg96zsZ+vScOMsbyM5XnX3tqg/5c4rGVgmBjLD+892ADoQb6ssIW43K7
GWP5zsGbwXnlo2RspD7iTeMOxlguWXaZ3+Ez6MMT3L8WtzLG8qVxZ/a/egv6rGN9VgS7GmP5
7IBjcX/8BfoaQ5e//NLQGMsnvQ5Ub/mEoR//9N78WsZYLq5eeOHufdBPv2qQ7m5ljOUila0Z
E2+Bvvhj2cWHhsZYLry3ron9NdCrOWXfnKFtjOWCgytKz18A/ablV5d6cIyxnL9s0byRf4I+
33FH7TcfjLC8dtwfvjbHQb92sXPc6hdGWF4xIOvzoUOgbzkuaMKAB0ZYzvWatHbwPtBX7+UQ
Wa3ECMs51cf25u8CfbspF2veO2eE5Tkqo3gFW0F/NyJ456pjRljOuhe/t9dG0Hdp/qZeXJER
ljMPRsZw1oDeulXhWPftRljOWBYmWrkc9Cf3PTrA32CE5ZRxfc92WAz6pV+2vHqw3AjLSQN6
jH2dDfrebXsb7ZtvhOUELz/HebNBf1nLrtGCmUZYjqnue7/ldNDn7AptN2aSEZYHq7Sac28S
6Lu/Ch/UP9UIy6H3mvlMSgd9UHjm9PbDjbDc96DjB/sU0F/42PiwS7QRloOW1V91YSToa209
olc3zAjLAeNqBiUlgF714u6hNr2NsNxpgLW2KAb0k05PeiP0N8Kyr5dp4eEI0Ht03pBt0s4I
y17V+ZFDQkH/tvWmCKGnEZbdVLSsDIJBP/ehKMLaxQjLLvc4pwuCQH9lw8yFdg2NsOx48HNy
7wDQPxy8TLdpLSMs2y9705DbGfSnXD8U+loZYdlu3LPbK31B/2SG66Z+RkZYth3wYEZHb9C7
trZ6PUrHCMtWXjdbvXEDfVpG7/T5XCMsC6tffjPPFfTBj3ZE7ftkiGWBypnlbk6g9xzyZUvp
S0Ms8+4d7XbfHvQuw9/2NnpkiGXNg/vVM+uAvuD+qOjWtwyxzF22a3vDGqAXlMe+SLpkiOWy
sVsGXbRm6Cfuurv1lCGWP/RfZzbKDPSOhT5d3hwyxPLrViuOi4xAf8NPx6PJbkMsP7NdNKpY
D/Qj09S2jdliiOVS7h/1I7VA31WzWcGxNYZYvnN3+g0DVdAPqLXN13ypIZZLDkyctv1bkow/
L89MjMk2xPKlpWnufT6Bvu6Vi+7HsgyxfHZs0kvuW9DXX7JzQ+2Jhlg+2T9+yaoXoE+L7Xc0
M8UQy8WtIrt2egz68jZlM18PM8RykW2Yytt7oF+6u9goONoQy4Xcvluzb4Le4yMRcCbMEMsF
d7uHuV8FvWHhm+4+fQyxnH+gs8mD86DX6PNX9f0Bhlheu7TtkczToH99uv1+jw6GWM4b6zmi
0THQj50/yPmwlyGWc/s3q3PpIOhNHq0b36m5IZZzWjleG7UX9MGJEwpLHAyxPMe2/uRqO0Ff
tDj6WkxdQyxncWu2OLIF9PODbz/WsDXEcuZdq+eRG0Dv69v/5XIzQyxnHDBZJFgNes7UtLc+
BoZYTlmq33nHMtAPfn+u7LmmIZaTxmoSfReBnvfstX4OYYjlhP6cTSrZoJ8cneHU/qMAyzGt
PoesngX6uaZNhnJeCrA82PaNoPM00O8Q7ji5u1SA5VDus4NvJ4L+hmtxxzG3BFjue/d+Qs44
0C+7bPul1WUBloMO3KjpkQz6qDt/XOf9KcBywNJLlx6MAL3r5ffvbhQLsNxp7J8TJseDvuUR
YZctewVY9u1/1KVxNOi92998M7VAgGWvVvsfXxoE+gMZDR9EbRBg2c12V87oAaDfYF1Wzz9P
gGUX7pb2tn1Bb+3R/M+WiwRYdry7tuxIIOgXz3x6scFcAZbtDyxfH+UP+kauhm2qTxNg2W7p
wr6GnUBf79S+OtbjBVi2HTtXf2db0OcHqWdYJwuwbNV/elFfL9A/767Sr8YwAZaFrSYOVXUD
vf6Ka/sbRguwLLBNs13jAvpH47dv8ggXYJnHTTrf2RH0fgtONunRV4Blzbtx6e8aMOqLEv/A
+O4CLHMPDHGebwf6lGnTrOZ0EmC5bEnoQ4/qoHdofXjqbh8Blj+k9fnjoRWjvrjYJv+xmwDP
Id3bThGCfk3XqCmWTQVYfubZ+VNjQ0Z9x51dp1tDAZZLq7Vdc5kH+mc9qk2fVVuA5Tscz15j
NEF//lnaocs2AiyX3HHVra4C+sOJdc5WEwqwfGm/w56jX0fC/NnCmbuH8gVYPrukXnT0R9Bn
ZblPO6IpwPLJtBo2Rm9A739sR8fqHAGWi0Oszux8DvoljaZxMz4ZYLnI0yQt+BHovZO7b332
ygDLhdX0HdTuMcLbYePgnk8MsFzA0by35gbo3XRbNjh91wDL+XeI2X5XQB/8ZY1K2+sGWF67
/5P3+3Ogjzdd++7IeQMs5y15/W7+KdBHCt5rdzppgOXctKd5nkdBr+7Tvv21QwZYzgm536P0
AOgf5cXujdxjgOU5njc0p+4Bfcx2h0i1AgMsZ1W7tNNhB8OepNDeyzcYYDmT8+fgK5tBrz/g
1lzflQZYzrhzxCJ5PejHtRtX/d1iAyyn7C86WX0V6NX6NNFeOc8Ay0lLdo45thT0uboPugbP
MMByQtpm+5iFoDfkjFWzzjTAckzI2ltG80C/SkO97r2xBlge7Lk8a9dM0G/gxh7dMMoAy6HV
Fnr2mwr6uCuHHqUmGGC5L2fua7WJoD8Toj8rKMoAy0F3pi1bOxb0PQP7/ekSboDlgP0TArqM
Af0br9MrrIMNsNxpSarah+GgNxmUaK0TaIBl37SRBQviQD8tf7jnNz8DLHuHxA1sFQX6UwvL
TD76GmDZzXOI8NFA0G9t1Hjlx1YGWHapFnpsan/Ql11s87m8uQGWHTl9khz7gF7YZYRQz9kA
y/Z3utW72gP0c/PKtarbG2DZbn+nkuSuoDdXF112q22A5epL2kyt0ZERP1tEE0NEBli2SvNw
O94G9F6vOtacbGaAZWGI618xrUAvePixoFBggGdPh1zjlqDPmzC89RsdAyzrVqvXpbAp6C+l
1rjaWM0Ay5qcGtwQB9AfFwYnDf/GxzL3juUW9QagTzKb3PjQBz6Wy4qMQ9fVBn32Nr6G8BUf
yx9y9Yy72oJeK7q3SvwTPpZfp2oUf7AEffH7m/YX7/Gx/KwfMXyhKeg7WvBmut3gY7nU41Nt
LwHoRywNdt54iY/lO6LXVx7pgv5mY/9qtc/wsVxCPJ00TQP0ixoN6pt3jI/lS7fvNXPign7T
att39Q/ysXy2qOTp1bIRUL420H6xazcfyydzLy5I+QD60i2ru/gV8LFcnHq6Y83XoO82arrd
8418LBf1O/Lt+DPQJz3pMGrGaj6WCz2KNsaWgr5GzLCuLZfxsVwg2tnP5C7oY/ssK3i5gI/l
fGKzwe4S0Hc8HZW/di4fy2tvrzkQchn0iWPi2kZn8bGcV7QsXuMc6C/m+U1wyeRjOTd3QY31
J0Efc291onY6H8s5qXMudj0C+mVqtpb3x/CxPKfftPEf94O+/G3fcYeH87Gc5TGh6aLdoB88
SXPrhqF8LGeKUh95bQf9ybCjBYuH8LGcQYzMfrwJ9PXP+c6eF8bHcsrtoe2mrwN9izmu3bKD
+VhOKhr8xWkl6ANOteYuDeJjOSF3wLprSxjp/6jV8s3+fCzHpPbuk7oA9D1vDvU60ZGP5cH9
uunV+gP0A73ePHraho/lUI9O+07MAP2le/4LjVrxsdxX1CZ26BTQFzb1iWjdgo/lIMKjmukE
0NuZjemW3ISP5YDbLud2pzHiP3vt4H2N+FjuVNR4XP/RoD98IX2TZj0+ln1z6zppDgd906OF
Dj1r8rHsnVr9wfqhoHeprvJhiw0fy279LOf6RzLKHwuehok5H8suHsZtPoWDvqH75CGpRnws
O4r0Pi4KAf1VH/sGb/T4WLYnNFZ79wb90ocrAqK1+Fi2u10e9KQ76K0W5z55pcLHcvWij9pZ
XUBvY/HHX1/K9LFslfuq0LkD6NsMX65V87U+loWpTyKv+4C++Y3dTsGl+lgW9LtnleYJerNn
DceuKdHHMs+j5HStFqC/96atisY5fSxrii6mnGwC+mtZnY4lHNHHMpc43SiuMegPtR774M1u
fSx/vVV8x7Q+6F2NRRHpm/Wx/GHfvpl7aoH+pVnfsFqr9LH8evEOrwHVQG9tHfn06kJ9LD9L
2fRW0xL0RxvH6y6epY/lh8FrVmwwYdSnHnOuJk7Sx/Jd92XdAwxAX235swF9U/SxXGKzQOOz
DqO+K5y8OzBRH8uXy2fvWKwOeodRo9+GDtHH8rlbUyNac0C/9us+y3Eh+lg+uW+8+dMvw6E9
atLJZ0sPfSwXL045kfUe9PXuGY1911Efy0UpI0Y3eQX6+d0+3vb11sdyYfDQBiVPQe9+5Gx8
fjN9LBe4D76Z9hD0uukx7es01sdyvs2A6bXvgH5Gj9kjttbWx/La8l4ep64PZ7T3P+h0tdbH
ct6tgFdxl0Df0LmVCddYH8u5+zouFZ4F/ddmhqsO6ehjOWexj//eE6Av7P/5bDZXH8tzUtxV
Q4tB31SYtDztkx6Ws4JdtmntB/2EseqNRr/Uw3Kme+PwjYWgz9VRy5hUqoflDJu6pt0KQO9U
emLL6pt6WE4ptz36OR/0p+ctOVlyUQ/LSbcsRuauBf3skbtv2Z7Sw3LCPqO6Pnmg79Cy1ddR
h/SwHLOYd/1pLuhPDGvj/KhQD8uDU9SnzJgP+k8Wf/4xcIselkODy1s0nQv6tlrHnT6v0cNy
X/ePz0uyQL8n3cQyd6keloNsXi0aOxn0IUUpfYJy9LAcUP64s9140Mf13qNZa6Yeljvdukuc
TgV99cdxDdQz9bDsu+/6pvhRoH8zsOH1T2l6WPZefKG/2TDQLzoZWY2TpIdlt5RThvtiQU8k
T9e3jNPDsktw8aHQIaDvf+nB1naD9bDs4L4vUTsc9JuXrRRN7a+HZXubHbXy+4E+c+W7kHs9
9bBsV55/uVsvRvyYXJ3Q0V8Py9VvrZ74pRvov5l5LTnRXg/L1vuWui7xA71Pe909fbz1sCxc
PP+JT3vQT2n69rFKSz0sC1Jmz3/WmlF+luQ22eush2Xd4KkdZnqAvqjOok1T7PWwrOE+/mvT
5oz6YkyfiJjaeljm2qRsuOEM+rk1nSPDRXpYLvs2PHhcI9Bb3A4+OtRMD8sfb8by69QD/dTA
sskzBHpYfr03Yv/pmqB3WK17+LCOHpafLeoflyACfcy1xDQ9NT0slyb3qm5uAfq6A8pPD/nG
w/KdvgEX9hkz9Ku7Ft74wMNyiVvHjDA+6HekffYPfcXD8iVrnyY6OqC/vbl/XvkTHpbPfnMr
zVcD/ZaEy/s23udh+eTNpvO6E4z4fJO+PuEmD8vFexv5ln0eBu2RM8NGd7rCw/L+RXU+L3kH
eodVf7i2PMfDcmGy7do2L0E/dkb+K4+TPCwX9LXo/fwJ6L2XD9oUVMzDcr6bEW/Wg2GM75m8
MyYU8bC81pq31+U26FP3rxh5fBcPy3nf1GJuXgP9zZXHc2y38bCce/ObTfpF0O8edert9I08
LM/f++FMnTOg96+1fJZgDQ/Lcxe9TPvzOOidC2zGrVnOw3JW8mOHxMOg7/Xo6ekei3lYzux7
9555ESO8m6aNNc/hYTnD7frsol2g33Z84OrXs3lYTrW+0Dp8G+h7u+Z3uj2dh+Wkbyff6+SD
fuatkWPuZfKwnHDz8MpNa0D/QGOjR1kGD8uxe/cG9lgB+vVvDZbVTeNhefCi7VpfF4P+ZEyL
jdGjeVgOTc7ftTQH9Jd6LY45OpyH5b59Vw9pO4fxfH3Gvmgaz8NykNtSyxfTGfH/mdtkTzQP
ywHW80/NygR9oNDYL3AwD8udvs1Kds0AfYuVaT5a4Tws+96c0vBWCuht8x/angnhYdlrb8bt
9CTQt71Y8GJdHx6W3RYlz6ibCPrIZy5bFgfxsOySPLzVmRjQH8iZMHJVNx6WHfvGvkkcDPrn
r7LaHenCw7K9W8RyizDQ39YzaVjWkYdlO+v+3fYHg75855qGbdrxsFz9W0/1gT1B31RrW8Aq
Hx6WrW76b9ftBvqBKlF5Nl48LAv3dhi0uTPoN7cuqb/enYdlwaLWZoHtQB+05sZffi14WOYl
ux3/6g36lFeWH7RdeVjW7Nt01DJ3Rn00xKTddWcelrlujer7NgP98RUmL/c78LD81arOjRdO
oL9i2uzl3oY8LH/4Wm3a7IaM9HYhssu5+jwsv75h7t6sLuhLmg02L6vDw/KzPYYvb9VgpIc1
u7u0qM3DculC3SUZNoz6t/merzNr8LB8d4xa13rmoH8RM6sGYcvDckmfb9yzRqAvfed0cpwN
D8uXWn7YMkwf9Df6hH22suJh+ZzVy1BLbdD/cW3b1tPmPCyf/PrI+IAq6LU5GcRcIQ/LxTfu
FA8sT4TyO/Pb3XgTHpaL9lwbzvsM+gtmFiGDjHhY3r3wvN2Wt6DvSOSmxAt4WC4Yc/Jq4F+g
99K08ZrD52E5v8/hzG+PQe/i/n7jKT0elte23Nt8+X3Qxx7sfNySx8NyntX2Z763QB84q9rc
sTo8LOd+3bjwr6ugXxXcwLRci4flnBurOs25APq+wUSXmZo8LM/Zs6S82Z+g96v1xqeFBg/L
WQtz8m8fA33L5DplZWo8LGeOmRUy/hDoN3jOjL+gysNyRp8pgvr7QP/CtXTjARUellNaZhw8
uxP0b7+t23KEy8NyklVywvCtoN9x1SHtHoeH5YSvw2pabQS9yuqZ5iaM8yjH3Ii5dGA16NWO
5acFEzwsD94zaMKg5aDfWNZ8x75yXSyHLgxx0VsM+lo91+9rwjiPcvCYno+3ZIO+umvb7EPf
dLEc1Mc/J2g26PvUMPeJYJxHOaBlh/bl00B/6eTn4hqM8yh3smpdtnwS6D+XT7b88FUXy75f
W65vlw76gFzXNrcZ51H2vtGk78tk0PdCzqPstqeh/tyRoFcnprDuj7LrQrui5gmVD6/TmGpD
70SDfggSfyjb9zG3nRABenfk+aBs19LwfP1Q0Dshzx/l6la66ef6gv44kr5Qtv6q6jwiCPQ2
SPpFWXjj6wOrANBbI/kDZcGe93MPdgL9OST/oay38K82Eb6gtyxn52+UNcc8+qjnDXrbZuzy
A2Vunzurt7oxni9SPqFc1uJaz56uoB+MlH8of7A8r0M4gX4qUr6i/LrsxO4V9qCfjZTfKD8r
ORTVvg7onyD1A8qlu/dYv6oO+hyk/kH5zoKCP+dag34nUr+hXDJ6Y2oLM9CnIfUnypd6r2p8
1xD0s5D6GeWzLZbcnaAH+mVI/Y/yScucWQ20QF/DnN2+QLm4bKb3eRXQcyez2y8o7y+Z/G7E
twRo7yPtI5QLd6fnWX8C/Vuk/YVywYIxPQ69Ab1nX3b7DuX80cM0B78Avd97dvsR5bW9Y3bq
PwZ9QCy7fYpyXotBg7fdA/0ipP2Lcq5liEWvm6CPXctuX6M8vyzoJHGVYX9zdvsd5bklXcfk
nQf9LeT9AOWs3e3tO5xmhFfIfv9AOXOB961XR0Hvncd+v0E5Y3TLrD8Ogr56JPv9CeXU3k08
W+4Fvcpr9vsZykktGr6+uwP0T5D3P5QTLO2WTdwCeh8f9vslyrFlogD7DaD/gry/ojy4xEzt
wirQn0bej1EO3S0oGLkM9HN3sd+/Ue67QGegzSLQJ+mz3+9R7jlaVXh4Huhnvmb3H6Ac0Pvr
0cGzQB82n90/gXKnFu9H8qeB3ug5u/8DZV/Lv+oWTAT9K6R/BWXvstLrvcaB/irSf4OyW8nt
KZxkhv9I/xDKLruvtlw5AvQ6Zuz+J5QdF5x70SEe9EO+sPu3ULYffWLx6yjQv0T6z1C2633I
b94g0Gf1ZvfPoVy9xR6O2wCGPpbd/4eylWXB5nt9QD/iHbt/EWVh2YYBkwJBv06T3X+JsqBk
pVFDf9B3v83uH0WZtzv38IWOoK/fjN3/irLmguxhSW1BP/UEu38XZe7ombVFXqDftJndf4zy
116TrxxuCXrXx+z+aZQ/NE+fNMQF9MLt7P5vlF9bjGlm4Ah6x9rs/nWUn31JfFrQAPQrR7P7
71EuvR69oLcd6ItXsccHUL5bOLAjtzro4w6wxx9QLpnf79tKK9BfHMMe30D58qigjR2FoG+2
gj1+gvK5Xl37vRGAPm0me3wG5ZPN2xtk8xjpZzV7/AflYgvvA26aoJ93lj2+hPL+Ly3i73NB
3+Qte/wK5d3XnWtkfo2X8fZE9vgYygWF9hcbfgT9iy3s8TeU8+fXHn/xNegPj2WP76G8bpSo
6ajnoHdcwx4/RDmvl9kj0SPQe4ayxydRzm0uyC6+C/rM6+zxT5RzLHTaRd4Affs17PFVlOd8
UflicAX0a4PY47coZ10vW7v9HOib32GPD6OcWfiud59ToD9Qiz3+jHLG/Bc8laOg75/MHt9G
OWVU6d5VB0D/oC57/BzlpF63YzrtAf2XG+zxeZQTml8Vvd0O+iMu7PF/lGMszp3N3gz6pA7s
+QUoD/5yfKz7etA7WbDnL6Acev2g44OVDL2QPT8C5eDC3fczl4L+2Cr2/AuUg+Zvm9NoIejf
LmfP70A5YNQGn0t/gH7NZfb8EZQ79Vr5YdRM0PuksOenoOzbPHdVtamgf3mKPf8FZW+L7KAj
E0DvEcGeX4Oy25cZ2lFjQT/8CXv+Dsqu1zMLBWMY6aEPe34Qyk6F4yJ3DAf9rv3s+Uco288f
bdU3DvTVxrPnN6FsNyrxtEoU6Ndps+dPoVy9V3TK6oGg72HFnp+FsnXzgY069wd9kxHs+V8o
Cy363XnbmxFeN/b8MpQFXwJn5vQAPXcUe/4aynrXu3h5dAW9Sw57fhzKmoXt3j7oAPqVj9jz
71DmzvdaMbkN6I157Pl9KJcltejeuBXoVdLZ8wdR/tjTWeNyC9BvNGPPT0T5dTP7HaObMsIb
yp7/iPIz89oRtg6gT27Bnl+JculnG/Oj9UG/rQl7/ibKd68JT0TVBn1Hc/b8UJRLdhmMNrQF
vSiIPf8U5Us52g12WoJ+QQZ7fivKZ5NUbvY1Bf35o+z5syif7Fk2TVUA+gY92PNzUS5u9s59
jS7o99xnz/9Feb/5i5edNUD/h5A9vxjlws8Pl7zjgN67nD1/GeWCa7e6zi+Lg/CPZs+PRjl/
1xUVzw+gT9jDnn+N8tqcs1sfvgL9gRXs+d0o5yUdD5vyDPTHPNnzx1HO7XnQxKGUYY8je346
yvOb7T5y+Q4jvDbs+e8ozzXfNmJMCegXWbDn16Oc9Xl9neqXQV9qwp6/j3LmtbxrR8+CPrkN
+/sAlDN2LZ4cfRL0c2ayvz9AOTVnXgujIwz737G/b0A5KWnG8537QX/lOfv7CZQTe2YuCt4N
+s832d9noBzbbFxnte2g1xzJ/v4D5cHmo4m1m0B/15L9fQnKoZ8TNvmtAz0nl/39CsrB16L6
v88D/cRS9vcxKPfcFW64YAnoi9qwv79BOSAn+JDnAtBbeLC/70G5U1JgYulc0A+yZH8/hHK7
nl1qTZ0B+ho12N8noezdrN1lhymgr3OM/f0Tym7mXhOvjAf95ovs76tQdvnc3DU5DfT1ctjf
b6HseM3pSfXRjPgxZ38fhrL9rgbzjw0D/UYX9vdnKNvl1OoQMxT0x++zv29DuXqSzVejSND3
8WZ/P4eyVU/hhl3hoA+4xf4+D2VhM4PgfiGgv3mM/f0fygJzbb56b9C3P83+vhBl3mfu/rXd
Qe8yl/39Isqa174M7dKFkR7Os7+PRJm7663th/agPxHO/v4S5a/Zz88v8AF9RCb7+06UP4x8
mN7KE/Tl75DvSRF+HXTL+VFz0K9UZ3+fivIz1ysPpzYBfex99vevKJeanf3DsTGjPFzJ/r4W
5bufjrW9Wg/0o5PZ3++iXHL1wKfkWqDv+if7+2CUL+8sXFOjGuiH92V/f4zyueytvY5bgL5h
LPv7ZpRPjlyvG2sC+nFP2d9Po1wclLfH2AD0fUezv89Geb/r4uhCHdC/28r+/hvl3WbzbELU
Qb/Nnv19OcoFn7LOqHMY5fMa9vfrKOdfnZS27stQmN/RmP19PMrrdo516Poe9G8c2N/fo5yX
Pereh5egz1zG/r4f5dyRCbMXPgV9sCV7/QCUc4KiWns9BP21D+z1CVCe6xr+/tFt0NvEsNc/
QDnLLHjltOug31jAXl8B5cxPPQKdLoF+ljl7/QaUM676aV07A/pHZuz1IVBO3em7K+UE6F+k
sdefQDkpu9WQmsWg3z+Rvb4Fygkjm1ueKAK9fSl7/QyUY4KcTsUWMp7Xa/b6HCgPdm2QbFIA
+qdb2et/oBxqVqvh7nzQt9Rgry+CcvAn69sha0G/YyV7/RKUg66aztDIA32zruz1UVAO2Mlv
tT4X9NUus9dfQblTttabrvMZ6bMxe30XlH1Hcpd/nAP6V4vY68eg7B30JWBRFui3bWKvT4Oy
m+tbNe/JoHePYK9/g7Kr2fOCxxmgt2zNXl8HZadPDwZOTwX92CD2+j0o21+9KXQeBfov/dnr
A6Fst/PysWuJoF98lb3+EMrVs88kpcYy4l+Fvb4RytYjj9WrNQT0lzTZ6yehLAw6UHIiDPTe
XPb6TCgbuhZOHdoP9Od57PWfUNYz2+pm2gv0Tfuy15dCWfPTur92d2Ok//bs9atQ5l5dkdvf
D/StQtnrY6H8dceiLprtGfllFHv9LZQ/zvuDu6E16FfuYK/vhfLrEVlb/D1Ab72KvX4Yys8C
J4V+agb6Fm3Y65OhXOoy1nixM+iXGbLXP0P5rnBUsXcj0G8SstdXQ7nkY/zwJ3VBn13GXr8N
5ctXIu2yaoI+icdeHw7lszvCrjqLQD+3I3v9OZRPzuubed2ckR9T2OvboVw8okfzNGPQ32rM
Xj8P5f2Bfs9q8UE/+zh7fT6UC118F57UZqT/Gez1/1AuELbqFKfGiM/d7PUFUd70sVm5KQF6
zeHs9QtRXnvFMX/P51hYD+wFe31ElFfsqB8y4B3oGwWx119EOXdeTYHWS9D3UGGv74jy/BHW
Bzc8Af0rf/b6kSjPCTRNCHgA+maX2etTopzlwq/5+RboR/mw179EebJQ69Lia6A/Np29vibK
GR85E1pfBP2UG+z1O1FOufK56dM/Qb9wIXt9UJSTdrx5lHUc9E4T2euPopw471l2k8Og753H
Xt8U5ZgRD9qV7GPEZyB7/VSUBwfe/JK2C/REL/b6rCiHulxeV3sb6MecZq//inKw8EyfUxtB
n9KMvb4syj0/HtWLXwP6v2ax169FOeDK/n3CFaCP82Svj4typx27YvcuBr2KiL3+Lsq+87ZU
C80BvdEE9vq+KHuPWHdOaw7o53dgrx+MslvginEbp4N+zlH2+sQou7oscuqWCfqpV9jrH6Ps
JPzjwed00B++y15fGWX7j9Pn5qaA/ssV9vrNKNtdmdjGJwn0Ls3Z60OjXH1H2senCaA3NGev
P42y9byk1TNiGOlByF7fGmXhiPieTQeD/mEMe/3s/2PvvuOp7v/A/yNJRqTiLCuF7JGRJNmk
QkY7kr1SKjSsSkWSZFSE7JmVkUQoJDNbyigjIysi9bu6Ptd1Pc85316/v7+32/d9brdzXd07
j3M4633e7ze9n9Red8CR+f1J6O3GKY/PTW1WRasS3+PQv56nPP43tRlxx5yED0FvEUR5fHFq
0y2Y8tQbQ5+sR3n8cmovt+9rOLMP+kVzyuOjU3u+QMcbrwc9fyzl8depPR2+S/qFBvTPz1Ee
353a4+eV+k/uhN5zhPL48dQeMpO9y6RE9nw9pDw+PbX7FMQ0nmyF3rSR8vj31O7h2jxnIgm9
wzHK4+tTu32eO3FpC/TuJZTH76d2UzunWewmsufrO+V8AGrXFbAxavNCv7qUcv4AtV+HMxaN
4aG30aWcb0Dt8vO09iHroR91pZyfQO1nZosERTbow+Mo5zNQ+6nCzJv3q6G/20k5/4Ha2Vxj
F/3oobdKpJwvQW3TyZrDRj9d/nODEOX8CmrrZz17HjUD/fPVlPMxqK3qksE7Mgz9Z1/K+RvU
lpd85LO1F/rdRpTzPagtNhk84NUC/aMSyvkh1ObL8tV8Uw29zw3K+STUXu/ilsRZCr0fjSvF
/BNqM0larz6RC73wZ8r5KtSmmTRzyEiGPs2dcn4LtWczdd8uREGf7Ug5H4bao87bJTXuQt8y
Qjl/htq9EuJ3bl+HfvYa5Xwbar+b4JnpugR9hhbl/BxqV2eymwiegd7iLuV8HmqXOtMVuNpC
r76dcv4PtXMkZnHPj0J/1JFyvhC1kyc+eTLuh97gCeX8ImpHZ7b37NeBnmWYcj4StUOca1Qe
7YDeUZty/hK1r0k8ixmVgb7IgHK+E7UvTqTTyQtDv6qRcn4UtU9nRp/04Yb+UhvlfCpqWzsH
v6rjgD4un3L+FbUPSfgK4xihN1yknK9FbaOJMzctl53h9WhDOb+L2lqZVmOZ09C/m6acD0Zt
JWezvYtD0BNKKOePUVtKQveJ5nvozZgp55tRe9OEEsedZuh7dCnnp1GblCnm1vMaer5lyvls
1F7jzNMm9Bz6feWU89+ozSDBrngmB/oodcr5ctReHKe9X5pE9vgrU86vo/bXjJml1VHQWzlQ
zsej9qDTp6MmIdA33qKcv0ftDvH2FzH+0McfoZzvR+3G8Wr+sYvQl12hnB9I7ZcZxX4Kp6GX
iqWcT0jtYqf0T7420G+Vo5x/SO108Wjt+iPQKyZSzlekdvz47RS8EfQBcpTzG6kdkeHDbKUN
/Z5FyvmQ1A50OuP0RBl6zm7K+ZPU9hW3aliShj7Ln3K+JbXPjZtKawtBn3macn4mtZ0zdO6G
kKDHv6Gcz0ltcyelufdrof96lnL+J7UPiIuZbVkFfewqyvmi1NYf5y5y++EE+yOeU84vpbZq
BhuxbAr6I+cp56NSW96J9hLzEPTBTyjnr1JbRHym17QH+kgPyvmu1N44Pqga1wT9flfK+bHU
Xp/RFjf+CnqdUsr5tNRmcaqm31YCfcMqyvm31P4pVmx9JRt6RxfK+brUnh1Lq25IhL7mBuX8
XmqPpkeJEB9CjztAOR+Y2r2OtwOt70C/chfl/GFqvxPzmci+Bv2H1ZTzjaldPXbaYPkC9KkN
lPOTqV2WfjJHxxV6URpLivnM1M5xNF0fag39nBHl/GdqJ4vpnPtwmOz1+ZJyvjS1o8e2dYgY
Qu99hXJ+NbVD0kWVzmlBv52ecj42ta87cj8s3w69QBfl/G1qXxRj+8kiDb1TFeV8b2q7jdGY
HxCEvjeecn44ta3Tp8sfE6EvcaGcT07tY46DApPs0EdJUM4/p7aBWNtVJQboXWYp56tTW2vs
9dDVJUf4/cd2yvnt1FZOL9Jt+gr99DDlfHhqSzmmpZE+Q/9IinL+PLUFxaJYbbuhb42jnG9P
bcJYkEtuI/QfCMofNllyIs2R7t30swp6g/0hfLOOnEjTO56W1XsGPc8HrfOvznEivSh68t69
J9CrbzT48MCbE+mJLybzHxOgL9VLNXa7yYn0YJr2QbEH0KvpmrXsC+VEusth27PzwdBL1h4z
k4jmRLpeVJS74ir0eyXqe9iSOZF++YXkteYC9Bl+hcdnszmRLkxb03fwFPRXL8v3dD/jRDrT
gUY9wQr6KVfrva+qOJGOE52O/3oIehpdx5zcBk6kI74MMCgbQL/Ly5U+vpMT6aC0Vlt/Tegn
8Amq4QOcSPs6vK5tVoLeP2a75a1xTqTPiRaJ8UhB/36Di+21eU6kHb+kBtltJnv9f72s70vL
hbR52sOveQToPTc8YfZh5kLa1CHIiIYd+oC3+nG+G7iQ1hf1ztu9EvqS8ngWf14upNW+uHKG
LzrA73NNs2gHbeFCWj7N0r1/EvqnTz7siZDlQlrMwaRL/BP00uEXuRN2cCHNJ6qt7NEFPYuf
Tk6eNhfS678oRlc2QL8QmMH42pALaaY0ERr2KuiZ6IjcPYe5kP5pTzpxuBj6oSMrx2asuJD+
JrKmMjEL+lCVz65rTnEhPTr6a/N0PPRRNsppop5cSH9MnfLfcZ/s8ckOCN19hQvpd/YDI9dv
kz2/K4+KOAdxIV0t0rr73RXovx9UsbsbwYV06eirDF5P6O8mvdzzLI4L6ZzUQjYHF+g/DPi8
+5TOhXSyfarr05PQey3iFjkKuJCOFnnYQnsI+uWqLc/UyrmQDh29JbdnH/TbVh9ef/YNF9LX
U73CIzSg17Q9QJfayoX0RXvX7wPboO+2aPbp+8CF9GkRy8OSktBb6Vy+TRzlQtp61Pi55ybo
d23nFD4wy4X0kVQt3ld46DfwHdQJ/8mFtIG9os9aNrLH8wHTeAcjDmkdEZGBI/TQ17DMcHCv
wyGtPErUTP5uD/v7fT88teTGIS2Vypo0MwG9Y2Fsa7oQDmlB+1+MOwehL1zD7rggjUOaIDJl
f7MT+ky/lee1lHFIs4/217XWQ782w2ImTAuHNH3qOwn+SugTsxl7RwxwSC/avQp2LII+UG1A
dudhHNJftxROF2RC3x7aOxVmhUN6cCTFeEU89PWHxtZPueCQ7kp58HRvJPR5z+nu7/HEIV1v
dwt3Pwh6GTPS5fQrOKQrt3h5fvKDvkpEsZT1Ng7pwpFTPVIe0Ec8OmjmGolDOjPlhMpFZ+i/
eFzXan+MQzrOzjjmtSX0ze8ar+/MxCEdtkWLbt1B6MuI6sTUQhzSQSMKJ4/thd6MfnaRswKH
tG/Kllcp6tCvuf5j07W3OKTd7YjCc4rQD7/3CJtvxyHtuIX1pqoE2fPldkvfoR+H9ImRn18C
BKB3Gj2i0TeGQ9o05euedhz000wcFw7O45DWs+vP2riG7Puv7BpvocUjrbrl3VrnFdCvFByJ
NGDBIy0/UnWmaMEOtoeMz1xs4MQjLZZS0Eo/Ab3ZjdwQA3480nx2KQoGA9DrrR7ubBHFI825
5UHkgw7oW3n37zsoj0eaaSRw6fNb6Etw279+VMUj/TP58lGZCuiZlUaL7HfjkZ61PfXiUiH0
2qWZKd9M8EgPC5/gr8mA3rK+q+SKOR7pj8P7/dY/hn7qQuXX9Q54pN8la346HmFH9u97q9SS
zuKRrrVV0E67Bb3uUeUn273xSJcKb0n55gv9Caab8s038UjnDROY1dzJvv/t61vs7+GRTk5m
cbrlBP2EvIbfyhg80g9tf9Z3nICencFfIy4Vj3SI8FepTQeg53bbs35XPh7p68N9IS57oFdn
YPna9wKP9OXkltliNehdvym1XqnFI33atsqUQRH6GHHT8i2teKRthQsKDcWht1B5n934AY/0
keFkQtRG6Dcd3x/vMYpH2iD5/sVhLrL318mdYZvm8Ehr2Qb2yrJCL/Fc9GrTLzzSSsKXVb3o
oLe1S3T2ZiIgLTPsElc7bwvrowuqBtIbCEgLJlvQc45Db+kbLDTIS0CaZLvf2qIf+lux4tMR
IgSk2YU1q9PbyXrnvIx9cgSk6YflRRbqoN/jWWnGqEpAejFJOFD9JfR51TQTL/UISE/YECaC
CqC/+YbN2cuEgPSgEItBVzr0tEcftO4wJyDdNbScvTkO+ofFBht/2hOQbkyaXOcaDv2i10fD
srMEpCtt+s6WBEIfGPvT4oo3AelCoZb2Vb7Qe9ucMNALICCdOVS5bf956BNsXxE4wghIxyU9
fRDtCL321eHS7hgC0hE2ycsjFtDz9N9QSkojIB0odP+4nBn0agPOAW5PCUhfGQoo99aHXkro
eJZ6OQFp96RLAnW7oC/y2Ry/vo6AtKONy1UuBeg3zt+2GWojIH1CyGLohBj0QgUu88/6CEib
DhnpZvJDvxYXaRgyRkBaP0kj7Tsn9F13Bs7YzxOQVrWRZ9VkgV6/aMsRDToi0vJCwi7BtNDL
btdl5GMlIi02hG/q/mYD86naN55b5iIivTGJWVZoDPrXR+4/7tlIRJrTZjn0dB/03+LCgp6L
E5FmEpr89rwNegF/WrkYRSLSNEMfD6yugz7i8+vQK+pEpGcTm4uNy23IjlfRmWa/l4j0qHUl
KeYp9K2uW9yNDhKR7hV8evlLGvT3JDPmt58kIt38OemjfCz0R/2OCgu5EJGuTYxU8w2DXpVB
nnG9JxHpUuuA+LcB0H+Xk7tHd5WIdJ7gJQa8D/RHTh15O32biHTyZ2fbk+egH5jIyhy8T0Q6
OtG8NssB+m0v5RU6EohIh1gbiS2ZQ9/0dvHk2ydEpK8JagRpmUJ/rIJRvvIZEemLn+W+3tkN
fSrPqeSSV0SkTycKGb1Xhf4Xn3rx0yYi0rbW+DxhebLHvy7MLqeHiPQRQWZON1Hopfp987KG
iEgbff5x/gUf9G0d/JGZ00SktRInOpk4oU97H7wha5mItJL1x+2mzNDfeDsplM1IQlpKsDkq
lgb6A9IW9XnrSEhv+lzxa2zOGj4vtzGtKuYhIU1KzLdQ/AK9yDOOxrItJKTZrZMq/D5C77Yl
QahmKwlpBsHIzQ2t0E/fnWBu2UlCevHTTX/CG+g1Ynku9OqRkP6acHHEqgz6QwE2Z76YkJAe
tHLenZ0PvQPx5/R3cxLSHZvNM36kQt+bgF9Y7UhCuv6TIZtODPTWDLPexPMkpCsT1F3v3oN+
4UFNgIQvCeliK7mW3pvQz2h0cqrfIiGduVlITsQbesLhk3wHI0hIx3/ChZ89C31FVmbiqcck
pCMSmL6X2UP/w/9n0o1MEtKBVj8OsZhDHxXxcGN8EQlp380TJWYm0N+le7e+rJKE9LlPH3ge
60FPs2/64vsGEtLOCU3eEzuh93DQPviji4T0CauK/m1y0Jslbk4mfSYhfWBzvsZVEei7U/ts
VKZISOt/Skxs5IU+4lJzmMUP0v/hGZrgZ7+nvf71jvznXMj8zxo5DeUJM2bMmDFjxowZM2bM
mDFjxowZM2bMmDFjxowZM2bMmDFjxowZM2bMmDFjxowZM2bMmDFjxowZM2bMmDFjxowZM2bM
mDFjxowZM2bMmDFjxowZM2bMmDFjxowZM2bMmDFjxowZM2bMmDFjxowZM2bMmDFjxowZM2bM
mDFjxowZM2bMmDFjxowZM2bMmDFjxowZM2bMmDFjxowZM2bMmDFjxowZM2bMmDFjxowZM2bM
mDFjxowZM2bMmDFjxowZM2bMmDFjxowZM2bMmDFjxowZ8/+7bqVZ89d/mf46q7ucdXY7Q8NA
S3ubRvUvM/x1/vXXiYHsGkz//HkFzf954vnrPEgPDqah+/v/bKo3/vlTJM2/FzOqlnv//ruV
NKv/vp6us5vV6VMujhZudi7OfC7WfBoWbhZ80krqZ0+fs/rrqwr/9UWHV/zv/N+JjgY7/XP6
62n6+zH6fWKmkaKDZ4vpn2fm9wMX/c95Bjv/X30W++d5m/nDM83413loxf/ehnyxq/5+h/57
mfhf50d/XfCnv/v39M9V/779353qGbq/HURj/M/f0P7z/98nHnh9/X36+dd5NdlygPpE/0+7
guw6LDQ4ssLwn/PfX9uLhuZ/l9L+/15Kh7iUlUaK5t/X/P/umeoZ+H5Rf2bzVv7nHvy7LKT9
e0lEQ2P913n1P6Ynu5f/tv8ugVb//SzI8LnZWrlZ0LDTXFP53f2+5Hct9N99Xa/6r6W5Nr1W
WgP2KGTO9CKB2z+OPqsXAatlLM4LbQPrHfOwC9AGVx5O5fpuAj6j2Lba9SQ4L19Pe+Y0WOr1
oXYvH3BQ2LFyzmCwsH8uS2E0+OW2kjLLDLBG8MdBfAnZ9599y7urFlzPIv8goRO87pih8oVh
svuTfcTh8DxY07pLUothw38u32p0Z/sG8KCkYfj2TWC5WjtdLVmwZads1mE1MN99/cYLhv/z
79NMrmleojkYt2PxRI8L+GDp4ADJCzwtX6NiGwR25TO/UBoFjj5tE8ubAT6RFFcYUALOKe2q
pqsDe6n1dl7pBsfr+cys/QJ+7x3FnboIfraJw3wPE+d/fsRW+XIJD84rjNqTvwU8qXib0XMb
mGNf0JKuLviJZZjE5oNg2VvJKcx24OG7b9yW3cEs35gjFq+Du79cIK6IBCc6yK7iSgEPihtY
yheBDbPnFCxqwPQ799wK7wTfmbjq0DkCFkl91yW4CH4p4zbgxcT1nxU3JdwaJIAvf8/oMxYF
v2bvHmrcDi7Oupx8QB8cu2pKdOwI+PG2YM8AJ/DyobKH8pfByh5f7o8HkX29aJ9LTx6B81sX
9L2egEcl3q49VA6+UeH9TrUZnFr8IEp2AGy+J/2szCyY+Y2W1Y6VuP+cHrXyrDEneMfeGxnn
hcCsN9y5khTB3Bu9n/Xrgn3w++NEDoMrH6W+u+wINiBoHf5wCdwh9EVF/zb4dbzV9aoYcEaW
v+ruHHBBxpZT7yvAm/eJ8F1sBb+863xQaAhs1dC36cMC2DnzbMBjJvx/1lfaGOZGAtP3ftpj
JAF+Zl/1TFkVrE6qG5Y1AmdEMfcqnAR3sz6M1zkHxtE/1LS+Ds4LlaoNvg+OMbm6vTodzG5Z
F8fyAsxzUmX1sSaw222Ce8kA+FtW4oLQN7DzFsnQGEbCf961zGAsSARvbPDfVSQOfjOy1vqg
KrhQneUNw37wsN+gR7kVOIqB7eINd7Lb62foPhYADvuyJ2ZXNNntPXNslc4GP82d9pOsBNPQ
nctXagfzJ909s38UHOk5+dpjmez2JLeWZLAT/7PvoXWHJgXAoW6aaSoKYKb+G0UP9MCO3xKD
GI6BH3632+rlCq4TS8pbeRXs0rANfz+CSLY8oHdUTgevOzf5dOwFeAv32Iq0FvCA39SR80Pg
KdGfbwyXwGa5XIeV2Ej/+WKn3nppAfCOK2m08gpg2ipjcd3d4Lb3trH2x8G3tNba3j8D7sm+
HNDhD/blGNmw+SHYJeb2Wu8n4Jn3hddHKsGjD3OuWnSSXb6imWN4HJx494zSJTru//xWdImB
nwucyP7Ot0UUvPnrqcJQVbDC0O6ckyZgNv/Pnur24DNdNSRpLzCrw7U48VBwmVYe57YU8IqV
M377S8Hb60ZmL7aAecy03fKHwVyL5SzLy+D0ZK6a/et4/vPEImtekTD4s8zpDgkVMJMaflfu
fvDT+g8LWnbghG2pTKOXwWcen/R5GArmm6azPZYKDmvzrpEsA8s19z1hbwNbKktvoR0ju76O
7w5aOt7/fH/11wV2HLjuQpS1lASYcC3/znEN8Dytw9XoQ+CbZQv646fAh4JjZ3b7g5fVnlwp
jiK7/ZzzzNvyyL5+tdHd17VgZ594Ies+sNZse8v6BfAX0SdNdqv44PP4So9BEwc45pXMuCoP
+GGDe3rhFvBgmHuoohw4ToYmrUwVbNTVN2egD84dmfEcMQMXDbCr3rQE+5zl2i3rAtb2GI0d
9ASbfz+lF30NvFU2TM88BKx8XzdZJBpM033ZbjkFzNUrHtqRDxZ/YyRRUg5mYJnYkfwW7L+P
vuZhJ/hFYHRr5Cfw7Mpau0dT4Ce7IwIylsFNO3l3Va7mh+U3ziN4YAP4nWWlF/NGcO3qTRuU
JcCXDF6YnlUC5/aW6xVogTe8ODxPux+8z7L8hMlx8NYk0bvZDuCPLe1BnO7g3rhVR65cARvt
oqVdCgYL7Ge74hkFFv7m/YUuFXywKmVH6FPwcneft0QFuK/j6rOmBnBQ37eZSz1gr4T7UrIj
4GMW3een5sCq/UKNhXQb//On6zM7b7CBF/Sfvj1BApuWjXlpbgEbcOCOSsuT9RylDoLqYFt5
u5xNBuB1EckKYkfBa3+y0e2wB7v1KODMzoMfHHju7XkF7K+vppZ0B6yo52T5PhrM8GhklJQO
Xja1+WBVBFZTfaBd+ArsOqEisv4d2fVL2YM8+sBl7S0ewxNg9q0Gs+Y/wD7RRvQDqwXg/V2U
/sSZC0yikaOh3wzuqHv1PU4GXHVQLX63Kji5KpDh5x4wD1ewUPFhMFF1+xpvO7A9v2/5vvNg
yaCjOluugh32vUlhuQu+JlQ/9j0G3N7qhJvKBEsRCmSnS8Auhc80l2vBay6GmHB0gou2m9lL
D4H7e0QCDs2Bj5jJvri1YhMsjwsfsL5ZC+beEnqJgw98ZsyQ86QE2MuW7cMLZbCeEMuHzbvB
rN2B3GEHwR0W3bHstmCNGq0z986B+YwEIjddBQu51RJK74Jz++7/sogDuwcs7GXPBqvE67PX
vABHx6/cHVgPfrk3nOHQe7BojqeezBjYTVKCZ8MSmHhR7gYd02ZYX0/E313EgbNwdurLQuBt
0QWxTArgX1bR+Ru1wEyrSq5pmoBlr+SRzpwE/9gn7ZV2Buxi0Jc/4Ut2e/3u1cohYIeP2aX3
YsEJMbtjvj8BF/9kOmtbBi4/UqzR3wB+lbSFy+YDuPSwxOy3CbCn/+OBOz/BMY124wprBGF7
/rwNfoQbXHH4ukuCOJjh1/N5xx1gtZu/inbuAfM06ZZyHwVzmz5iWeUETtzLmLF0Ebw+KSRp
KRD8msGIflUUWOHd8TfcGWCmbd30qs/BkoIjhU5vwYtmeZOJ78Eah5yyv4yDAwL1GJR/gvGC
17+FrxH6zxJKtjd/8YCFS2RqT0uCc/zkSqd2gucNKp0vGoBbGhUHOSzArLHTgvmu4GBjs52W
vuCk881y3HfBO0NbOQYeg7fId/Tm5YEtJs3iQqrApkc6rC62gRWEB6RPD4HV1jKyuS2AucZ7
VvquFoblS8Yyz0MCOOgEi+VLUbCtenbXrDJYKfN+kOxe8BuJS/6Xj4PP+q6rbz0FvvmQx2Gb
L7gs1com5S44Bv/0zeYE8ELEQEzmU/AevupJ9Wqw2Yx6+adOsHviDp7QL2BlgTi2fcvg7vST
0ZxsW+D9MHi5ZZQPvKLha8EbGfCa9jLjIg3wundLObmm4GTW/O5iW/ARVYaOek/wnji27MlA
cOXQvAv3I3CL9hj3gWzwd2++ykcVZP37eofZVvCFbYo8psPgOvr4wcpF8Hk982pVVhG4P3nl
zTW84DNBAqzmMmDahiHvFZrgdq7zCnlm4OYSVaXT9mCN6uKQHZfAz35Z6HEGg2uHi+x/xJHd
3lv95cl88PjLNOapanBwqvOjX93gEKWtL4mT4GmauEuadKLweWd7svviBnB3qcSncmEwr8Kz
+PXK4JVTtQJn94Gf/tjsOHACPHX8gf/xc2AaIq33yA1wlvjW4z5RYJ2HOEmhbHCQW+T3rkpw
WXNy9cMOcOznnYmOY+BLC8ce6tGIwfvTmTVffj34YOPBRSlh8JaGg+e2KYOfa4vIGhiA8bqT
SmdPgnfj34Unu4PvKDEbjwaCPU7VeyjFgtmrjq2LzAczB9GLMtSCl1zZ3vj0gr2c2paZZ8CX
Owrexq8Sh/3FDnhNfRLYTMPWmU4aHDyGM36tCQ46mkTz4BDYjiH88iUXMG/T6Q7nK+ARobc4
l0jw7cWdepczwS8rHM8/rABfCPqRWtMBPrT0aHLlBJhdi83AcIUEfL7pMbWn4cBeAQdub5AA
J8rV+AWpg08Kyb7gPAjeMuemn+kM3jTpJm9yBVwlyH+d+T5Yzc1FvykLzH3xaFBiFTgn7+uB
gG5wtYN8gdcU2e3Xqef5rZKE/VsDcofDucF7EqULimXBjN8tW8Z1wSI5tM+kzMGpsdoefufA
emGe+M+BYB3LocQDj8Ecn/vFuovAP5lrnzo1gld10BqwD4GDbOdXVCyT+SfbpBqTFOzPvPzD
LX0D2PeHH56HH5x7xKk/XAxcGeXXilcEj7+/t/BYHex29Lyh/D7wI59fnxoPgePbV5ecsQbH
cp3r5DkNDhES1mi+BNbhYaQLugG206LDG90D2/Kvus0TC75hz2w/kw7+iGd80lgINrVZtH5a
Cc56OxAa3wi+w1q780EPmaUKnO4Pg7cHFm2KmwVvrhqwz6GRhu2vJn2tNyzgqJ9sz8dwYP5J
/VbOzeAfcUL3dKXB692r6a7uAPeIH9lUowt2G15Hw2kKfiW7+aHDCXAaQ+3PGmcw+ytVCekL
YIJOnVSsP9ioPZeFEAqul1OvfhADPhFTayeYAX7TFD1fVAQeWol3N30FHvgRNrXUDHb/6Wmf
+gHM+150xGIMvPf7ereN38GFGolrx1bKwPOpaVxdygFOxUdE3+cFM3hO3vcSAzc+76lw2gau
e/+Z10oL/PB8ZL7VfjBL05UAF3OwZfeOZF8n8FP5YxyPPME75K40VvqDk3uNRmZCwRdnXC3F
4sBOX29pOmeBS9YeCCsqAT8wvXZkTS048EpLtGM7uFbkl+W7QXBWwtsczWkw30bGkLJf4IWY
i4yarLKwvbG4TGohgMv7LNvshcGywze3s8iDf3gb7y9QB/vRJ21yNAQP5To9FTkOvlQSwTbj
CN6/nVuhyhOcpTonG3sdHN7Cy+ofBj40F1NzLh6MS/A5dSoHbDjQwOhWBnbLj470qQef3sIg
EtkDNjooUF48Cn7tjLP+vACuT2ThI63a+p81TcS+Hd4Avj2cPJkgAJYeL1y3KA32XBthd0AV
LLzFf6FsL5hduu617FHwW6Oc3icOYKfhGxqKnmB+72Sa2uvg6Q+2BOtw8PnVopEsieDgHcZX
nueBeWn2DbhXgF8TA56rNIMzEy4R2PrAqrSxtKOT4PkBN8+Gn2C+g5cDX7DKwfKkSntnMQm8
8MX8/gtRcPLksYQGJbDrwR6bUV3wLMFjhO0gODykS1rVFmxxO03nwnlwp/FDufJr4KHoEzQc
YeDihKInTgngdP4Te9/lgSX3bezTrAQXTledetkCzjrCs1JvgOzrO9El9kyDv0XsPexJJ/+f
rb8MiglwgN1ZMja284Ofq4dqhEmDb1+8dd98Fxind0dM3hBs8iWKjssCzL+vYNMKV7AKbuj+
ojfYJlbZaTkYbE779jFzLJiJLVdrczb4ghXbMb1yMN6WcdqzCZyvVctU0AfOpPV/8nMKzOF8
7oshnQKsP21rq3jCAU6dHNlFFAC/XVp0uiMLvpd1wGCdBnhW88BUjDH44ZDxQSUrsEJWVvCH
s+C4jIZHwdfAohP8t/eEg5s88TacyeAb5pJSXwrBV3P7p9/UgC/eLSwo7AIvSir7Z38BZ9b9
tHv6A1xaFW77mlUR9meGxgcM8oD7m6q7WaXAaSqZFuq7wHMV9IJXjcB+Hr7SLZbgipSBQPGz
YPm2UdXQa+CeZ+amqyLACWbr266lgPtGu6o5noELft1WSKsDRzltEDXoBb87uj+d9it4aJ9i
5QvabbC/hafows11YEO6pm6LzeB3kT7jmgrg2NHmInldcJhNi+7Ww2DCXHiiihM4e3x7p4kX
+J5g36jHHbDRbNZg6mOwjWR7w3A++LNiYL5sNZj4i+lRQBdYkS7s3tcx8M8D5+JO/AI75y/V
961Vgs8ru4ubnDeB3ziYpK9SAB8+MuqSqQs2upHgfuIIuHBmvkHABbzd+KDXtA94nlcnuD4U
rHpLl6UwCXwmYuRHZjG4kvGZQ+5bMOdZsxNVH8EtOsdHB2fAr6o9WDlWbYf7y2XSqk8AZyon
7r4nDt6weYvnF1WwbXqM/T5j8PNHbYLlNmCaigeFqhfAn1pfb6kPAn+I2HbFLg7M09zWxPEU
HGgUSKqtATcu7nG+/R5sVsbZdGIKfCBneK/GSmX4/YSfdVOyePDKhZYKaXFwJm5Dw45d4KXV
WdwHTMCKya+LvO3AKxgDMwougV0vSK1YvgOWC/9aaZAIrlVgW84pBj+61pYv0ADe/Sv8e9wA
+KVberPUAjiI0233W5YdsH1/ztTZnR+cEvBSU0YeLFki0bOot4Ps91Vwys3HwRdUGE4Vuu0g
+30KP9+MG2AOy82Xs6PBBhr+TpW5YFKK/uHP1eADPnxGXL3gof35R8xmwBs0qq4mMqrA/nFO
jg56HvDKbVbHTsuCFSpuC0zqgBu87WUuHAMnz7be43QDlx4rPlB+A5xZvurahUdg5eF8Yc18
cLd95W6eN2SX5ystMvSBPzIL7/z1DZx65/HmVaw7Yfm/p66QRwDcKFmzQmsb+BiuZu2lfeDn
3XQTFVbgNbr5jwkXwamSQio+IeDmk0nV88lgq5rAvZdfgCP2yXevbwPn0zB7PhsDO9PuIBbR
qsL19y3fIrGCGzcyEK/jwAd3E6uXBMAZOfQPzkuCbY4HRS4pgX+cv17jrwVO03wtRTICuygI
tBYeBU8PupcctQP7rE0ZYzoLZsiKcCjzBtP2Se28HEjW/zrmpBEBFrq//gdHPHjjBe2pkSyw
bOqUUc0zcI7LWqHs1+AQs5QzsS1gh61lKvc/gP03WgQ9+AL+cvOBbcI82F7Qr6VgxS64fRqp
1mY2sNWF0jPfiOAthvvyNwqDKyNWxZhtBW+tZle6pwp+WxBxo1sfbJrQFCpyECyiMG3hawU+
7CU33+8Kbv/ZdUj/MjgkWfBu6U1w2qB54rZw8MO7bfefPwZbr+08p/cE/FKxVPVjCdjY+uPP
yzXgjxeiCwTbwN4Dh13b+8kuD/GWuTMJPr/Gg9b4B/i149NB3tVq8PPAg5n9sxvAZvhu2paN
YL7AZzrFkmA16ZbnqcpgO5FUm3hd8LTKW/0kU7Dih7dnci3Bn4XUBqpPgXU/VcYOXQInhc3l
swWA77m6C+6KABtZfft+IQEsyMOtUJYDLmF8NMhaBr5hIrva6i1Y7Me9tKoucGupf7PksBrZ
/uv6K4/nwHqpsjV8K9T/82oH/8QkdnDI5SgBRR7wzoa92s2i4DBBD9y5beDnUr+iBLTBc17V
vV3GYK7g8r4HJ8DrarszrE+BT/mz6m2/DG7YYPQcHwh2K05ZQ3cfXJeP15xNAi9ppdt8zQfb
ZJ269K0CrCt/4RZDM1hN7FM030fw+ERdocYE2HPMpP/0D7B2UvLGNCYNWH4XTXuP48AZHdb0
SkLgC53KecFyYMXc7PApdfAdv1VPjxiBt4Zc5Ww2B3sZu5cZuoClXaWfdV8C4ypXrTkVCH5t
YVa25gF43wOfzoIU8BztuLlDIXjafuWxLa/BUjPbO6ZawSwrVrVXDoIJRv0WcTPgidG93jfp
NGH/mkTLtktrwaOdi/c8+cBmDGYPfSXBeVKXje+pgO9tZ3mVswfseiZuoesI2CNkaIbVEXy+
0v/F7gvgjNStlqE3wQXHEz4PRZLd3oUwQ60UcC9nbVpWIXht46plgWowTz1pz+N28Kqq7sfi
Q+Ds76TVFd/AHZqlVywZtGD7l/SYh40T/Cggr7dqM7hYo6/OXw6s+5B7yliT7PrBlvvFTcB3
anOW2K3Ahkns07/cwCny11UWr4Bnd4mM/AoFP9zHusieABZL2uMhng92NGF1MakCe0q5DVxv
BQ93Z/W+/gTuqZux5fgGVtjrcdOWQRu2R6547a7lBAuf2Z2nIAS+9lPo7RMFsPy0VbScDliO
/ojUqwPgvZ3WASfswEarRwqYPMEmArtLS2+CR7OXEi8/ANvudPHUSweLHeLbtfE5uD/i9gqG
enBDtHrtXC+Yn68mamoSrPw559p3Gh3Yv/Yu8hYrB/j0KOmZuACZ9fu4Dm0Fl6iHpIdoguc0
+nzaTcEbZ6/GCNuCmbeorL3mAU6aedszeRPsH/OL2fohWOf546ThDPBK7juF51+A63vitdc1
gXltXx591g82v9Sy4tQs2FKmWk2aQRfWzxfCBH5xgXkeK2d1bgEr2pZMlG0HTw7zfsnbA96w
9XxG/nGwyJsOlQpX8HSiRfJ7PzCLstwMfRg4xOuS8LZkcMEXe0PPYrDzla3nq+vIrt/D9Vjg
A3iO62BvwBRYb05NhpZeD34f/K1gsh8n+PaDI7rrtoDzfqnxPtkO3qi5U/bQXrCG9fOgtRZk
t7dWanvbGbB7Jrta8jU9st+X+pDuHwkmrRO46pYO7j9l9875BbhtTiv+XDP4+rjpz4BPYC2P
7WOZC+D50KjzH5h3w8+DiOKp3LxgfH3MLTsZsKHxU9EKTXC3pkyQyEHwGvGPpY8cwfsCQ15v
9AYzD2zMyb0LTs9xvW6UBI70szb+VQyWHZ3kLa4HdwWsmPPtB88IRL8/+A08d71iQIVJH9YP
si+vleEByxX1nZKVAat/XsmirgVmzGQeNz8Etism8NxyBg9NWKS89gVXOHFFsoeDN0e5z9uk
gXXEauvfvgCn4g5sUXsH3p57dm3VMDg84dId02WwynxT7re1e2D/FkePb4Ig2PwNy2rL7eAQ
ix4DSQMwy43ak0xW4GynY/tmPcBThYLE8SDwxvCi5tnHZF+vJM+buQgs5fhcUroebObpPWo1
AK40znmWsgDOZ2DL/MG6F9YPVQ7VHRMAu+w/zt+oCD6gN1VgsBf8tXv0/kdLcLuHRKuXB1jM
ONFV4jaY/bjGhbF4sOQBuuWiYrBWXR9teCM4nzQW6vcZvOaqTLnXD3AGX/2dAI59sH3y9cPa
JGFw1Ydg/WYVsO2btXtYTcCTVx/wHHAA0/Y61+X4gIcev7PgjgDvyuIeDc8EFw3GndpYBS7b
XvXXNj9Yv3I8wWYavKftldwdegNYPxK4nfmNDVziz65+kghe6mD73ikI7lhl2mYmA47e+OTj
+x0GZI/nJLejLnh6cfgunQn4ld85nVhzA7Lly9ldOo5g2eXXV+bPg32K9+Oy/MA7lunoXG6D
14lWais8AP/quzG5Kgm8Z0nv18cc8IMguksvS8G9s1nO6bVg+zemXdFtZPf/28+qyH5wWWKe
QtQE2NbsulzKItn33xZU9ZzB8D/TafSNd3GAnXFReTS84K5Ng7ySouA74T1y1grg7p9PfiSo
g7n1gz0n9oEX2jLydx4B25vvyou0BU9t8L/4ww38JPTFelsf8P0xSb+eW+CIx/z1B++Diwv7
lz4kGpL9e84qDpdc8Hl7fvyqMrLrexluSK4DG47msxp1gvcr5DKu/AzO8sphKZ8Gr3Xk57v2
C0yjeFrXmMUI1jfyibdF8WCXvZHzzILgMGaHG3MyYMHQDbuHd4JHZDaqD+qDxTp53EcOgtds
jP46bw1uMVHIZXMDyzldeyXlAy5dLyZ9OAjM8+Tt96AH4KtHNwjVJYPpvme/4HgK7uc1qz1R
AV639fmekkbwteOxBry94Ijwqs6AL+ALMV8nab+D49gnIn0Y9sPPQ4yDepnWg48/SayM4gev
0l67X0kSTEPICP6oDI5fc+xKsB5Ye4pFTu8AONc9KYXVGpyiyzfSdQZ8YtF5MdsHvJ4zeDDk
Ntid72rmpSiwcaTx8dNpYAYapp+nisDr2vPveLwGFyc58Qe2gv1W6uclD4A77pwybJgiu3//
+yqqMzTBKv/79/Ls/5z/PWIGdswTzJgxY8aMGTNmzJgxY8aMGTNmzJgxY8aMGTNmzJgxY8aM
GTNmzJgxY8aMGTNmzJgxY8aMGTNmzJgxY8aMGTNmzJgxY8bcSsNG878Z7f/OTF9FS3ubRpXm
9z+E/99QdvKB8sw0MJad+vS7G6QHB/8zHJ5N9X9j3ZnJvjz5WPd/b9/2r/PvofYraCjHuhcy
/x7rvuovqbucdXY783uq+7PfV/l9we8ryv11/mzDX2g04/Xf1PFXF5rDLH6Q/ujfvWv64/Um
09AHd+CfGy6S/ujf/aznkxLTKehZzezcVRdIf/Tv/r3z6yyzr9AvH215Lf6N9Ef/7lWrbcbN
JqFnbrBLxc+S/ujffbgD+1mzCejjGFR46adJf/Tv3qdvp5bpOPQ93eabxydJf/Tv/rKpm7nx
GPSuMf3P3o2T/ujffc590QrDL9D78bX0FX8h/dG/+1j+Aae9o2Tff9yusEcjpD/6dz9KizPX
G4E+eki5w3eI9Ef/7pnntCI1h6E/J9KSePIT6Y/+3RPNxnCqQ9Cr8nHQag6Q/ujf/TXBK73b
PkOPW2D4LNBH+qN/940rbo7IfIL+x6FSY9oPpD/6d68v7bFNdBD65w4a+u97SH/07743pO7t
xgHox1/FvynoIv3Rfz9fGT8T8P3Qv/AdbQruIP3Rv3ua5BvlbH1kr2cJvmO2baQ/+nf/UmeI
d+VH6Kfe7XNReUf6o3/3bvPBZd97oded96Vf10z6o/9+PJ/9iht/Dz17yEvS5wbSH/3387Uj
/dXHHujvELgKCt6S/ujf/VLFGtGWbugNvK43+78h/dG/T6vkHzVUdnnBUSeFeJxNa0hIp+Bb
c/M7oeed670u8JqEdM1dofaEDugjVJv4J8mmUlN70oZ127126Bd7FhWKXpKQpln40eHX5kV2
VFOLWp8yEtLz6o5PXVuhd8li7NApJSH9aKSx8dg76Jc/zx1fU0JC2mBTjNDuFugNTotZNBeR
kH5ct7dYvhn6ca+srtACEtJyBwyv8zdB/+OpzxuTfBLSpp3EO8yN0MspJMhsyCUhXfeT/91s
vRfZURuJbC1kU8WpfW1du8H7t9C/Hvt0/HYmCWnjnxkMVXXQbyqh36SXTkI6eRd+Lu0N9EmM
7odWpJKQ1ly6SQyphd5afyfdsyQS0j8G3DzO13iRHRXtIME1gYT0yX06a45UQ1+/VBm7+TEJ
6bui5zp3viZ7veV7hXfEkJBuoXHp2vgKekk6/8Ub0SSkD0/Fsa+sgr45v71mG9lUeGozFPlc
+FwBvQXJmX44koQ0c4Yn4fVL6MUWNVNCw0lI5z/7MZZYDv2rgEMFO++RkN7geG76ahn0DNcS
xUdCSEiXvjcROfkC+lWcm5jvBJOQ9qxmDN1VCr1gX91ehSAS0ssXfkjwPPcim0J/f747gIT0
ow9Ri9+fQX+V6wq91w0S0mynD357Vwx9n6CfG58/CWkm3Qf8WUXQ3y0K0iq7QkJ67dqGC9cL
oXeuj/I45ktCmsBqTm9RAP2DnkSWJS8S0vqcRcWKT6F/1PLo571LJKQzHmhEs+VDb0+8vFfy
AgnpzljHzE+50O8PlV965U5CuuDl/dHiHOiFw5+vOHqOhLSkuKLR7Wzod84x2EydISHdFp8x
eOIJ9GcWVm664kpCOsPHMlYuC3pZy4xtG1xISNdmlfuvyoQ+6tRkYrwjCWmOLMnwjnToT797
bi9jT0J6OGR9Q3Ia9KrPVl8ptSEhPUjbLuGeCv3R2oopHSsS0r5y7/O1UqBfydeT03SChHTr
90DL9clky5/9aq8OmJOQ3paxW6EvEfpYt08ivUdJSDOqXpbKSIB+DWveB4vDJKS1t97Y6x4P
PYn34cDAARLSh6Mag9UeQ+8rGCB30pSEdBFv4TfmOOhNcM7v+veTkA7/Guf1Lgb69ZbSL44b
kpCOap0RefgI+saJstmuvSSkwzexLpyIhn4rN6ObsT4Jaf4Kg8/CUdDn1Uxtr9MlIX1MkG9u
/AHZ61nXdreaNgnpOKMegZz7XmRHRTWJfapBQlqg+5Pr2Ujo33rGKW9RIyGd0fewVyGC7PNl
myLp/k4S0rs5Ley+h5F9fg3+VFm9g4T096d31hbfg95NvvfxOSUS0ia3b7V5hJK9Pi2e6/Yr
kJDWXcgpULwL/WEOP0l9ORLSJe3H877dIXs/ntlgnCdDQnpXzNSb3GDoawQPPSVIkZCuMKha
drkNfYi+vImXOAlpOTcFXdEg6LMvB0n2i5CQVuaPz/gUCD1ti5aGujAJ6Y/frUUeBUDP2q0W
EreZhLS8aHep2U2y9fN4KxyNAAlpdqfD9mw3oNcvvNN6mI+EdCKtmtgrf+irSSnV+dwkpDXy
h1dcvEa2vmF86ysrkYT0wvvkr1JXoX9/hFf/JI6EtNLh7qlBP+g5cnd2FW4gIf3gXgdDhC/0
p/bVRzKvIyHtcnlBQs8H+tXJqTeOsJOQPn87xG7JC3rXJ7nJaawkpINql/PTLkM/3fl6boGJ
hLRxUfC6w5egf7OuykWDkYT0O8dYn9UXoXd6c31D0EoS0vKXrv566gl98djkp1Y6EtLmq0MC
LT2gV+5v+0CkISGd/JQozOZOtr5xD09nvkxE2v3Juaaic2SfFy53d8ctEpFeSPrmb3kWek0z
3mf980Ski1sm9FjcoJ/KD9/HP0dE+vhkNj7vNNn790zbqmPTRKQ1tofMHHKFPvdbXn/EJBHp
TRf72mlOkW2vbWH50DRGRPr14teqBGey7ZdzeUuMo0Sk4/3WPtd1gj761VUFlSEi0oHWd0vG
HKCP8Te74zpIRNro3KuKIHvoPZNoGB/3EZF2ZFxskrQj+zwNM7vf3EtEOn724ucGG7L9A9oy
2jQ9RKTzQqNoXKyhPyhtzSrWSUR6tiKFl9UK+pTd1V9M2ohIL/ZOqKVaQm+zTrzvUgsRaZ2K
IjutE9DPXLcde9xIRHq43vRunznZ8kFqL0v1WyLS+I9rX1w4Dn3a+uydo7VEpCeM5cfWHyPb
fnlpeZWpmoj0AhMbIeMI9AN7FHuEq4hIDx/7rK1xmGx7yoBGXfMlEWlZMYazXQfJ1idHAwuP
vyAiXVhXEONyAPqT5Znb3UuISJ/AadSuMCNbP1wh/yaoiIh07cmZr2EmZI/nyWmrx0+JSH+8
wrp+izHZ+sPGXKanuUSk/as7ZYqNoMfTaRe+ekJEWuVHtL6eIfRKhhedWjOISD+3fWzeuY9s
/9gQ35b+VCLSD5vFnG32Qs+ymW50LImI9LSHt9usPvRLc2OZc/FEpBfwA6e9d0P/tSHj3I9Y
ItIpwxG2zHrQc+axq9I+IiJtHfbJ+J4O9DqSfavpHxKRvruHRYFHm2z5c2i0mT6SiPSOnH1r
EjXJ9id3f4tcEUZEOr9vqlNMg2z99nPjUZq7RKRlTm+NzFGD/p2SGvfSbSLSbqcP7FbYBX2q
8Pr2mUAi0sfsoieLd5Lt3w5iuzl6g4j0AxOtqztUyD4fO35s/XCNiLRixm3mF8rQf2OIb2vy
IyKtdbTIZ+d2su8/sc7ppTcRac8a2qHSbWT7A9t3fH9yiYh05ZtEpR2K0K/VLz0f5UlEej73
04Vieeivaa8f9j9PRPq49lKKvBzZ+l7sSt1TbkSkT5QJVGbLQt9UaRZp6kpEmlR/v0ZUBnr2
7IZ2JWci0j/uPiiKl4I+eVF6BbcDEekTKeZ3SZJk+3vfqBGWbYhIb8NvMr0rDv3GT1XE7pNE
pEMteGlXi0FvHn2ZocCCiPTuMwF3L4mQre8NSXYGHyMiHZYdwzIlDP1u7YchtoeJSCdNhTlY
CpGtD3y8Kq1ygIi0pmdC1rvN0Ps7ZeatNSEibbdiRYfGJrLtF412woAhEem2/E8DuRvJerPy
Ezl7iUjnifg18/NDv3ereIDXbiLSIg7sj4N4ydZXV3aH6OkQkf42WW68yA39w5Cb7us0iUgz
x775fJIEvaPGklLXLiLSAq3mB+oJ0Mvwj3VEqxCRPmqYlyyPJ/t8aSAaWmwnIt2ss9AWxQX9
XIROHL8iEWnpI8f6V3CSbS9bS735sJWI9P5Irlrb9dBvqAqufSBNRLrV0/hWHQfZ/pDPgo9M
JIhIZw5piEquhT4+OkubVZSI9CsZ3ONgNrLlid3Qy5dCRKRvEZZnvrKS7T+xu8l2bhMR6ZBq
fm4DFui5rIzFhPiJaDflcmcyQa/YQINr4yYinSrzcZZpNfQj5XpNvgQi0gUmLbHWq8h+fko7
birORUSaj6VIoHwl9Jd+xSa1riMi3RRc4kGgJ/t8aRN4eYGdiLSRP+Pj03TQJxqKJfCyEpG2
WHoVXUNDtv0y5LavfDURaW9zfkfeX5dhf3b50xfmDESkb91RZXJbhl4/P3V2mY6ItFGjvvvr
Jeiv7Vk9HvGLgDSpyikfvwj9dNXNJOkfBKR1LteW2S9Ab7JmaGP1AgHpDy43Iou/kX3/b1qP
HpkjIE0606iyeg7682ZrzCamCEivelWZZToD/aNKbdbLEwSkVzIHfImbgr6/R9GL+QsBaWsd
7anxSeg/CUSkhQ0RkF4YIpUpTEC/soN4h3eQgHTkUwkz7zHow2Y8JBI/EpCuHIjOfz0KPbeE
ra/IewLSm2lvd7GOQB+4/CAgvZOA9GY6viqjIejDw2t1xNoISF/Yftot7BP0E9PJhSnNBKSf
n0gY6hiAPqL6S+emBgLSY7c6uAn90O+6p50W9YaANJ2sCOehj9Dfu39KdH01AekrQoUNEb3Q
R34kHLxeSUCaOeaRblsP9OsVFmUWywhIt0ku+3J0Qz8u/7LA7jkBaePqMc89ndA/VBf83FZE
QDqn8IaUfzv09wM7S3Y9JSBtnTER96IV+qY1t5VScwhIl2fJNX1rgZ4/7PtR9iwC0mc2uxeL
NUPfXPpms1saAWliY/1Ri0boN84XBLcmEZBurjMsDq2Hvtr7VNTWeALS+XdxDa/qoL95Oknv
TgwBadf5bQ/ma6F/W7Qm/MtDAtJ1m+q4hWqgFycd8FGPJCAtGTdoYvIaeuI2OcbIewSkpfZF
qPlWQX8jwExk7A4BaeLJpf6MCrL763Dqs3IQAemLrvzyHeXQr2KX3h5wk4B08nHu7bRl0J8N
1hNqv0ZAepcz7ZRwKfQBn51S+PwISFfKfjDdV0L2/XzdXWbjRUCaNeq1g1sx9K6n7tmnXyAg
/WGsViKiEPoxjqXEifMEpB2fLUQXP4WezlHcTcKNgHTn3PFn3XnQ+y71NDqcIiBd836N31IO
9Eckip8lOhKQ7mFcN4fPhv6q+VnZD7YEpHeYe7ArZEE/2Fsqv8GKgDQf3953Rhlky9sRrUod
CwLS+u0PdzqlQb8j73mXx1EC0g+sTxleSyFbf3BocU8+SED6Ck0LY3QS9B9kFKPemRCQjhSu
P5mXQPb+Enmo9dOQgDR7rv3JmsfQqzllndm8l4C0auUThvex0EdxbRTQ0yMgLdKYpDf5CPpc
zfj9jloEpBciTSVpoqHfz/V5RaAaAWlf2Ypctodk6zPZ4TIpKgSk3/yYbuO+T7Z+pWbXW6FE
QPra49FwkQjoN9MwM/TIE5C+vzFzTi4MekUJ8eQpGQLSD8pVJ3eGQu/N411BL0lA2nB9gp9O
CPSJsnmmG0QJSMsv9mTvC4beZ8DDXECIgPRs1Ii7SRD0HkU+HyQECEgPu71pPxgI/eyBi80K
vASko1R83x65CX2xF0FRhUhAei8968Fj18keH0ZWNjUuAtIncPYex65BbzxOb6q+joD02IZw
8aNXoJfVy2VWYyMgLfgx2O2QL9n64fEaCRVmAtJfY/fvMfWGnvnewnOFVQSk97ztempwGfrG
C9MFEisISPcS+J/oXiRbHjbZEgV+4ZE+foNPcZcn9O2+rEPrl/BICxxt3qPgTrY8zAzB08/j
kb7NK/lV9Bz0NYmp2V+n8UiHRUvw8LpBX/9tVWbXBB5pmbHXbeynoe89cZT15Sge7aNTONpT
0IeOHa1P/IxHmiMwZmDSCfqLrSWT1/vxSJcY1sq9dyD7vI4TP2Xbi0f66ZsTa2rsoPcjndyr
2YVHWvPAUcdcG7Ll+YH1N3jb8EjfDEvf+9AK+pHcaZ75JjzSNNZqeX6W0K/uzV1T9xaP9NQn
tod2FtDnt7GYRdfgkZ5wW7ly73Ho3WdyvjlV4ZHWeMIxIXUU+sP9h8eUyvFInz0jaMpxGPpN
Gc/lVz7HI53RLqE8fYBse7kk6F1dIR5pRVn8o0ZTsv0D3Vcr7+ThkV7ANV/KMCbrtx9aafwE
j3SbmWb7dSOy7d9zFffWpeORVlk8mnrCAPqTIR5ujUl4pG9pMnxT2kv2elgpF3PzMR7pq0VC
RWv1odd9lsml9giP9Cu2tOnPumTv33Uxnd/u45Hett09tlgbev8vLweTw/BI37Q5VxmoCf3U
wyaFAyF4pO8tBBw6qg59Or1/E30QHunuG9EnxHZBv/Nq9JPMG3ikp8dDur+rQP+R6V2LyVU8
0vPieyqrlKHfwza4bdEbj/Ty9SfEYCXoc6IC+h5cxCPtdebRxwOKZOtjptdrlNzxSNeHLa3l
kyd7PM9EfG07g0f6SmNC5idZ6BfrPE1OueCRnpC+nJ8iTbb+0DXxjcEBj7T3L8vNjpLQvyvO
a7lvjUda+Yncspg49DLyt0ZFT+CRFh1v3D4mQvb6fLlNqfgoHunSYI6PKcLQS9y4WK55EI+0
emLbsJUg2fLcFudVb4xH2tBuwZhvE/TsS42uxgZ4pKU1jQQ6+cnWN+7bR7TvxiO9+91zo2Be
6DtPpk6ZaeORDv2wrl+TG3phVdOLrWp4pGePijV+J5BtD37cqmCggkf66ZleYjoO+qIaVv7q
bXikJ1xpKo9wQi91MEVxhxwe6U+tNq+Z15Mtz6saL2ZJ4ZH21RnZVLQWeifBvWM8Ynikc6IO
dJ9kg57LfexqgBAe6caW6yNrWMn2T8ae15nbiEeabZ3+3gImstfD+RKZIzx4pPvTzzAeY4Q+
puK6ehkej/RxYiPnCgboy3TTPPg34JFOo5F2T1wBfVftTIsXOx7pI4JWojq0ZI8Pi6xhNzMe
6cGLSpLDPy/B53G30lfZVXikvxIu+177Ab0aZ2/WDTo80k3KPwQEFqG3Tf8a1LOMQ3pQ3Zuj
dB76jcnagWLfcUiP5b/TN5uD/qxMUaLHLA5pP+WKpolp6HGRpPcVkzikT8Wvj7nyFfpNxnoS
zF9wSHO+D36Km4D+KzMpct9nHNJ18z/XpX2BvifakXSnD4e0ttDmgu0j0O8TwhU09OCQXiit
jq79DH0O7Q8b5g4c0k4ymTVmg9D7N38T12jBIZ2zPlJ+oA/64MDhVZ71OKRzufQHHT9Az3w+
Zza9Bod0BPONptke6H/JKsz0VOKQFullX/bsgv7Bq0N0TGU4pMXuP7b61Q79k0g6/q3PcEiX
WtMw+bZCr3htw97DT3FIF1z7NEzXAv1U54XrXtk4pBkPsNP6NkK/9hhvQ0w6Dul1+3cY/npL
3n/he5GEQ5qVSbjD8w30FzLrLnbF4ZA2Ew4Ina2Gfjw3u286Cod0pyvfVcdX0LPZ39zLGIlD
+sTLmOT+CugL7HVeEkJxSC+zVC6blkPPM9e7XeQ2DmmDw7v8akqhJ82pFcvfxCE9W1evoFQC
fSiTg7LqVRzS9WH8hJQi6PuD95VreeOQtqebEOYsIFseJo5q613AIW3gNHTCJw96ma2KDXrn
cEgf1yl7NZoNfeGcnKmOKw5pQS6R/UZZ0Cd4D3SrOeKQlndpZShMh75eX+24kg0Oaa9514/E
VOilSkw+SJzAIf3IJrP3UhL0awQEDvMdxSG901+Rpjce+i2N2U1rDuCQPin+XF05DnqWDcxq
i0Y4pKt2DidFPIL+4l6ltP49OKR5ck6KzjyEfnpMn6VaB4f01as9dbvvQ69yyehkqjoO6Rev
F27GhZP12UdybqjgkA4OsTg5Hwr9U1uvOattOKRZmUvN9EKgly9uFNu5FYf0jHme5YPb0Av/
sDXbIIlDevrtyLXRQOiX3p5wG96CQ5r2Hl2Zwk3ozfm7fQs34ZDOpo1j9fOHPll9yucqLw7p
8zb2znVXoNcdenVqHwGHNA3/8sd1vtA7iXvv5dyAQ9r79qLl/8fencDF1OiPH59WbbNvlVAo
Fa2KUmkiESX7roWS0r6pJNFCyFpJ9hSRtWyRNUq2tNuLUJYISQn9p4Xv0eP73Mdz7/39/vf+
zvGq03vm0yxnmTlznJmmL4H+2OHhivfo8qi/KYg2bg0jPN/FDilJlpFHvXtAZsKjEOi9nNcF
T5OQRy1llG7RcyH0Pov9pdkUedSRRyKaZgZA/zaLE33tMx91ALXi1CZf6A+v3/sy/CMf9dqr
I5YWeRG2TybPMdat56PuP3veOGkP6Mu/LfZ59JKPesXIor7mbtAvmzJobewzPuqe4j2/+s0j
rO8tBfF6VXzUz2nVFalzoa/0CI0ov8dHvevJ8axSR8L6xV09PriMj1qtSXOtqD30xydbdePf
5qNOvVI2X3sm9AnrP+zMvMZH/fbsKPNp06DfZPy4x5grfNT5dlxmxGToMyocFled56Pu/uz6
g7QJ0Gcp5p7zO81HPeVJy84CO8L2yROnxyLH+ai3pg2c9coG+gtjEp/GHeaj7h4jfIgbDb1f
ztl83n4+asuSN0fVR0LPqDdauTWVj5pfGmttaQl9P3GP/r128FHPy5tZPNuCsD0cfW7/9s18
1NxHdaMDh0K/I3RLt+7xfNQOllePrjSBfmqgr0X8Gj5qRb6fxHYj6GdtyZwqE8tH3eDqa3nY
EPqS46XWiyP5qE+bjPE8pw+9/dDx/LrFfNTxsZHh13UI2zMeaeenB/NRjxyyLah8APShlhOG
5frxUQf2U5xUqQF9r90Ptmt48lE/UFzEfa4GvcLYm6WrXfmoN940z37ZB3pNkaPVr534qMfU
PDerU4a+NEz+uvUsPuoVX+t21fUgbG8nJK3cPYWP+umGoupXitCzBNdUPo/jox7Bo4rW8gnP
v2mj19mO4aPu+USx5TEH+qYBZ8q2jeCj3msScPMuE/rsNRffvTTno1aYsH5hIQ36T4+7PTUY
wkf9wuzFp0uy0JsdND2wyICPWriFYnNMCvoRjazR57X5qBsjlAN3SxBeX0wYdrZVnY/aal2t
1zpR6Jd+jJEw68NHva46x2hRaxg8P1/Z1XthDz7qXC69aO4X6Ktdp3EO8fmoHfbYDRrdDP31
SYH3HjP5qPc01c3TboTeqCY/gCXHR10yYZoT4wP057z6VptL8lGPe9jSp/4t9NIWzqruFD7q
ml2WB2++hj7zuovJ+s881F9WbmtJfwG9xR451eMNPNQ5c3yoy55D75lq8aTsDQ/1hGLVx9Oq
od9a+97vQy0PNc9JNVCrCvq4RnYFtZqHOs+/LL/1AfRj87bRVB/yUB8es6Wi8C70LhVrFIwr
eKirJe6nbCuHXs/y1SfrIh5q1+RP/dxKoN9QlrNv6nUeauf7E1z0b0N/k8fWmXOFh3pCgO30
5hvQp7mJrXA7z0Ot/cJK9FwB9BcV9x3zzOah3p6wzT4iD/qRQarHvbJ4qPsdOOQhyIX+woMd
sR4HeagnGJZrfjsPvcmdEfque3moq2jem7JzoD+gN+aw/S4e6jcvi4/4ZUO/W/IlZeIWHuo+
djMCNU5A369yTj/LBB7qhxtnVT/MhL4590tv/bU81FOqdZrXHoZefLJEg1IsD/VNI43j5geg
V666nSweyUOd5bCVW5cOvULwaf6LMB7qovf35ZPSoG+V7bvgWhAPtftM47OCFML8DQ9KSPfh
oV5wW1ykZjv07PMqmyLdeahjJ8c9WbkF+l2VW31mOvNQu59WdhyQBH2UzrzeuvY81Nc3ywZf
j4defqLYQZFpPNQ7p21Wn78eeupsEebt8TzUpqocH9E10BfXNIzdMoaH2qfxsk3ySugn6YS6
zh3BQ5218kOOznLoRfobzlQ356E2c31+7lIkYXlOL9J8acRDrVXxZNzECOgPOYtVpOvzUKe/
HOD7OIwwfzVjHJ0H8FB/CuzW0zME+rzaHleV1Hio9zOOTW4MJDxflyTSinvxUB/n+PMW+0E/
cPEVgygFHurV2yLtRbyh3/QuxmQgm4facnmfAZELCMt/2YU+lXI81Ac81y4WnQ+9TaTTm+WS
PNQeGaLjlzhDb6jmuFWLwkOtMv1karMj4fHwXo727WYu6kCb50G+s6Efdj1ij/cHLuoh/Pwr
z6dDv9junLhcHRd1tOS6tdOnQH9LZv3otOdc1FdVgooLJkD/QLZniEkVF/UzjYOrB9tBP+r1
xvW37nJR2yS55qSMgf5svWaCfQkXNW17uYPsKOiXaKhGv7rBRf2xt2agryX0dhtvzQnM46JO
vrzuc6mAsPwYLdb5cp6LOrhs8BtDM+jrJ65/vSSbi1p01pix8cbQn/YJ3ELJ4qLOZjbK1xsS
nn/XbjcLP8BFbTTHycZaH/paq9WlTWlc1OPcjz/foQ193YurTj47uKhZn+Sff9AkPN/te/H8
aRIX9dOoo6NG9CMsD6fWuEzZwEW9buhuufg+hOnpN7fy8iou6ilfGLpVvaDfvrTnFN1oLuqm
YUpHNZSgj2ldej0pnIu6YNizOB95wvK5c6bFt4Vc1OfKV14+xoG+5E5UtqMvF/XCSM2JjYww
wvvzqg0vuHNRjw2qGmxIJdzfKNdTSs5c1HOj8n18paFf2kofHjSbizpVo7llvwT0E32eV9yY
wkX9YHlcxWMR6AtmiYQoj+OiPuWyRYT3bRH8/8eXSHVfay7qEi2j4JGfoS9x2l57bhgX9bm6
hcZBjdDT98adkTblopaa7Dk85T30LP7W3eMNuagtj2nEX38D/bgZKrsTtbmoqV9yNN6/hD43
ZVnOnX5c1KwcQ1FeDfTX7+i/56twUXN67+o+uBr64KuZVpMUuajrm2QCJldCH223PieOzUW9
fe9iad/7iwif37lgymU5LmoDXdF7sRXQD5ep4zVJcFG/0d5ZvaME+vLGQopGKwd1v7uz+2YV
Qu9u85A3tYmD2uGLeXLudehvStTNXPaOg9rkq/Xwonzole2eF+9/yUEdUb64x4Nc6B+cPB9+
u5qDWl7+gdrT89Avd1ky58MDDmqZ53NnvzgD/Ssrk6Xscg7q4E+s869OEpbnUQqPdAs5qHcl
PLN+lQX95Ri7sDFXOahLk8tbag4TloflfR3mXuSg9vetLH6cAf0hj7exwac5qGtYTTfu7IXe
U1OesiqLg3q7JL/2xm7C8n+BfyX5AAd1q6Ne73M7oM/wCX6wN42D2u3SkJADW6Av/VRmnbmd
g7rPFNVXmzZB/zrvIeP0Jg7qqUWPAyM2Qs8xszM8v46DWtLPgT9vLfS6T1pOX4zloP72ddXN
Uaugt0nJT7u4jIP65sWJierLoT8mva/53CIO6uy1u7wlIqG/tHTf6ewADur95+ynVoYT5tea
Z6+PenJQq2n4jzkRCn2hXFz83nkc1KYDrlivDCIsn77Vx5MdOKiD9xiPm+kHvUBGMHnVNA7q
W41ZszS8oN9pQlsYMp6Del0L17PBDXrtKzd7uYzmoJ7CFCzJcYF+rCh70tjhHNT1TOb6pU6E
6am1XN7QlIN6wsR5O4fPhl42tdZXwZCDOnxP9wzR6dC/0JR1adHioPaaInv43CTo6wbeabyn
xkEt2Y+2f+E46PX62g041YuD+uPEls1aNoTpuSVaZqM8B3XK2vSwqpHQnz+9N3EBk4OaJ/rS
bt1w6F96N5VZyHBQPxq5gWlmDv2+nKKbXDEO6iYfv/PPh0DvK5oWWdPCRn2sdfrUuEHQn7Ft
+XKigY16aw2vQlcfevuUQPOoOjbqyasDTYu0oH846uY4u+ds1DEXBNFeGtDHK5UOkq9koz5z
2eKwlCr0T+8FfHpUwUYtsBKc2qEM/RD/rKTdt9moafai2wcqQc+YdlB5XgEb9Y61NvaX+dC/
ub05Qe0SG3VZ/uPG8Wzoh9le+/r0NBv1nZmRLg9o0M88td9hVxYb9ftu7/bOlSE8nt+6eHHm
ATZqjze1Z2okoLf2ParJSWOj9kjst2O+CPTbRAbvuraNjfr2S1e7519C4fXDsf4DliSyUUsa
z73m2AR9y+uoYr21bNQvdYqodz5AfzNx0pYny9motz9y5du8hX5YREHc+gg2avus+09zXkJ/
6oTO0aEhbNSOoZX+ms+hX1ldL/fSl41656y+l+IfQ6+2Pz59ozsb9flH02+2PIB+cLeLq0zm
slEXVWptdLgD/cMjQ849nslGPSjJhnmhBHrR4GcjYyaxUbsnOY7oUQh9/v7Luhq2bNT381la
C69B/2VhS8S1EWzUJ+bK5N28An3JxJsC96Fs1DlfX1NVLkK/Zvb2YKnBbNTURfaivjnQz8/7
oJumw0b9lSWy6+xJ6OONA/wE6mzUj2wDaySzoHcZUTvinjIb9S2xGSU2h0IJ7w8YcsxPgY06
aqbNnLX7oH8yNiRPmsVG3augZv2tVOhnbimN3iHDRl0rl+cgsxP6yKadLQPF2KhV9ZcUDNsC
vfKJKXr5LSzUj+L23ViYCL1n4gbd6Q0s1Gln787btx76UHOe2MvXLNT2e1ISKlYT1t+DVzND
nrFQd/NPmCK6Avrze09YSz1ioQ7eaLFXIxJ6q4Hs4oRyFmr9MwbRtuGE6S9rNKV3IQs1v+pz
nWcIYf3i76k6mM9CbWjcs3JlAPRZzyTCjC6wUMeY9ZuS6g19sor6wEunWKiPpGwdd9od+rEs
SeaYoyzUeTPFbtxwgd6Ve0m5ZB8LtVTSoyv3HaF/oJTuOSOFhTrl4mGTmpnQl2ppiz1OZqGe
H9ao+XYK9LeqGl7P28hCvTFVd/2H8dA3PMkf9HoVC3W47b0FDTbQXwre/tonioXa8YLnqXcj
oa8V2ar4MYyFWmrR3IBXwwiPJ+GMm8GBLNTrtfW3PTYj3H5OCOOLJwu1yrmxuqVG0OcmR7wL
n8dCvdVsQP/cgdBbK1EWiziwUM+oN1l1SBv6Fa6t5yOnslA/Hllnm6gBfcym4/kS41io458c
9AntC/3rnbG7lo9ioXZf+aJ+Ri/olapYM6UtWKh3DJS5MVgR+knpCqKrjFmo3VoWtjK40OuX
bkqV02ehTlXbvOI5HXr3qdnj1miyUMcoFzufkoH+StEDDqMPC7VNSPT6GAnoLfvEt67vzkJd
+5QtN54C/W6RtF4cDgv1EeMHRfyWENj+XSMdlSjHQi3tbVJ17yP0Hu/qTRQkWKjpW1cbJNdD
f/D1+SlbvzJRWzWq35j8CvqyRcynvRqZqAW3XVOpz6GX9Tz5YfcbJmpB0vbTF6ug7/XtdIJ6
DRP1m3JlOb/70I8LnX3vYCUTdb2WILFnOfR2d+WLB95hok6aYjMl/zb0Zg47407fZqJerrzR
2uM6YX6ZPe4xrICJeqyPoyc1D3rtwWLx1y4yUXOGfju3/wL0agI3kUmnmagN44+ZWJ6B/o5q
5sJHmUzU1XEFVXePQx+nny3lnsFEfXXy0owFRwjLW2liXuNuJuqUvj03N++H/r3lrtyorUzU
1eZVeyLToF+k58niJDBRj2mSKJbeCf0p51tXUuOYqGPSr/NXJ0Mv6XunflAME3VB1oJAmQTo
z8+s3HMtnInaOapnbdRa6KdYB7c4LGSinrNS0bMlFnp7zVDRT95M1MpD4kW8oqC3cPtWuHY+
E/VjmXMpD8JDCH8vzilY04mJ2iXwqu3IEOiL+1gy8qczUYe/f9x60B/66V9WHnCZwES9u3nQ
CYYX9FxbxzmSY5ioY1rfeXrPh15f02DkvuFM1DsCTPpemwP9yOFnfcaaMlHn5RqXK8+Gfn9C
c8NHAybq7HHMpf5ToTfqpfNyhxYTdWbQU5VL46FPX/96no0aE3V9TMlxORvo+ZIyy1p6MlGP
zv9iPNEK+m5H9004yGeiPuQTlpEggD4h0ajeicFEfe6gR7fSIdBXDfX2U5Bmorba/Gw0zRD6
FPcJ70pEmKhLTL/6WeoQnl/yB0av/8xAbbA2PyxIA3rnPZlWEz4wUIv6T3FJ7QN9wYGcsbzX
DNR9j2dr3OoB/fbVa048fMpAPVG2Ne8DnzB9ThzblP6QgTqFpT+Ey4KezpCTW1jOQN1sOytC
Xw76yt2XtKwLGai39Y1OHCMJ/cpp0/i9rjJQ7686GuJIIdzfXTuqmy4wUCccfKbm+zkYppf+
uYPl2QzUBxJVtixugN7skNb6U5kM1GuinUui30BfNDNn3/YMBuoMQcaN2Fro/Yrj2LGpDNQ5
bvWRsU+gn7C/+6uF2xioTcMGNEY9gJ6hOch2QSIDdc31ycph5dAHfhCfOnctA7V6pGOr123o
rxzqpua4goE6ftfQdbOvQV9xjHfHaSkD9ZzPlYUjL0Mfe3LuqvmhDNR39fXPaJ2Dft/8UPsA
fwbqggvKdvRT0LN6WfhEezBQN9fGr3h9FHrx0ObKLS4M1ElnA52vZEA/TtTi5kl7BuojSumV
m9Og31HAnHZ3KgP1+kO0T247CMtnt9ydlPEM1IKnsXsNN0OflSl9VWs0A/XTj5/qvmyAPrV7
8xvH4QzUuo8N8s+vhr77ErHByaYM1LdcFXXDY6CXSf588a4hA3UeNULVKAL6vUaMzF46DNQv
+SapdSHQF1wq1/BQZ6AesrLnvu3+0D+7bGFzXoWB2lqtRXeMJ/QbAveYKnRnoE5x22H0YR70
iwbn9AzlMFBrr67ISXSEfh5/G+8JlYF6XbjnacMZ0M+yyhs1vhsDdVytuvbtidAnrZW/l09h
oJ7fr5Azz5awvMUfrVzVQEc9zKbPgk9W0B9Y5eiwqYaOeqHic60oAfTHXGcsybxHR90kUT5d
bgj0leyY1Q9v0lEf0Dv8au1A6NMvJpTKX6SjzvPsX0XTgn57crc9zsfoqJUGiRrEqkE/wlVt
3MW9dNSRR2qrv/WC/qCmTB+dLXTUahnrX3srQG+cmeR9aA0ddXnywdEPWNCXyscsN19GRz1n
88vPw+Wgv2Y2+eyTQDrql3MbmvdIQD9Qe5pzkhsdtf70AEuJ1oXweQYRR247zqajfr2QWWHf
BH3IbruppuPpqOmzfQ5lvYN+UvdPtv1H0FH3HjYwX+wV9LRjRrLaxnTUq1qae9g9hd5gV9Bz
Sy066tJGp8MJD6F/vKZ6sI8KHTXFWDK4ohz6MSnLph3h0lFfm7UkgHMb+p5u/VIkZOioG9fN
3WVTAP2jk4HePt9oqDckWrUsuQR9TeDj7u/e01APPZ8dfugM9KunHhJbUUND7fVqjPqdY9B/
jnobafiAhvrYitiGLwehr9Ja/rn5Ng31p7VfKpX2Qm8jrZZXdoWG+nzkgJrBO6FnXxxtVXCa
htrLa7vk2M3QTx1lcb/0MA11WFXpEIcN0B9Q6v28KZWG+twbu4gFq6BP9i26aphMQ52oG1fi
FwX9sBHfnq1aS0M96Fw3vcDF0B+xV9nVEkVDXaptlOAXBH2YQZh9dCgN9fPE+C/u3tAnnuFH
avjQUE+KWTfXfj70hT1yA2pdaKizJLfl2ThBHz2VlXxpJg114Gb13oNmQN98ZtaMU+NpqHsM
2uGnOBH6Ow5nelwbSUPtN8r9WPMY6De5dbNtMqOhPnCovrrEEvpvVnfGDzegod5zMvFruhn0
i/OXJmZo0lDLqpz7GjwIerPurBg9FRrqZdy8quE6hNt/3G51GZ+G+p6m7G5pdeh3sE9yk2g0
1HqONYJryoTlxyV4bYgEDbWKUuaJaAXC44lioGvoFyrqp5oXKKYs6GXGmclt+UBFvShnbvc6
GeiDVah1919SURfPeS26WQz6Z/cKQsyeUFE/oe/NMv8SBM//cq29L9ylor7DfqTxuAH6HuH3
pzsXUVHrdL/rtLgO+mmS2us0C6ioSwc8nMp5Dv3YoXOM2BepqIenD5Da8wh6XfnFV3pmU1Hv
GN7NW78C+ueTFDJsj1JRl0vvX5FdCL3P3bmzd+6jos6cZjfe5Cr03Z7yx/NTqKg/P9K+cvIC
9DOUF7VkJlNRRyQvrtLOht55y/Ri/41U1Gblnjt2HoVeO1HEZsZqKmpXdZ1vcvuhzxtsXjkv
mop6sc+bJv8Uwu3vOU0sOZyK2rGsbFVZMvSqhZK6DQupqB1y5bL0NkJ/SyLoYaAvFfXIlyfd
V6yCfvETk6heC6io/UyaD92NhH79p0nb3jlTURfWvF2kGgb93EG9Ul7bU1E3zD51c0EA9CVn
Jskxp1NR9x7lsfOAB/SWYZJTHCZSUY9d2bu+xhn6R4WJzRW2VNSqgrenesyG/tujLN9Fo6io
Fc9UN9pOhr75zXjj0cOpqB0jaHuCbaHXWpd23XIoFfV7q0WXto2Avv/BHqkLjKmoN3zUH3XW
DPr8RbOHnjegor6202RghSH0EeMG8QS6VNQ1K5KWvNKCPnDSrr1v+lNRt/BHa3xWhV69V7bX
9X5U1MWVtgPEekL/cmBWTXkfKuoPm7ZHSfKgH+x8dw1TmYp6XXcDbXEa4fGWH1m1SImKOtpV
TPWLBPTGwbTpPAUq6lFJ35zffA2E58uVdwMec6moq2o4L+99hL5Y1eTIQxYV9Z0M/SMX66Dn
PVq0XJZBRa050SIr5Rn0GwpvBS2gUlEPce73dvFD6OkzRn/+KkNFfSr0qsPEMujrr1TF5UpR
USeNkBDpcxP6ZeXTDuZIUlE3RJ259foy9GXWVqUvxKmoP3fLu3wkB3qlUaLhY8SoqEv3f3nk
dYww/S/tVn0iQkW9bpKBQr8D0D++8mnEIQoVtfjHod53d0OvfSZk+pFWOdRHlr16EL0F+jMp
Y+7VfpNDvbtJwn7ARuivmWsPm0Y4v6tnTZr65sZK6H3MQmRav8qhTt+YFuu6DHr1MewDDwnn
d/WSzKz+X0Kg9x/ovqOBcH5XV6db3F7pC/3dOvdZloTb29Wn5sv5s92gzz4YMuMm4fyu3nH/
Jj3REfqlb90Y6wjTu6tvVZmmsKZB7xgT1LCaMD+7uno0VS3WDnrDDOOwy4TlpavPPnyb2GwF
vTw9TWkwYXnsahubDfVzh0Lf40Oz9TPC8t7VzoN2aF81hN7S6uLCQsL61NWr1c7ZqGlBH3Yj
V76RsL52deHajVbhfaF3txPdMV2Wijrq+gX+7e7QK98/uOgz4fGmq5X4T890Z0OfIKXc6y7h
8ayr36xP0naSgX7O1JKmj2wq6m9DghbsFIH+UtLMheP5VNQX3jC97jYFwPEanvZSrxWpqCud
v+rJ1UMfWLFKPq8nFbW4dn6mUQ30058msqt6U1HHH/38yv4R9Ac9tJ2HEJ4/u9qrv/Ld8DLo
97396F9GeH7u6sx9OYFJN6C3jQ3POEp4/u/qAJkJZzNyoZeQFPgVGlJRRzTbZ5w6Df1E7gZt
HRMqark39ibnj0J/b2vaiCIBFXWQ+rX559Ohf6wV3v2EFRX158Ncnewd0Advf8V6ZENF7Twu
K/ZAIvSSVyPjrAnbm12dt4kZsDkO+kaPra+aCNuzXa1euuXFkijo11uNWP3akYp6EPP0M4dF
0L8wHXaz73wq6kZu0VxjP+grCiRkU7ypqD3mDHOQc4O+dUXGTmfC64+uHq3lUXLHAXq3j+9r
PJZQUSc77TuzfQr0p6cMMT6znIraYet4tr0t9FsfhbyyW0dFPUIzu4xnCb1U09BJ/TdTUQ+V
0Pt2dQj0vIK9+baE16Nd3a9YckWAHvQPa+2TTmZQUS94FOWloA79K+tNfLfjVNTbe7w/cLIn
9P4zpBLmnKeiVum3YagdF3r1mS/tUwn7D7r6xMC0nlWy0C+ue39Ru5SKWrn7/FHuotDfvzte
WrSSijpnPOdkXZM/bB/QDoX3JOyP6WrO80oX97fQm97p7R71kYo6MPyrXdUz6G0nmbEMRGio
l0/Z6mP3AHp6j89ntKg01BWmtZdOFkM/XC93v68CDbX3OulhCgXQc3leyhQ1Gmr7oaov/M9D
f9pgyOy7+jTUVudnZeUfh57iX7jjqzkN9WvOrS2cA9BndDNT9bSloT6Vv3HbjBTolVY2qasT
9r929bCnl7M2J0HvZ/bwqbYbDfWdpoC7RWugf5LYf/3ShTTUFPYxabFo6GmtK5yVY2iom3LW
mGstgt75VutG2QQaaqMWatA4X+gPRrZOGE7Yf9/VDdaD0z1coc81mF2Sn0VDHZ8sV7R0NvQP
jSaN2JxLQ609fvWrtROhz5FdXnGyhIZadNjR94nW0Du5UU72eUpD/f6Q3+NEc+jn7TKgP2yg
oW60vJy51hD6BSpiMk8l6KhXKyQ6Le0PvbvhqUIjPh21bnpp7QIV6KW/0ZbfV6ejvlY4f7gd
H/ro5IGTbwyho1a8PdijPxX6WzFXZsva0lGrjexjTxGDPrP/vUtJDnTUqdrSnFtNfvB6aWdr
aoAfHbXz3dwV8W+gz91m3mdHNB31iXqNkxOfQv9pa++Z8pvpqG/40pJl70E/WCNrftUBOuq+
GYIBOYXQe8xYOu/TBTrqp8ar57tcgT4vbI/n7DI6auNu2dbdzkDv/uRqAuslHfWpEauu7ToC
fdHDXh95rXTUJ7xynxnsgf7pO5NxhjIM1HvMeyZe2AL9RbctZZJcBmqPk5PKLddDn6Z3hE1R
ZqA+xVbadTEG+qnTbrJU+jNQT7ml+m5QGPTmIZvvuQ1ioI5K7nUx1deP8PeAmX73LBiozeZk
ycq6Qu85K7bSz5aBes3w5dfdZkE/g52pPnA6A/WDOLOWC+OhD7iWNKE74fiprtY/H5DAGEno
dTKc+vsyUK9Zmh8/zZSwvtzInj53MQO1UvqND0l60Pe4/NksN5aBevkA/sEiNeh94uWYYwjH
03W1wYBBZ0SVoL/0oqn0cwoDdcyzY/IDmNAXBg1eVXyYgbrHccULtpLQj6w4YlCcw0DNZL/K
cG3xhf3XoReLmgsYqHXXpZWH1ENvv9F0jnUFA7V+yZUhUc+gl+R4P7tAOP60qz8n5BbH3IN+
Z3rvGY7vGahzUliblxZCPzln57l+FCZq86LeKwIuQ3854jiDT2OiFhd3THbIhv5ec6atthIT
dZiq/rVhh6CPkAvw89RkotZS/crsuduXcLyCw6JSIybq5w3KHu82EeaX0wLnuSOZqKujm4tz
VkNfHdRLQ3EKE/WHK48EEUuh1/6UdLnJmYl619a+R0yCoNexidNr9WeinlSr2P2tuy/h889s
3bUimah9F1HDkh2gf/Qqf37kBibqUAXLm2aToJ/CWqMmksJE3T9AVPruKOhHPS/duucoE/UG
9RUaHmbQfzUJuexPeL9LV+vkMDQ+6UFvtUo0cX4REzUluUQkVA16749p0tGPmah30cUPNShC
f9TKml/wjol64uiifq506M0+RJ8wEGWhVpg0z61IDHo5+oFH+SwW6tOMlx4Dm3zg+C/BlZhl
hPefdXX5kCUD4l5DPzVjU5qzAQv1G0fLlEdVPoTXi+l6XiNYqF2fTLzarwx6m80ClR1TWKiT
X+cmzS+AvrFkgfsnVxbqGwv3UHedhV58TqvowhAW6iGW0ipFR6H/Wpxc1YPw/tGuZld8vNGU
Br2zTkbTq60s1EELfWXlk6GnZnoKHh9iofbqv7hcew3074PfH/hKeH9wV78+xlU3Wwb9HK1e
Ay1KWKhz1gz8PCwIetXdR/MzCO+37urFL65ZCdyhD/Ua5mDWxEIdl369m6E99Ed9Rr75SHj/
eleLO6gMVpkAvdlMnltZDzbqzTZZt8StoG9Wr8m9r8tGvWGG89VKY+gPx5W/l7Jkoz4gz+6Z
qQW9y8zaF7OmslF3O725YJEK9KcfSm+rIHw+RFdLsW5dNuVCn6/JoYSEs1Gbrl8q/VEK+kdu
tyQtNrBRB0SJ9rFu9obXN/T6VJ09bNRScf0PJlZB/5He55Yl4fNOuvrcHsm04nzol4tLBkXc
YqOeP5v97csh6B9aa6978oSN+tLUqxncROhLGZqMBZ/YqNXHb9yltBj6sy7LXijIcVAPYd8t
ZjpD/3jAWfpbZQ7qcO+P+h9HQy/LcQh6Sfw8pS6mGJscz9ODPj2kjiE3hoP6tVmDTYw89JWj
6osnOnJQ97ZwqhvY6gX/f3X9w+GLgRzUVz9kxxY+g76/z8otk1dxUJe+GcCdfgN6hRVjY+m7
OKiTDlQuu50JfdSnao+3JzioTZe9uWK4GfrDD5uNP97goB612aNoxRLoD+rZPFUmfN5eV3Ne
zE0qcIH+1qQ0B79mDmpL3TJq0xjo65Mrd7ygc1EPenOyL0cf+vCrz5Ni1LioPWZ9LuwhD71d
TPZQazMu6jeuS0W53zzh9e1192i9SVzU5/arH26uhl58aB8n8wVc1Moul65eL4DedXnvPL9l
XNTiC/nDVh2GPjPlQvr1zVzU46bXUI0SoI/o6f3V+igXtQG/tmdxqCfh7x2dPlV3lYvaZ2Se
63RHT8Lx5qMLTxA+n7irUyVU798cAX3j0meGaU1c1FGvti3Q6Q+9yBGvx6cYPNTKMhdlwxnQ
F1i8yHunzkPNquYcyv7oAdu/B4Pv21nwUJ9w4o14cg/6vJtOnJLpPNTTSo1yP52H/vhDWddF
fjzUjs+eKX5Ohd7KTaRg1Coe6vnxi4e+iIVexOidjkkqD7WXzhTlK17Qz9M+vH7CWR7qJ2Ny
MuMmQj+nn+HTNeU81OfOv68ZZgR9/7zdPerf8lBvTdbPfKoEPV8w3CBImo/6dM8MMX8R6Lm9
onqqEP7+SVeLn156q+7ZAtjf8aW1uN6Uj5rVWiQ++Rr0Yoaho54R/h5OV+tvP5iUfgj6+Piq
wG8+fNQ29kphtRug9/kyZoLpKj7qp1ylXZyF0HuLiJRtT+OjFk3d2TxgJvQfI30+ql7go1Z8
sXGhnjn0lhZGGUWEvz/W1fXny+R694F+tn2fN7sIf5+tq4MbTTK+SUIf91rzRCJDHvURqw2W
+S/dYflzDWo53F8edYBd8rVFt6DP0Jl1os5KHvWoOFkdpUzonzU5VdoR/p5kV2tO3Dg3PQF6
AxMz7/LF8qgn5ZZOVw6BnpFUYr+U8PdLu/pz/nyxqFnQJ5ma7B13XB61pNyj8SXm0IsMPWNh
WSSP+vaHU4NofaBvqL/eb1adPOobCf67DSTdCX9/iz8xSVoBtX7W4lirF26wfV8dfLZJVQH1
oSOm94bfgH502TfHxYS/T97VDWtklw84DP37+S8GqzkooJ5XPWQpZQP0E3ZlmH5YpIA6/Tbj
9PkA6AvnvXJ7mqSAWr31am/3qdDXnl16uvm4AurMhPgDrcbQP2wO1RhYooD6s17yqAgl6G+9
5GWuq1dAzXv8sebVt/lw/6UK7Fg0RdRf+NmLhj2GfkaS0beT/RVRN2S8bIjMhX52kSB7qbUi
atb1GMsjadAHfa4P83ZVRL2zIXxm/nLoC2JWj1garYg6+F22ZoEb9FLaq+gndyuiDrrB3n18
DPQ7RDZXMC4por58bfbx1VrQHw9PTF5TpYja1Nl+wlg69NxtSZN1WxVRnzIpcv9c7wrHo92v
lWjs0R31WL7r23XF0J+zkthXadod9YE7V24zj0HfGLnTtG5md9SWgStawxKgH3x717mei7qj
3vBmqEdxEPT+YqpaAcndUY8b7dWNNQ36zDWhMXXZ3VGnrFx30cQYempwv4KVd7ujHraHs8pO
EfrgSc8+jGnujjolwn6Sbcs8+Pxx8xQJPQUl1LQneVTDB9DPLIz4amyshDrPe8teybPQ58jc
uTdvuhJqRmpP1qVt0AdNHbjtZIgS6iF5y81cw6GXXTDBckCyEupzT0QVG+2h/zpY4/bl00qo
1XOPbPIyh37i3UrB0vtKqNWe79lzuxf0x8OKE52+KP3BHyjrzgi3TtrucefXKdnOZyzKzwNp
0qRJkyZNmjRp0qRJkyZNmjRp0qRJkyZNmjRp0qRJkyZNmjRp0qRJkyZNmjRp0qRJkyZNmjRp
0qRJkyZNmjRp0qRJkyZNmjRp0qRJkyZNmjRp0qRJkyZNmjRp0qRJkyZNmjRp0qRJkyZNmjRp
0qRJkyZNmjRp0qRJkyZNmjRp0qRJkyZNmjRp0qRJkyZNmjRp0qRJkyZNmjRp0qRJkyZNmjRp
0qRJkyZNmjRp0qRJkyZNmjRp0qRJkyZNmjRp0qRJkyZNmjRp0qRJkyZNmjTp/7suo9CE32WE
X8O9A70C/CmSIiJrKQKhJYVfrcJBhvAb338Wo/xx6Cn8eioOXkcRbR/TBbGdP22mfD/7osBi
nWj7ZQ1j9CZcdtvQjcJoH88SjscJx6LCiksZzlBv/5kl/C5OkSdcL1VoprAmnmLJ6Lilkp2n
jGBwhd9t3LxcbFyc/AOU/X0Dnfxc/JXnuQVYjLOI6LwplL94Uyi/dVMoXW4KBb8pAlZEx5SS
Fl41hWLpFOCkrCf8fXXhNJ8gnOY2hMkr94sZ+391EC6l7dOobWBTdIXf/SRXy4RSTekdizCF
EkzRF361DYbtU65t/jkTVobtlGXC74xOz5aiUAKlvl96x5wT/PKaJYm3obXjujpOFyec5iy8
dn3C3BJ8v7DWrpfEoUgR5iuPoti51FEoop2nyxFW4p2UUZ3nfD/3+/hb5y1pJQzE3/zW+sdh
fftlEy+N++OUtnsjIfziU0yF32/eaBuUBNOMj10NypvYeW/6do4n/bAcRapbxzWKdF6zaOcl
vFkscblVYcaFDbUunOWqWZ2/YX2hfbQCLEc5Ik75MQ3EhLdDovMSfh6m/zx7LswhuOMSxNov
QVK4YklR0oRrZcf9kfuzxSpi9Bsr4S/Nj97WaIWsan/xkv7CAJf0D5b0n4ZfNQcjuj427KMM
6JyH2O90HdIpyp1z/s+Gv3a5Mr91uW31QcHP90HkL1+XyG9d11+/XNnfulzZX9wH0b98XaK/
dV1//XLlfuty5brcB5H/gmVJ5L9gWRL5L1iWRP8LliXR/4JlSfQ/dllSjYghnAZbHvFDX2wc
+mK3eZs6tj067nXH9NenuP2Y6waCn8d9BaL/cLmCgXjtC3+8rIn+8Qril4N57x+/3XFNyzvH
KzrHBykandsA2thldNwnXYu2QLVzMoj86caQVLcZXSaDLsXjD5OOOONEf/mq8V82GSi/MRla
keFXk0GUnAwxhNfmsDE9uW0oYwhgYxhZKQQ7ukwG6wsiv/Ei829NBsG/aaUQ/9Ol4Yj47y0N
8ArmP2xpkCAnQ8xPr9N/fn14wfzXKwVxMlijjw3/cc8U3f6lS8N/7EOk1P/1ySAfIddlA7I3
hQObxIR9vuss+rTvhJL5w8bw9/NVOneStu3qhUI5omd7N1iiVXoJjceYKdFHZibtPb1jx5yF
sKynRHXe/PuU5M6Z0krYbSXS5UVb21f9/9TuxJ92rnTcQ0b7bsbvp3MowT/t+v1rL0FE/mQx
URZ0TDNpyljJAJkN1H5UP8kkGS+qGHWGZIRMDLU7VVS4IP7+NNP9X5lm6hHrfuwYE2nffUe8
38aiXXch8nLLRHRPpgi+7xCktU+977+zc2fqj6/vg3T7tk6w8J+zsKvv8pQv1X5+10HjR7OV
oie8cHnCdBOnRPx0L8T/4Srd+n0PrsiPzSVx5D9Hug6bJX49+aR/3HzKH+4A8c5TIjr2SXbZ
N9kqbFo72+8/t34/ve1+wekXBB1fEcIvgUXnttiFn38mjttuoyhFXaDbPmHqJe1F9dp/+tbq
Ifp3VoU/24NJXBXy5DZQHYUPGy/lvKim9BmSxXIx1El0kX/bqlAg3FRwEz4qcuVgvIrWcXrb
+O+uCuKdm0+ind9hsem6KnzfN9636Lo6LTeFsCpgL6Gl25+Ffl4VLEb9f7AqiP5it3XXG/53
VoVf7aZPj+sYd96cVsL0au369X056fj6Z1cFOcr3VUGW8u9aFaQonhLy9LZn0vUSpvSOZ1JT
escz6b9rVRgrXOTlhZe/nQpjF3rH6S70v7MqdGxLSP+0o0v6x1IjsNDsvB8dWyPfH0jFoBD8
u551pdq3VLZIOtLbtlQWCqdt25ZK2/g/6Vn3r01fsf+F6StKkZG2kq6SGSGjLe0unSOjImMl
vUw6VUZauCmpL9Y5fZX/+vTVF2vfU/g/PH31fjxodkzBX+3iJWwt/vg9DkWn/R6GtXY8ZqwQ
nufbfj8NOov+Eawfr7CFq9ePR5oB7S9dO44E0Gq/yyKdt0Gk/cgNacqRQd//e1Sm/fgLbcJ/
cEtSFgi/9/9bcxUu98/mqoL0DXq5TA7DTLqenimTxJgsLcVIlokQTgJdkd+fq7oi/4lz9Qal
Y662zbRfzVWR9n/CV2CXYa62vR7TVw6Y7xLgpNU++b8fISLSPvukKVXa3ye/ePvzlFT7k9q/
dhbDdfzZLF4t/lU2TKKAekRcRW6aRBr1qrixnEAijvr3Z/Fq4Q0pEP5+2/ircBsrTALGbae3
7Y9J6xyrCO/qNAkYt51+VXh6XOfYWHi6QALGbadjszhK7O/O4lvfOmbxvW+/XnFFv6+4UT+v
uB0HVWlR9Nuvp9tfmMOS7bfyf2M++9BGyd2hN1HjaR5yx+il1AO0KLmt9BwqPECLq//OA7S4
+h3hRsIo4V1oGzcJL8eHBuO2048JT/foHJcKT4+nwbjt9K3C06M6x2234wANxlFy+HxeLfN3
5/PWzlX5GLIqi3WuyqIXfl6Vp+tNcgvwcNGiqLVfkQQyoyXbb9r//MyVowRIz5eRoU2UTZJe
LfOcqiO7TiZV5haVL1zplMV/f+tGWXgjTFs6GmycJN0xI55TYawj23F62/gf/f6//nFavHPm
ivyYneLts5NFuJwB7RruFDjXyd/NyUvZx8/bx9vDTbgKK7b/ptgf5uz3ucno3JZaIPzuSRlD
8Wq7lr89R2V+OpbyV3PUX1pW9hy1UXaTtI5sMrVYdq3MSNll1NP/xBw16Zzy2HhT55xLpsK4
WLbj9LbxP/r9f/0cleico3I/5qhE+xxl/jRH2zTCNcQn4P/PGSoviGg/7HOFyNL2Bw5NyjCK
x9b2C2nbdl9GUZb889eIf+XgCeK+y7YdSREREZTS0lKKo6Nju9vGtbW1P3Yy/ac0/40DOX/I
gRzI4XeG1xHf97fWUQRi8Oxp8Ce7jcmBHMjh10MdhSH+857N1v/CZ2eyIRuy+esNPLeKEp5b
ySlFNmTzd9YiMXItIhuy+SfXInHydR45kMPffJ33fS2SINciciCHf3ItkiTXInIgh39yLepG
rkXkQA5/a3gtiBBE/jiuJLHzw702UTZ37st37TxnhhBSX1tb294zMJHw+23rXtsbAPUG6ik7
tx136+Pt5hXgr+zk56LsHRjg7+bs0nbcprJTsJu/soebp1uAv56xsoufn7ef8hwnv45wroeb
j4+Ls7JTQJf257drtm7u/CGp/eCVtkNzRCjTCO8gGiDUb90U6u/cFq32Q4i/vzWr63Ev8ynj
2g9L8xBuGm9sP67ld4YkQXZE2zhb8KL9EtsPSBJuHTiKUdo/iStRAB8Ht/vHGwo7bkkqRalz
51DXN350WGABhRTl53f+d7y384JggpSU8OQZQU4eTcILncUTXuu9usTKsZd0OXmr0vnfpPV2
5qXrOOYMnp6jYLNC2pKlXdpff6fA5VCqQUyzuH0TY1aT2tC1hbozTzjdzZMfaLFi9rDybXUm
nktqwron8B/eEaXEr6XcDr19z7X6vrt1jxSL2aqWJw0aBdFfT9yNs1GkBEbY35VOH/ZFVrza
9AXXZ8O8s6mGd8ZzZJYfi6Xa5JRqBC1ir6afEb2ZWG6lZn54vqbDaOmqSlGfQlNuFj2dt/JK
aGzt10MpBqE9srjJN094Vu3LNNcs1ezDljRbWLfhIePqZ/6ggdoDdPP36uueHXls5pWrqSrf
wumbV+XrPAy+xq/0rw4bZphiwnoRfdz1ztd7cUEVwctsFWc13l+6cV5FRRhfY++Nmp7PTNW0
RrmX6itn3G7qfbrKJuxJ7KqrurPctlBdy4J6m8oyGwNHq+jVNF5xWxHkaDb4CHPJ2Zxts/Zd
jJHd0neL/SCz8UkR27+6NiT5LJ9tauoXWdY/1IFtFH56ttFd5pJR498PKah2Ch+UV1SbS8uZ
reTZZwL3de+RZZfV7kvFHd+W66bRV7xQJ8hrhkHWrSe31uw1HK/KcxmaNDawR1K0hFPeC7m3
s/t6ZvoNYmbln5Q2Z927bJ9oa3lTdJC059qkQbERzWO2HHh48o59mvfR1JNBDYIJoU9SN2xP
7ffCOlQ4OinZENbaek7kjtTWOzNcvJxZAqMfH63X9s6VcX7e7i5zA5TdvOZ5//Ij9ojHy5GP
qR2ffvf9I/b6dz5Y/fxhPn88ZI7Sfsjc7z909Bd4th8NCO9bbn8kz6r6fEfEk/H9QcOr/dpG
BPu4+Ll5ungFKFs6BbhQvNsfIvSNdHT0dfX1KR25SHtO+zm3thTG7feoIxJtj9oOAxzrHeAy
x9t7wR8SsfZECpafn88Wbz+b+tPVuPj93Ei0N9IdFxHgPdfb4+fzJdvPbz+h8/SVlL7wv9Bt
H1dgfvPGjaG/mm7wgGokEGt7Mxul9Z8cRAT/DwAA//8DAKQyclE=</Template></GraphPadPrismFile>
